# Fluorinated Phenylazopyrazoles for Monitoring the Photoisomerization in Complex Systems

**DOI:** 10.1002/chem.202501856

**Published:** 2025-07-11

**Authors:** Lukas Jakob, Jacob J. van der Wal, Radek Tovtik, Jakub Copko, Alessandro Iagatti, Mariangela Di Donato, Stefano Crespi, Nadja A. Simeth

**Affiliations:** ^1^ Department of Chemistry, Ångström laboratory Uppsala University, Regementsvägen 10 Uppsala 752 37 Sweden; ^2^ Institute for Organic and Biomolecular Chemistry, Department of Chemistry University of Göttingen, Tammannstr. 2 37077 Göttingen Germany; ^3^ LENS – European Laboratory for Non‐Linear Spectroscopy Via Nello Carrara 1, Sesto Fiorentino Firenze 50019 Italy; ^4^ INO‐CNR Via Nello Carrara 1 Sesto Fiorentino 50019 Italy; ^5^ ICCOM‐CNR Via Madonna del Piano 10 Sesto Fiorentino 50019 Italy; ^6^ Cluster of Excellence “Multiscale Bioimaging: from Molecular Machines to Networks of Excitable Cells” (MBExC) University of Göttingen 37075 Göttingen Germany

**Keywords:** bioorganic chemistry, photochemistry, photochromism, photoswitches, time‐resolved spectroscopy

## Abstract

Photoswitches have been impacting diverse areas of research, introducing means of dynamically regulating their environment through reversible photochemical isomerization. Among the different classes of photoswitches, phenylazopyrazoles (**PAP**s) stand out due to their facile synthesis, beneficial photochemical properties, and the long thermal stability of their metastable forms. Not surprisingly, they have been applied in different fields, from material sciences to pharmacology. However, once incorporated into complex systems, following the photoisomerization behavior of **PAP**s with routinely used analytical methods becomes challenging. In this work, we focused on synthesizing and studying the isomerization behavior of a series of differently substituted **PAP**s possessing two trifluoromethyl groups on the pyrazole moiety (**F‐PAP**s). By studying their isomerization with steady‐state and transient absorption spectroscopy, we found marked trends in the isomerization efficiency and photophysical properties of the different switches. Most importantly, these molecules can quantitatively isomerize between their stable and metastable forms, with relatively long lifetimes of thermal back relaxation. Leveraging these characteristics, we highlight in this work the potential application of **F‐PAP**s as photoswitchable ^19^F‐NMR probes to generate light‐responsive vesicles.

## Graphical abstract

Fluorinated azopyrazole photoswitches can be obtained in a straightforward synthesis on a wide range of substrates and isomerize efficiently under light‐irradiation. The molecular motion can be followed by 19F‐NMR spectroscopy even in complex systems, such as dopants in vesicles.

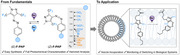

## 1 Introduction

Adaptation to external stimuli enables molecules and, by extension, entire systems to respond to a changing environment, resulting in “smart” behavior or life‐like features.^[^ ^1^, ^2^ ^]^ Such responses can be triggered, for instance, by alterations in pH, temperature, humidity, the addition or removal of ions, or illumination with light.^[^ ^1^ ^]^ Photons have been shown to induce fast, precise, controllable responses due to their high spatiotemporal resolution.^[^ ^2^, ^3^, ^4^, ^5^ ^]^ Small organic chromophores that undergo a photochemical reaction can be utilized to translate the energy of a photon into a molecular or system response. In this context, photochemical switches, or *photoswitches*, are frequently employed since they reversibly isomerize between two or more forms upon light absorption.^[^ ^4^, ^5^, ^6^, ^7^ ^]^ Moreover, a plethora of different types of photoswitches are available, allowing for the adjustment of molecular structures, isomerization mechanisms, UV‐Vis absorption, and photochemical properties, alongside the change in properties upon isomerization to fit the desired application.^[^ ^6^ ^]^ For example, diarylethenes,^[^ ^7^, ^8^ ^]^ fulgides,^[^ ^3^, ^5^, ^9^ ^]^ fulgimides,^[^ ^10^, ^11^ ^]^ and dihydroazulenes^[^ ^12^, ^13^, ^14^, ^15^ ^]^ undergo electrocyclic ring‐opening and closing reactions with a pronounced change in electronic properties and UV‐Vis absorption spectra, whereas spiropyrans^[^ ^16^ ^]^ undergo an electrocyclization followed by a double bond isomerization. Photoswitches isomerizing about double bonds, such as (stiff)stilbenes,^[^ ^17^ ^]^ imines,^[^ ^18^, ^19^, ^20^, ^21^ ^]^ or azobenzenes,^[^ ^22^ ^]^ are frequently used to affect the geometry of the system they are embedded in, capitalizing on the pronounced structural differences between *E* and *Z* isomers. Due to their beneficial properties, facile synthesis, and wide tunability, azobenzenes have emerged as the photoswitch of choice for many applications.^[^ ^22^, ^23^, ^24^ ^]^

In most cases, additional substituents must be attached to the photochromic core to incorporate the selected photoswitch into a molecule or system. The influence of substituents needs to be carefully studied and rationalized to predict the performance of the photochemical switch for a given application, as they affect the electronic structure of the switch and thus, its photophysical and photochemical properties.^[^ ^6^, ^22^ ^]^ Conversely, substituent effects can also be exploited to tune photoswitches' properties purposefully. For instance, push‐pull substituents have been used to shift bathochromically the UV‐Vis absorption spectra in azobenzenes.^[^ ^25^ ^]^ Additionally, the introduction of *ortho*‐substituents to the azo function^[^ ^26^, ^27^, ^28^, ^29^, ^30^ ^]^ or the cyclic diazocine^[^ ^31^, ^32^, ^33^, ^34^, ^35^ ^]^ analogues favors triggering the reversible photoisomerization via the visible‐light addressable nπ* transitions.

More recently, heteroaryl derivatives have been in the limelight: among those, phenylazopyrazoles (**PAP**s) have become increasingly popular over the past decade.^[^ ^36^, ^37^, ^38^ ^]^ In 2014, the team of Fuchter studied **PAP**s in detail, finding them to have high photoisomerization quantum yields (QYs, Ф), long thermal lifetimes (*τ*) of the metastable *Z* isomer, and to isomerize between both isomers quantitatively.^[^ ^39^ ^]^ These switches are available in large structural diversity and have been applied in host‐guest systems,^[^ ^40^ ^]^ surfactants,^[^ ^41^, ^42^ ^]^ in liquid crystals^[^ ^43^ ^]^ and hydrogels,^[^ ^44^ ^]^ as photopharmacophores,^[^ ^45^, ^46^ ^]^ and building blocks of biomacromolecules, such as peptides,^[^ ^47^, ^48^, ^49^ ^]^ to name a few examples.

**Scheme 1:**
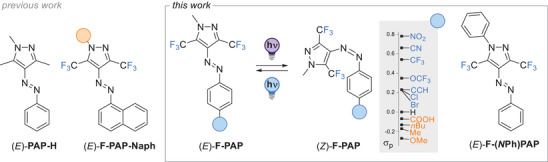
Photoswitching of **F‐PAP**s and derivatives used in this study.

While their impact on the field is undeniable, their precise control in complex systems, both in materials and biology, is challenged by the limited possibilities in following the photoisomerization process with analytical methods, such as UV‐Vis absorption, NMR spectroscopy, or high‐performance liquid chromatography (HPLC), due to the diversity of the media they are embedded in. To address this key limitation, we introduced fluorine atoms at a position that is typically not used for functionalization of **PAP**s, that is, the methyl groups on the pyrazole ring. Fluorines are rarely present in biology; they are NMR‐active and thus used as probes for biomolecules.^[^ ^50^, ^51^, ^52^, ^53^ ^]^ Consequently, this variation should allow to follow the photoisomerization of hexa‐fluorinated **PAP**s (**F‐PAP**s, Scheme 1) in complex, light‐controlled systems with simple NMR spectroscopy. While the groups of Matache and Khudina previously studied differently substituted hexafluoro **PAP**s (Scheme 1),^[^ ^54^, ^55^, ^56^ ^]^ they were, to the best of our knowledge, not yet investigated as photoswitchable ^19^F probes. Moreover, in this work, we thoroughly studied the photophysical and photochemical properties of a scope of differently substituted **PAP**s. To facilitate predictability for diverse applications, we also varied the substituents on the phenyl ring and synthesized the *N*‐Ph analogue before incorporating the switches in a fatty acid as an example for light‐controllable biomolecules.

## 2 Results and Discussion

### 2.1 Synthesis

**Scheme 2:**
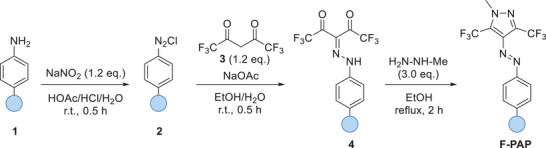
Synthesis of **F‐PAP**s from the corresponding anilines via diazonium salt formation and reaction with hexafluoroacetylacetone, followed by ring closure with methyl hydrazine. The sequence can be performed without intermediate purification to yield **F‐PAP**s in 3–46% isolated yields after a final purification step by column chromatography to provide sufficient material for further analysis.

We synthesized the **F‐PAP**s from the corresponding aniline (**1**) derivatives, adapting established protocols for **PAP**s (Scheme 2 and Supporting Information).^[^ ^38^, ^39^, ^40^ ^]^ Treatment with NaNO_2_ under acidic conditions generated the desired aryl diazonium salts **2**, which were directly added to hexafluoro acetylacetone **3** in a buffered ethanol/water mixture. An immediate color change indicated the formation of hydrazone **4**, which, after 30 minutes, was extracted from the reaction mixture and, after dissolving in ethanol, directly converted into the final **F‐PAP** with methyl hydrazine under reflux. The sequence worked reliably for substituents characterized by different Hammett‐Taft parameters^[^ ^57^ ^]^ (*cf*. Scheme 1), requiring only a final chromatographic purification step and affording isolated yields of the desired products between 3 and 46%. In addition to **F‐PAP**s with an *N*‐methyl pyrazole unit, we also introduced an *N*‐phenyl pyrazole heteroaromatic side of the azo function by replacing methyl hydrazine with phenyl hydrazine in the last synthetic step. Single crystals of **F‐PAP‐CCH** and **F‐PAP‐CN** (*cf*. Table S1, S2, and Figure S194) were grown from CD_3_CN solution in an NMR tube by either cooling down the solution in a fridge or slow evaporation, respectively. The crystal structure of the former compound shows a torsion angle (C‐N = N‐C) of 180.00(11)° and an NN‐bond length of 1.240(2) Å, while the latter compound shows a torsion angle of 178.91(14)° and an NN‐bond length of 1.235(2) Å. This is comparable to literature with similar values of 1.236(5) Å and a torsion angle of 179.88(17)° found on a mono‐CF_3_ pyrazole (CCDC: 259364).^[^ ^55^ ^]^

### 2.2 Photophysical and Photochemical Characterization

**Figure 1:**
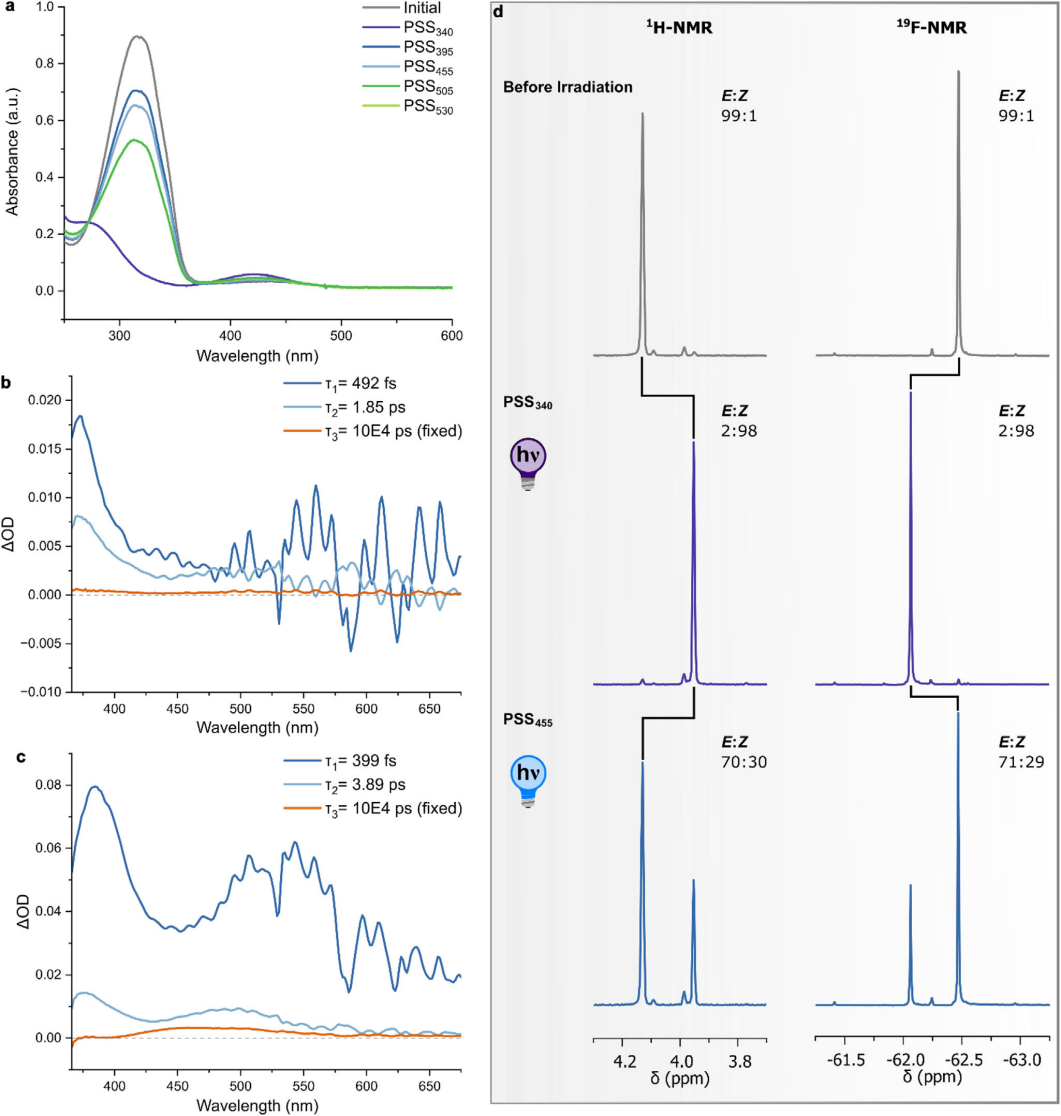
Photochemical characterization of **F‐PAP‐H** in MeCN. a) UV‐Vis absorption spectrum in the dark, at the PSS_340_, PSS_395_, PSS_455_, PSS_505_, and PSS_535_. b) Evolution‐associated difference spectra (EADS) of **F‐PAP‐H** in MeCN. c) EADS of **PAP‐H** in MeCN d) Excerpts of the ^1^H and ^19^F NMR in the dark and after irradiation with 340 and 455 nm light and distribution of *E* and *Z* isomers.

With the novel **F‐PAP**s in hand, we next studied the photophysical and photochemical properties of the compounds. The UV‐Vis spectra showcase the typical absorption profile of an azobenzene with two absorption bands associated with the slightly allowed S_0_→S_1_ nπ* and the more energetic and brighter ππ* (S_0_→S_2_, see Figure 1a and Table 1). Compared to **PAP‐H**, the UV‐Vis absorption maxima of the hexa‐fluorinated **F‐PAP‐H** are hypsochromically shifted by ca. 20 nm in the ππ* and 5 nm in the nπ* (see Table 1).^[^ ^39^ ^]^ Moreover, the reduced electron density of the chromophore core impacted the value of the molar extinction coefficient (ε) of the compound (19,000 in **F‐PAP‐H** vs. 22,700 M^−1^ cm^−1^ **PAP‐H** for the ππ* band at their respective maxima).^[^ ^39^ ^]^ We furthermore noticed that MeCN shifts the UV‐Vis absorption maximum of hexa‐fluorinated **F‐PAP‐H** bathochromically for the nπ* (5 nm) and hypsochromically for the ππ* (2 nm), in comparison to DMSO,^[^ ^54^ ^]^ whereas the ε of the ππ* band of the compound increases in MeCN (19,000 M^−1^ cm^−1^ vs. 17,000 M^−1^ cm^−1^ in DMSO).^[^ ^54^ ^]^ We noticed that the nπ* and ππ* bands are shifted in the opposite direction. This is likely because the ππ* transition is more sensitive to polarizability than polarity, and MeCN, being less polarizable than DMSO, induces a blue shift of the band. The nπ* is less sensitive to polarizability and overall undergoes a minimal red shift. Although MeCN is considered less polar than DMSO, given that these shifts are so minimal, we can conclude that there is hardly any solvent effect.

**Table 1:** Overview of photophysical and photochemical properties of **F‐PAP**s in MeCN.

Residue^[a]^	λ_max_ *E* [nm] [ππ*, nπ*]	λ_max_ *Z* [nm] [ππ*, nπ*]	ε *E* [M^−1^ cm^−1^] [ππ*, nπ*]	ε *Z* [M^−1^ cm^−1^] [ππ*, nπ*]	QY_340_ [*E*→*Z*, *Z*→*E*]	QY_455_ [*E*→*Z*, *Z*→*E*]	*τ* [h]^[b]^ 80 °C	PSD_340_ [*E*:*Z*] ^1^H, ^19^F‐NMR	PSD_455_ [*E*:*Z*] ^1^H, ^19^F‐NMR
NO_2_	325, 444	285, 426	24 900, 700	10,500, 1300	0.09±0.00, 0.12±0.01	0.09±0.01, 0.28±0.02	3.4	19:81, 19:81	83:17, 83:17
CN	318, 441	278, 426	24 200, 600	8600, 1200	0.13±0.00, 0.23±0.01	0.20±0.04, 0.37±0.03	2.45	2:98, 2:98	78:22, 78:22
CF_3_	310, 437	266, 421	21 300, 500	5700, 1100	0.14±0.00, 0.09±0.01	0.15±0.01, 0.28±0.02	4.31	2:98, 2:98	76:24, 76:24
OCF_3_	315, 427	276, 420	21 200, 700	5600, 1300	0.19±0.00, 0.13±0.00	0.18±0.04, 0.30±0.02	– ^[c]^	2:98, 1:99	74:26, 74:26
Br	329, 430	290, 422	24 300, 700	700, 1400	0.19±0.00, 0.14±0.02	0.20±0.01, 0.37±0.01	2.35	2:98, 3:97	73:27, 74:26
Cl	326, 430	285, 422	23 000, 700	5700, 1400	0.22±0.00, 0.15±0.01	0.18±0.02, 0.33±0.01	2.15	2:98, 2:98	74:26, 74:26
CCH	338, 437	300, 424	27 100, 900	6100, 1700	0.16±0.00, 0.35±0.01	0.20±0.02, 0.29±0.03	0.85	10:90, 9:91	76:24, 77:23
H	315, 420	273, 420	19 000, 600	5000, 1100	0.28±0.00, 0.17±0.02	0.16±0.00, 0.31±0.01	2.13	2:98, 2:98	70:30, 71:29
C_3_H_6_COOH	329, 424	293, 420	20 200, 600	5200, 1300	0.25±0.00, 0.39±0.01	0.16±0.02, 0.42±0.04	1.93	3:97, 3:97	72:28, 73:27
*n*Bu	330, 426	293, 423	23 500, 800	6200, 1600	0.24±0.00, 0.21±0.01	0.20±0.01, 0.31±0.01	1.70	3:97, 4:96	71:29, 71:29
Me	330, 426	292, 422	21 500, 700	5500, 1400	0.24±0.00, 0.13±0.01	0.18±0.00, 0.35±0.01	1.82	2:98, 2:98	71:29, 72:28
OMe	350, 430	320, 427	28 100, 1800	10 300, 2700	0.27±0.00, 0.18±0.00	0.23±0.03, 0.26±0.05	1.11	13:87, 13:87	69:31, 68:32
F‐(*N*Ph) PAP	318, 430	265, 422	21 700, 700	7800, 1300	0.20±0.00, 0.06±0.00	0.17±0.02, 0.30±0.01	1.41	1:99, 2:98	73:27, 73:27
PAP‐H^[^ ^39^ ^]^	335,425	296, 441	22 700, 960	5770, 2300	0.46±0.04^[d]^, –	–, 0.56±0.04^[e]^	0.29	2:98, ‐	82:18^[e]^, –

^[a]^ Residue R on the phenyl ring in para‐position to the azo function in order of σp Hammett‐Taft constant^[^ ^57^ ^]^ or compound name.

^[b]^ Lifetime of the Z isomer measured in toluene.

^[c]^ No relaxation observed, only decomposition.

^[d]^ Irradiation at 355 nm.

^[e]^ Irradiation at 532 nm.

We studied the photochemical isomerization of **F‐PAP‐H** by means of femtosecond transient absorption spectroscopy to understand the excited state dynamics of this azo switch. A UV‐light laser pulse (340 nm, 150 fs, 50 nJ) excited the ππ* transition of the molecule, promoting **F‐PAP‐H** into its excited state (Figure 1b and Supporting Information). The evolution‐associated difference spectra (EADS) obtained from global analysis show a short‐lived component with an excited state absorption peaking at 360 nm with sub‐ps lifetime (τ_1_ = 492 fs). This signal can be attributed to the motion away from the Franck‐Condon region and internal conversion to the S_1_.^[^ ^58^, ^59^, ^60^ ^]^ A second component with longer lifetimes (τ_1_ = 1.85 ps) and similar spectral shape (with the exception of an excited state absorption band appearing at 500 nm) is associated with the decay of the excited **F‐PAP‐H** to the ground state through isomerization and internal conversion. Finally, a long‐lived component with features comparable to the metastable state signaled the completion of the isomerization. These results are comparable to the parent **PAP‐H** (see Figure 1c); however, it is worth mentioning that the decay from the S_1_ in the fluorinated compound is twice as fast than the methylated one (1.85 vs. 3.89 ps), but still happening in the same time scales as recently reported by Schlücker and coworkers.^[^ ^58^ ^]^

Intriguingly, we noticed that the residual signal of the metastable state was more pronounced for **PAP‐H**, possibly indicating a more efficient isomerization. Indeed, steady‐state spectroscopy in MeCN revealed a QY Φ(*E*→*Z*, ππ*) of 28% for **F‐PAP‐H** compared to the 46% reported for **PAP‐H** (see Table 1).^[^ ^39^ ^]^ In general, the isomerization process can be followed by UV‐Vis absorption spectroscopy, which shows upon illumination a decrease in the ππ* transition of the *E* isomer around 320 nm, accompanied by an increase of the nπ* band of the *Z* form of **F‐PAP‐H** at *ca*. 420 nm (Figure 1a and Table 1). At the PSS_340_, we observed an isomer ratio of 2/98 *E*/*Z* both with ^1^H and ^19^F NMR analysis (Figure 1c and Table 1). These distributions are similar to what was observed upon irradiation of **PAP‐H** (excitation at 355 nm), showcasing that substitution of the CH_3_ by CF_3_ does not influence the eventual PSS.^[^ ^58^ ^]^ Subsequent irradiation with blue light (455 nm), favors the photodynamic equilibrium to the side of the *E* isomer, with a ratio of 70/30 *E*/*Z* at the PSS_455_ (^1^H NMR, 71/29 *E*/*Z* in ^19^F NMR analysis, Figure 1c and Table 1). Additionally, we checked for photofatigue of **F‐PAP‐H**, and after 20 cycles of *E*→*Z* isomerization (340 nm) and *Z*→*E* isomerization (455 nm), we observed no changes in the attained PSSs at the irradiation wavelength, indicating the robustness of F‐PAP switches (see Figure S32).

Quantitative formation of the *E* isomer through *Z*→*E* isomerization can be achieved thermally. The thermal lifetime (*τ*) of the metastable *Z* isomer plays an important role in determining the application of the photoswitch.^[^ ^61^, ^62^ ^]^ Due to the slow relaxation of **F‐PAP**s, the *τ* measurements were conducted in toluene at 80°C (Table 1). Replacing pyrazole CH_3_ groups with CF_3_ led to a nearly 10‐fold increase in lifetime (*τ* (from **PAP‐H** *τ* = 0.29 hours to **F‐PAP‐H** 2.13 hours). Strong EWG, as CN or NO_2_ groups, further increased the *τ* (**F‐PAP‐NO_2_** and **F‐PAP‐CN** 3.14 hours, 2.45 hours, respectively), while OMe showed opposite behavior (**F‐PAP‐OMe** 1.11 hours). Weaker EDG and EDG have smaller effects on the lifetimes. Compared to **PAP‐H**, we can observe at first glance a linear relationship of *τ* on the electronic effects for **F‐PAP**s.^[^ ^38^, ^63^ ^]^ Performing a Hammett analysis on our molecules (*cf*. Figure S101), a change in mechanism is indicated by a subtle change in slope. Notably, the CF_3_, CCH, and OCF_3_ derivatives deviate from this linear trend. **F‐PAP‐CF_3_** and **F‐PAP‐CCH** are outliers, while **F‐PAP‐OCF_3_** did not show any relaxation in toluene at 80°C upon irradiation. After several hours, we observed only decomposition. Replacing the pyrazole *N*‐methyl group with phenyl led to a slight decrease in *τ* (1.41 hours). The apparent effect of substituents on the ground state isomerization mechanism in **PAP**s, compared to a less pronounced trend in **F‐PAP**s, together with the large difference in lifetimes between the two subclasses, points to an in‐depth study being required to fully rationalize these observations.

As we expect substituents on the chromophore core structure, in particular, in *para*‐position to the azo‐function, for example, as demonstrated in **PAPs**,^[^ ^38^ ^]^ to influence the photophysical and photochemical properties of the switches, we also investigated the analogues **F‐PAP**s in detail in correlation to their Hammett‐Taft parameter (Table 1 and Supporting Information).^[^ ^57^ ^]^ We observed that both the *E* and *Z* isomers follow the same general trend for the *λ*_max_ in the ππ* band without noticing an immediate correlation, whereas the OMe and CCH seem to be clear outliers (Figure S98). Interestingly enough, in the nπ* band, we observe a clear V‐shaped correlation for the *E* isomer peaking at the H, where the CCH is an outlier (Figure S98). For the *Z* isomer, we see a similar trend and correlation, with a more pronounced bell‐curve character (Figure S98). It is interesting to notice that these “V‐shaped” Hammett plots are often found when correlating photophysical and photochemical properties of azobenzene.^[^ ^38^, ^64^ ^]^

**Figure 2:**
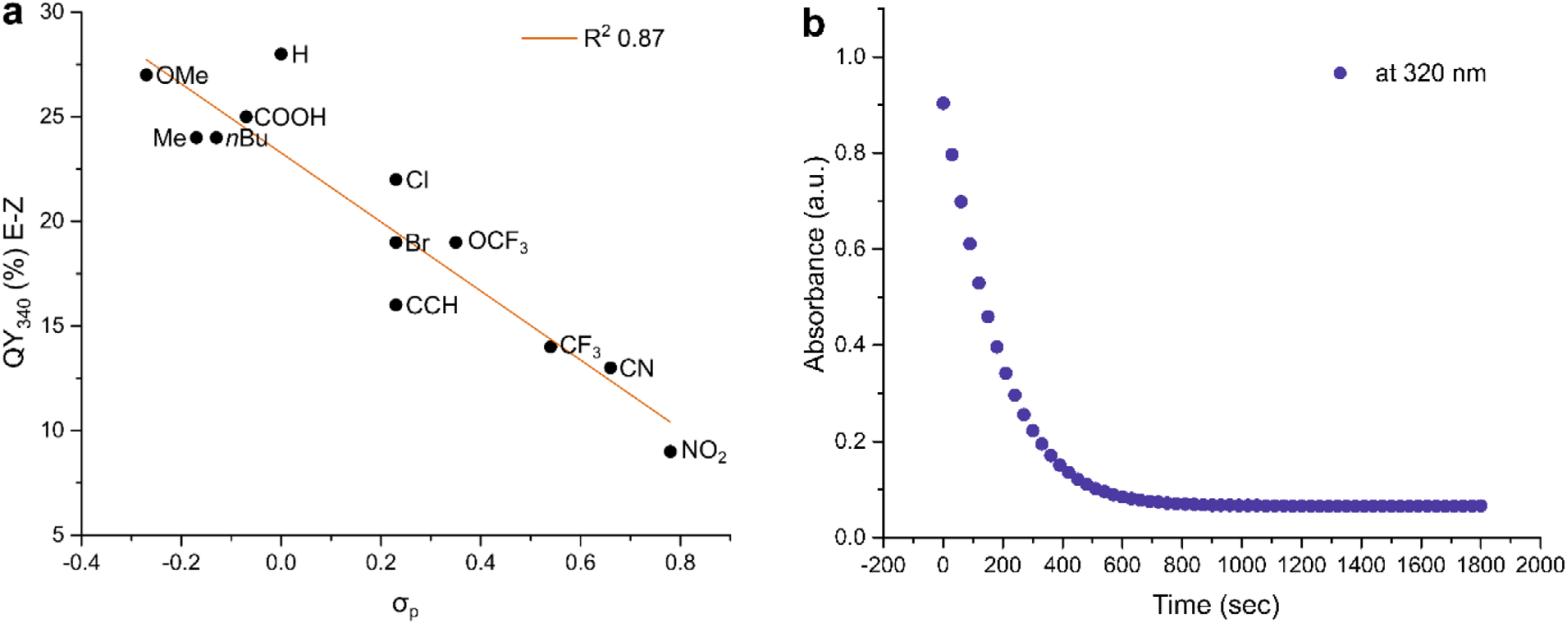
a) Correlation of the percentage of quantum yield_340 nm_ *E*→*Z* of **F‐PAP**s to the Hammett parameter (σ_p_), with the correlation shown in orange. b) Evolution of the wavelength at *λ* = 320 nm at PSS_340_, exemplarily shown for **F‐PAP‐H**.

Investigation of the Φ(*E*→*Z*, ππ) displays a clear effect of decreasing quantum yield with stronger electron‐withdrawing substituents (from OMe, 0.27, to NO_2_, 0.09, Figure 2). For Φ(*Z*→*E*, ππ*)_,_ This trend is less pronounced (from OMe, 0.18, to NO_2_, 0.12), where we observe the COOH, CCH, and CN substituents to be outliers with higher QYs (0.39, 0.35, and 0.23, respectively, see Table 1, Figure 2, and S100). Φ(*E*→*Z*, nπ*) and Φ(*Z*→*E*, nπ*) follow similar trends but are less marked between the electron‐donating and electron‐withdrawing substituents, and, in both cases, CN can be considered an outlier with higher QYs in both forward and backward photoreactions (0.20 and 0.37, respectively). While studying the *Z*→*E* nπ* photoreaction, we noted that OMe (0.26), COOH (0.42), and Br (0.37) are additional outliers (Table 1, Figure S100).

For the photostationary distribution (PSD) at 340 nm, no trend is observed in the Hammett‐Taft parameter, however, we see clear outliers in the NO_2_, CCH, and OMe substituents (*E*:*Z*, 2:98 vs. 19:81, 10:90, and 13:87, respectively) (Table 1, Figure S99). The PSD at 455 nm shows that the percentage of *E* isomer strongly depends on the electron density of the substituent, with increasing *E* isomer percentage for stronger electron‐withdrawing substituents (*E*:*Z* in OMe is 69:31 vs. 83:17 in NO_2_). It is interesting to notice that CCH seems to break the trends in every discussed parameter (see Table 1 and Figures S98–101). Comparison of the PSDs determined by ^1^H‐ and ^19^F‐NMR is in good agreement with each other, and in most cases, they are the same. For a few measurements, we found a deviation of 1%, highlighting the potential for PSD distribution using ^19^F‐NMR as an alternative to ^1^H‐NMR (vide infra).

### 2.3 Following the Isomerization of F‐PAPs in Vesicles

**Scheme 3:**
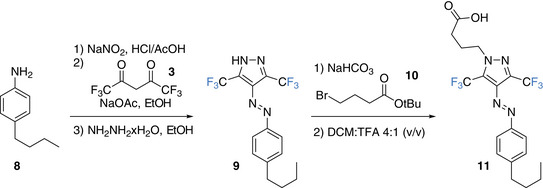
Synthesis of photo fatty acid **11** from diazo coupling of 4‐butylaniline with hexafluoro acetyl acetone, followed by ring closure with hydrazine and alkylation with bromo‐butane acid ester **10**, which was subsequently hydrolyzed.

Finally, we wanted to study the isomerization of **F‐PAP**s in a more complex system to evaluate whether standard ^19^F‐NMR would allow us to analyze the extent of photoisomerization. Taking inspiration from the impact of light‐responsive fatty acids and lipids on the properties of lipid bilayers,^[^ ^65^ ^]^ we decided to synthesize an **F‐PAP** analogue, fatty acid **11** (Scheme 3). Starting from 4‐butylaniline, we followed the same synthetic protocol as described above, however, replacing methyl hydrazine with hydrazine (*cf*. Supporting Information and Scheme 3). Alkylation with bromo‐butane acid ester **10** and subsequent hydrolysis gave photo fatty acids **11** in 12% yield over two steps.

**Figure 3:**
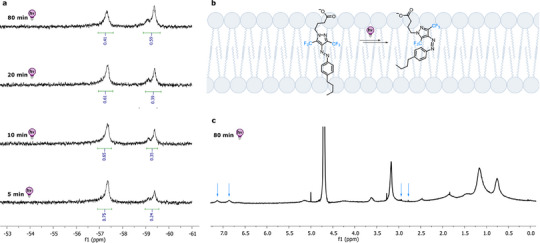
a) ^19^F NMR of photoswitchable fatty acid **11** as a dopant in a vesicle at different irradiation times (*E* isomer around ‐57.3 ppm, *Z* isomer around ‐59.3 ppm, integral normalized to 1.0). b) Illustration showing the *E*→*Z* photoisomerization of fatty acid **11** as a dopant in a lipid bilayer. c) ^1^H NMR of photoswitchable fatty acid **11** as a dopant in a vesicle after 80 minutes of UV light irradiation. Blue arrows point at signals indicative of the photoswitch.

The so‐obtained photoswitchable fatty acid **11** was incorporated into vesicles by mixing it with dioleoylphosphatidylcholine (DOPC) and cholesterol in a 15/32.5/2.5 (w/w/w) ratio in CHCl_3_, evaporating the solvent to generate a thin film, and dissolving the amphiphile mixture in D_2_O, which was basified with NaHCO_3_ (*cf*. Supporting Information). Then, NMR spectra of the vesicles containing the photoswitchable dopant were recorded after different intervals of irradiation with UV light (Figure 3a–c). The ^1^H NMR spectra showed signals in the aromatic range and singlets between 2.5 and 3.0 ppm, indicative of the isomers of the photoswitch, which were difficult to resolve (*cf*. blue arrows in Figure 3c as representative examples), while the ^19^F NMR was cleaner. Indeed, the photoswitching process could be straightforwardly followed by ^19^F NMR spectroscopy analyzing the changing ratio of the *E* and *Z* isomer peaks, respectively, found at ‐57.3 ppm and ‐59.3 ppm (Figure 3 and Supporting Information), underlying the potential of **F‐PAP**s as controllable photo manipulators in complex systems.

## 3 Conclusion

In this work, we have introduced a series of fluorinated phenyl azopyrazoles with varied substituents, which allowed us to fully characterize them photophysically and photochemically according to their Hammett‐Taft parameters. In particular, we could identify a dependency in the *λ*_max_ of the nπ* transition with the σ_p_ and a negative correlation between the QYs of isomerization and the increasing electron‐donating character in the substituents. While showcasing excellent QYs of isomerization and PSDs for *E*→*Z* photoreactions, these compounds are slightly less efficient than the nonfluorinated **PAP**s, as confirmed by transient absorption spectroscopy and quantum yield measurements. Moreover, they do not revert quantitatively to the stable form when irradiated on the nπ* transition. However, they show a substituent‐dependent trend allowing them to recover between 70% and 84% of the *E*‐form. Most importantly, we showed their potential as photoswitchable ^1^H‐ and ^19^F‐NMR probes by preparing the light‐responsive fatty acid **11** and following its photoisomerization as a dopant in a vesicle remotely by ^19^F‐NMR spectroscopy. These experiments underline the potential of **F‐PAP**s as precisely controllable photoswitches for incorporation in complex systems, allowing them to switch to defined isomer ratios using photons as reagents and precisely follow the isomerization process using NMR spectroscopy.

## Supporting Information

The authors have cited additional references within the Supporting Information.^[^ ^66^, ^67^, ^68^, ^69^, ^70^, ^71^ ^]^ Deposition Numbers 2453602 (for **F‐PAP‐CCH**),2 453 603 (for **F‐PAP‐CN**) contain(s) the supplementary crystallographic data for this paper. These data are provided free of charge by the joint Cambridge Crystallographic Data Centre and Fachinformationszentrum Karlsruhe Access Structures service.

## Conflict of Interest

The authors declare no conflict of interest.

## Supporting information



Supporting Information

## Data Availability

The data that support the findings of this study are available in the supplementary material of this article.
