# Supplementary material for: Fluorinated Phenylazopyrazoles for Monitoring the Photoisomerization in Complex Systems
Source: Chemistry. 2025 Jul 11;31(43):e202501856. doi: 10.1002/chem.202501856 (PMC12319390; doi:10.1002/chem.202501856)
Supplement: Supplementary file 1 — Supporting Information [file CHEM-31-e202501856-s001.pdf]

Table of contents

1. General Information ... 2

2. Chemical Synthesis ... 4

2.1. General Procedure for F-PAP Synthesis ... 4

3. Photophysical and Photochemical Properties of F-PAPs ... 20

3.1. Molar Absorption Coefficients ε ... 20

3.2. UV-Vis Spectra ... 21

3.3. Photochemical Isomerization in Transient UV-Vis Absorption Spectroscopy ... 28

3.4. Photochemical Isomerization in Steady-state UV-Vis Absorption Spectroscopy ... 30

3.5. Quantum Yield Determination ... 45

3.6. Photochemical Isomerization in ^{1}H and ^{19}F NMR Spectroscopy ... 59

3.7. Thermal *Z*-*E* relaxation ... 72

4. Hammett Substituent Parameter Analysis ... 80

4.1. λ_{max} Analysis of **F-PAPs** ... 80

4.2. Photostationary State Distribution ... 81

4.3. Quantum Yields ... 82

4.4. Lifetimes ... 83

5. Vesicle Formation and Manipulation ... 84

6. NMR Spectra ... 86

7. MS Data ... 131

8. X-Ray Data ... 139

9. References ... 142

1. General Information

Commercially available chemicals and solvents were used as received. Dry solvents were dried using activated 3 Å molecular sieves or from commercial sources.

Reactions were monitored by thin layer chromatography (TLC) using Merck TLC Silica Gel 60 F_{254} aluminium sheets or Merck TLC Aluminium oxide 60 F_{254} aluminium sheets and visualised by UV-light (λ = 365 nm or 254 nm). Chromatographic purification of products was accomplished using flash column chromatography (FC) on pre-packed silica gel columns from Silicycle 60 (0.04 – 0.063 nm/230 – 400 mesh) and reversed-phase flash column chromatography (RPFC) on a pre-packed C18 flash column from TELOS on an automated low-pressure Biotage SP4 using eluent gradients.

^{1}H, ^{13}C, ^{19}F-NMR spectra were recorded on a Q.One Quantum-I-Plus 400, JEOL JNM-ECZ400S/L1, Bruker Avance Neo 400, Avance III HD 400, Avance III 400 or Avance III HD 300 at ambient temperature. Chemical shifts (δ) are given in parts per million (ppm) relative to the residual solvent signal (for ^{1}H detection, δ = 7.26 ppm ($CDCl_3$), 1.94 (CD_3CN), 3.31 (CD_3OD), 2.50 (DMSO-d_6); for ^{13}C detection, δ = 77.16 ppm ($CDCl_3$), 118.26 ppm (CD_3CN), 49.00 (CD_3OD), 39.25 (DMSO-d_6). The splitting pattern of peaks is designated as follows: s (singlet), d (doublet), t (triplet), q (quartet), m (multiplet), br s (broad signal), or combinations of these signals. ^{13}C APT NMR is indicated with a (+) for positive or upwards pointing signals with an odd number of attached protons and is indicated (-) for negative or downwards pointing signals. Coupling constants (J) are given as absolute values in Hz.

LC-MS was performed either on an Agilent 1260 Infinity II combined with an InfinityLab LC/MSD iQ mass detector (ESI) using an InfinityLab Poroshell 120 EC-C18 column at 40 °C eluted with MeCN/H_2O + 0.1% formic acid or on an Agilent 1290 Infinity II HPLC system equipped with a 1290 Infinity II high-speed pump and a 1260 II Infinity DAD HS UV-Vis detector, using an InfinityLab Poroshell 120 EC-C18 column with dimensions of 50 mm x 2.1 mm and 1.9 µm particle size. The HPLC is coupled to an InfinityLab LC/MSD G6125B detector equipped with an ESI source for ionization. LC separation was performed with water (A, 0.05% formic acid): acetonitrile (B, 0.05% formic acid) eluent system using the methods LC1 and LC2. LC1: 1→10 min: 10% B; 1→10 min: 10%→90% B and 10–11 min: 90% B, 11–12 min: 10% B, Flow rate: 0.8 mL/min. LC2: 1→10 min: 10% B; 1→10 min: 10%→90% B and 10–11 min: 90% B, 11–12 min: 10% B, 0.3 mL/min. HR-MS has been recorded on a Bruker Daltonik microTOF spectrometer using ESI and Time-of-Flight (TOF) or on a Thermo Fisher Trace Ultra Gaschromatograph via electron ionisation (EI).

Absorption spectroscopy for the determination of thermal lifetimes were done on Avantes AvaLight-DH-S-BAL in quartz cuvettes (path 1.00 cm) at a controlled temperature of 20 °C. Irradiation was performed *ex situ* with suitable LED light sources from ThorLabs. Otherwise, UV-Vis irradiation experiments were performed in a screw-cap fluorescence cuvette (Hellma 117.100F-QS). Containing a 5 mm PTFE-coated stirring bar (VWR), using a home-built fibre-optic setup as reported in Volker *et al.*[1] Briefly: consisting of a Quantum Northwest Luma 40 Peltier-based temperature-controlled cuvette holder with four optical windows: an Avantes AvaLight-DH-S-BAL light source coupled with a 400 µm fibre (Avantes FC-UVIR400-1-BX) to an SMA-to-SM1 fibre adapter (Thorlabs CVH100-COL) containing a plano-convex lens (20.1 mm focal length, Thorlabs LA4647) in a 1-inch diameter lens mount (Thorlabs LMR1S/M) placed flush against one side; an Avantes AvaSpec-ULS2048CL-EVO-RS spectrometer coupled with a 400 µm fibre (Avantes FC-UVIR400-1-BX) to an SMA-to-SM1 fibre adapter (Thorlabs CVH100-COL) containing a plano-convex lens (20.1 mm focal length, Thorlabs LA4647) in a 1-inch diameter lens mount (Thorlabs LMR1S/M) placed flush against the opposite side; and a 365 nm LED (Thorlabs M365FP1) coupled with a 600 µm fibre (Thorlabs M114L01) to an adjustable fibre collimator (Thorlabs CFCS5-A) mounted in an adapter (Thorlabs AD15F2) in a 1-inch diameter lens mount (Thorlabs LMR1S/M) placed flush against one of the remaining optical ports (i.e. orthogonal to the spectrometer light path).

Quantum yields for PAPs were determined in acetonitrile following the procedure and instrumentation described in Volker *et al.*[1] The absorptivity of the PAP's metastable (*Z*-) isomer was determined by weighted subtraction of the stable (*E*-) isomer from the spectrum of PSS_{340nm} on the basis of the distribution experimentally obtained by NMR spectroscopy. The photon flux was measured as described in Volker *et al.*[1]

NMR *ex situ* irradiations were performed with a 340 and 455 nm LED from Thorlabs (M340F4, M455F3, using 600 mA and 1000 mA of current, respectively). The LEDs were used at their maximum current, resulting in the following powers: 0.78±0.00 mW, 13.75±0.01 mW and 15.75±0.01 mW, respectively.

For transient absorption measurements, we studied ***E*-F-PAP-H** and compared it with its nonfluorinated analogue ***E*-PAP-H**, synthesised following procedures adapted from the literature.[2] Transient measurements were performed employing a setup based on a Ti:Sapphire generative amplifier laser system (Coherent Legend Elite) coupled to a Ti:sapphire oscillator (Coherent Micra). The system produces 40 fs pulses centered at 800 nm with an average 3.3 W power and 1KHz repetition rate. The excitation wavelength at 340 nm was as the third harmonic generation of the signal output of a commercial Optical Parametric Amplifier (TOPAS, light conversion), pumped by the fundamental laser output. Excitation power was set at 30-50 nJ for all measurements. The pump beam polarization has been set to magic angle with respect to the probe beam by rotating a λ\2 plate, to exclude rotational contributions. The white light probe pulse was generated by focusing a small portion of the fundamental laser radiation on a 3 mm thick CaF_2 window. A portion of the generated white light was sent to the sample through a different path and used as a reference signal. After passing through the sample, the white light probe and reference pulses were both directed to a flat field monochromator coupled to a home-made CCD detector. Transient signals were acquired in a time interval spanning up to 100 ps. The sample was contained in a 2 mm quartz cuvette, mounted on a movable holder to minimize photodegradation. Measurements were performed at room temperature. Concentrations were adjusted to an absorbance of 0.9 – 1.0 OD (for the respective optical path) at the absorption maximum, which amounted to about 0.3 – 0.5 OD at the excitation wavelength. Before and after the measurements, the sample's integrity was checked on a PerkinElmer LAMBDA 950 spectrophotometer. The data was analyzed using singular value decomposition and global analysis, employing the software Glotaran 1.5.1.[3]

2. Chemical Synthesis

2.1. General Procedure for F-PAP Synthesis

The general procedure was adapted from published protocols.[4,5] The desired aniline (1.1 mmol, 1.0 eq.) was dissolved in AcOH (1.8 mL), and HCl (*conc*., aq., 330 µL) was added. This was followed by adding $NaNO_2$ (1.3 mmol, 82 mg, 1.2 eq.), dissolved in a minimal amount of H_2O. The reaction mixture was stirred under ambient conditions for *ca*. 30 min and then poured into a mixture of hexafluoro acetyl acetone (1.4 mmol, 180 µL, 1.3 eq.) and NaOAc (270 mg, 3.3 mmol, 3.0 eq.) in EtOH/H_2O (1.0 mL/600 µL). The mixture was stirred for *ca.* 30 min and then diluted with water (5 mL) and EtOAc (8 mL). The phases were separated and the aqueous phase was extracted with EtOAc (1×5 mL), and the combined organic phases were dried over anhydrous Na_2SO_4. The volatiles were removed *in vacuo*, and the crude material was analysed with LC-MS to confirm the identity of the hydrazone intermediates. Then, EtOH (5 mL) was added to the residue, and the solution was transferred into a crimp top vial. Next, methyl hydrazine (3.3 mmol, 174 µL, 3.0 eq.) was added, and the reaction mixture was refluxed for *ca.* 2 h. In the case of **F-(*N*H)-PAP-H** (see section 4.3.), hydrazine×H_2O (3.3 mmol, 102 µL, 3.0 eq.) and for **F-(*N*Ph)-PAP-H**, phenyl hydrazine (3.3 mmol, 324 µL, 3.0 eq.) were added instead of hydrazine. After cooling to room temperature, the volatiles were removed *in vacuo*, and the crude material was purified through automated flash column chromatography (2%→18%, EtOAc in *n*-pentane, SiO_2) using pre-packed flash cartridges (unless indicated otherwise).

Characterization Data

F-PAP-H: (*E*)-1-Methyl-4-(phenyldiazenyl)-3,5-bis(trifluoromethyl)-1*H*-pyrazole

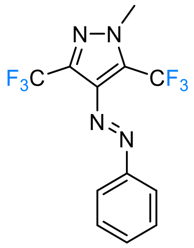

(*E*)-F-PAP-H
$C_{12}H_8F_6N_4$
322.21 g/mol

The crude material was purified by automated flash column chromatography (SiO_2, *n*-pentane:EtOAc, 2%→18% gradient) to afford the target compound (99.3 mg, 0.308 mmol, 28%) as an orange solid.

Analytical data:

TLC: R_f (*n*-pentane:EtOAc = 10:1) = 0.45 (yellow spot);

^{1}H-NMR (399.78 MHz, CD_3CN, 295 K): δ = 7.88 – 7.83 (m, 2H, Ar-*H*), 7.63 – 7.56 (m, 3H, Ar-*H*), 4.12 (s, 3H, NCH_3)* ppm;

^{13}C-NMR {^{1}H} (100.54 MHz, CD_3CN, 295 K): δ = 153.3, 137.5, 133.5, 131.7 (q, *J* = 39.5 Hz), 130.7 (q, *J* = 40.3 Hz), 130.5, 123.5, 121.6 (q, *J* = 267.8 Hz), 120.4 (q, *J* = 270.5 Hz), 41.4 (q, *J* = 3.1 Hz) ppm;

^{19}F-NMR (376.13 MHz, CD_3CN, 295 K): -58.31 (q, *J* = 1.2 Hz, 3F, CF_3), -62.42 (s, 3F, CF_3) ppm;

The NMR data is in accordance with previously reported literature.[6]

LRMS (pos. ESI): calcd for $C_{12}H_9F_6N_4$ ([M+H]$^+$) 323.07; found: 323.00;

HRMS (pos. ESI): calcd for $C_{12}H_9F_6N_4$ ([M+H]$^+$) 323.0722; found: 323.0726;

***(E-)* UV-Vis (MeCN, nm):** λ_{max} 315 (19000), 420 (600);

***(Z-)* UV-Vis (MeCN, nm):** λ_{max} 273 (5000), 420 (1100);

* A slightly broadened signal for the NCH_3 is observed, presumably due to weak coupling of the NCH_3 to the CF_3-Groups, which is not fully resolved in ^{1}H-NMR. This effect is also seen in the other derivatives.

F-PAP-Cl: (*E*)-4-((4-Chlorophenyl)diazenyl)-1-methyl-3,5-bis(trifluoromethyl)-1*H*-pyrazole

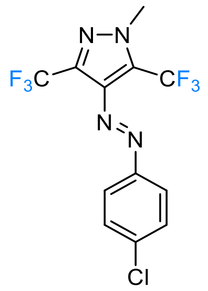

(*E*)-F-PAP-Cl
$C_{12}H_7ClF_6N_4$
356.66 g/mol

The crude material was purified by automated flash column chromatography (SiO_2, *n*-pentane:EtOAc, 2%→18% gradient) to afford the target compound (37.6 mg, 0.105 mmol, 10%) as a yellow solid.

Analytical data:

TLC: ***R_f*** (*n*-pentane:EtOAc =10:1) = 0.45 (yellow spot);

^{1}H-NMR (399.78 MHz, CD_3CN, 295 K): δ = 7.91 – 7.76 (m, 2H, Ar-*H*), 7.62 – 7.53 (m, 2H, Ar-*H*), 4.12 (s, 3H, NCH_3) ppm;

^{13}C-NMR {^{1}H} (100.54 MHz, CD_3CN, 295 K): δ = 151.8, 138.8, 137.3, 131.7 (q, *J* = 39.4 Hz), 131.1 (q, *J* = 39.9 Hz), 130.7, 125.1, 121.5 (q, *J* = 267.9 Hz), 120.4 (q, *J* = 270.6 Hz), 41.4 (q, *J* = 2.7 Hz) ppm;

^{19}F-NMR (376.13 MHz, CD_3CN, 295 K): -58.34 (q, *J* = 1.4 Hz, 3F, CF_3), -62.55 (s, 3F, CF_3) ppm;

LRMS (pos. ESI): calcd for $C_{12}H_8ClF_6N_4$ ([M+H]$^+$) 357.03; found: 357.00;

HRMS (pos. ESI): calcd for $C_{12}H_8ClF_6N_4$ ([M+H]$^+$) 357.0335; found: 357.0336;

(E-) **UV-Vis (MeCN, nm):** λ_{max} 326 (23000), 430 (700);

(Z-) **UV-Vis (MeCN, nm):** λ_{max} 285 (5700), 422 (1400);

F-PAP-Br: (*E*)-4-((4-Bromophenyl)diazenyl)-1-methyl-3,5-bis(trifluoromethyl)-1*H*-pyrazole

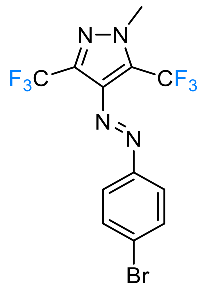

(*E*)-F-PAP-Br
$C_{12}H_7BrF_6N_4$
401.11 g/mol

The crude material was purified by automated flash column chromatography (SiO_2, *n*-pentane:EtOAc, 2%→18% gradient) to afford the target compound (53.7 mg, 0.134 mmol, 12%) as a yellow solid.

Analytical data:

TLC: ***R_f*** (*n*-pentane:EtOAc =10:1) = 0.38 (yellow spot);

^{1}H-NMR (399.78 MHz, CD_3CN, 295 K): δ = 7.81 – 7.68 (m, 4H, Ar-*H*), 4.12 (s, 3H, NC*H*$_3$) ppm;

^{13}C-NMR {^{1}H} (100.54 MHz, CD_3CN, 295 K): δ = 152.2, 137.3, 133.7, 131.7 (q, *J* = 39.5 Hz), 131.1 (q, *J* = 40.0 Hz), 127.3, 125.2, 121.5 (q, *J* = 268.2 Hz), 120.3 (q, *J* = 270.5 Hz), 41.4 (q, *J* = 2.7 Hz) ppm;

^{19}F-NMR (376.13 MHz, CD_3CN, 295 K): -58.34 (q, *J* = 1.3 Hz, 3F, C*F*$_3$), -62.56 (s, 3F, C*F*$_3$) ppm;

LRMS (pos. ESI): calcd for $C_{12}H_8BrF_6N_4$ ($[M+H]^+$) 400.98; found: 401.00;

HRMS (pos. ESI): calcd for $C_{12}H_8BrF_6N_4$ ($[M+H]^+$) 400.9831; found: 400.9826;

***(E-)* UV-Vis (MeCN, nm):** λ_{max} 329 (24300), 430 (700);

***(Z-)* UV-Vis (MeCN, nm):** λ_{max} 290 (6000), 422 (1400);

F-PAP-CN: (*E*)-4-((1-Methyl-3,5-bis(trifluoromethyl)-1*H*-pyrazol-4-yl)diazenyl)benzonitrile

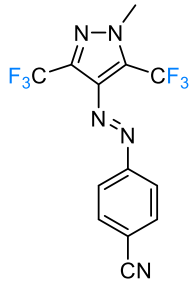

(*E*)-F-PAP-CN
$C_{13}H_7F_6N_5$
347.22 g/mol

The crude material was purified by automated flash column chromatography (SiO_2, *n*-pentane:EtOAc, 2%→18% gradient) to afford the target compound (46.7 mg, 0.134 mmol, 12%) as an orange solid.

Analytical data:

TLC: ***R*** R_f (*n*-pentane:EtOAc =10:1) = 0.32 (yellow spot);

^{1}H-NMR (399.78 MHz, CD_3CN, 295 K): δ = 7.97 – 7.89 (m, 4H, Ar-*H*), 4.14 (s, 3H, NCH_3) ppm;

^{13}C-NMR {^{1}H} (100.54 MHz, CD_3CN, 295 K): δ = 155.1, 137.1, 134.7, 131.9 (q, *J* = 40.0 Hz) 131.7 (q, *J* = 39.5 Hz), 124.1, 121.4 (q, *J* = 267.9 Hz), 120.2 (q, *J* = 270.7 Hz), 119.0, 115.9, 41.5 (q, *J* = 2.7 Hz) ppm;

^{19}F-NMR (376.13 MHz, CD_3CN, 295 K): -58.39 (q, *J* = 1.5 Hz, 3F, CF_3), -62.79 (s, 3F, CF_3) ppm;

LRMS (pos. ESI): calcd for $C_{13}H_8F_6N_5$ ($[M+H]^+$) 348.07; found: 348.10;

HRMS (pos. EI): calcd for $C_{13}H_7F_6N_5$ ($[M]^+$) 347.0600; found: 347.0603;

***(E-)* UV-Vis (MeCN, nm):** λ_{max} 318 (24200), 441 (600);

***(Z-)* UV-Vis (MeCN, nm):** λ_{max} 278 (8600), 426 (1200);

F-PAP-Me: (*E*)-1-Methyl-4-(*p*-tolyldiazenyl)-3,5-bis(trifluoromethyl)-1*H*-pyrazole

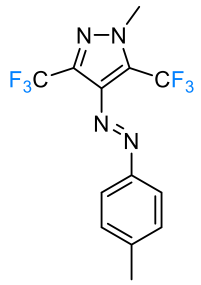

(*E*)-F-PAP-Me
$C_{13}H_{10}F_6N_4$
336.24 g/mol

The crude material was purified by automated flash column chromatography (SiO_2, *n*-pentane:EtOAc, 2%→18% gradient) to afford the target compound (90.3 mg, 0.269 mmol, 24%) as a yellow solid.

Analytical data:

TLC: ***R_f*** (*n*-pentane:EtOAc =10:1) = 0.55 (yellow spot);

^{1}H-NMR (399.78 MHz, CD_3CN, 295 K): δ = 7.93 – 7.67 (m, 2H, Ar-*H*), 7.48 – 7.28 (m, 2H, Ar-*H*), 4.11 (s, 3H, NCH_3), 2.43 (s, 3H, Ar-CH_3) ppm;

^{13}C-NMR {^{1}H} (100.54 MHz, CD_3CN, 295 K): δ =151.5, 144.6, 137.7, 131.7 (q, *J* = 39.0 Hz), 131.0, 130.4 (q, *J* = 39.5 Hz), 123.6, 121.6 (q, *J* = 267.8 Hz), 120.4 (q, *J* = 270.4 Hz), 41.3 (q, *J* = 2.8 Hz), 21.6 ppm;

^{19}F-NMR (376.13 MHz, CD_3CN, 295 K): -58.28 (q, *J* = 1.5 Hz, 3F, CF_3), -62.33 (s, 3F, CF_3) ppm;

The NMR data is in accordance with previously reported literature.[7]

LRMS (pos. ESI): calcd for $C_{13}H_{11}F_6N_4$ ([M+H]$^+$) 337.09; found: 337.00;

HRMS (pos. ESI): calcd for $C_{13}H_{11}F_6N_4$ ([M+H]$^+$) 337.0882; found: 337.0876;

(E-) **UV-Vis (MeCN, nm):** λ_{max} 330 (21500), 426 (700);

(Z-) **UV-Vis (MeCN, nm):** λ_{max} 292 (5500), 422 (1400);

F-PAP-CF$_3$: (*E*)-1-Methyl-3,5-bis(trifluoromethyl)-4-((4-(trifluoromethyl)phenyl)diazenyl)-1*H*-pyrazole

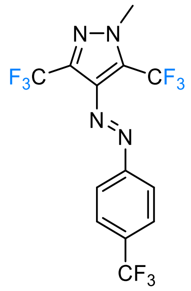

(*E*)-F-PAP-CF$_3$
$C_{13}H_7F_9N_4$
390.21 g/mol

The crude material was purified by automated flash column chromatography (SiO_2, *n*-pentane:EtOAc, 2%→18% gradient) to afford the target compound (21.9 mg, 0.0561 mmol, 5%) as a yellow solid.

Analytical data:

TLC: R_f (*n*-pentane:EtOAc =10:1) = 0.50 (yellow spot);

^{1}H-NMR (399.78 MHz, CD_3CN, 295 K): δ = 8.02 – 7.96 (m, 2H, Ar-*H*), 7.93 – 7.86 (m, 2H, Ar-*H*), 4.14 (s, 3H, NCH_3) ppm;

^{13}C-NMR {^{1}H} (100.54 MHz, CD_3CN, 295 K): δ = 155.2, 137.2, 133.5 (q, J = 32.8 Hz), 131.7 (q, J = 40.4 Hz), 127.7 (q, J = 3.9 Hz), 125.0 (q, J = 271.6 Hz), 124.0, 121.5 (q, J = 268.5 Hz), 120.3 (q, J = 270.7 Hz), 118.9, 41.5 (q, J = 2.8 Hz) ppm;

^{19}F-NMR (376.13 MHz, CD_3CN, 295 K): -58.38 (s, 3F, CF_3), -62.72 (s, 3F, CF_3), -63.22 (s, 3F, CF_3) ppm;

LRMS (pos. ESI): calcd for $C_{13}H_8F_9N_4$ ([M+H]$^+$) 391.06; found: 391.00;

HRMS (pos. EI): calcd for $C_{13}H_7F_9N_4$ ([M]$^+$) 390.0522; found: 390.0526;

***(E-)* UV-Vis (MeCN, nm):** λ_{max} 310 (21300), 437 (500);

***(Z-)* UV-Vis (MeCN, nm):** λ_{max} 266 (5700), 421 (1100);

F-PAP-OMe: (*E*)-4-((4-Methoxyphenyl)diazenyl)-1-methyl-3,5-bis(trifluoromethyl)-1*H*-pyrazole

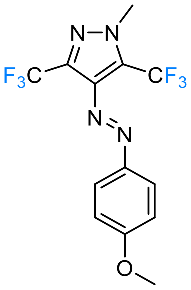

(*E*)-F-PAP-OMe
$C_{13}H_{10}F_6N_4O$
352.24 g/mol

The crude material was purified by automated flash column chromatography (SiO_2, *n*-pentane:EtOAc, 2%→18% gradient) to afford the target compound (27.0 mg, 0.0767 mmol, 7%) as a yellow solid.

Analytical data:

TLC: ***R$_f$*** (*n*-pentane:EtOAc =10:1) = 0.32 (yellow spot);

^{1}H-NMR (399.78 MHz, CD_3CN, 295 K): δ = 7.90 – 7.79 (m, 2H, Ar-*H*), 7.15 – 7.03 (m, 2H, Ar-*H*), 4.11 (s, 3H, NC*H*$_3$), 3.89 (s, 3H, OC*H*$_3$) ppm;

^{13}C-NMR {^{1}H} (100.54 MHz, CD_3CN, 295 K): δ = 164.4, 147.7, 137.8, 131.7 (q, *J* = 39.2 Hz), 129.9 (q, *J* = 39.5 Hz), 125.8, 121.7 (q, *J* = 267.8 Hz), 120.5 (q, *J* = 270.5 Hz), 115.6, 56.5, 41.3 (q, *J* = 2.6 Hz) ppm;

^{19}F-NMR (376.13 MHz, CD_3CN, 295 K): -58.24 (q, 3F, *J* = 1.3 Hz, C*F*$_3$), -62.20 (s, 3F, C*F*$_3$) ppm;

LRMS (pos. ESI): calcd for $C_{13}H_{11}F_6N_4O$ ([M+H]$^+$) 353.08; found: 353.10;

HRMS (pos. ESI): calcd for $C_{13}H_{11}F_6N_4O$ ([M+H]$^+$) 353.0832; found: 353.0828;

***(E-)* UV-Vis (MeCN, nm):** λ_{max} 350 (28100), 430 (1800);

***(Z-)* UV-Vis (MeCN, nm):** λ_{max} 320 (10300), 427 (2700);

F-PAP-OCF$_3$: (*E*)-1-Methyl-4-((4-(trifluoromethoxy)phenyl)diazenyl)-3,5-bis(trifluoromethyl)-1*H*-pyrazole

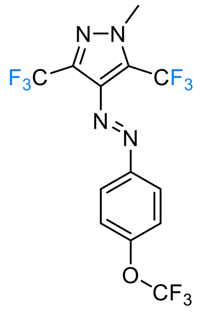

(*E*)-F-PAP-OCF$_3$
$C_{13}H_7F_9N_4O$
406.21 g/mol

The crude material was purified by automated flash column chromatography (SiO_2, *n*-pentane:EtOAc, 2%→18% gradient) to afford the target compound (173.5 mg, 0.427 mmol, 39%) as an orange solid.

Analytical data:

TLC: R_f (*n*-pentane:EtOAc =10:1) = 0.40 (yellow spot);

^{1}H-NMR (399.78 MHz, CD_3CN, 295 K): δ = 7.98 – 7.91 (m, 2H, Ar-*H*), 7.53 – 7.40 (m, 2H, Ar-*H*), 4.13 (s, 3H, NCH_3) ppm;

^{13}C-NMR {^{1}H} (100.54 MHz, CD_3CN, 295 K): δ = 152.5 (q, *J* = 1.7 Hz), 151.6, 137.2, 131.7 (q, *J* = 39.7 Hz), 131.2 (q, *J* = 41.6 Hz), 125.5, 122.7 (q, *J* = 1.4 Hz), 121.3 (q, *J* = 256.7 Hz), 120.3 (q, J = 270.5 Hz),118.9 (q, J = 268.8 Hz), 41.4 (q, *J* = 2.6 Hz) ppm;

^{19}F-NMR (376.13 MHz, CD_3CN, 295 K): -58.37 (q, 3F, *J* = 1.3 Hz, CF_3), -58.46 (s, 3F, CF_3), -62.59 (s, 3F, CF_3) ppm;

LRMS (pos. ESI): calcd for $C_{13}H_8F_9N_4O$ ($[M+H]^+$) 407.06; found: 407.10;

HRMS (pos. EI): calcd for $C_{13}H_7F_9N_4O$ ($[M]^+$) 406.0471; found: 406.0477;

***(E-)* UV-Vis (MeCN, nm):** λ_{max} 315 (21200), 427 (700);

***(Z-)* UV-Vis (MeCN, nm):** λ_{max} 276 (5600), 420 (1300);

F-PAP-CCH: (*E*)-4-((4-Ethynylphenyl)diazenyl)-1-methyl-3,5-bis(trifluoromethyl)-1*H*-pyrazole

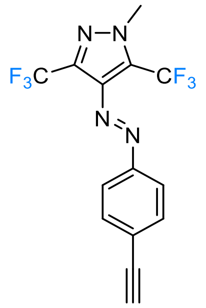

(*E*)-F-PAP-CCH
$C_{14}H_8F_6N_4$
346.24 g/mol

The crude material was purified by automated flash column chromatography (SiO_2, *n*-pentane:EtOAc, 2%→18% gradient) to afford the target compound (73.2 mg, 0.211 mmol, 19%) as an orange solid.

Analytical data:

TLC: ***R*** R_f (*n*-pentane:EtOAc =10:1) = 0.40 (yellow spot);

^{1}H-NMR (399.78 MHz, CD_3CN, 295 K): δ = 7.94 – 7.78 (m, 2H, Ar-*H*), 7.70 – 7.64 (m, 2H, Ar-*H*), 4.13 (s, 3H, NCH_3), 3.62 (s, 1H, Ar-CC*H*) ppm;

^{13}C-NMR {^{1}H} (100.54 MHz, CD_3CN, 295 K): δ = 152.9, 137.4, 134.1, 131.7 (q, *J* = 39.9 Hz), 131.2 (q, *J* = 39.9 Hz), 126.8, 123.7, 121.5 (q, *J* = 267.8 Hz), 120.34 (q, *J* = 270.6 Hz), 83.4, 82.0, 41.4 (q, *J* = 2.9 Hz) ppm;

^{19}F-NMR (376.13 MHz, CD_3CN, 295 K): -58.33 (q, 3F, *J* = 1.4 Hz, CF_3), -62.57 (s, 3F, CF_3) ppm;

LRMS (pos. ESI): calcd for $C_{14}H_9F_6N_4$ ($[M+H]^+$) 347.07; found: 347.00;

HRMS (pos. EI): calcd for $C_{14}H_8F_6N_4$ ($[M]^+$) 346.0648; found: 346.0643;

***(E-)* UV-Vis (MeCN, nm):** λ_{max} 338 (27100), 437 (900);

***(Z-)* UV-Vis (MeCN, nm):** λ_{max} 300 (6100), 424 (1700);

F-PAP-*n*Bu: (*E*)-4-((4-Butylphenyl)diazenyl)-1-methyl-3,5-bis(trifluoromethyl)-1*H*-pyrazole

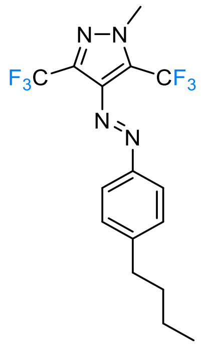

(*E*)-F-PAP-*n*Bu
$C_{16}H_{16}F_6N_4$
378.32 g/mol

The crude material was purified by automated flash column chromatography (SiO_2, *n*-pentane:EtOAc, 2%→18% gradient) to afford the target compound (152.6 mg, 0.403 mmol, 37%) as an orange solid.

Analytical data:

TLC: ***R_f*** (*n*-pentane:EtOAc =10:1) = 0.34 (yellow spot);

^{1}H-NMR (399.78 MHz, CD_3CN, 295 K): δ = 7.84 – 7.70 (m, 2H, Ar-*H*), 7.51 – 7.28 (m, 2H, Ar-*H*), 4.11 (s, 3H, NCH_3), 2.70 (t, *J* = 7.7 Hz, 2H, CH_2), 1.62 (tt, *J* = 7.6, 7.5 Hz, 2H, CH_2), 1.36 (qt, *J* = 7.4, 7.4 Hz, 2H, CH_2), 0.93 (t, *J* = 7.4 Hz, 3H, CH_3) ppm;

^{13}C-NMR {^{1}H} (100.54 MHz, CD_3CN, 295 K): δ = 151.7, 149.5, 137.7, 131.7 (q, *J* = 39.4 Hz), 130.4, 130.3 (q, *J* = 40.0 Hz), 123.7, 121.6 (q, *J* = 267.8 Hz), 120.5 (q, *J* = 270.3 Hz), 41.3 (q, *J* = 2.7 Hz), 36.1, 34.1, 23.0, 14.1. ppm;

^{19}F-NMR (376.13 MHz, CD_3CN, 295 K): -58.28 (s, 3F, CF_3), -62.31 (s, 3F, CF_3) ppm;

LRMS (pos. ESI): calcd for $C_{16}H_{17}F_6N_4$ ($[M+H]^+$) 379.14; found: 379.10;

HRMS (pos. ESI): calcd for $C_{16}H_{17}F_6N_4$ ($[M+H]^+$) 379.1352; found: 379.1346;

(E-) **UV-Vis (MeCN, nm):** λ_{max} 330 (23500), 426 (800);

(Z-) **UV-Vis (MeCN, nm):** λ_{max} 293 (6200), 420 (1600);

F-PAP-C_3H_6COOH: (*E*)-4-(4-((1-Methyl-3,5-bis(trifluoromethyl)-1*H*-pyrazol-4-yl)diazenyl)phenyl)-butanoic acid

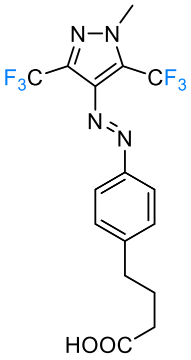

(*E*)-F-PAP-C_3H_6COOH
$C_{16}H_{14}F_6N_4O_2$
408.30 g/mol

The crude material was purified by automated flash column chromatography (SiO_2, DCM:MeOH, 1%→ 10% gradient) and subsequently with reverse-phase column chromatography (C_{18}, H_2O:ACN, 5%→ 100%) to afford the target compound (14.9 mg, 0.0365 mmol, 3%) as a yellow solid.

Analytical data:

TLC: ***R_f*** (DCM:MeOH =10:1) = 0.44 (yellow spot);

^{1}H-NMR (399.78 MHz, CD_3CN, 295 K): δ = 7.81 – 7.77 (m, 2H, Ar-*H*), 7.45 – 7.36 (m, 2H, Ar-*H*), 4.11 (s, 3H, NCH_3), 2.72 (t, *J* = 7.7 Hz, 2H, C*H*2), 2.29 (t, *J* = 7.5 Hz, 2H, C*H*2), 1.90 (tt, *J* = 7.9 Hz, 2H, C*H*2) ppm;†

^{13}C-NMR {^{1}H} (100.54 MHz, CD_3CN, 295 K): δ = 174.9, 151.8, 148.4, 137.6, 131.5 (q, *J* = 38.4 Hz), 130.5, 130.0 (q, *J* = 37.0 Hz), 123.7, 121.6 (q, *J* = 268.0 Hz), 120.4 (q, *J* = 269.4 Hz), 41.3 (q, *J* = 2.5 Hz), 35.5, 33.5, 27.1 ppm;

^{19}F-NMR (376.13 MHz, CD_3CN, 295 K): -58.34 (s, 3F, C*F*3), -62.39 (s, 3F, C*F*3) ppm;

LRMS (neg. ESI): calcd for $C_{16}H_{13}F_6N_4O_2$ ([M-H]$^-$) 407.09; found: 407.10;

HRMS (pos. ESI): calcd for $C_{16}H_{14}F_6N_4O_2Na$ ([M+Na]$^+$) 431.0913; found: 431.0907;

(E-) **UV-Vis (MeCN, nm):** λ_{max} 329 (20200), 424 (600);

(Z-) **UV-Vis (MeCN, nm):** λ_{max} 293 (5200), 420 (1300);

† The proton of the carboxylic acid could not be clearly assigned. There is a broad peak under the signals of the methylene groups between approx. 3.00 - 1.00 ppm. Together with the fact that there is no typical signal for the H_2O peak in acetonitrile (2.13 ppm), this indicates a rapid exchange between the carboxylic acid and H_2O.

F-PAP-NO$_2$: (*E*)-1-Methyl-4-((4-nitrophenyl)diazenyl)-3,5-bis(trifluoromethyl)-1*H*-pyrazole

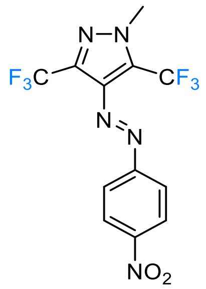

(*E*)-F-PAP-NO$_2$
$C_{12}H_7F_6N_5O_2$
367.21 g/mol

The crude material was purified by automated flash column chromatography (SiO_2, *n*-pentane:EtOAc, 2%→18% gradient) to afford the target compound (187.1 mg, 0.510 mmol, 46%) as an orange solid.

Analytical data:

TLC: ***R_f*** (*n*-pentane:EtOAc =10:1) = 0.25 (yellow spot);

^{1}H-NMR (399.78 MHz, CD_3CN, 295 K): δ = 8.57 – 8.24 (m, 2H, Ar-*H*), 8.22 – 7.80 (m, 2H, Ar-*H*), 4.15 (s, 3H, NCH_3) ppm;

^{13}C-NMR {^{1}H} (100.54 MHz, CD_3CN, 295 K): δ = 156.3, 150.6, 137.1, 132.2 (q, *J* = 40.0 Hz), 131.7 (q, *J* = 40.0 Hz) 124.4, 121.4 (q, *J* = 268.2 Hz), 120.2 (q, *J* = 270.7 Hz), 41.5 (q, *J* = 2.7 Hz) ppm;

^{19}F-NMR (376.13 MHz, CD_3CN, 295 K): -58.40 (q, 3F, *J* = 1.5 Hz, CF_3), -62.85 (s, 3F, CF_3) ppm;

LRMS (pos. ESI): calcd for $C_{11}H_5F_6N_5O_2$ ([M+H-CH$_3$]$^+$) 353.03; found: 353.10;

HRMS (pos. EI): calcd for $C_{12}H_7F_6N_5O_2$ ([M]$^+$) 367.0498; found: 367.0498;

(E-) **UV-Vis (MeCN, nm):** λ_{max} 325 (24900), 444 (700);

(Z-) **UV-Vis (MeCN, nm):** λ_{max} 285 (10500), 426 (1300);

F-(*N*Ph)PAP: (*E*)-1-Phenyl-4-(phenyldiazenyl)-3,5-bis(trifluoromethyl)-1*H*-pyrazole

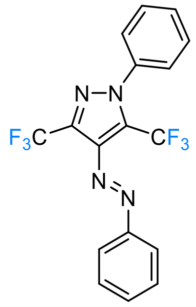

(*E*)-F-(*N*Ph)PAP
$C_{17}H_{10}F_6N_4$
384.29 g/mol

The crude material was purified by automated flash column chromatography (SiO_2, *n*-pentane:EtOAc, 2%→18% gradient) to afford the target compound (82.9 mg, 0.216 mmol, 20%) as an orange solid.

Analytical data:

TLC: R_f (*n*-pentane:EtOAc = 10:1) = 0.44 (yellow spot);

^{1}H-NMR (399.78 MHz, CD_3CN, 295 K): δ = 7.95 – 7.88 (m, 2H, Ar-*H*), 7.69 – 7.56 (m, 8H, Ar-*H*) ppm;

^{13}C-NMR {^{1}H} (100.54 MHz, CD_3CN, 295 K): δ = 153.4, 139.6, 138.1, 133.8, 133.7 (q, *J* = 39.5 Hz), 131.2 (q, *J* = 39.5 Hz) 130.6, 130.5, 127.2, 123.7, 121.6 (q, *J* = 268.5 Hz), 120.1 (q, *J* = 270.7 Hz) ppm;

^{19}F-NMR (376.13 MHz, CD_3CN, 295 K): -56.21 (s, 3F, C*F*$_3$), -62.54 (s, 3F, C*F*$_3$) ppm;

LRMS (pos. ESI): calcd for $C_{17}H_{11}F_6N_4$ ([M+H]) 385.09; found: 385.10;

HRMS (pos. ESI): calcd for $C_{17}H_{11}F_6N_4$ ([M+H]) 385.0882; found: 385.0877;

(E-) **UV-Vis (MeCN, nm):** λ_{max} 318 (21700), 430 (700);

(Z-) **UV-Vis (MeCN, nm):** λ_{max} 265 (7800), 422 (1300);

F-(*NH*)PAP-*n*Bu: (*E*)-4-((4-Butylphenyl)diazenyl-3,5-bis(trifluoromethyl)-1*H*-pyrazole (9)

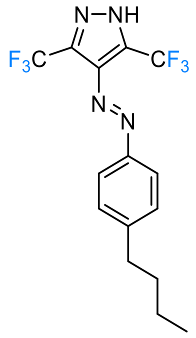

(*E*)-F-(*N*H)PAP-*n*Bu
$C_{15}H_{14}F_6N_4$
364.30 g/mol

The crude material was purified by column chromatography (SiO_2, *n*-pentane:EtOAc, 9:1) to afford the target compound (125 mg, 0.343 mmol, 31%) as an orange solid.

Analytical data:

TLC: ***R**f* (*n*-pentane:EtOAc = 9:1) = 0.50 (yellow spot);

^{1}H-NMR (400 MHz, CD_3CN, 295 K): δ 7.93 – 7.62 (m, 2H, Ar-*H*), 7.51 – 7.13 (m, 2H, Ar-*H*), 2.70 (t, *J* = 7.6 Hz, 2H, C*H*2), 1.69 – 1.57 (m, 2H, C*H*2), 1.43 – 1.29 (m, 2H, C*H*2), 0.93 (t, *J* = 7.3 Hz, 3H, C*H*3);‡

^{13}C-NMR {^{1}H} (101 MHz, CD_3CN, 295 K): 151.7, 149.6, 136.1, 130.5 (q, *J* = 38.4 Hz), 130.4, 123.6, 121.11 (q, *J* = 268.5 Hz), 36.2, 34.1, 23.0, 14.1;

^{19}F-NMR (377 MHz, CD_3CN, 295 K): δ -61.46 (s, 6F, 2×C*F*3);

HRMS (pos. ESI): calcd for $C_{15}H_{15}F_6N_4$ ($[M+H]^+$) 365.1195; found: 365.1191.

‡ The N*H*-proton could not be clearly assigned. There is a broad peak under the signals of the methylene groups between approx. 4.5 - 1.5 ppm. Together with the fact that there is no typical signal for the H_2O peak in acetonitrile (2.13 ppm), this indicates a rapid exchange.

F-(*N*nPrCOOH)PAP-*n*Bu: (*E*)-4-(4-((4-Butylphenyl)diazenyl-3,5-bis(trifluoromethyl)-1*H*-pyrazolyl)butanoic acid (11)

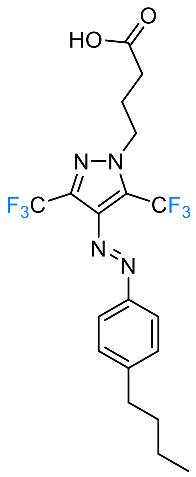

(*E*)-F-(*N*nPrCOOH)PAP-*n*Bu
$C_{19}H_{20}F_6N_4O_2$
450.39 g/mol

F-(*N*H)-PAP-*n*Bu (40 mg, 0.110 mmol) was dissolved in dry acetonitrile (3 mL), sodium bicarbonate (37 mg, 0.440 mmol) and *tert*-butyl 4-bromobutanoate (27 mg, 0.121 mmol) were successfully added. Reaction was refluxed for 12 hours after which the volatiles were removed under reduced pressure. Crude product was subjected to column chromatography (*n*-pentane:EtOAc, 95:5) yielding a mixture of crude *tert*-butylated product and *tert*-butyl 4-bromobutanoate in an 85:15 ratio. Crude mixture was dissolved in dry DCM (6.4 mL) and trifluoroacetic acid (1.6 mL) was added. Reaction was stirred at RT for 1 hour after which the volatiles were removed under reduced pressure and the crude product was purified by column chromatography (DCM:AcOH, 100:1) affording the target compound (20 mg, 0.045 mmol, 40%) as yellow solid as a mixture of *E* and *Z* isomers in 4:1 ratio. For pure NMR spectra of *cis* isomer, sample was heated in CD_3CN to 80°C for 48 hours yielding 95:5 ratio of *E*:*Z*.

Analytical data:

TLC: R_f (DCM:AcOH = 100:1) = 0.10 (yellow spot);

^{1}H-NMR (400.25 MHz, CD_3CN, 295 K): δ 7.82 – 7.70 (m, 2H, Ar-*H*), 7.43 – 7.36 (m, 2H, Ar-*H*), 4.45 (t, *J* = 7.1 Hz, 2H, C*H*₂), 2.70 (t, *J* = 7.8 Hz, 2H, C*H*₂), 2.42 (t, *J* = 7.1 Hz, 2H, C*H*₂), 2.18 (tt, *J* = 7.1 Hz, 2H, C*H*₂), 1.62 (tt, *J* = 7.5 Hz, 2H, C*H*₂), 1.36 (qt, *J* = 7.4 Hz, 2H, C*H*₂), 0.96 (t, *J* = 7.3 Hz, 3H, C*H*₃);[§]

^{13}C-NMR {^{1}H} (100.65 MHz, CD_3CN, 295 K): 174.1, 151.7, 149.5, 137.6, 132.1 (q, *J* = 39.2 Hz), 130.6 (q, *J* = 39.6 Hz), 130.4, 123.7, 121.6 (q, *J* = 267.7 Hz), 120.5 (q, *J* = 270.8 Hz), 53.4 (q, *J* = 2.5 Hz), 36.1, 34.1, 30.7, 25.5, 23.0, 14.1;

^{19}F-NMR (376.57 MHz, CD_3CN, 295 K): δ -57.57 (s, 3F, C*F*₃), -62.48 (s, 3F, C*F*₃).

HRMS (pos. ESI): calcd for $C_{19}H_{20}F_6N_4O_2Na$ ([M+Na]) 473.1383; found: 473.1381.

[§] The proton of the carboxylic acid could not be clearly assigned. There is a broad peak under the signals of the methylene groups between approx. 4.00 - 2.00 ppm. Together with the fact that there is no typical signal for the H_2O peak in acetonitrile (2.13 ppm), this indicates a rapid exchange between the carboxylic acid and H_2O.

3. Photophysical and Photochemical Properties of F-PAPs

3.1. Molar Absorption Coefficients ε

To evaluate the UV-Vis absorption profiles of our compounds and to determine their molar absorption coefficients ε, we measured the absorption of a stock solution of the *E*-isomer of our compounds in MeCN in three cuvettes with different path lengths (1.0, 2.0, and 10.0 mm). All measurements were performed in triplicate, the data were processed using Spectragryph (v1.2.15) and OriginPro2020, and the molar absorption coefficients ε were determined at the maximum (λ_{max}) of the bright ππ* transition. The absorptivity of the PAP's metastable (*Z*-) isomer was determined by weighted subtraction of the stable (*E*-) isomer from the spectrum of PSS_{340nm} based on the distribution experimentally obtained by NMR spectroscopy. The spectra obtained in MeCN for each compound are displayed below, together with the triplicate measurements for the ε.

3.2. UV-Vis Spectra

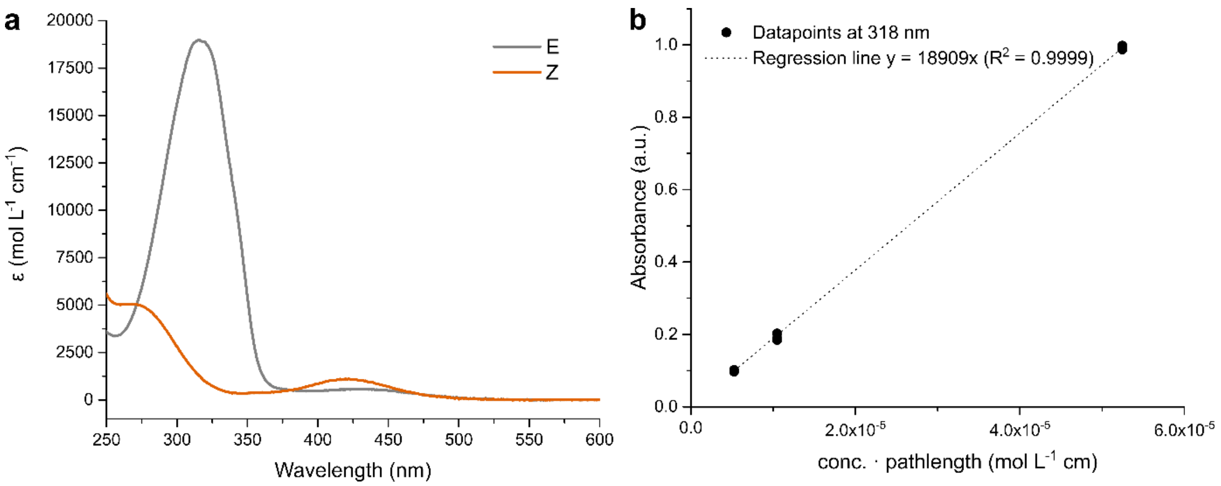

Figure S1: a The UV-Vis spectra of **F-PAP-H** (5.3x10^{-2} mM) in acetonitrile. **b** The regression analysis for the molar absorption coefficient (ε) in acetonitrile at 318 nm.

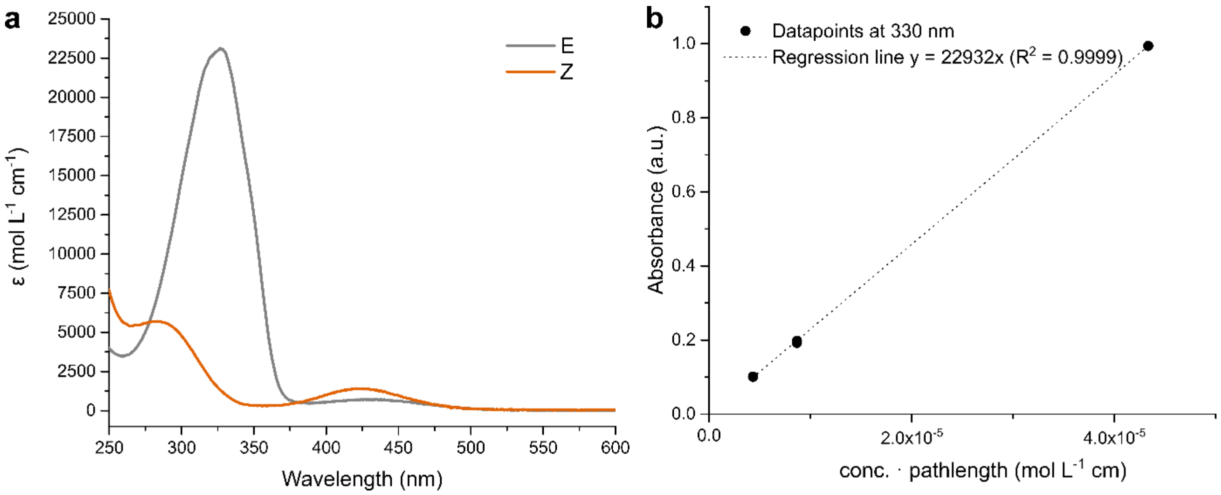

Figure S2: a The UV-Vis spectra of **F-PAP-Cl** (4.3x10^{-2} mM) in acetonitrile. **b** The regression analysis for the molar absorption coefficient (ε) in acetonitrile at 330 nm.

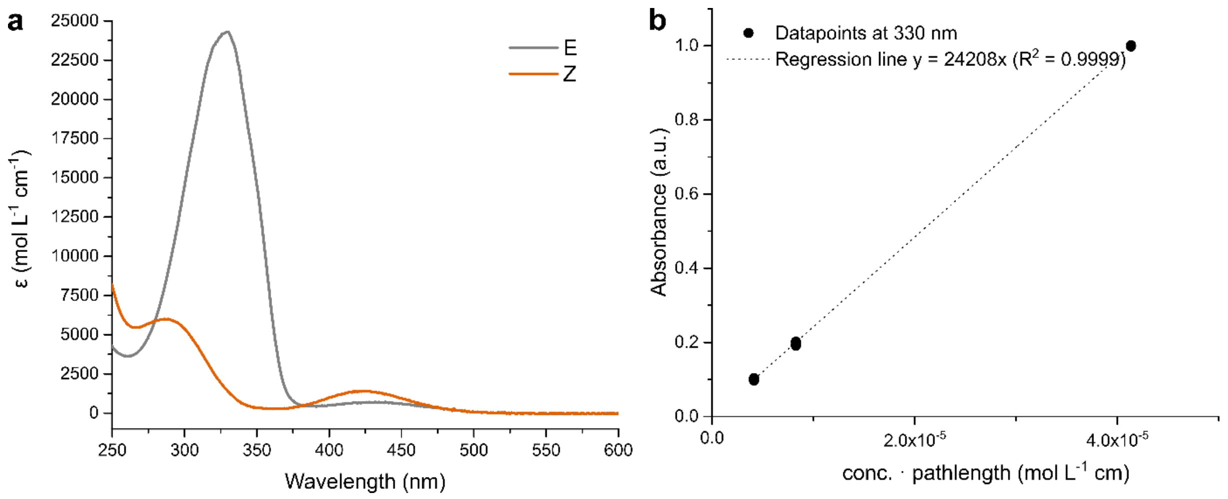

Figure S3: **a** The UV-Vis spectra of **F-PAP-Br** ($4.1x10^{-2}$ mM) in acetonitrile. **b** The regression analysis for the molar absorption coefficient (ε) in acetonitrile at 330 nm.

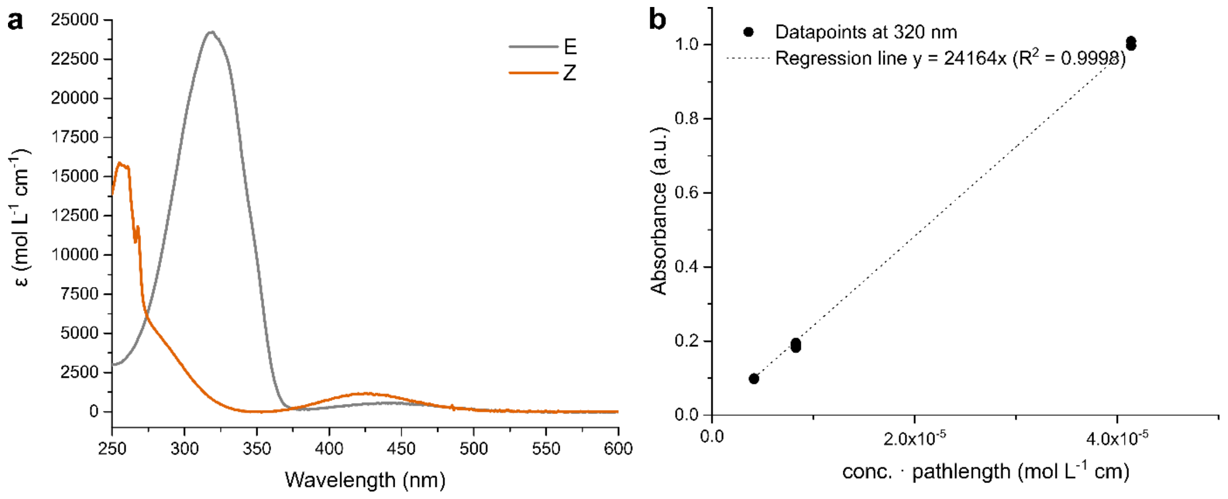

Figure S4: **a** The UV-Vis spectra of **F-PAP-CN** ($4.1x10^{-2}$ mM) in acetonitrile. **b** The regression analysis for the molar absorption coefficient (ε) in acetonitrile at 320 nm.

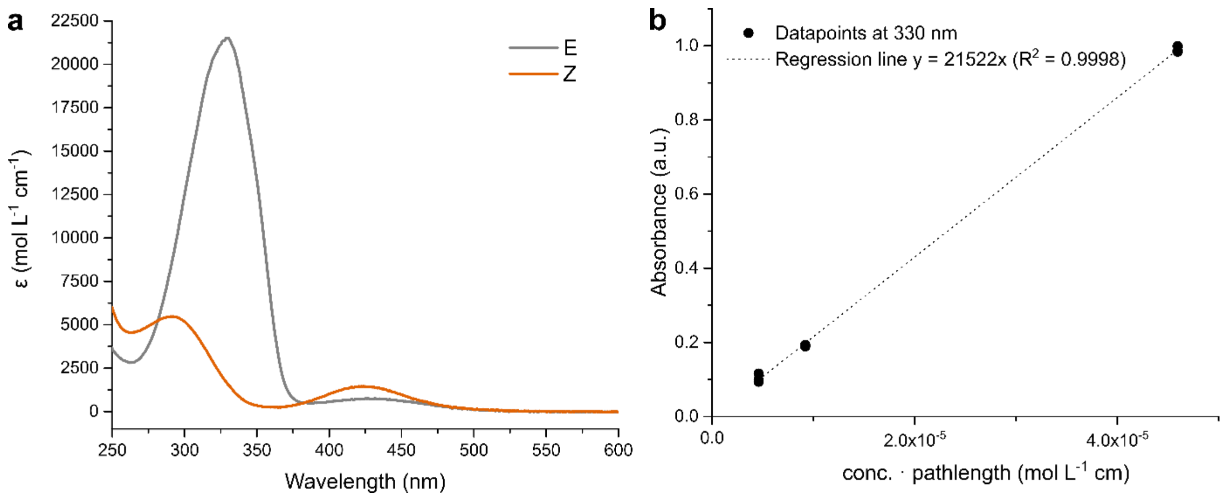

Figure S5: **a** The UV-Vis spectra of **F-PAP-Me** (4.6x10^{-2} mM) in acetonitrile. **b** The regression analysis for the molar absorption coefficient (ε) in acetonitrile at 330 nm.

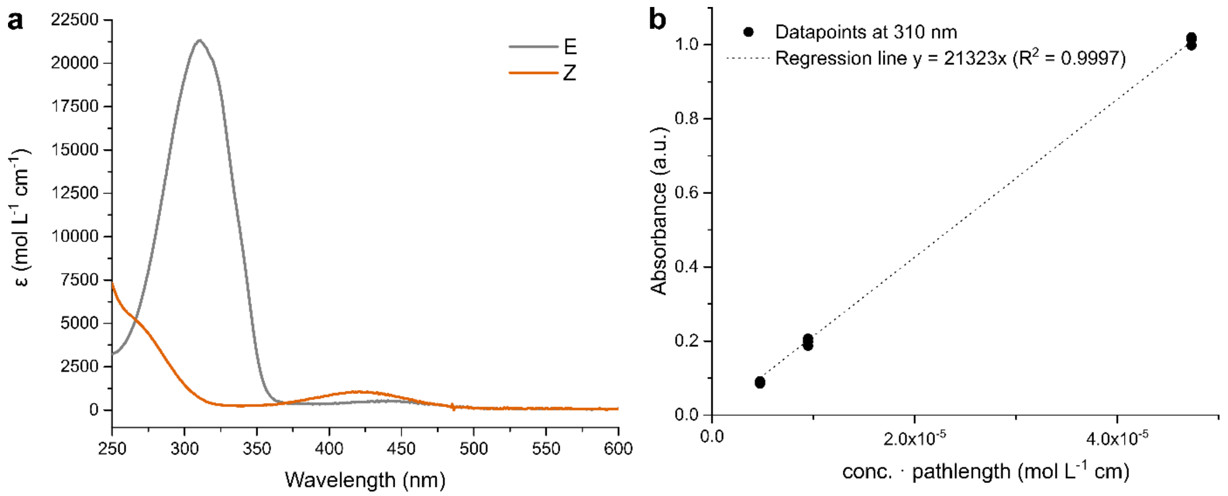

Figure S6: **a** The UV-Vis spectra of **F-PAP-CF$_3$** (4.7x10^{-2} mM) in acetonitrile. **b** The regression analysis for the molar absorption coefficient (ε) in acetonitrile at 310 nm.

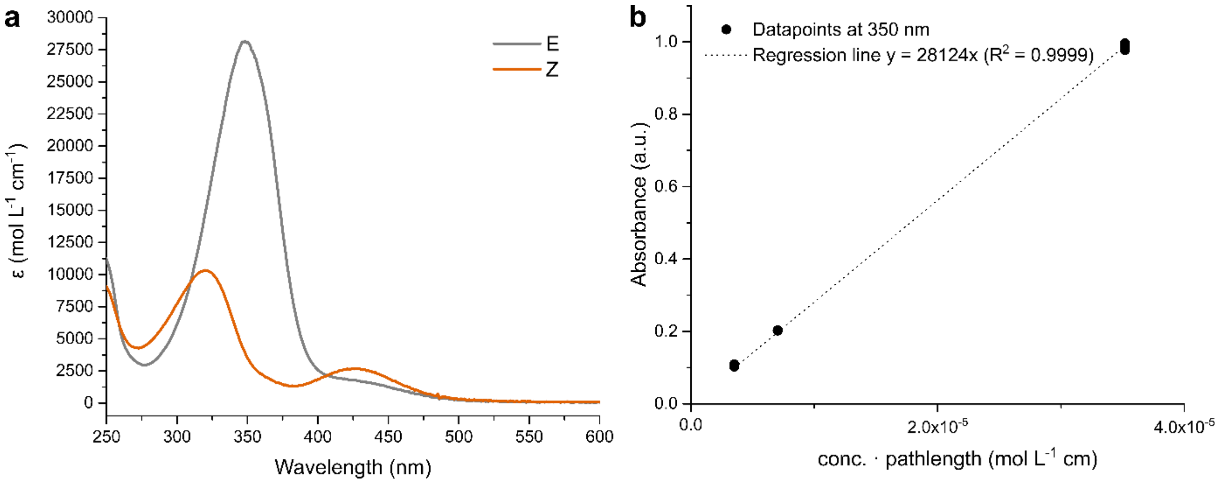

Figure S7: a The UV-Vis spectra of **F-PAP-OMe** ($3.5x10^{-2}$ mM) in acetonitrile. **b** The regression analysis for the molar absorption coefficient (ε) in acetonitrile at 350 nm.

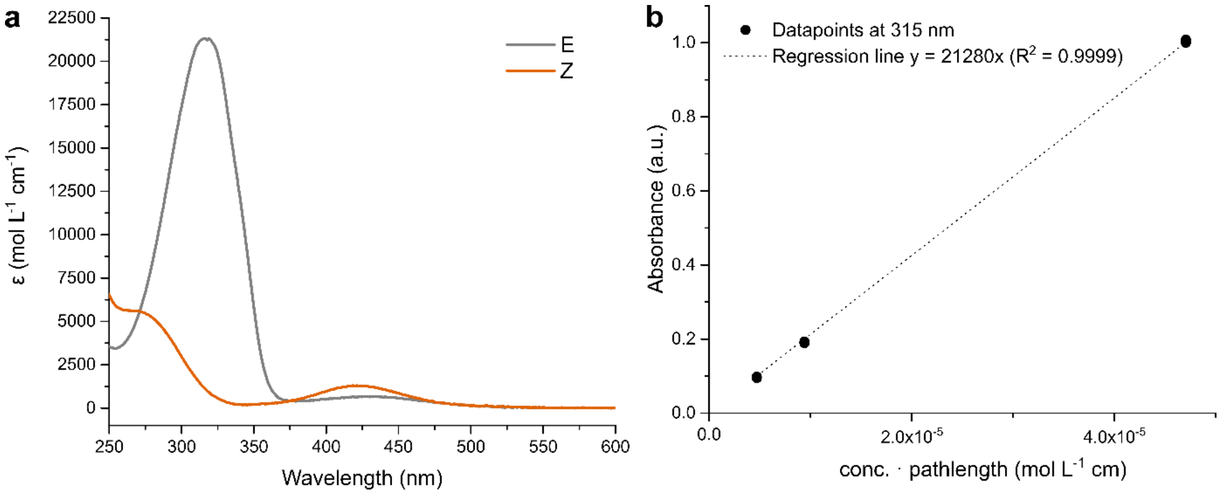

Figure S8: a The UV-Vis spectra of **F-PAP-OCF₃** ($4.7x10^{-2}$ mM) in acetonitrile. **b** The regression analysis for the molar absorption coefficient (ε) in acetonitrile at 315 nm.

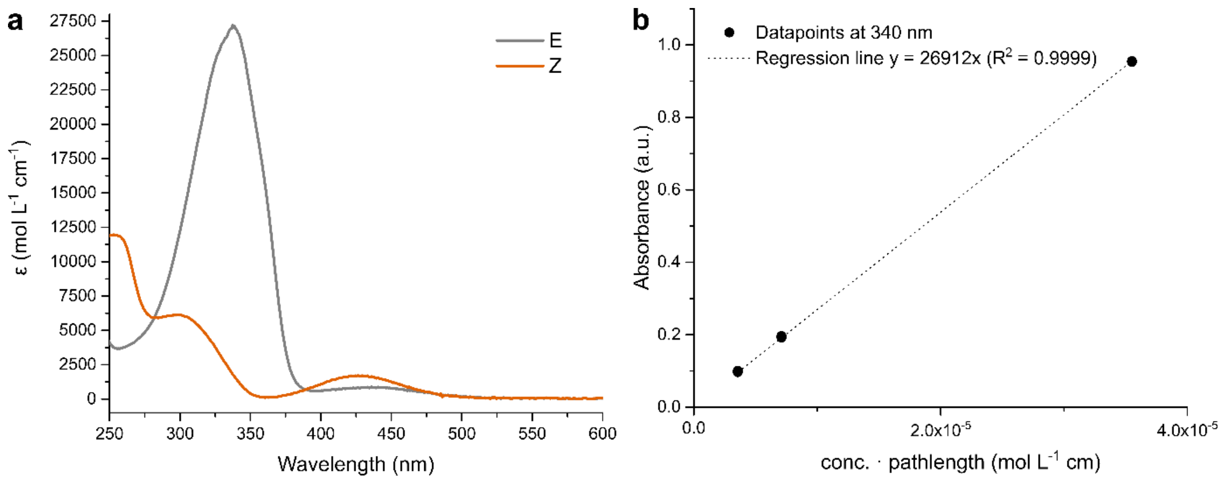

Figure S9: a The UV-Vis spectra of **F-PAP-CCH** ($3.6x10^{-2}$ mM) in acetonitrile. **b** The regression analysis for the molar absorption coefficient (ε) in acetonitrile at 340 nm.

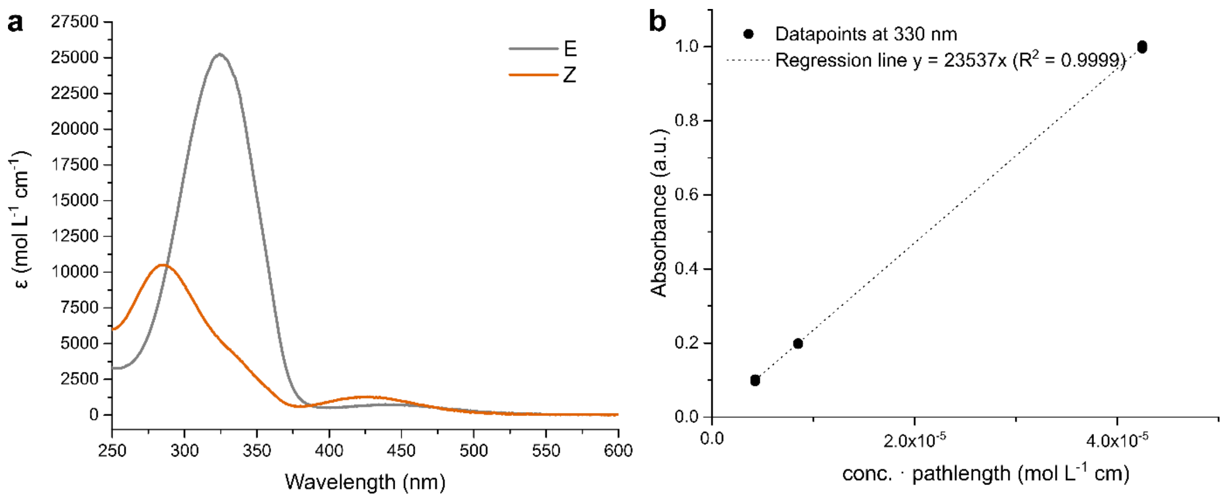

Figure S10: a The UV-Vis spectra of **F-PAP-*n*Bu** ($4.3x10^{-2}$ mM) in acetonitrile. **b** The regression analysis for the molar absorption coefficient (ε) in acetonitrile at 330 nm.

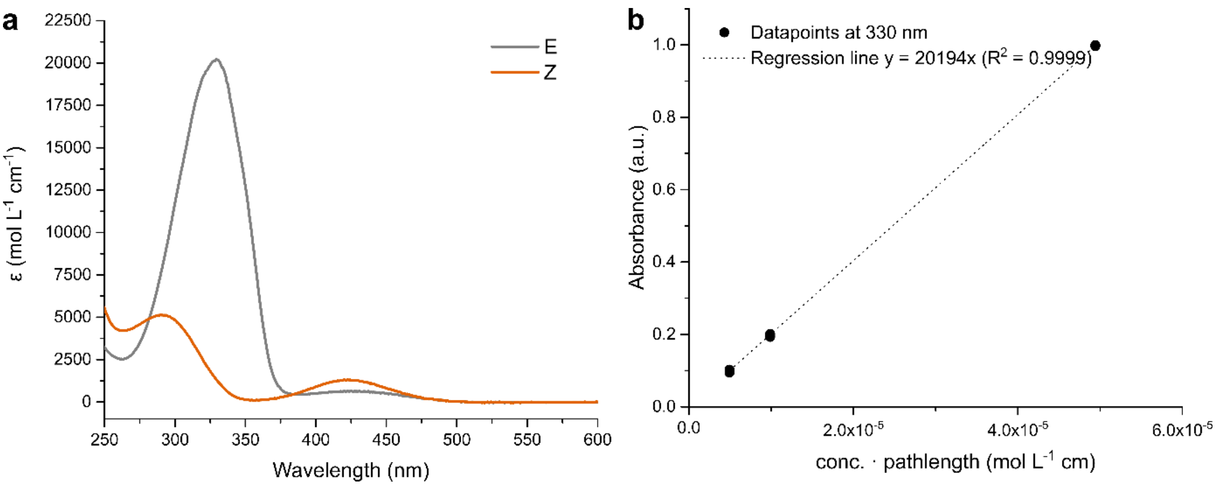

Figure S11: a The UV-Vis spectra of **F-PAP-C_3H_6COOH** ($5.0x10^{-2}$ mM) in acetonitrile. **b** The regression analysis for the molar absorption coefficient (ε) in acetonitrile at 330 nm.

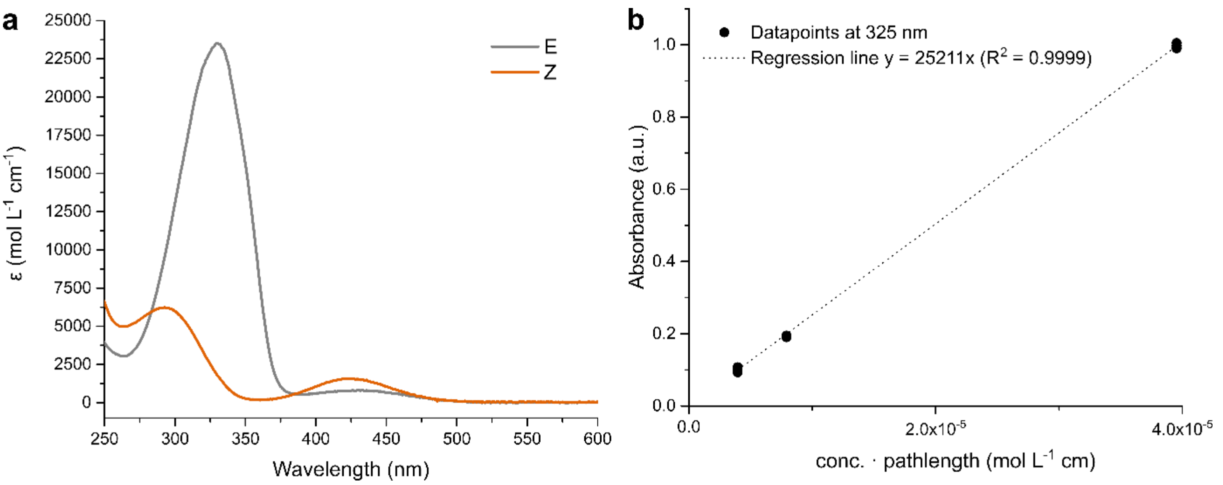

Figure S12: a The UV-Vis spectra of **F-PAP-NO_2** ($4.0x10^{-2}$ mM) in acetonitrile. **b** The regression analysis for the molar absorption coefficient (ε) in acetonitrile at 325 nm.

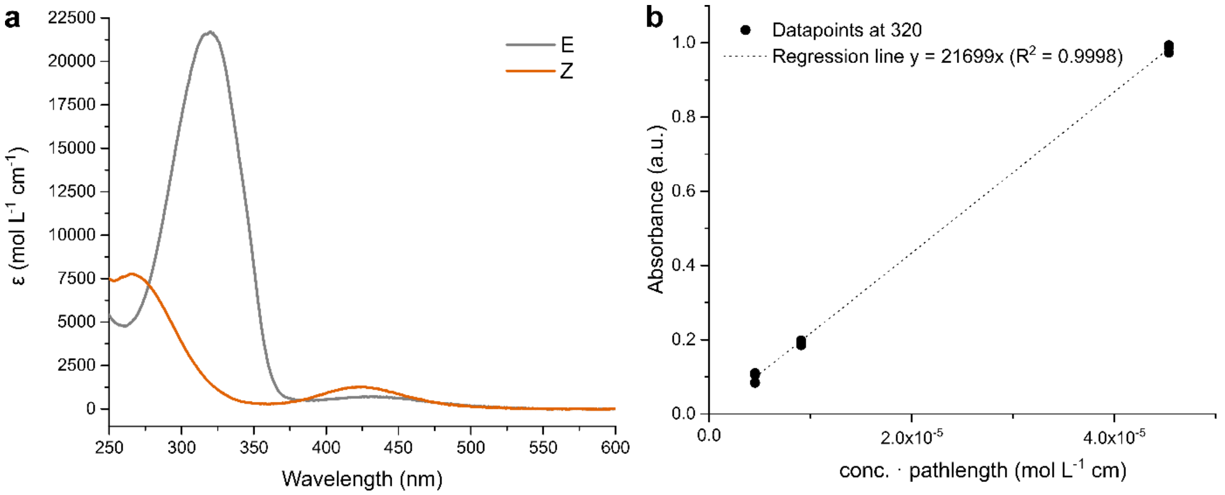

Figure S13: a The UV-Vis spectra of **F-(*N*Ph)PAP** (4.5x10^{-2} mM) in acetonitrile. **b** The regression analysis for the molar absorption coefficient (ε) in acetonitrile at 320 nm.

3.3. Photochemical Isomerization in Transient UV-Vis Absorption Spectroscopy

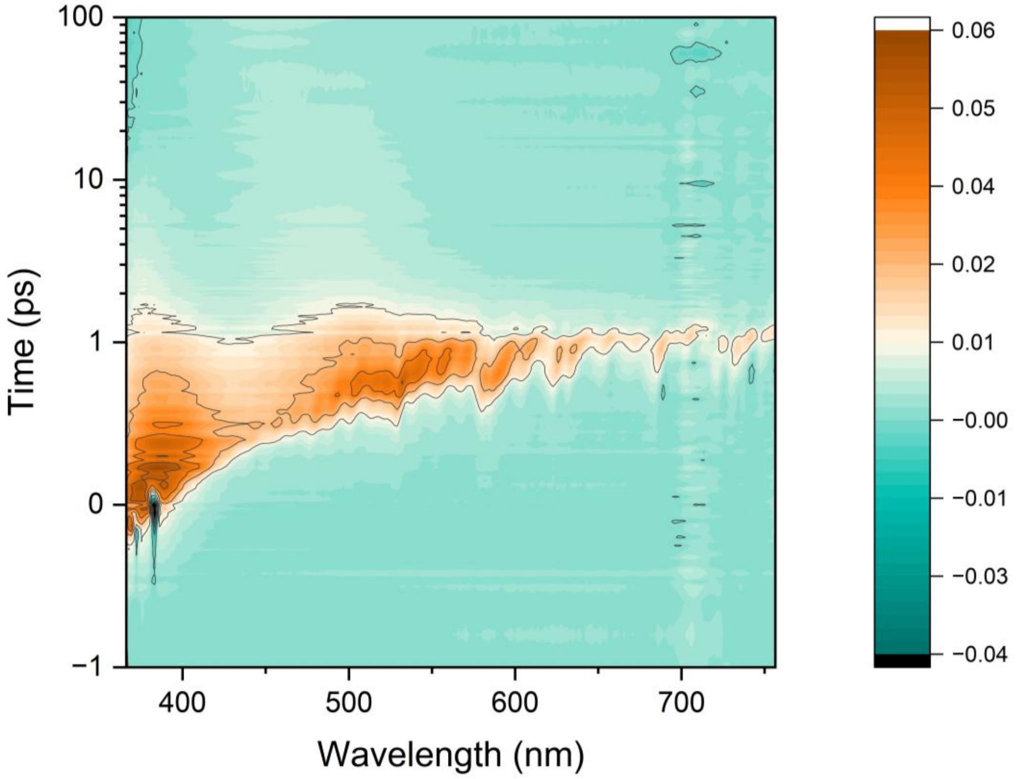

Figure S14: Transient absorption data recorded in acetonitrile for the non-fluorinated ***E*-PAP-H**.

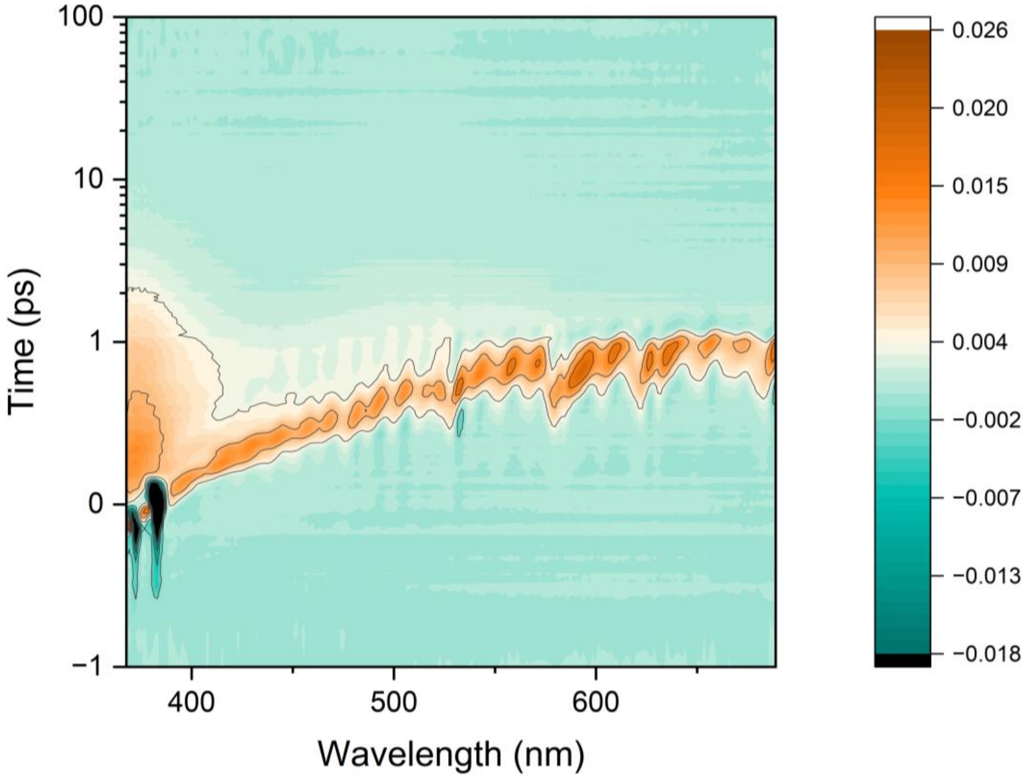

Figure S15: Transient absorption data recorded in acetonitrile for the fluorinated ***E*-F-PAP-H**.

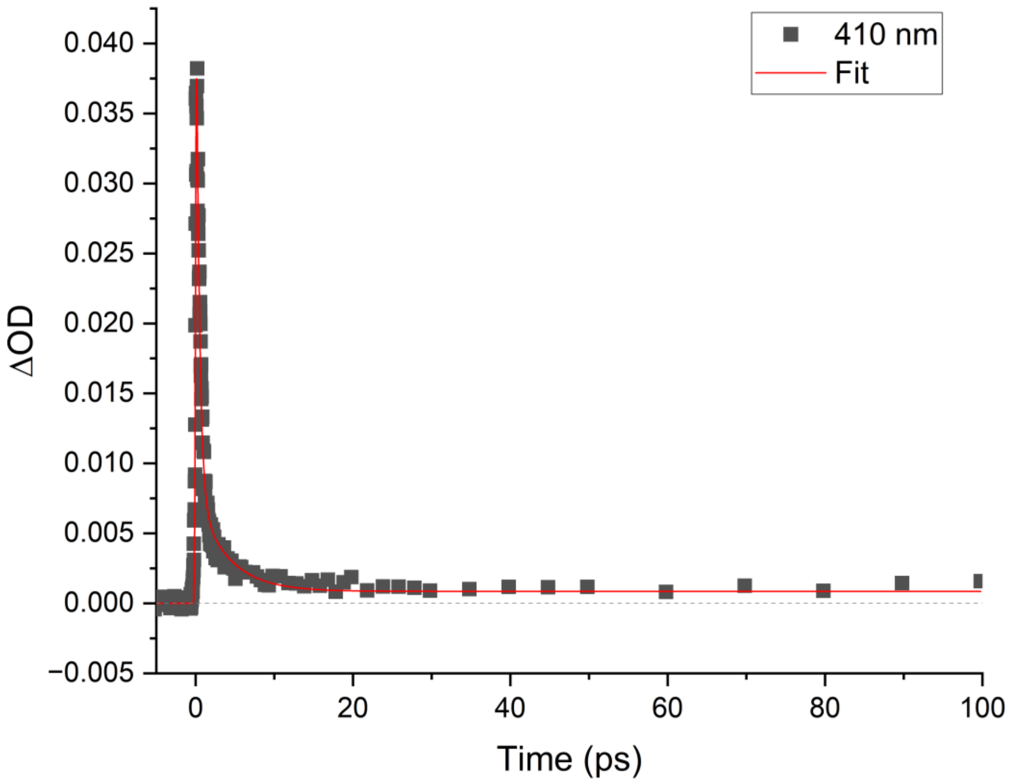

Figure S16: Trace at 410 nm recorded in acetonitrile for the non-fluorinated ***E*-PAP-H**.

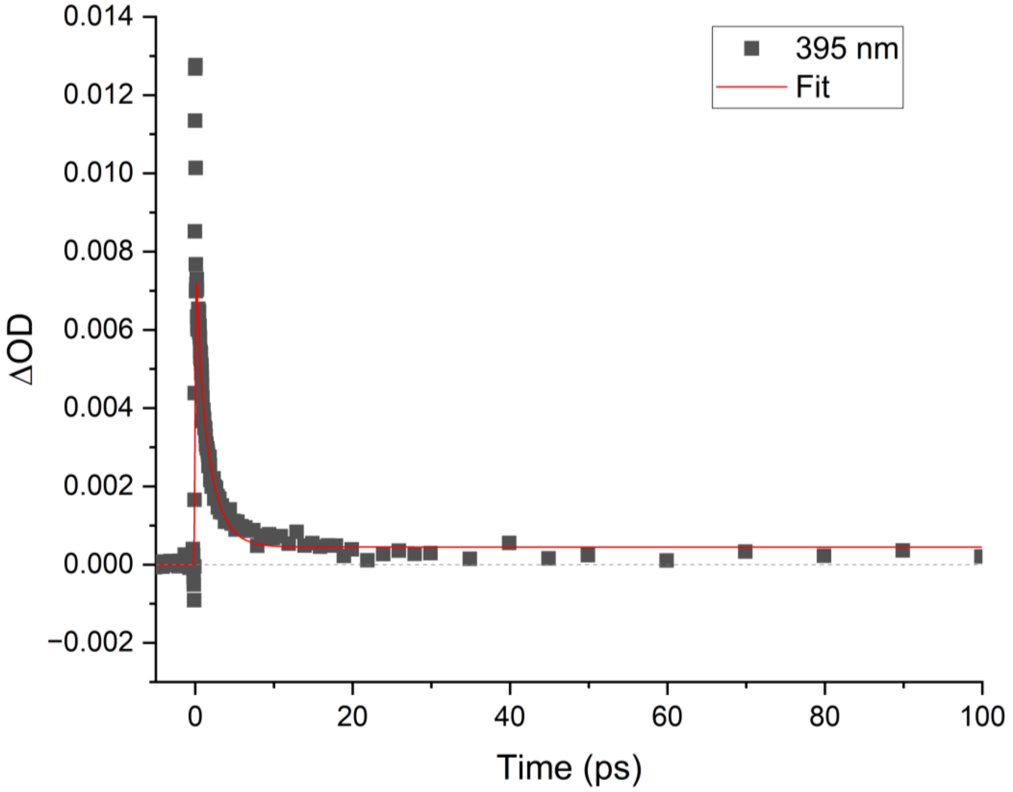

Figure S17: Trace at 395 nm recorded in acetonitrile for the fluorinated ***E*-F-PAP-H**.

3.4. Photochemical Isomerization in Steady-state UV-Vis Absorption Spectroscopy

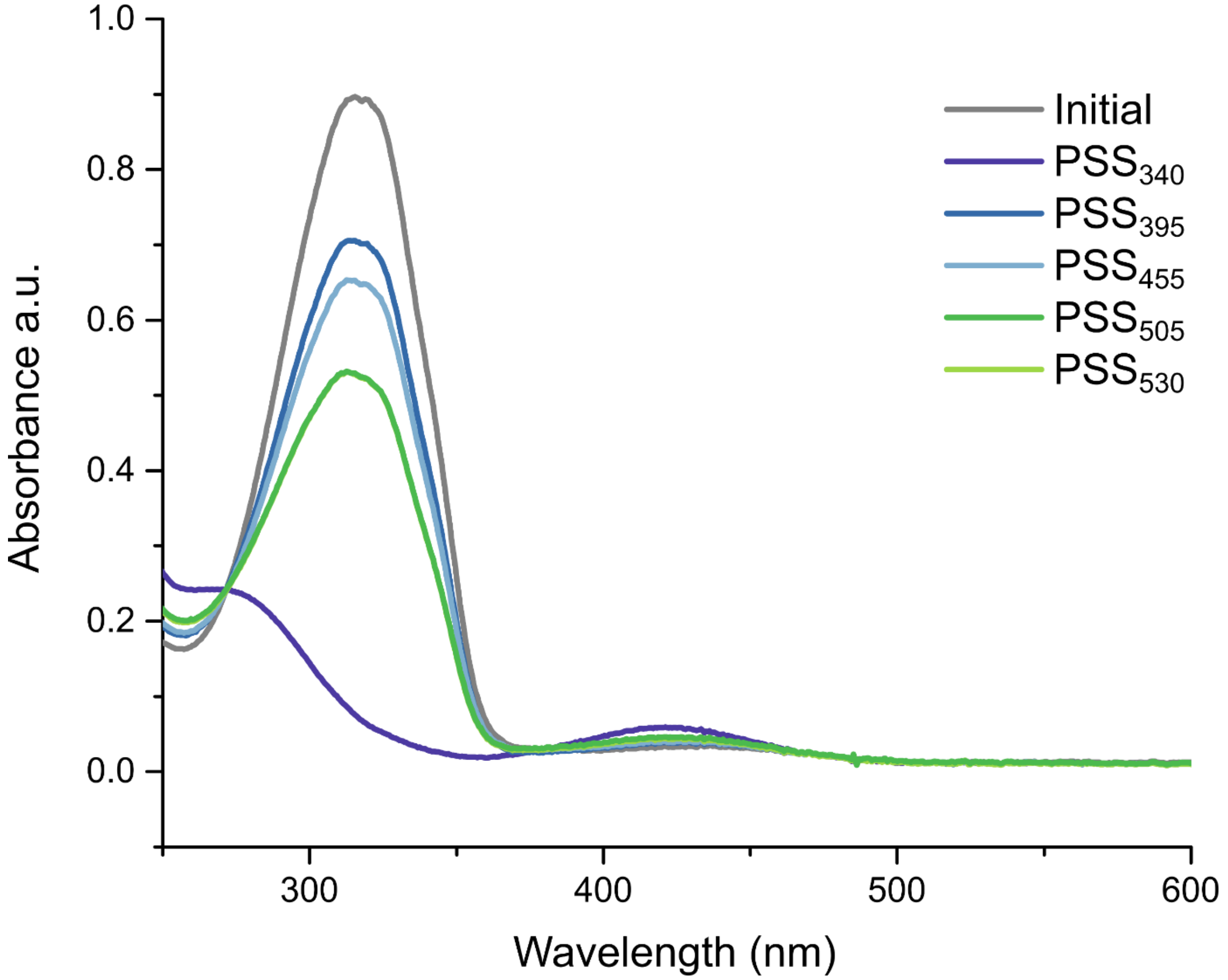

Figure S18: Plot of overlapped spectra of differently reached PSSs of **F-PAP-H** after irradiation with LEDs (340, 395, 455, 505 and 530 nm).

All the compounds have been measured in triplicate; for clarity, only one dataset will be shown in the figures below.

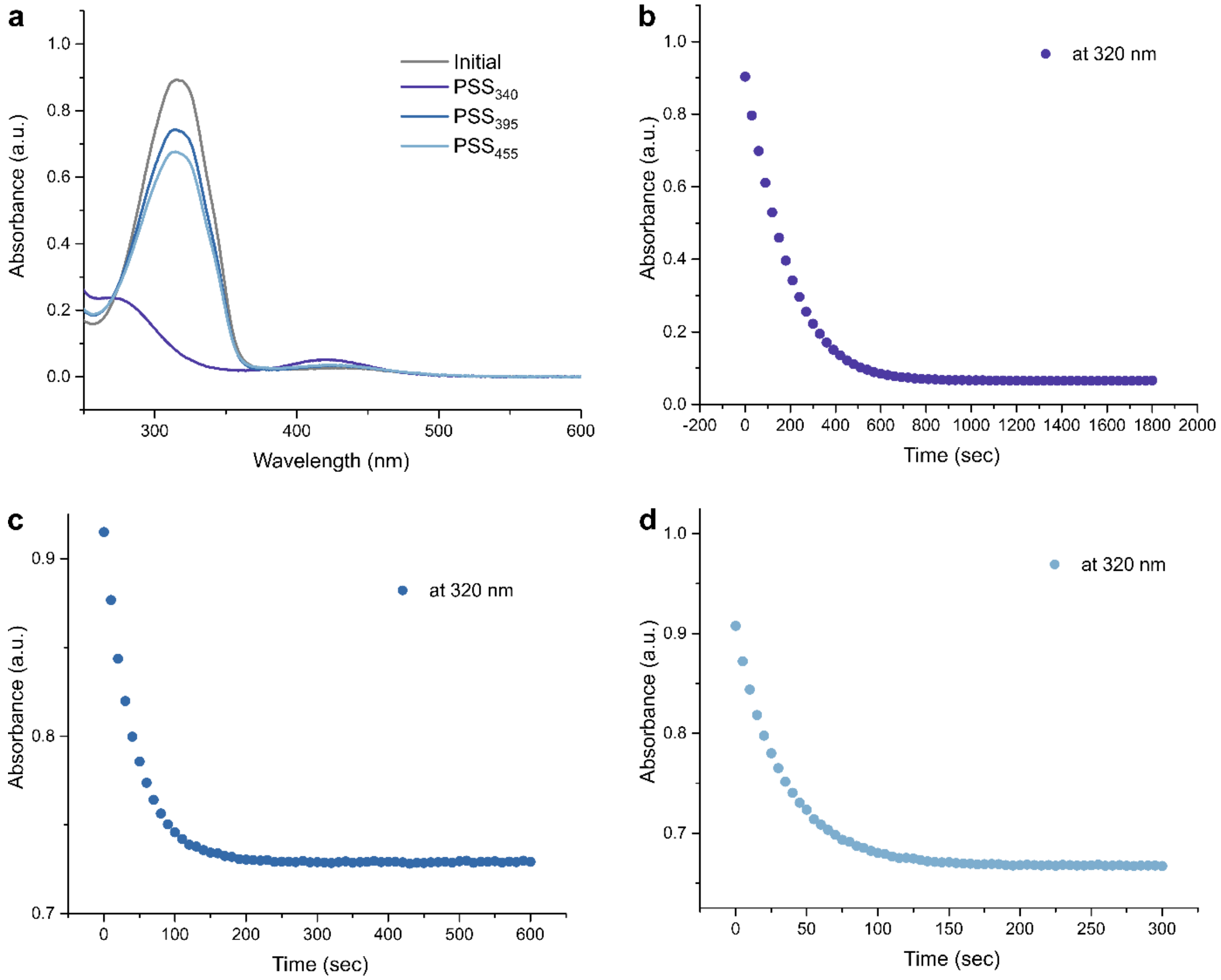

Figure S19: **a** Reached PSSs after irradiation of **F-PAP-H** in acetonitrile ($4.7x10^{-2}$ mM) at λ = 340 (purple), 395 (blue) and 455 (lightblue) nm. **b** Evolution of the wavelength at λ = 320 nm at PSS_{340}. **c** Evolution of the wavelength at λ = 320 nm at PSS_{395}. **d** Evolution of the wavelength at λ = 320 nm at PSS_{455}.

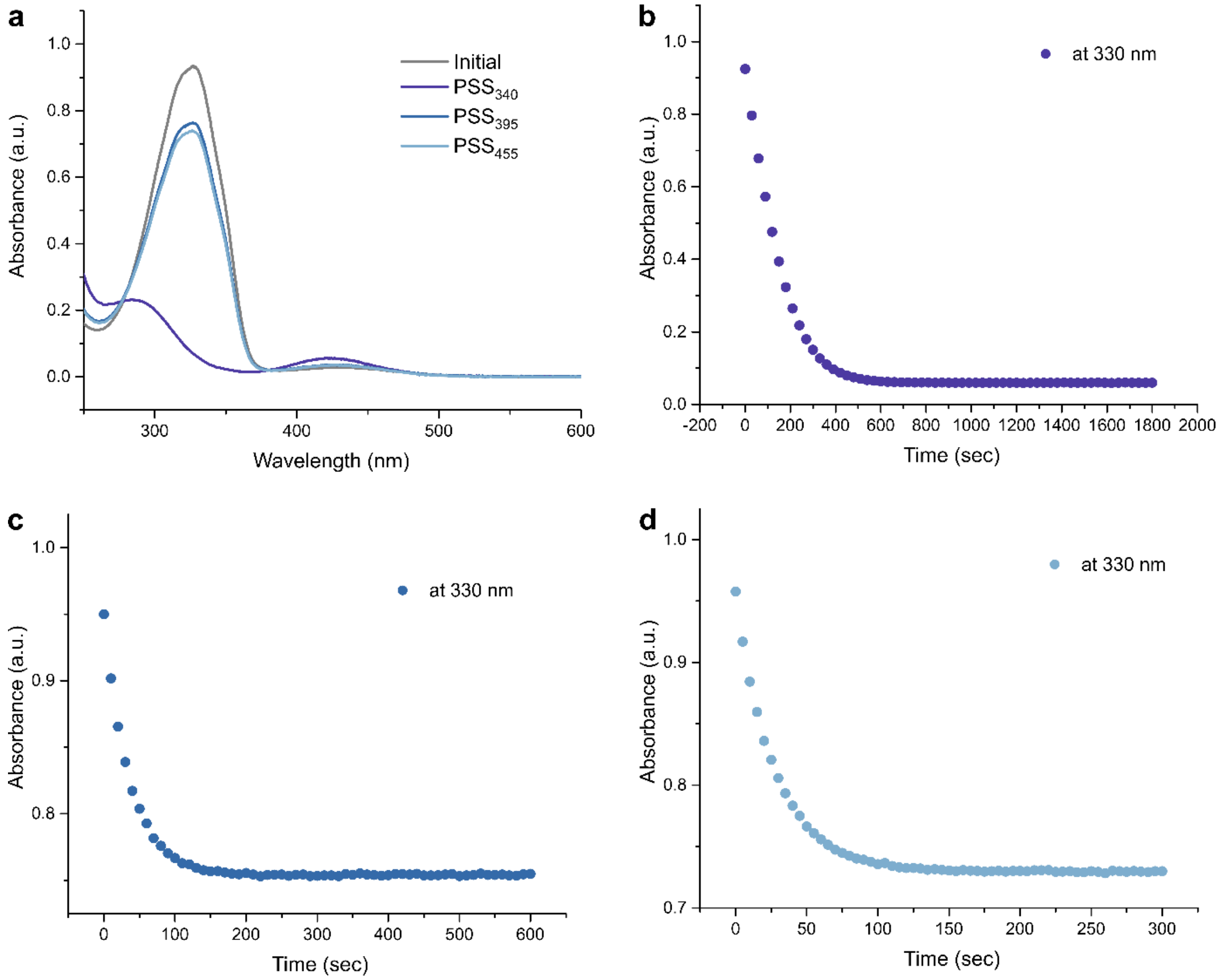

Figure S20: **a** Reached PSSs after irradiation of **F-PAP-Cl** in acetonitrile ($4.0x10^{-2}$ mM) at λ = 340 (purple), 395 (blue) and 455 (lightblue) nm. **b** Evolution of the wavelength at λ = 330 nm at PSS_{340}. **c** Evolution of the wavelength at λ = 330 nm at PSS_{395}. **d** Evolution of the wavelength at λ = 330 nm at PSS_{455}.

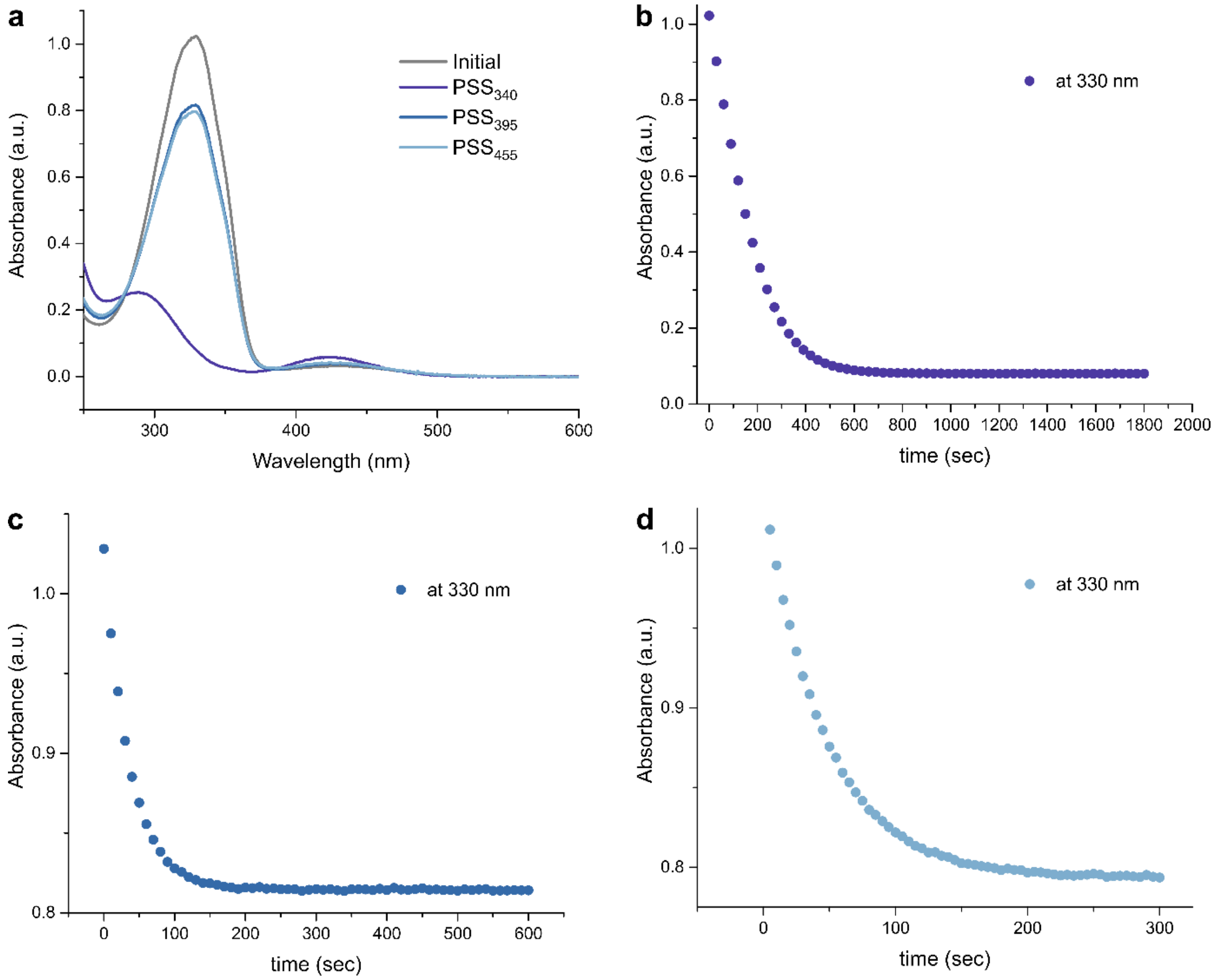

Figure S21: **a** Reached PSSs after irradiation of **F-PAP-Br** in acetonitrile ($4.2x10^{-2}$ mM) at λ = 340 (purple), 395 (blue) and 455 (lightblue) nm. **b** Evolution of the wavelength at λ = 330 nm at PSS_{340}. **c** Evolution of the wavelength at λ = 330 nm at PSS_{395}. **d** Evolution of the wavelength at λ = 330 nm at PSS_{455}.

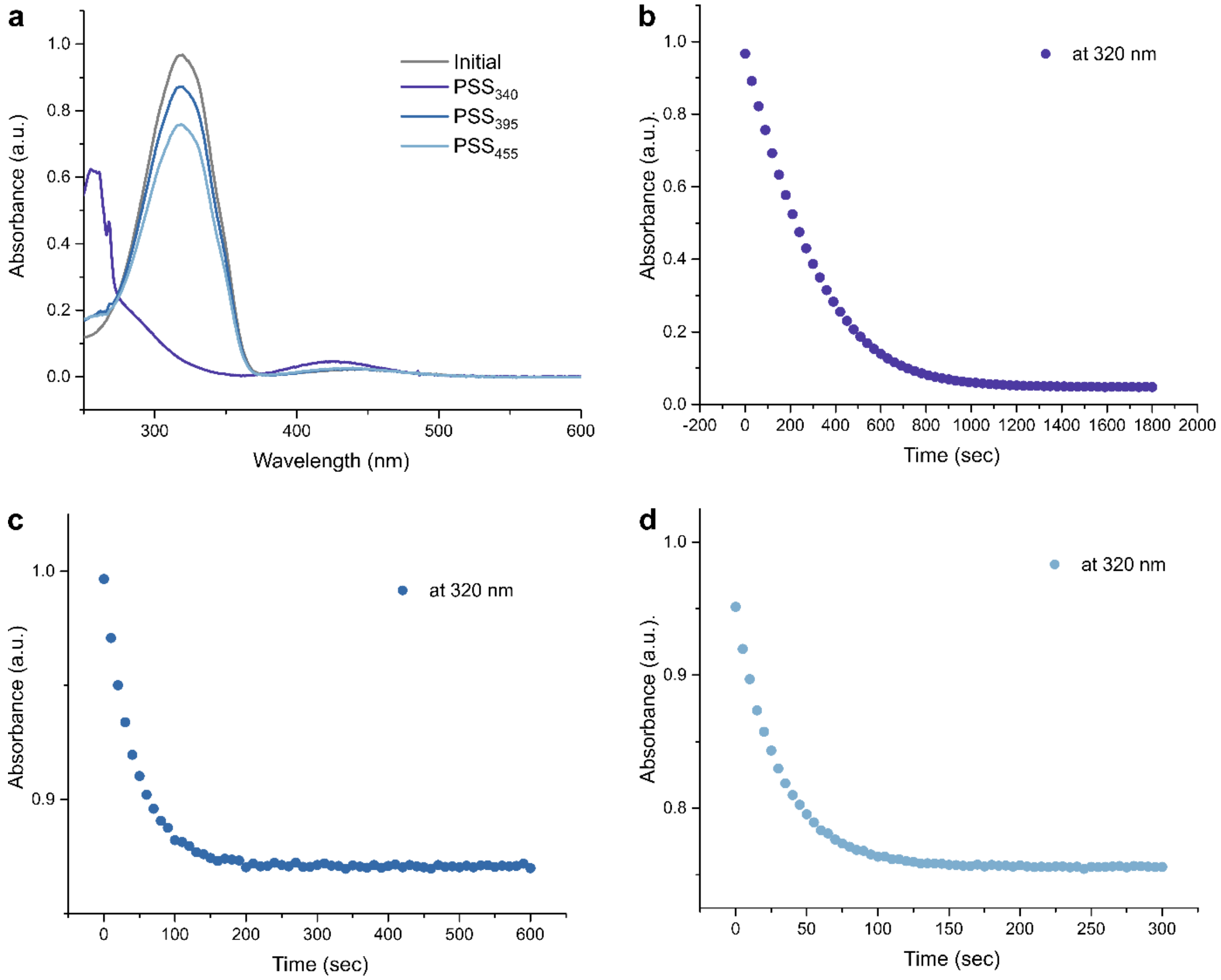

Figure S22: **a** Reached PSSs after irradiation of **F-PAP-CN** in acetonitrile ($4.0x10^{-2}$ mM) at λ = 340 (purple), 395 (blue) and 455 (lightblue) nm. **b** Evolution of the wavelength at λ = 320 nm at PSS_{340}. **c** Evolution of the wavelength at λ = 320 nm at PSS_{395}. **d** Evolution of the wavelength at λ = 320 nm at PSS_{455}.

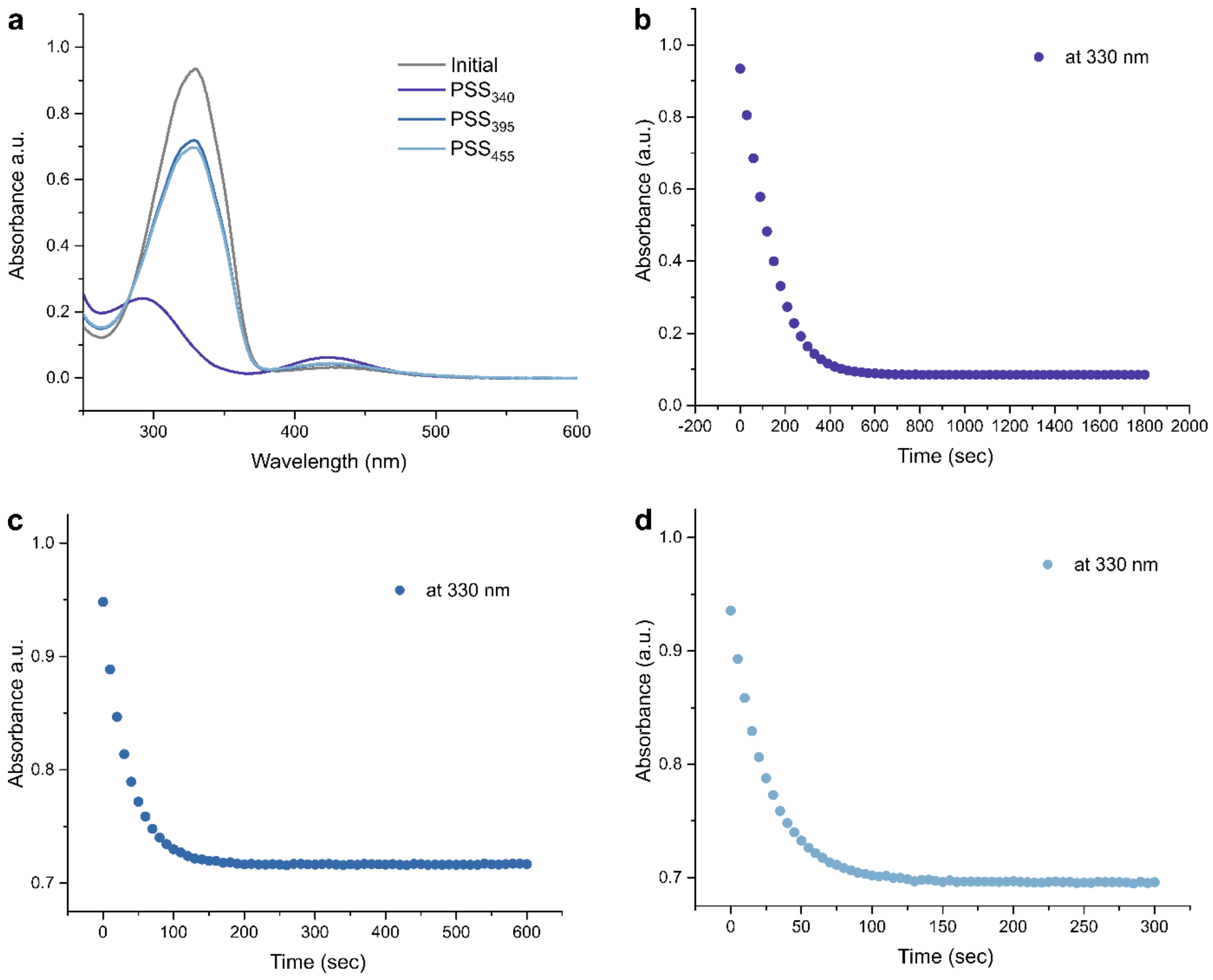

Figure S23: a Reached PSSs after irradiation of **F-PAP-Me** in acetonitrile ($4.3x10^{-2}$ mM) at λ = 340 (purple), 395 (blue) and 455 (lightblue) nm. **b** Evolution of the wavelength at λ = 330 nm at PSS_{340}. **c** Evolution of the wavelength at λ = 330 nm at PSS_{395}. **d** Evolution of the wavelength at λ = 330 nm at PSS_{455}.

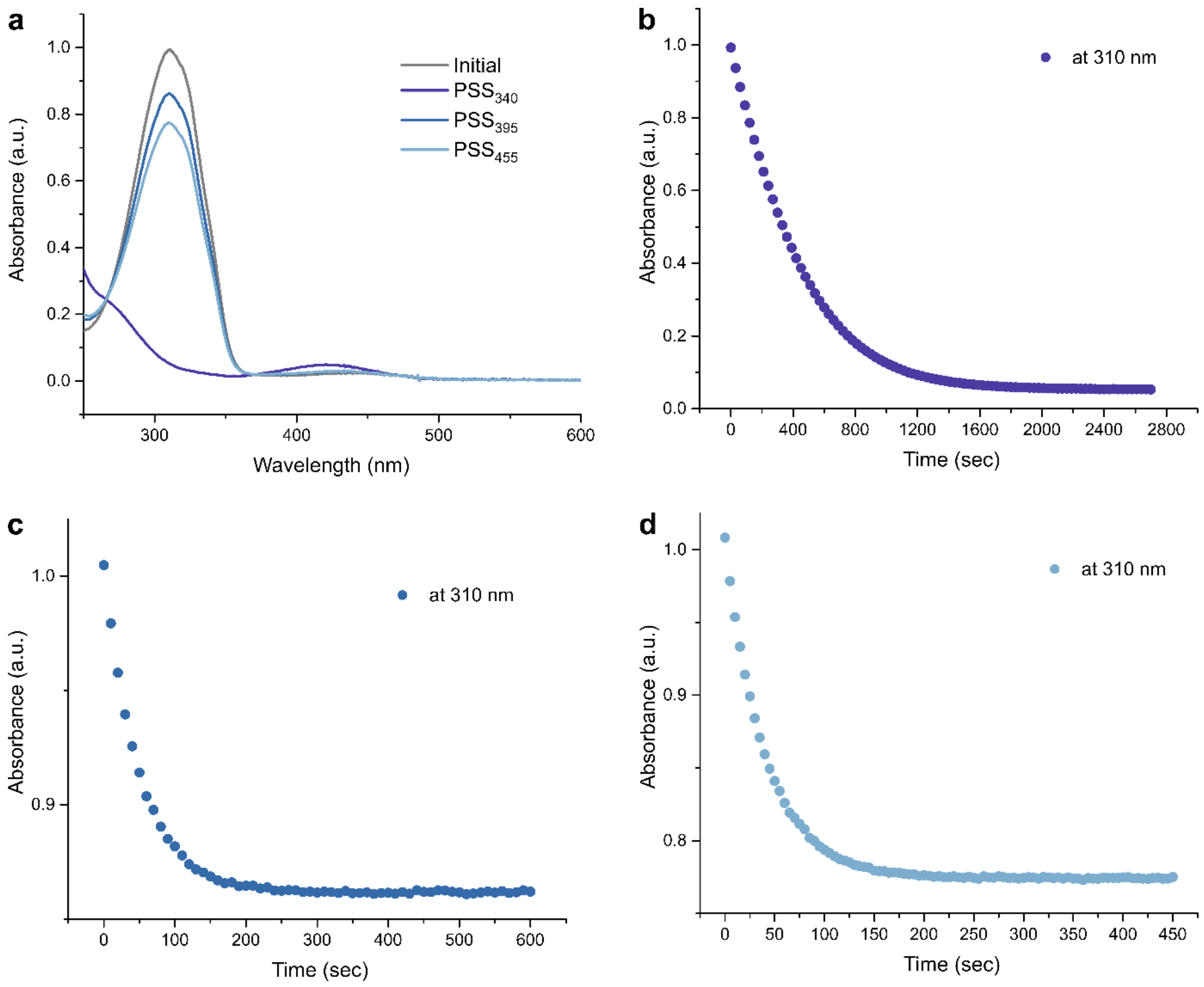

Figure S24: a Reached PSSs after irradiation of **F-PAP-CF$_3$** in acetonitrile (4.7x10^{-2} mM) at λ = 340 (purple), 395 (blue) and 455 (lightblue) nm. **b** Evolution of the wavelength at λ = 310 nm at PSS$_{340}$. **c** Evolution of the wavelength at λ = 310 nm at PSS$_{395}$. **d** Evolution of the wavelength at λ = 310 nm at PSS$_{455}$.

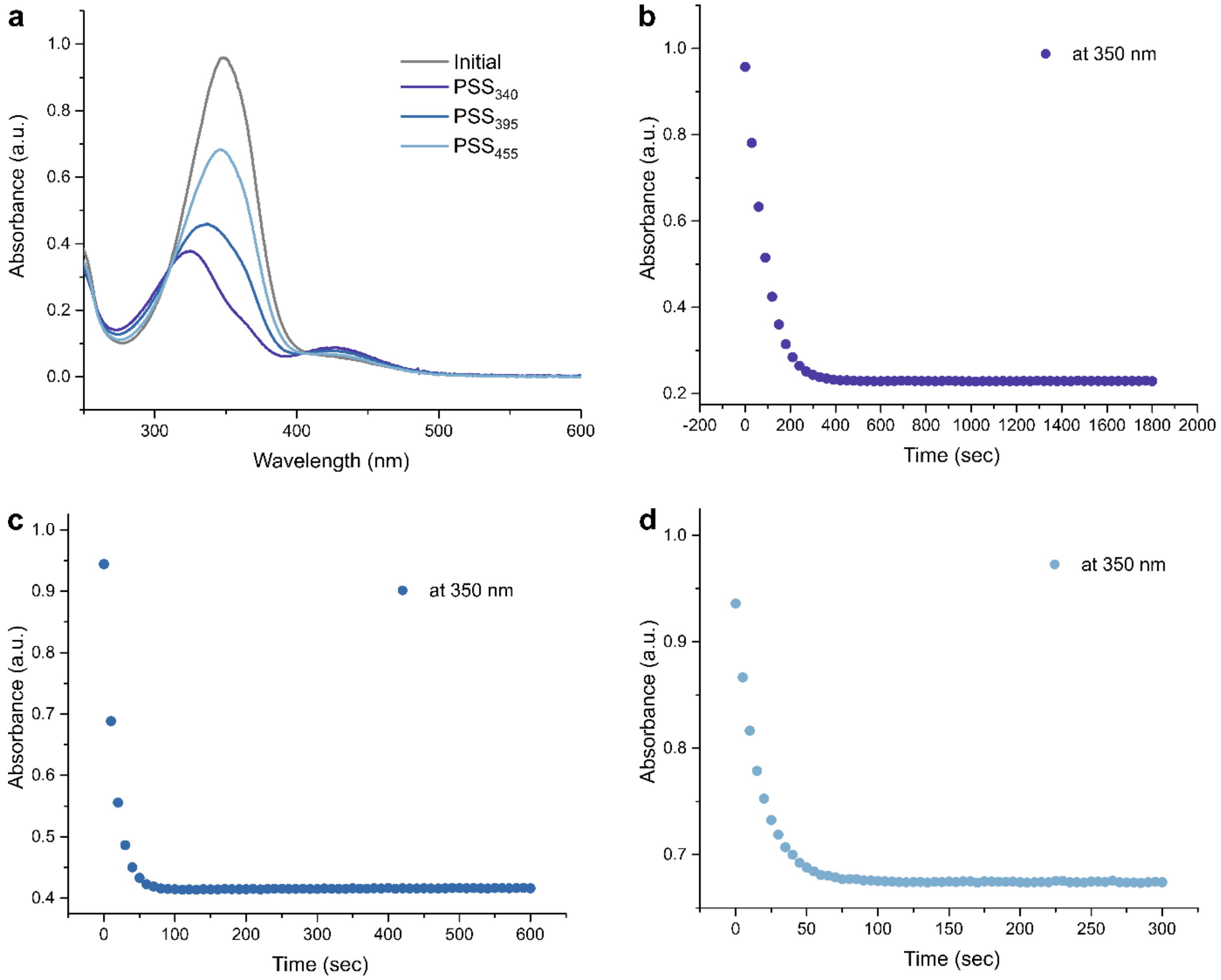

Figure S25: a Reached PSSs after irradiation of **F-PAP-OMe** in acetonitrile (3.4x10^{-2} mM) at λ = 340 (purple), 395 (blue) and 455 (lightblue) nm. **b** Evolution of the wavelength at λ = 350 nm at PSS_{340}. **c** Evolution of the wavelength at λ = 350 nm at PSS_{395}. **d** Evolution of the wavelength at λ = 350 nm at PSS_{455}.

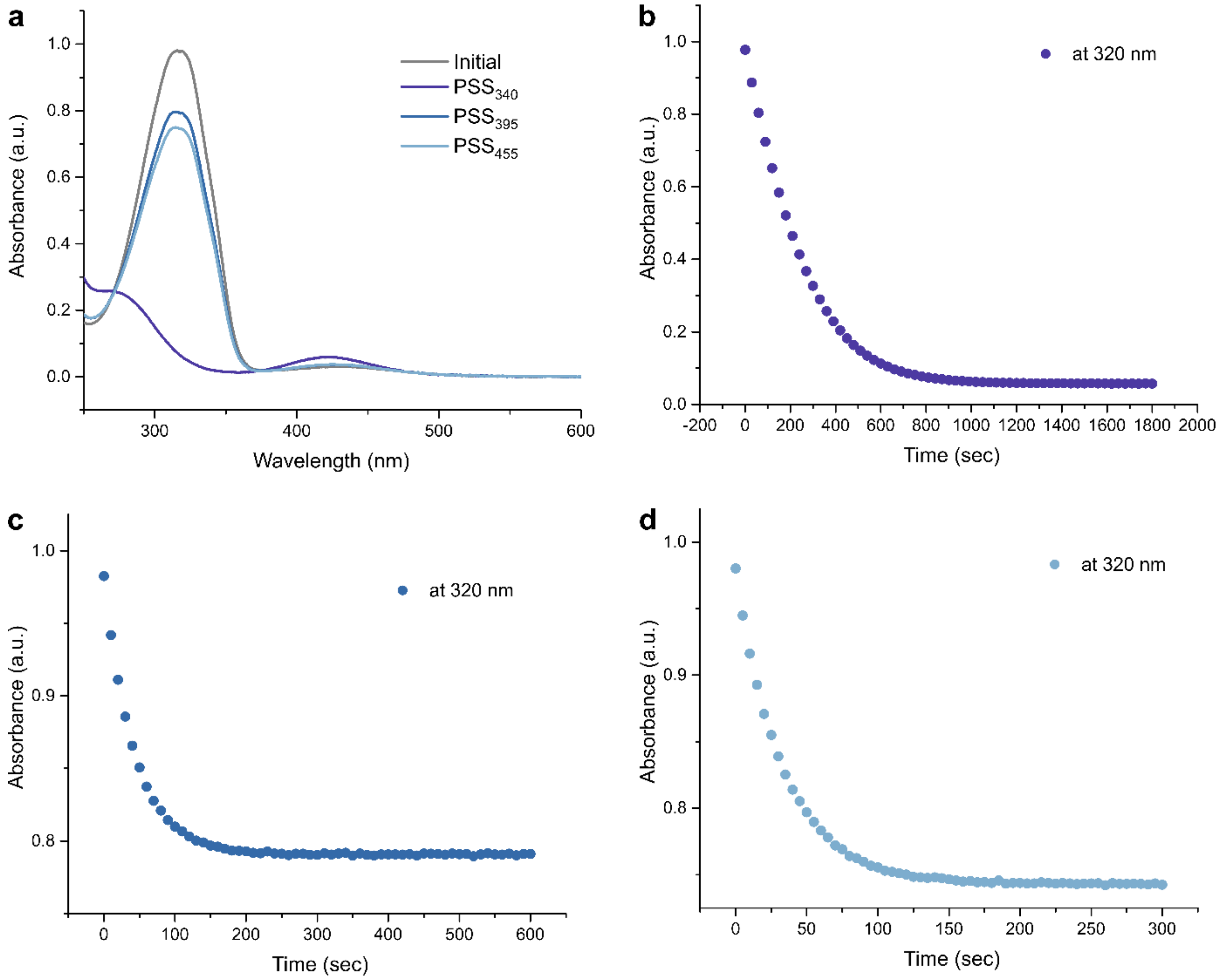

Figure S26: **a** Reached PSSs after irradiation of **F-PAP-OCF$_3$** in acetonitrile (4.6x10^{-2} mM) at λ = 340 (purple), 395 (blue) and 455 (lightblue) nm. **b** Evolution of the wavelength at λ = 320 nm at PSS$_{340}$. **c** Evolution of the wavelength at λ = 320 nm at PSS$_{395}$. **d** Evolution of the wavelength at λ = 320 nm at PSS$_{455}$.

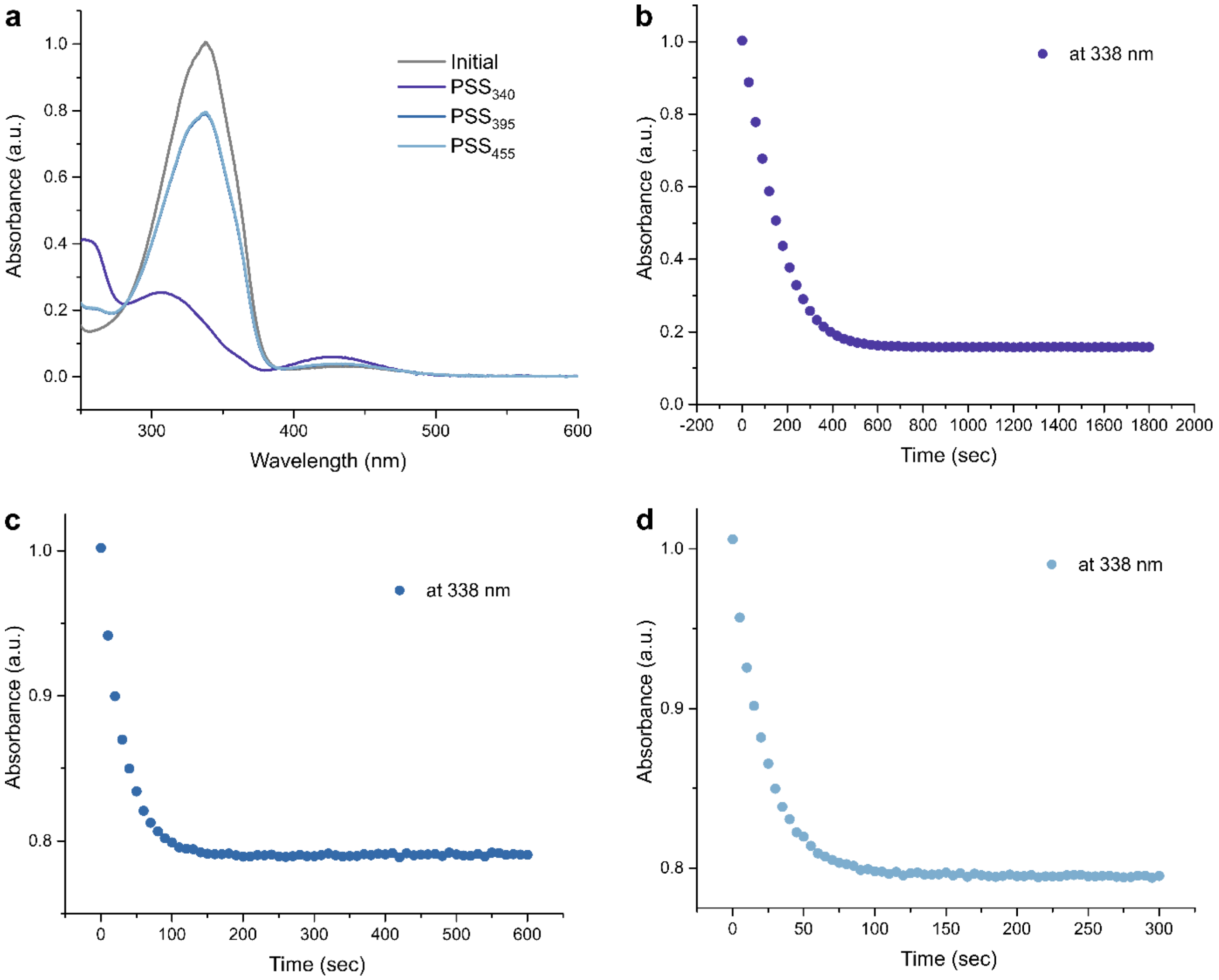

Figure S27: **a** Reached PSSs after irradiation of **F-PAP-CCH** in acetonitrile ($3.8x10^{-2}$ mM) at λ = 340 (purple), 395 (blue) and 455 (lightblue) nm. **b** Evolution of the wavelength at λ = 338 nm at PSS_{340}. **c** Evolution of the wavelength at λ = 338 nm at PSS_{395}. **d** Evolution of the wavelength at λ = 338 nm at PSS_{455}.

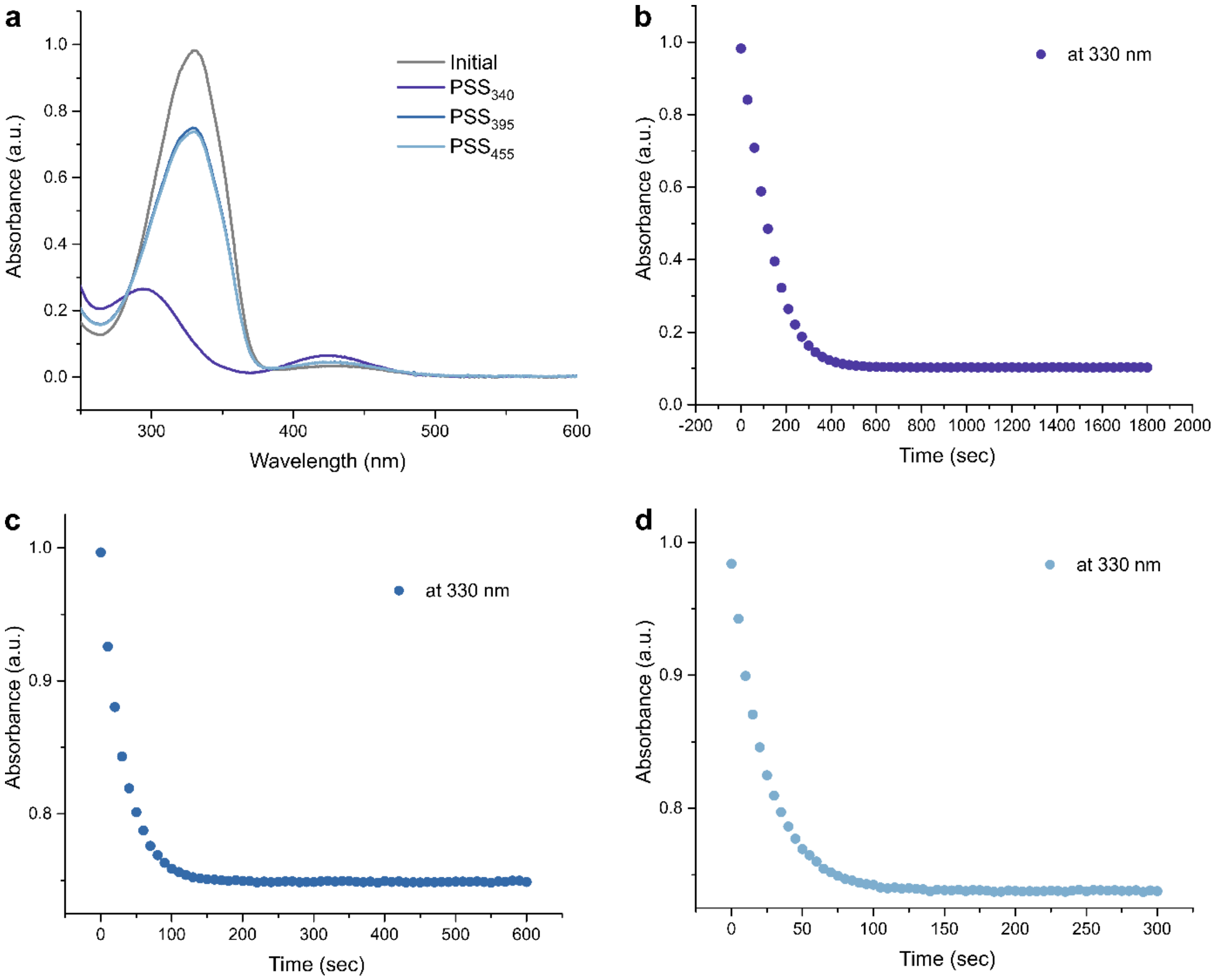

Figure S28: **a** Reached PSSs after irradiation of **F-PAP-*n*Bu** in acetonitrile (4.2x10^{-2} mM) at λ = 340 (purple), 395 (blue) and 455 (lightblue) nm. **b** Evolution of the wavelength at λ = 330 nm at PSS_{340}. **c** Evolution of the wavelength at λ = 330 nm at PSS_{395}. **d** Evolution of the wavelength at λ = 330 nm at PSS_{455}.

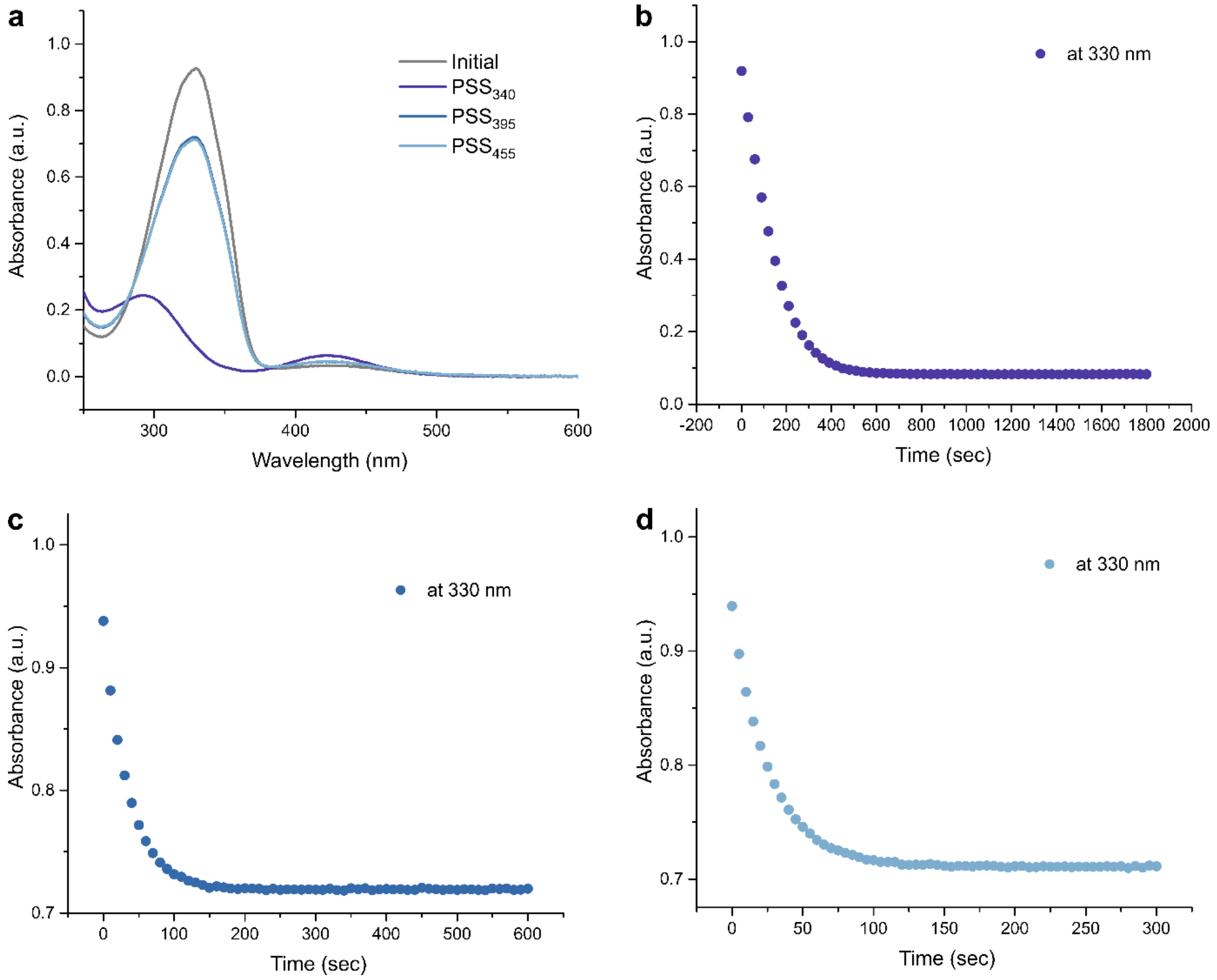

Figure S29: a Reached PSSs after irradiation of **F-PAP-C_3H_6COOH** in acetonitrile ($4.5x10^{2}$ mM) at λ = 340 (purple), 395 (blue) and 455 (lightblue) nm. **b** Evolution of the wavelength at λ = 330 nm at PSS_{340}. **c** Evolution of the wavelength at λ = 330 nm at PSS_{395}. **d** Evolution of the wavelength at λ = 330 nm at PSS_{455}.

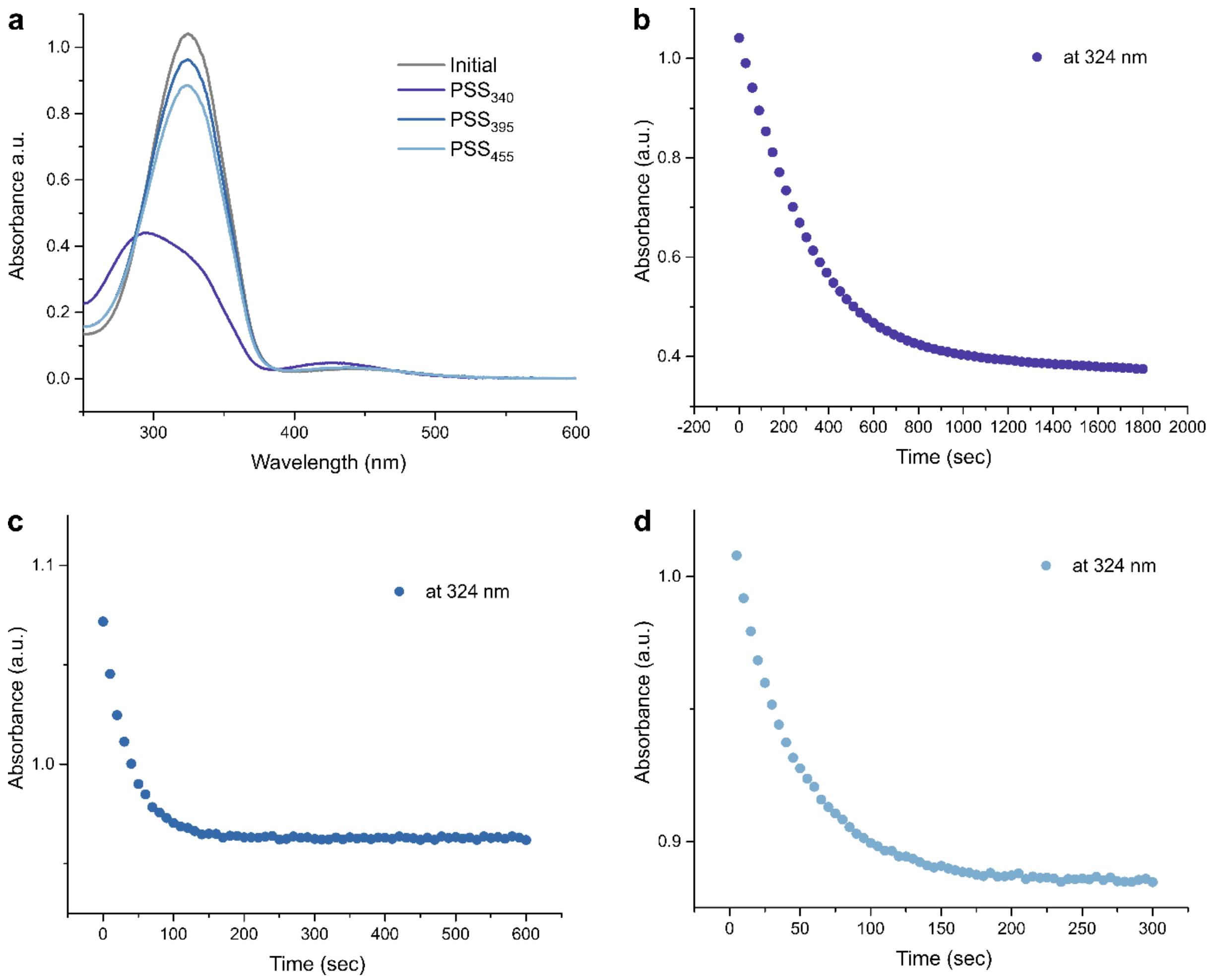

Figure S30: a Reached PSSs after irradiation of **F-PAP-NO$_2$** in acetonitrile (4.1x10^{-2} mM) at λ = 340 (purple), 395 (blue) and 455 (lightblue) nm. **b** Evolution of the wavelength at λ = 324 nm at PSS$_{340}$. **c** Evolution of the wavelength at λ = 324 nm at PSS$_{395}$. **d** Evolution of the wavelength at λ = 324 nm at PSS$_{455}$.

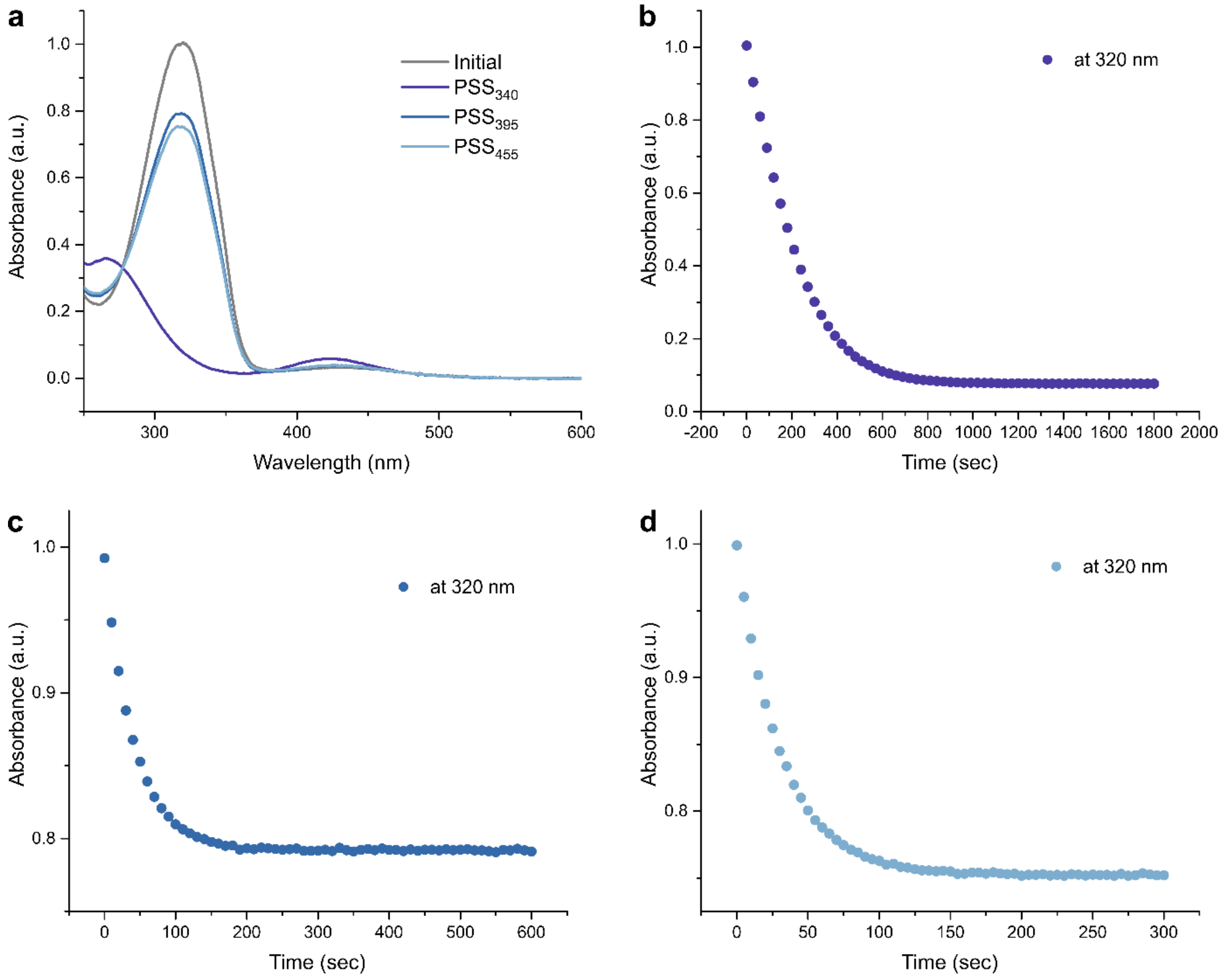

Figure S31: a Reached PSSs after irradiation of **F-(*N*Ph)PAP** in acetonitrile (4.6x10^{-2} mM) at λ = 340 (purple), 395 (blue) and 455 (lightblue) nm. **b** Evolution of the wavelength at λ = 320 nm at PSS_{340}. **c** Evolution of the wavelength at λ = 320 nm at PSS_{395}. **d** Evolution of the wavelength at λ = 320 nm at PSS_{455}.

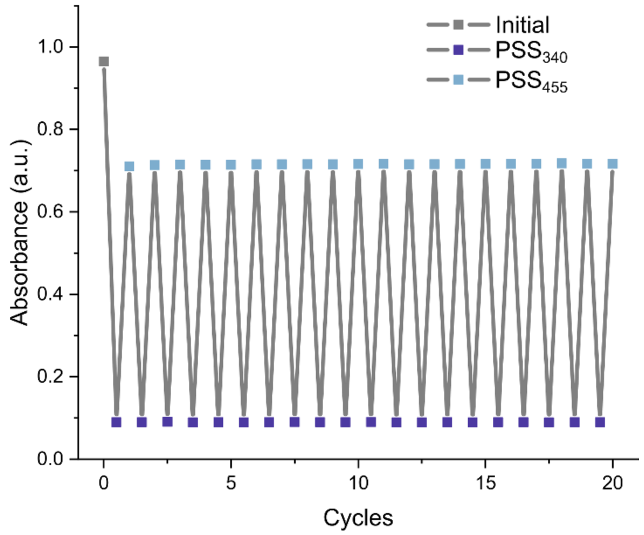

Figure S32: Plot of photoisomerisation cycles where we started from an initial absorbance (grey square) and where 340 nm light is used for reaching the PSS after *E*-*Z* isomerization (purple square) followed by 455 nm light to reach an alternate PSS after *Z*-*E* photoisomerization (light blue square). After 20 cycles, no photofatigue is observed.

3.5. Quantum Yield Determination

The spectrum for the metastable state (*Z*) of **F-PAPs** were retrieved by scaled subtraction of the initial spectra and spectra at the PSS at 340 nm. The *E*/*Z* distribution at the PSS was determined from *ex situ* NMR irradiation at 340 nm, see section 3.6. The power was integrated over the emission spectrum of the LED.

Quantum Yields at 340 nm

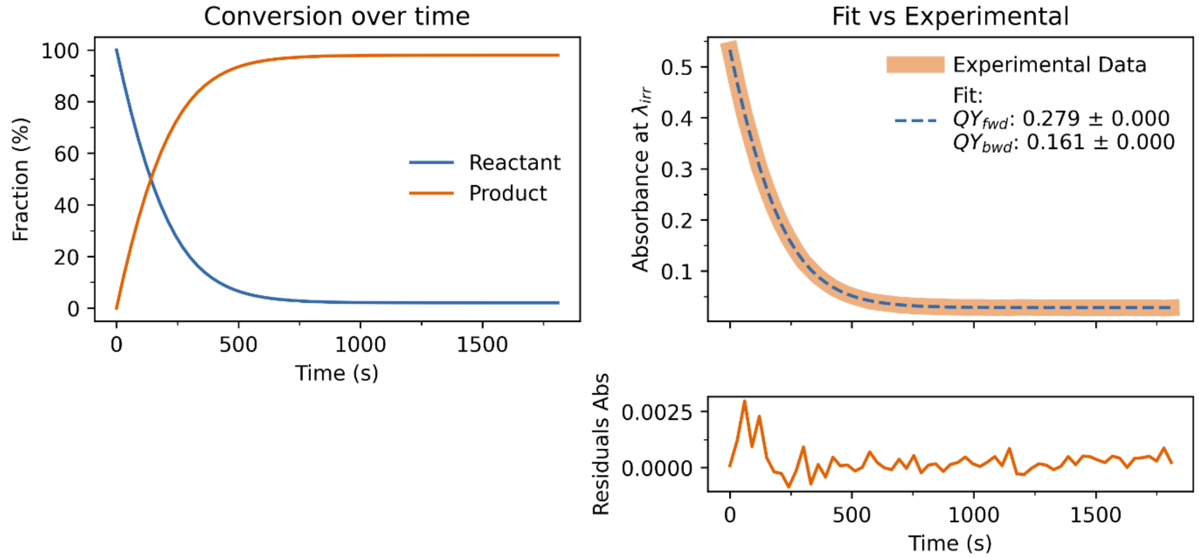

Figure S33: Relative concentration over time of *E*- and *Z*-isomers, experimental absorbance at the irradiation wavelength and fit of the integrated absorbances over the emission spectrum of the LED, and residuals of the fit of the irradiation of **F-PAP-H** in acetonitrile with λ = 340 nm.

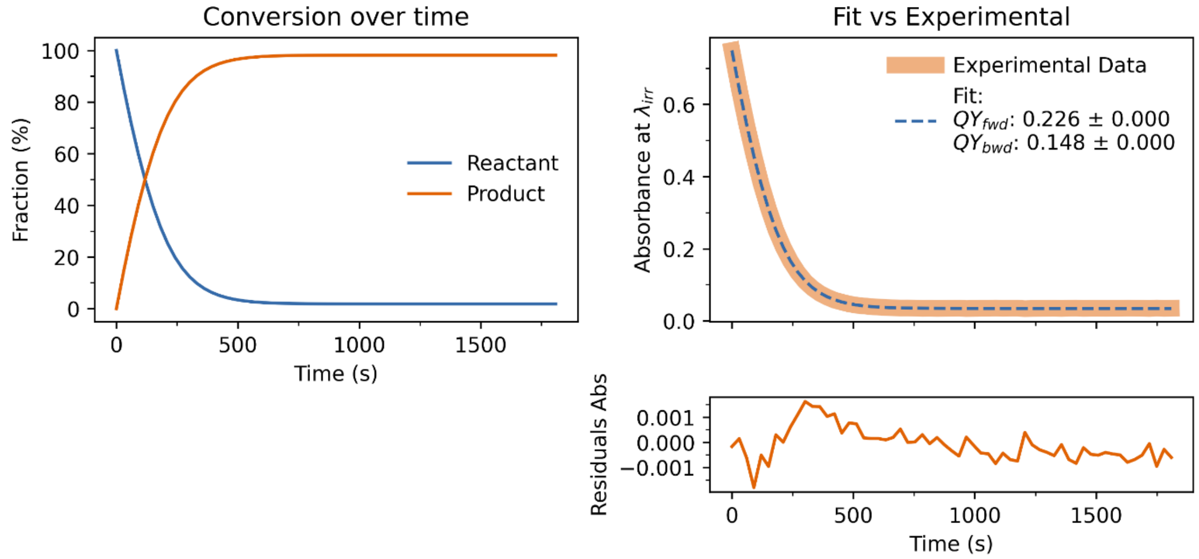

Figure S34: Relative concentration over time of *E*- and *Z*-isomers, experimental absorbance at the irradiation wavelength and fit of the integrated absorbances over the emission spectrum of the LED, and residuals of the fit of the irradiation of **F-PAP-Cl** in acetonitrile with λ = 340 nm.

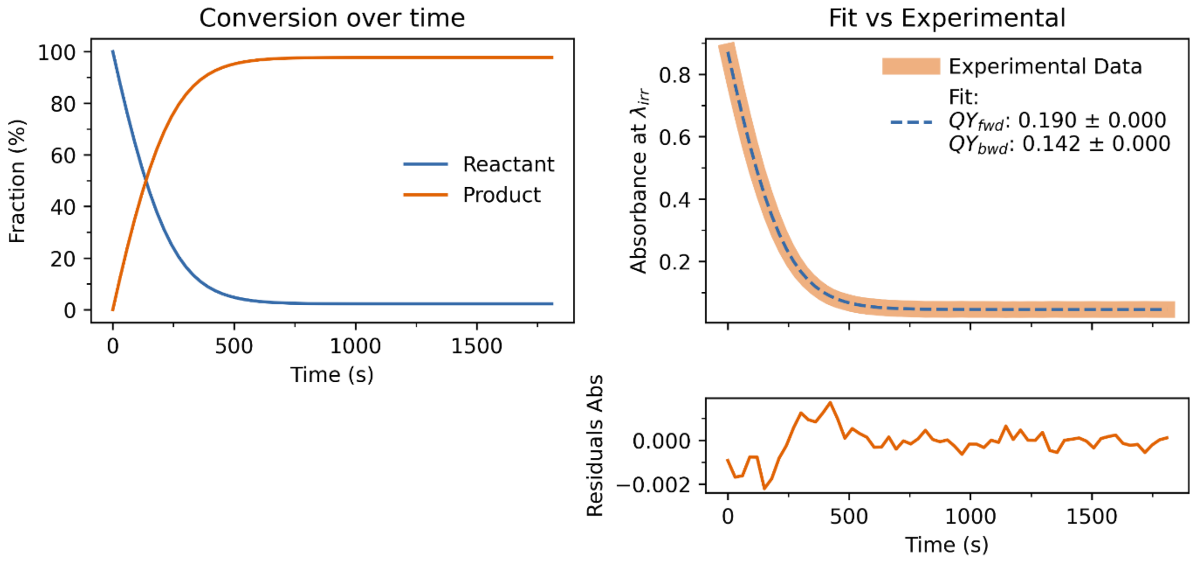

Figure S35: Relative concentration over time of *E*- and *Z*-isomers, experimental absorbance at the irradiation wavelength and fit of the integrated absorbances over the emission spectrum of the LED, and residuals of the fit of the irradiation of **F-PAP-Br** in acetonitrile with λ = 340 nm.

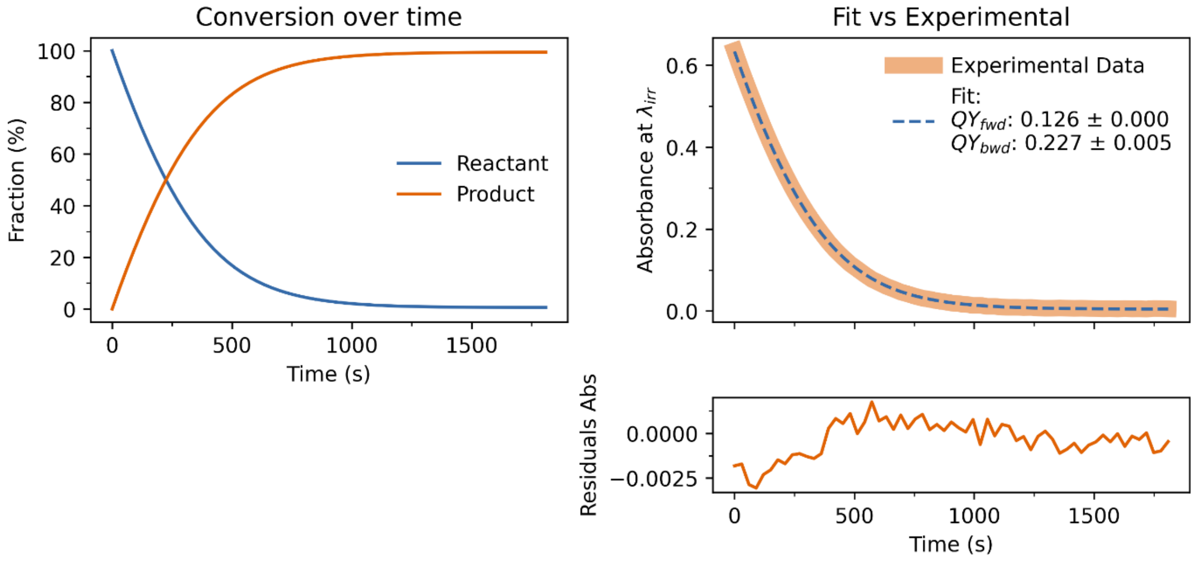

Figure S36: Relative concentration over time of *E*- and *Z*-isomers, experimental absorbance at the irradiation wavelength and fit of the integrated absorbances over the emission spectrum of the LED, and residuals of the fit of the irradiation of **F-PAP-CN** in acetonitrile with λ = 340 nm.

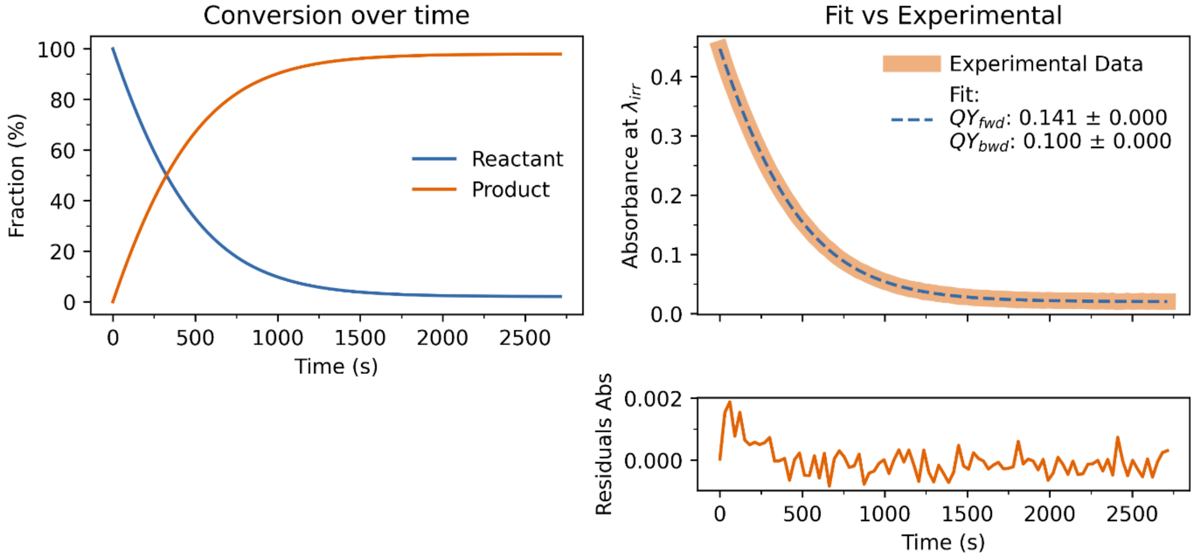

Figure S37: Relative concentration over time of *E*- and *Z*-isomers, experimental absorbance at the irradiation wavelength and fit of the integrated absorbances over the emission spectrum of the LED, and residuals of the fit of the irradiation of **F-PAP-Me** in acetonitrile with λ = 340 nm.

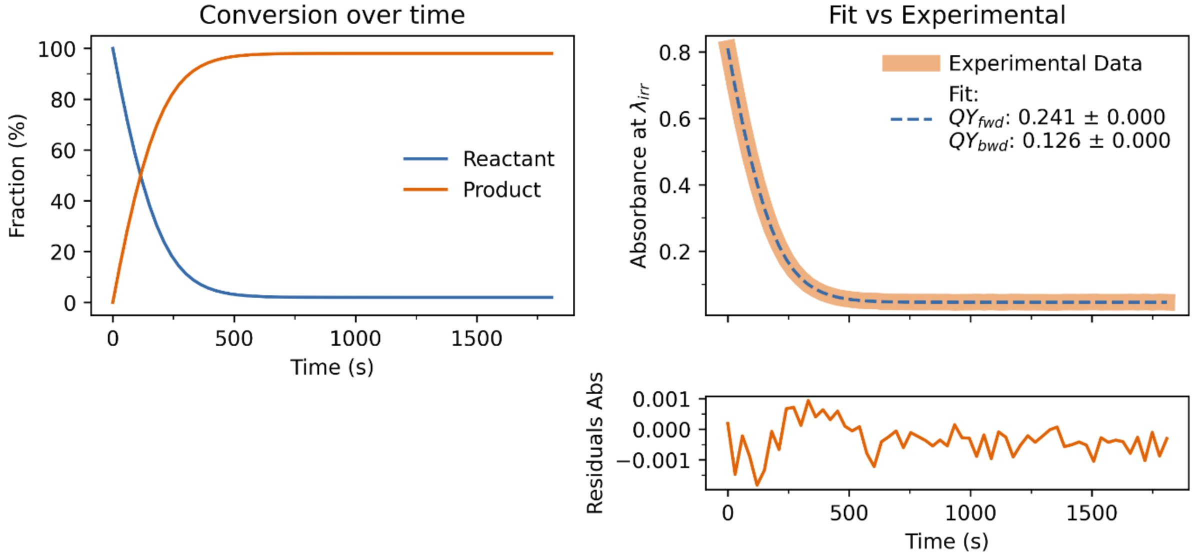

Figure S38: Relative concentration over time of *E*- and *Z*-isomers, experimental absorbance at the irradiation wavelength and fit of the integrated absorbances over the emission spectrum of the LED, and residuals of the fit of the irradiation of **F-PAP-CF_3** in acetonitrile with λ = 340 nm.

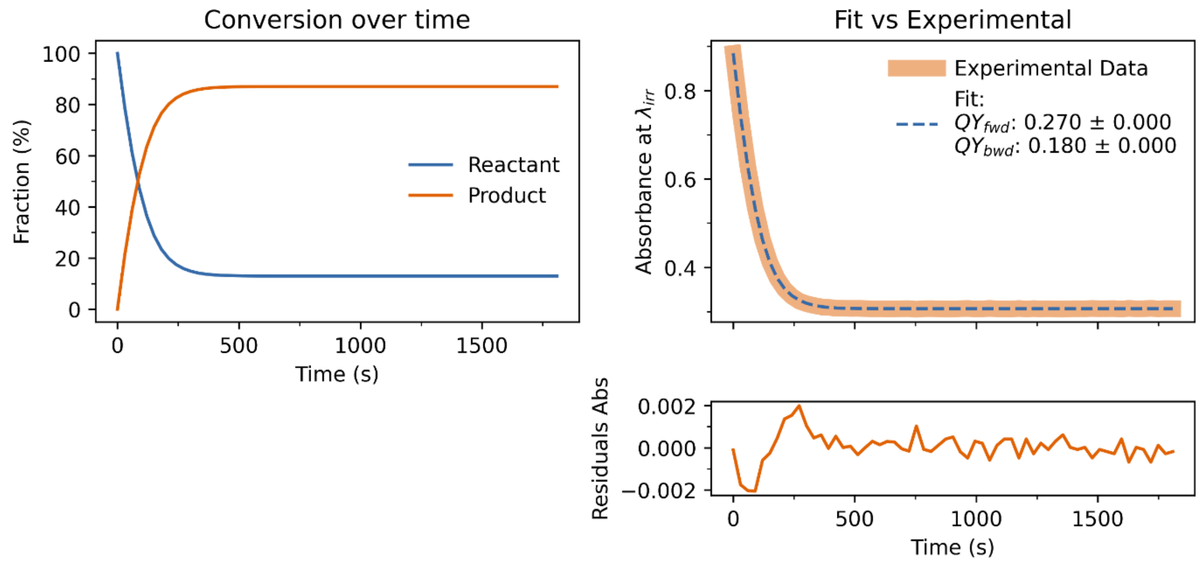

Figure S39: Relative concentration over time of *E*- and *Z*-isomers, experimental absorbance at the irradiation wavelength and fit of the integrated absorbances over the emission spectrum of the LED, and residuals of the fit of the irradiation of **F-PAP-OMe** in acetonitrile with λ = 340 nm.

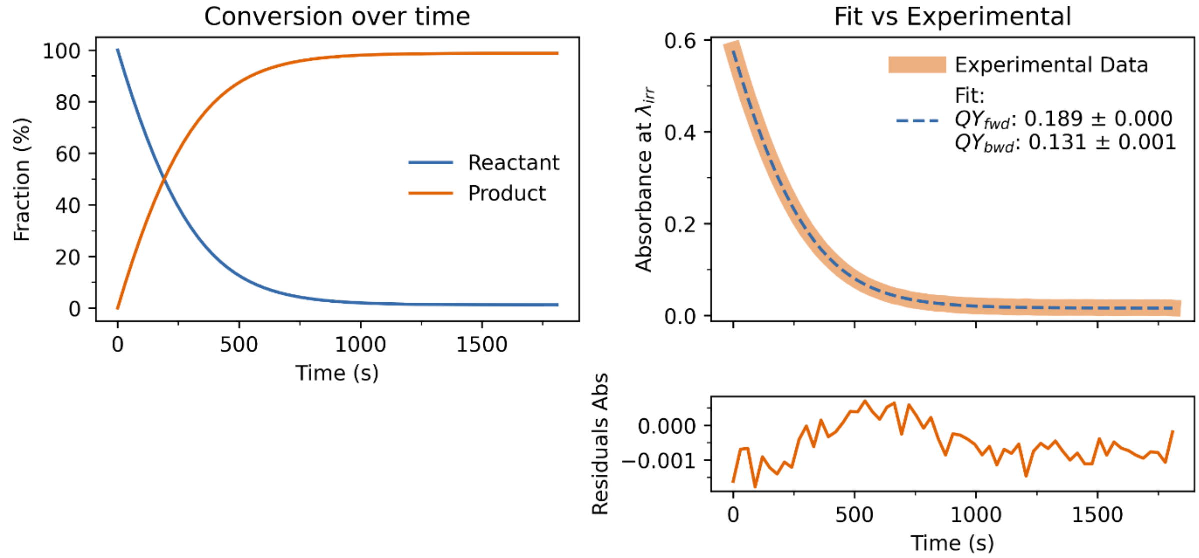

Figure S40: Relative concentration over time of *E*- and *Z*-isomers, experimental absorbance at the irradiation wavelength and fit of the integrated absorbances over the emission spectrum of the LED, and residuals of the fit of the irradiation of **F-PAP-OCF$_3$** in acetonitrile with λ = 340 nm.

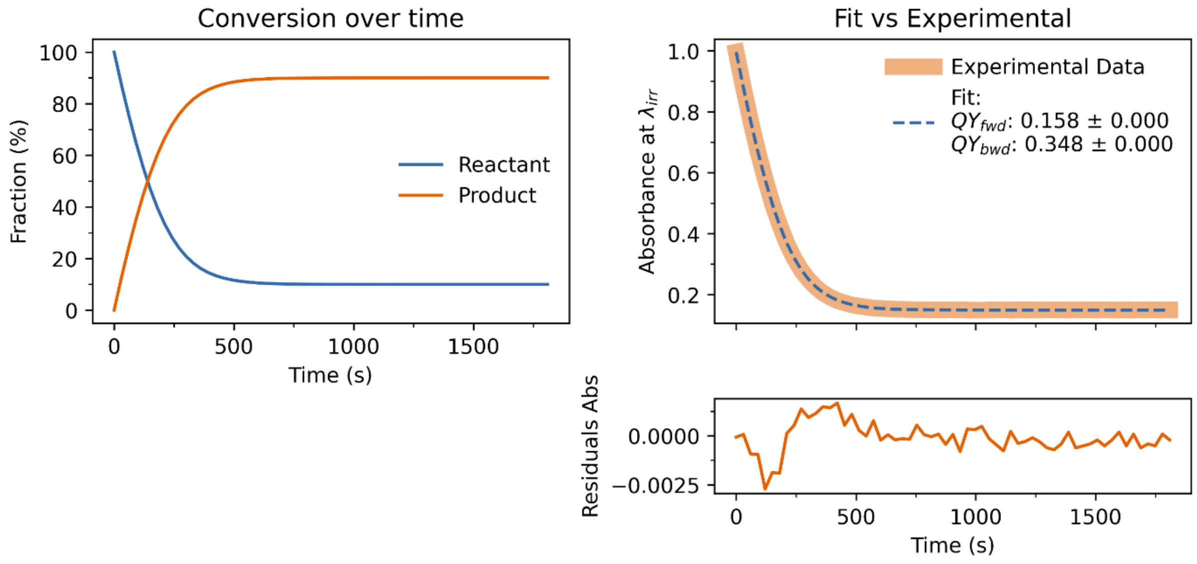

Figure S41: Relative concentration over time of *E*- and *Z*-isomers, experimental absorbance at the irradiation wavelength and fit of the integrated absorbances over the emission spectrum of the LED, and residuals of the fit of the irradiation of **F-PAP-CCH** in acetonitrile with λ = 340 nm.

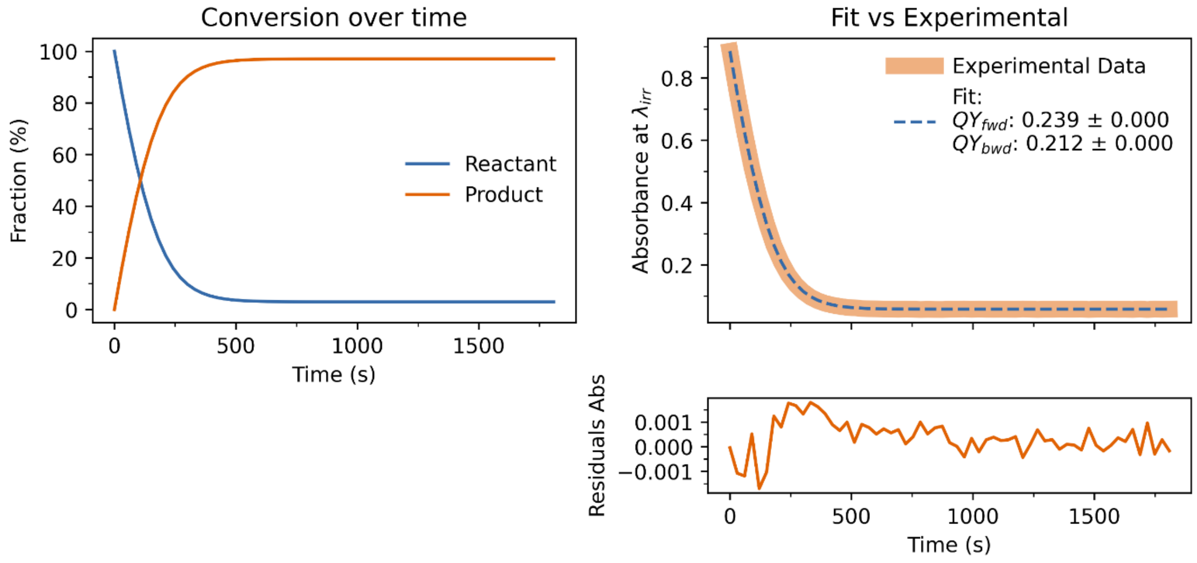

Figure S42: Relative concentration over time of *E*- and *Z*-isomers, experimental absorbance at the irradiation wavelength and fit of the integrated absorbances over the emission spectrum of the LED, and residuals of the fit of the irradiation of **F-PAP-*n*Bu** in acetonitrile with λ = 340 nm.

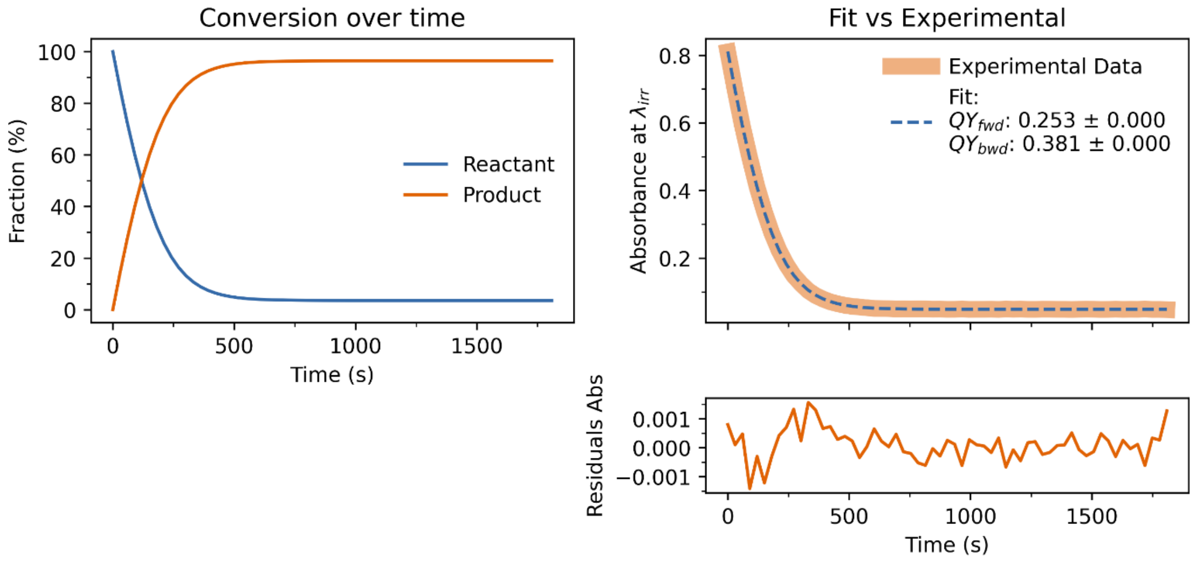

Figure S43: Relative concentration over time of *E*- and *Z*-isomers, experimental absorbance at the irradiation wavelength and fit of the integrated absorbances over the emission spectrum of the LED, and residuals of the fit of the irradiation of **F-PAP-C_3H_6COOH** in acetonitrile with λ = 340 nm.

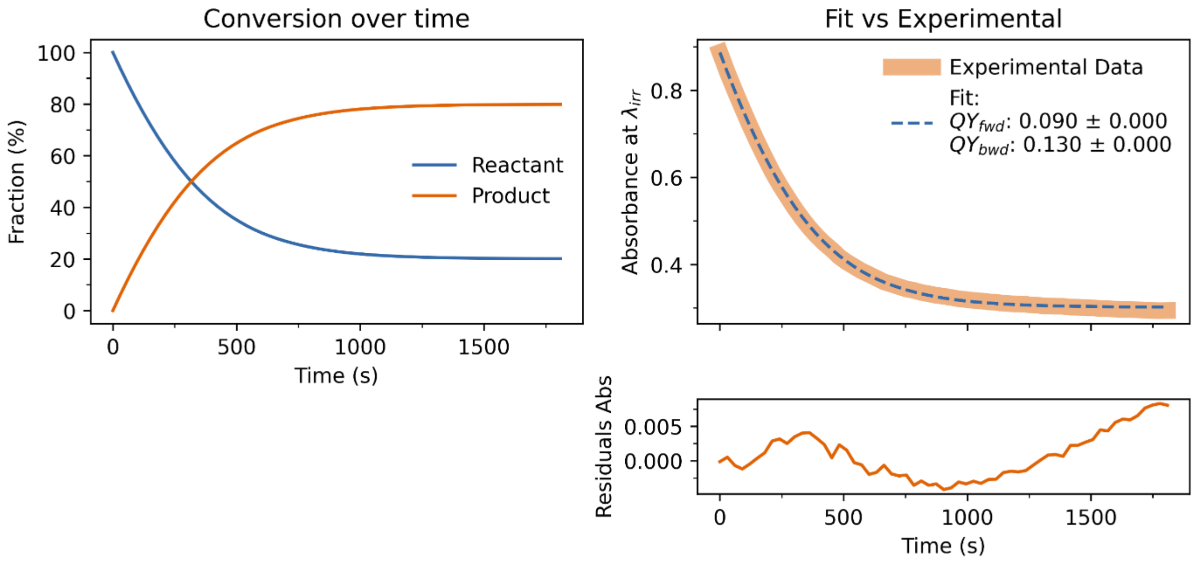

Figure S44: Relative concentration over time of *E*- and *Z*-isomers, experimental absorbance at the irradiation wavelength and fit of the integrated absorbances over the emission spectrum of the LED, and residuals of the fit of the irradiation of **F-PAP-NO_2** in acetonitrile with λ = 340 nm.

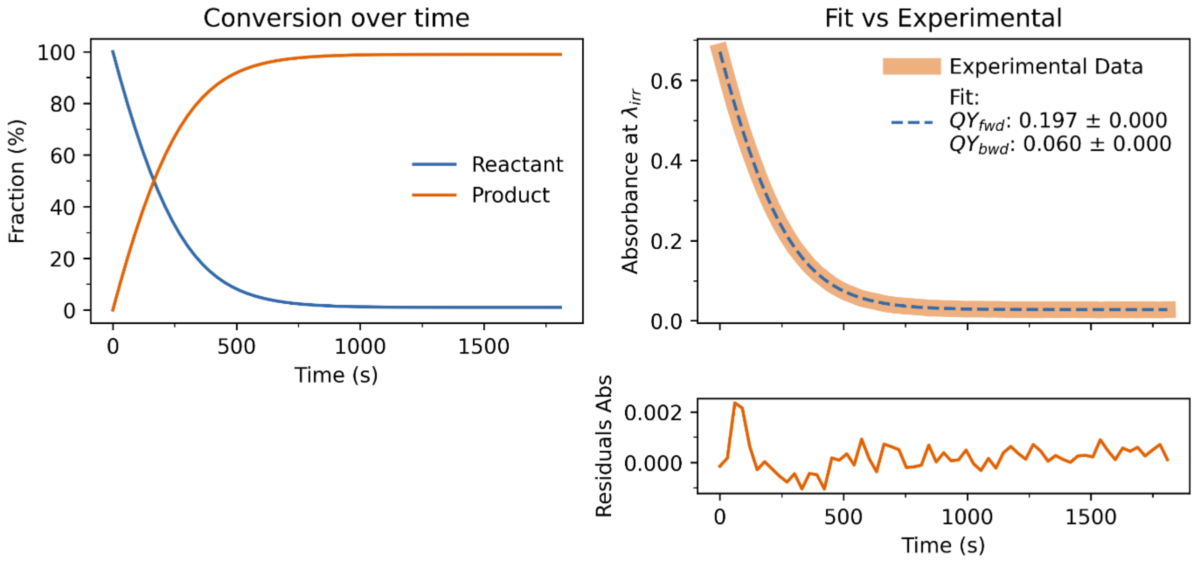

Figure S45: Relative concentration over time of *E*- and *Z*-isomers, experimental absorbance at the irradiation wavelength and fit of the integrated absorbances over the emission spectrum of the LED, and residuals of the fit of the irradiation of **F-(*N*Ph)PAP** in acetonitrile with λ = 340 nm.

Quantum Yields at 455 nm

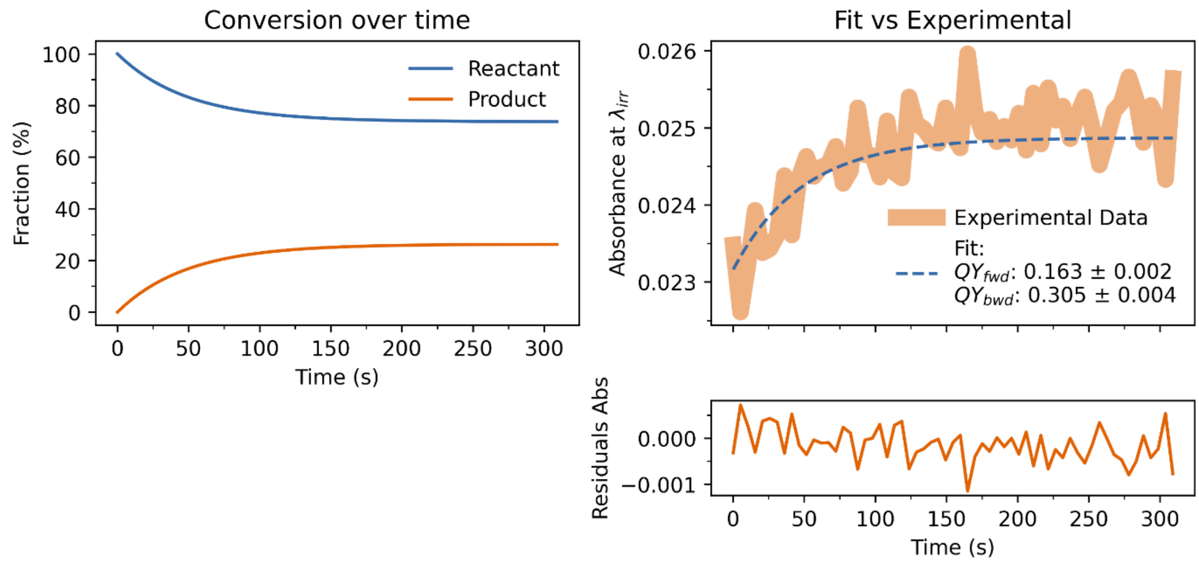

Figure S46: Relative concentration over time of *E*- and *Z*-isomers, experimental absorbance at the irradiation wavelength and fit of the integrated absorbances over the emission spectrum of the LED, and residuals of the fit of the irradiation of **F-PAP-H** in acetonitrile with λ = 455 nm.

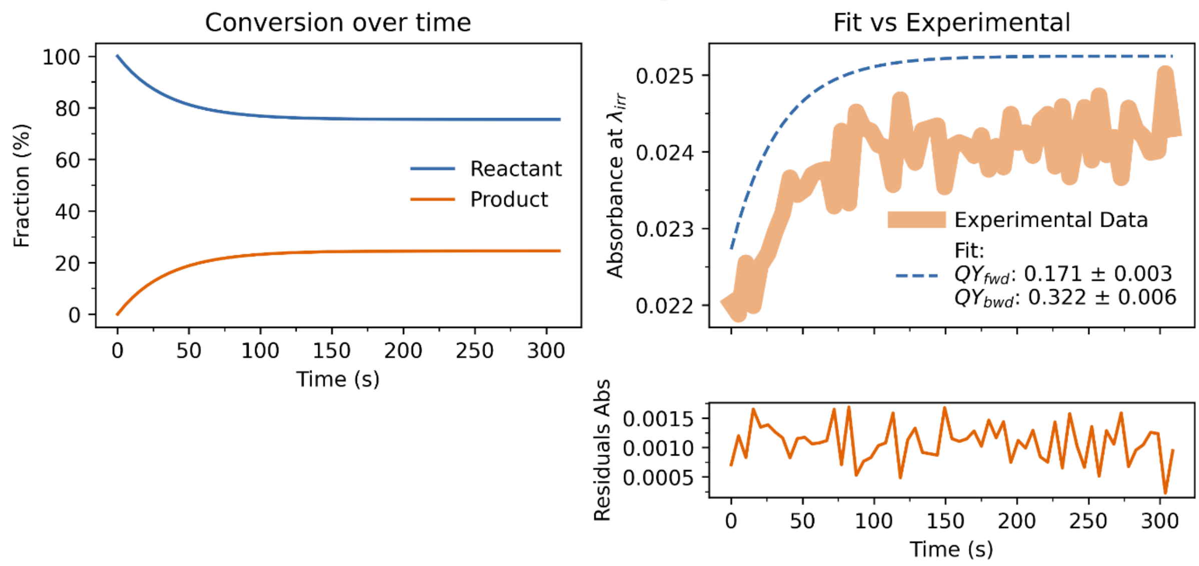

Figure S47: Relative concentration over time of *E*- and *Z*-isomers, experimental absorbance at the irradiation wavelength and fit of the integrated absorbances over the emission spectrum of the LED, and residuals of the fit of the irradiation of **F-PAP-Cl** in acetonitrile with λ = 455 nm.

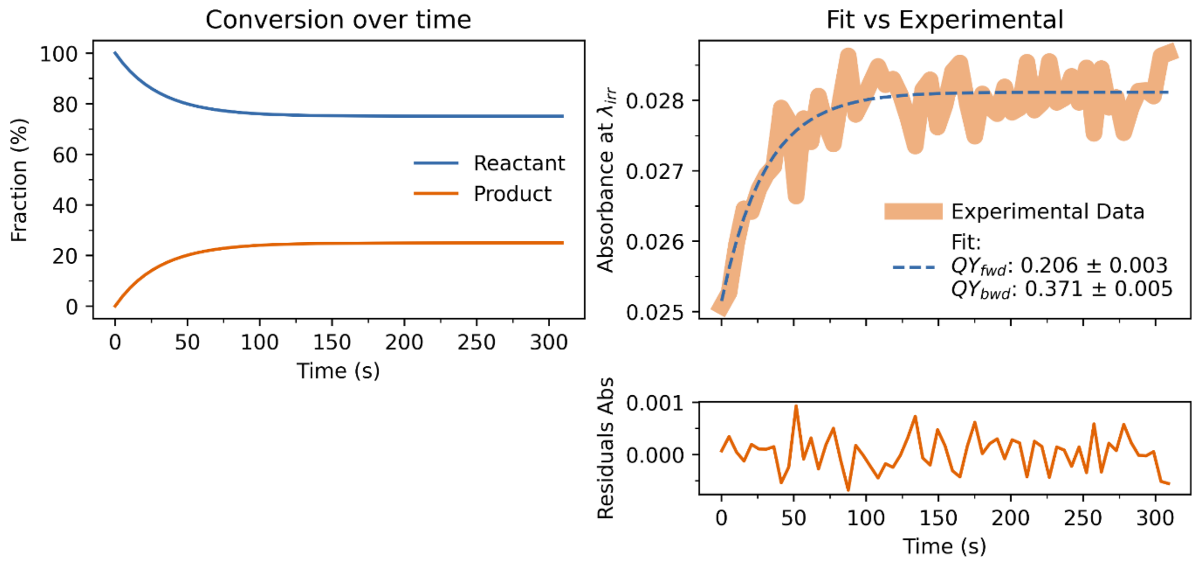

Figure S48: Relative concentration over time of *E*- and *Z*-isomers, experimental absorbance at the irradiation wavelength and fit of the integrated absorbances over the emission spectrum of the LED, and residuals of the fit of the irradiation of **F-PAP-Br** in acetonitrile with λ = 455 nm.

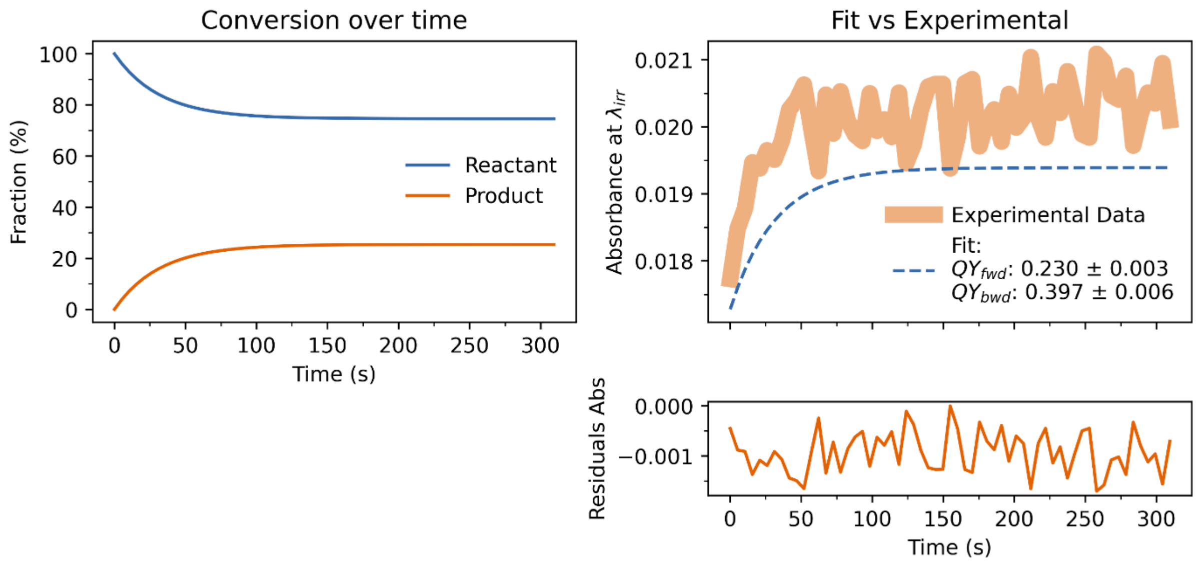

Figure S49: Relative concentration over time of *E*- and *Z*-isomers, experimental absorbance at the irradiation wavelength and fit of the integrated absorbances over the emission spectrum of the LED, and residuals of the fit of the irradiation of **F-PAP-CN** in acetonitrile with λ = 455 nm.

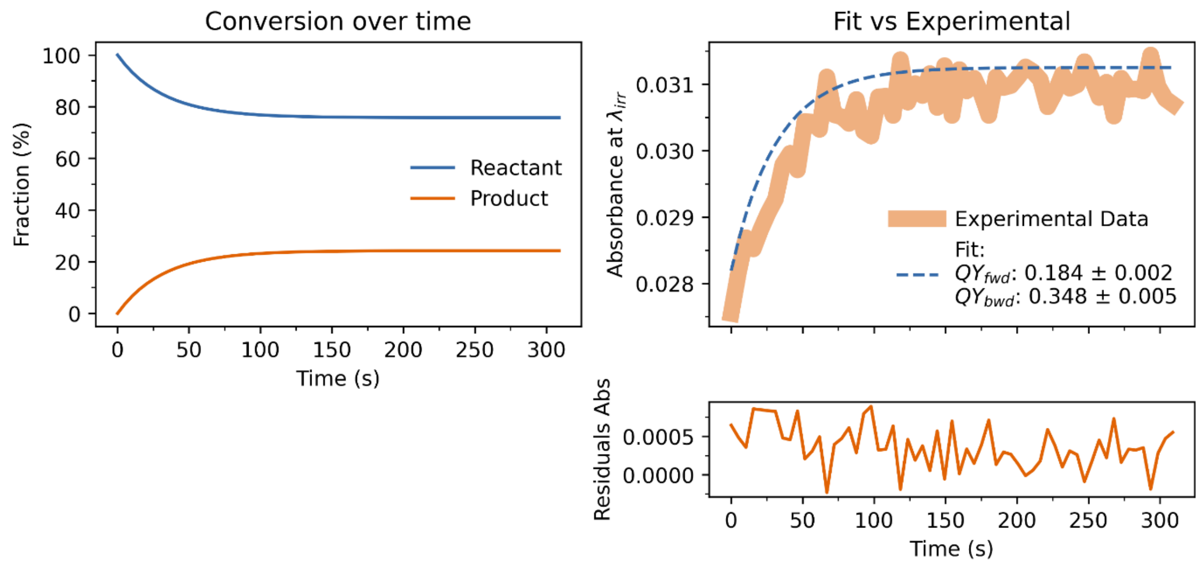

Figure S50: Relative concentration over time of *E*- and *Z*-isomers, experimental absorbance at the irradiation wavelength and fit of the integrated absorbances over the emission spectrum of the LED, and residuals of the fit of the irradiation of **F-PAP-Me** in acetonitrile with λ = 455 nm.

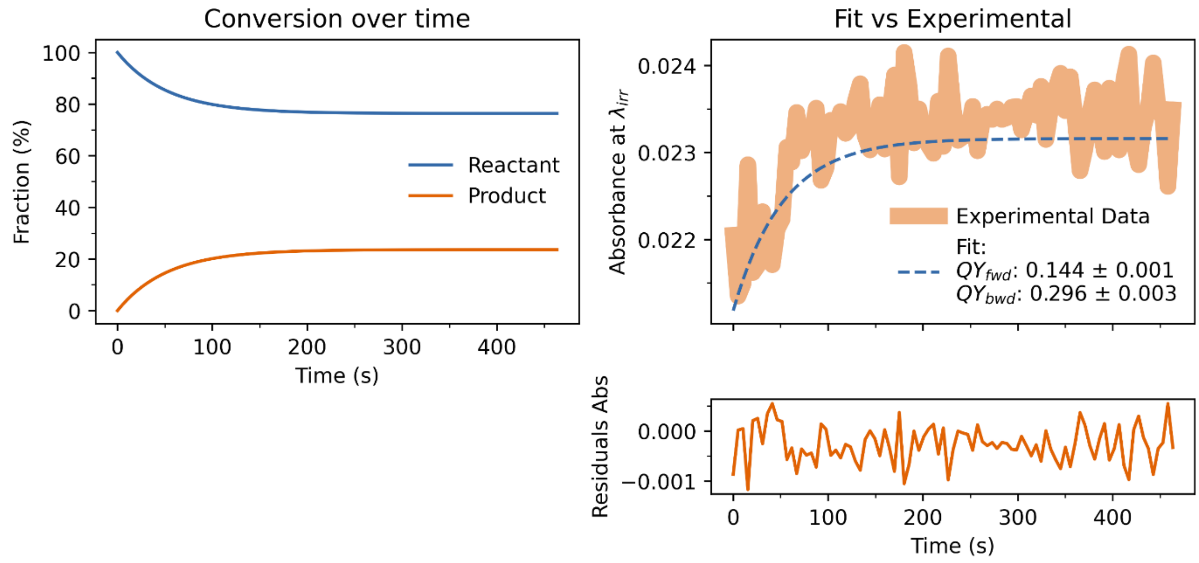

Figure S51: Relative concentration over time of *E*- and *Z*-isomers, experimental absorbance at the irradiation wavelength and fit of the integrated absorbances over the emission spectrum of the LED, and residuals of the fit of the irradiation of **F-PAP-CF$_3$** in acetonitrile with λ = 455 nm.

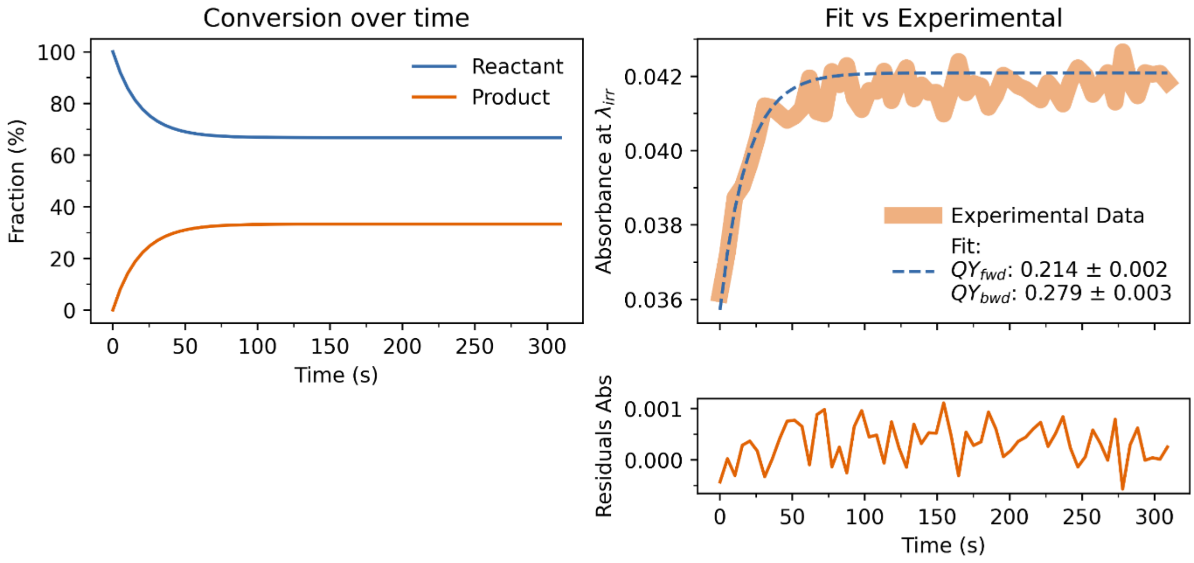

Figure S52: Relative concentration over time of *E*- and *Z*-isomers, experimental absorbance at the irradiation wavelength and fit of the integrated absorbances over the emission spectrum of the LED, and residuals of the fit of the irradiation of **F-PAP-OMe** in acetonitrile with λ = 455 nm.

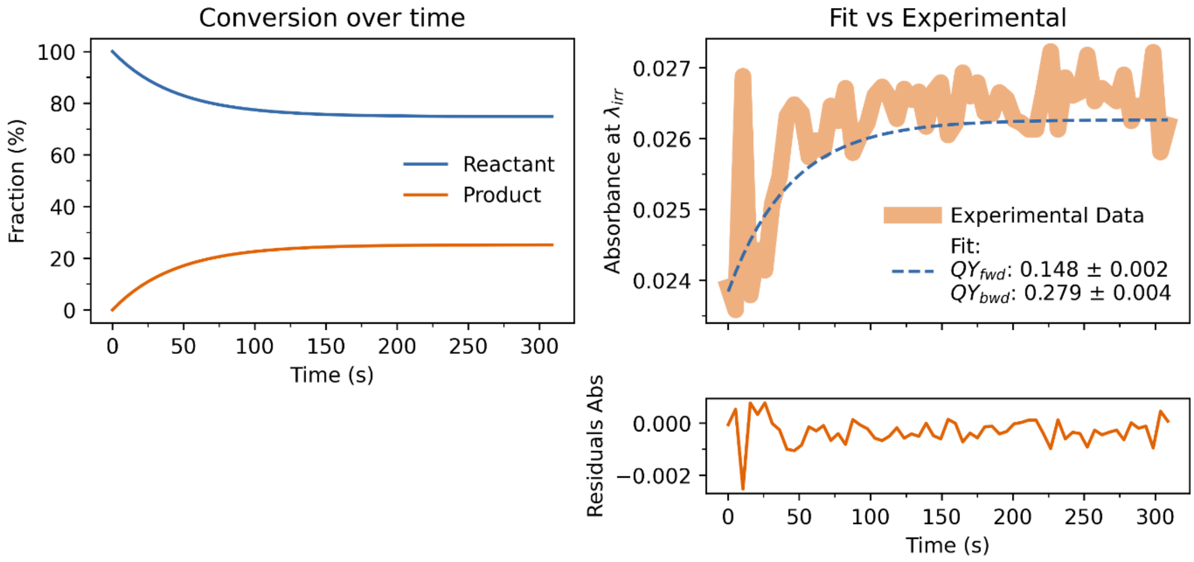

Figure S53: Relative concentration over time of *E*- and *Z*-isomers, experimental absorbance at the irradiation wavelength and fit of the integrated absorbances over the emission spectrum of the LED, and residuals of the fit of the irradiation of **F-PAP-OCF$_3$** in acetonitrile with λ = 455 nm.

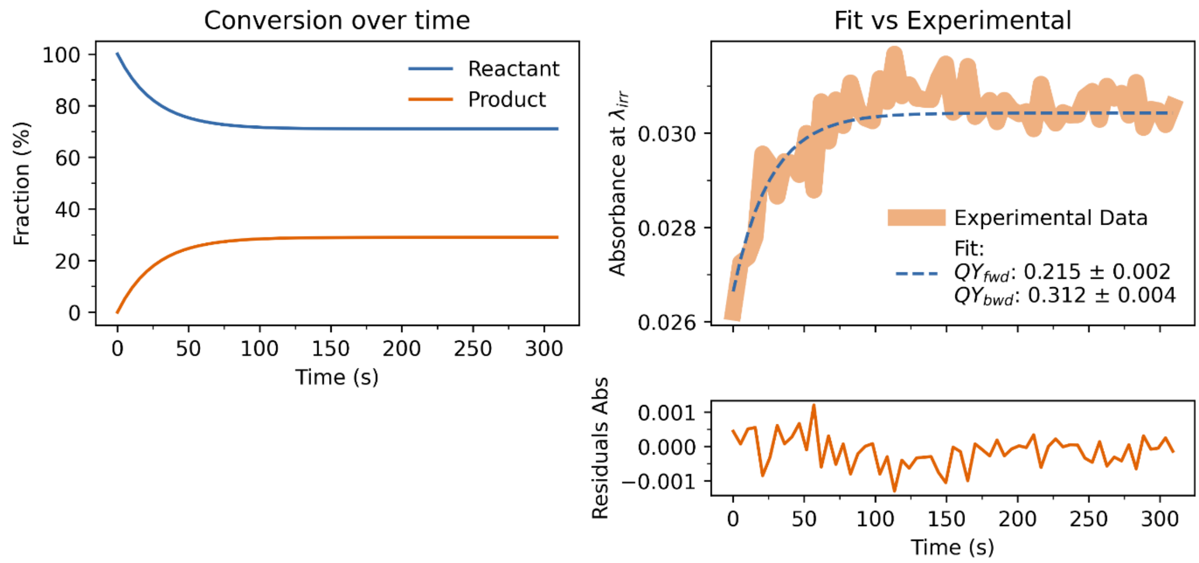

Figure S54: Relative concentration over time of *E*- and *Z*-isomers, experimental absorbance at the irradiation wavelength and fit of the integrated absorbances over the emission spectrum of the LED, and residuals of the fit of the irradiation of **F-PAP-CCH** in acetonitrile with λ = 455 nm.

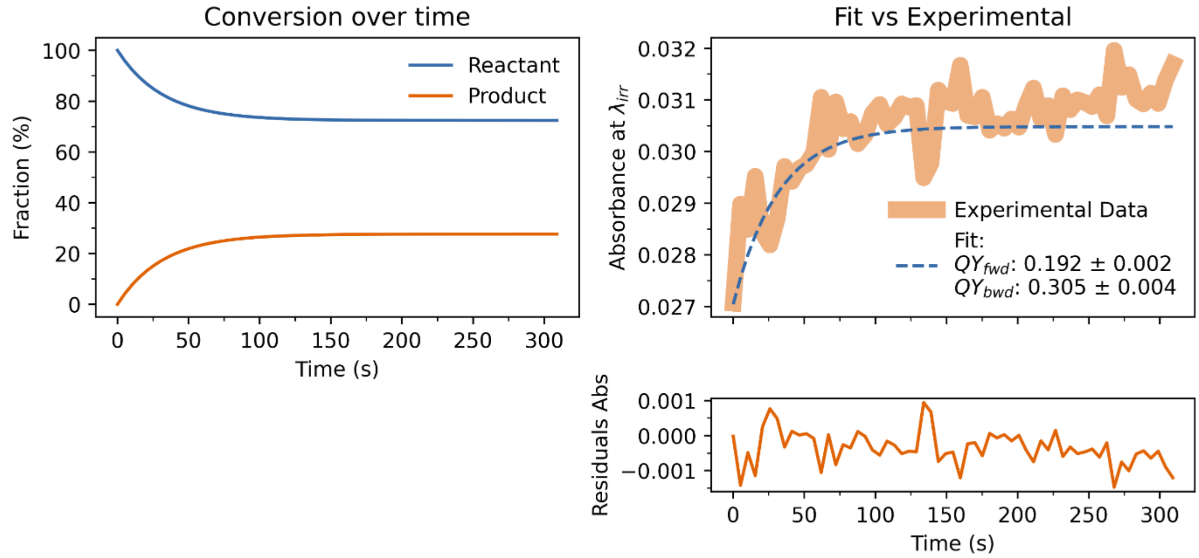

Figure S55: Relative concentration over time of *E*- and *Z*-isomers, experimental absorbance at the irradiation wavelength and fit of the integrated absorbances over the emission spectrum of the LED, and residuals of the fit of the irradiation of **F-PAP-*n*Bu** in acetonitrile with λ = 455 nm.

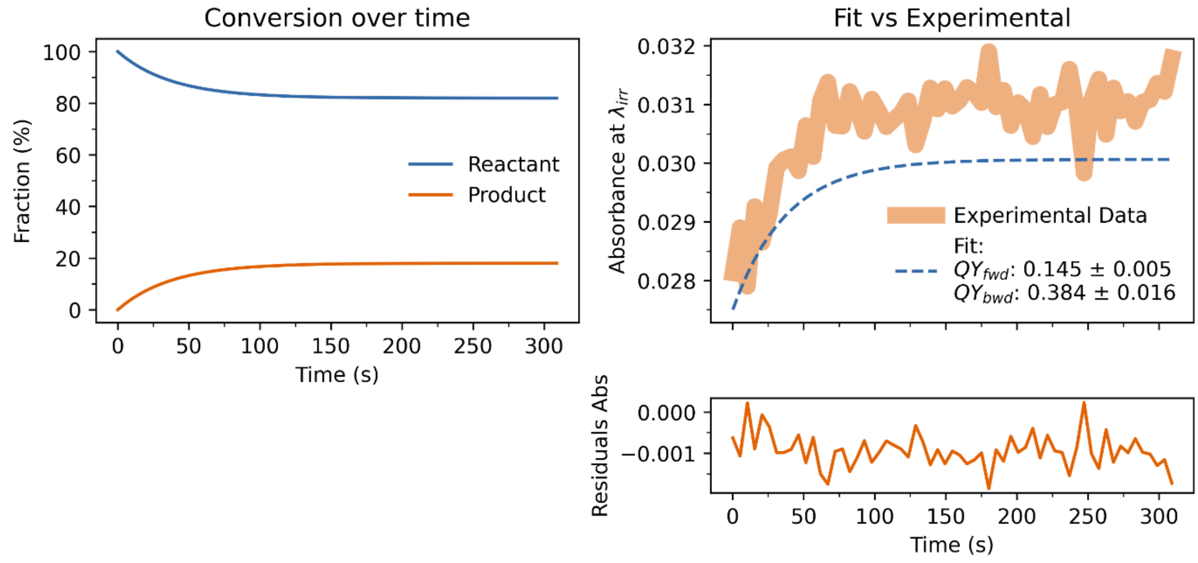

Figure S56: Relative concentration over time of *E*- and *Z*-isomers, experimental absorbance at the irradiation wavelength and fit of the integrated absorbances over the emission spectrum of the LED, and residuals of the fit of the irradiation of **F-PAP-C_3H_6COOH** in acetonitrile with λ = 455 nm.

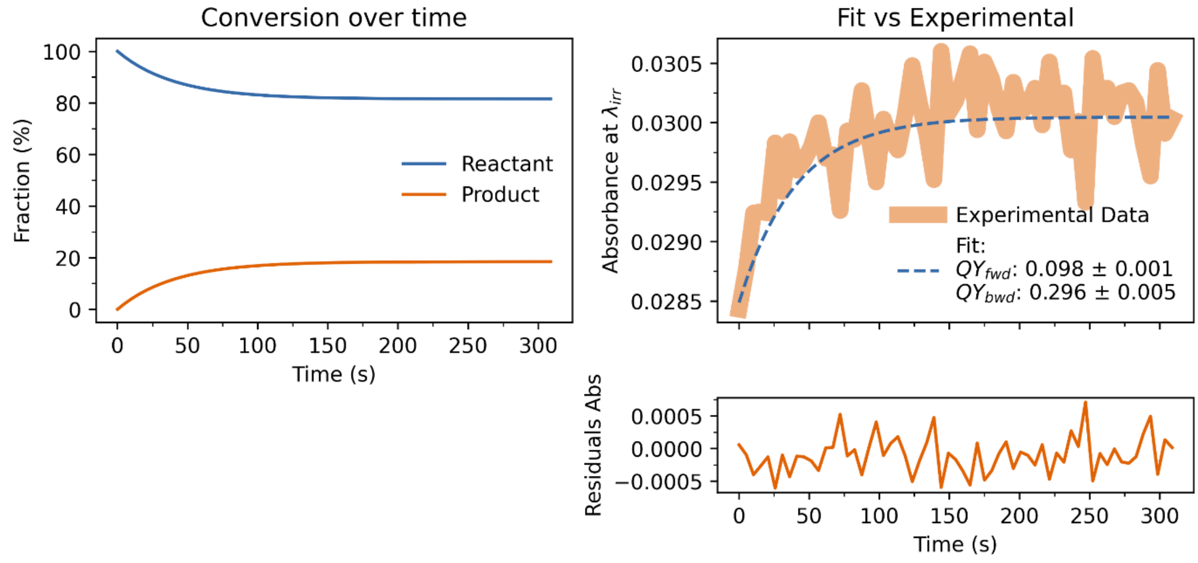

Figure S57: Relative concentration over time of *E*- and *Z*-isomers, experimental absorbance at the irradiation wavelength and fit of the integrated absorbances over the emission spectrum of the LED, and residuals of the fit of the irradiation of **F-PAP-NO_2** in acetonitrile with λ = 455 nm.

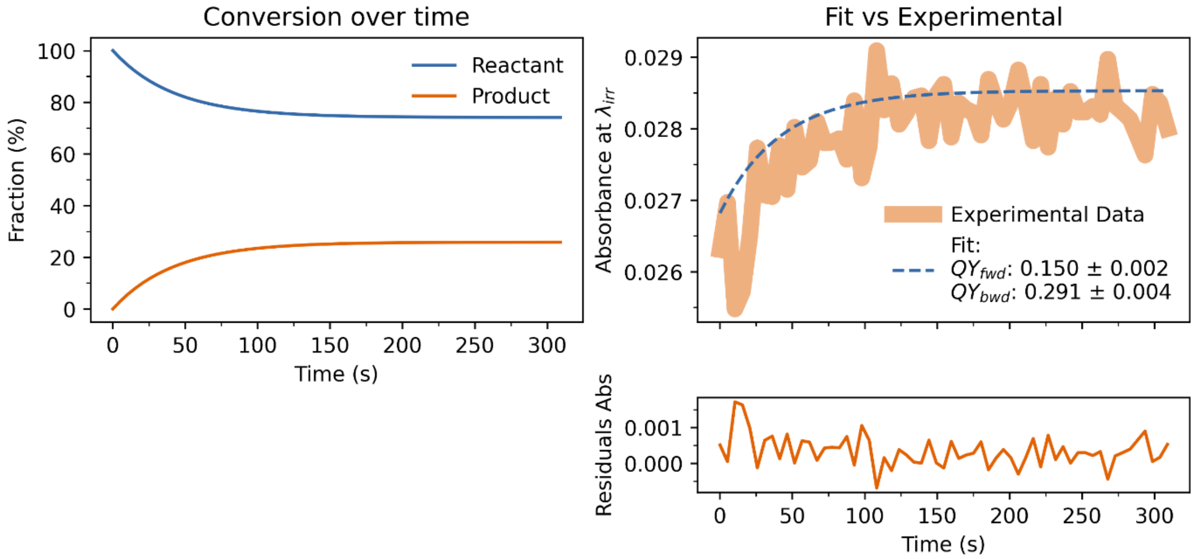

Figure S58: Relative concentration over time of *E*- and *Z*-isomers, experimental absorbance at the irradiation wavelength and fit of the integrated absorbances over the emission spectrum of the LED, and residuals of the fit of the irradiation of **F-(*N*Ph)PAP** in acetonitrile with λ = 455 nm.

3.6. Photochemical Isomerization in ^{1}H and ^{19}F NMR Spectroscopy

F-PAP-H: (*E*)-1-Methyl-4-(phenyldiazenyl)-3,5-bis(trifluoromethyl)-1*H*-pyrazole

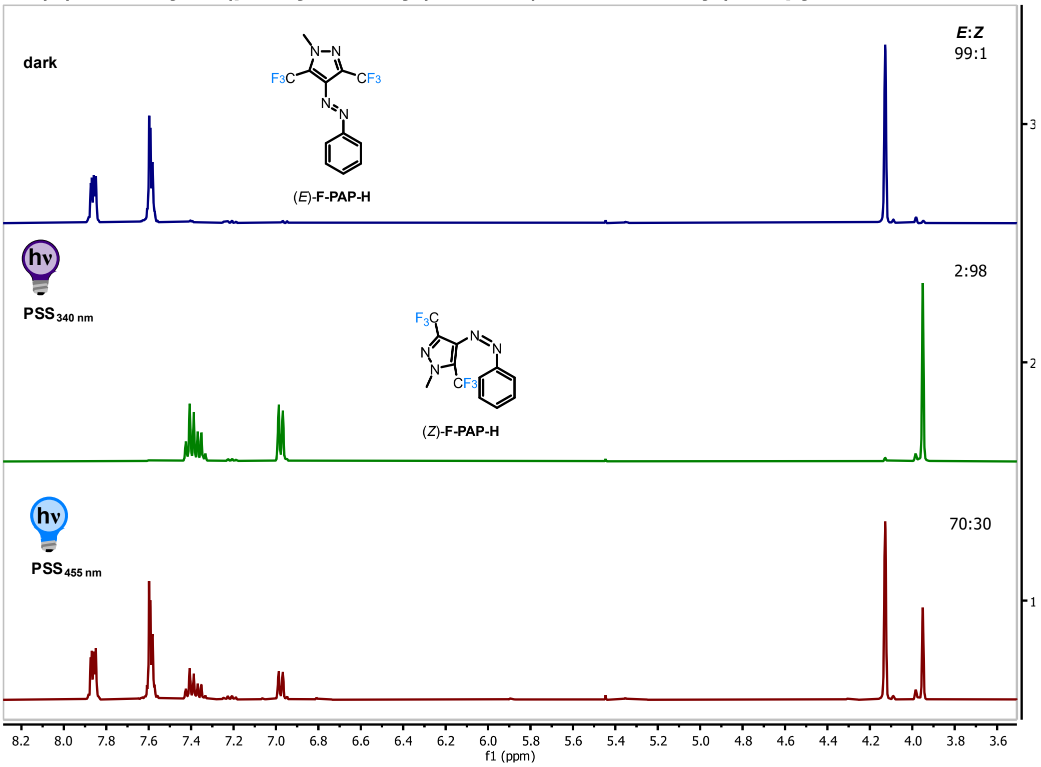

Figure S59: ^{1}H-NMR (399.88 MHz, CD_3CN, 295 K) of **F-PAP-H**. The upper trace shows the spectrum before irradiation, the middle trace shows the spectrum at $PSS_{340\ nm}$, and the lower trace shows the spectrum at $PSS_{455\ nm}$

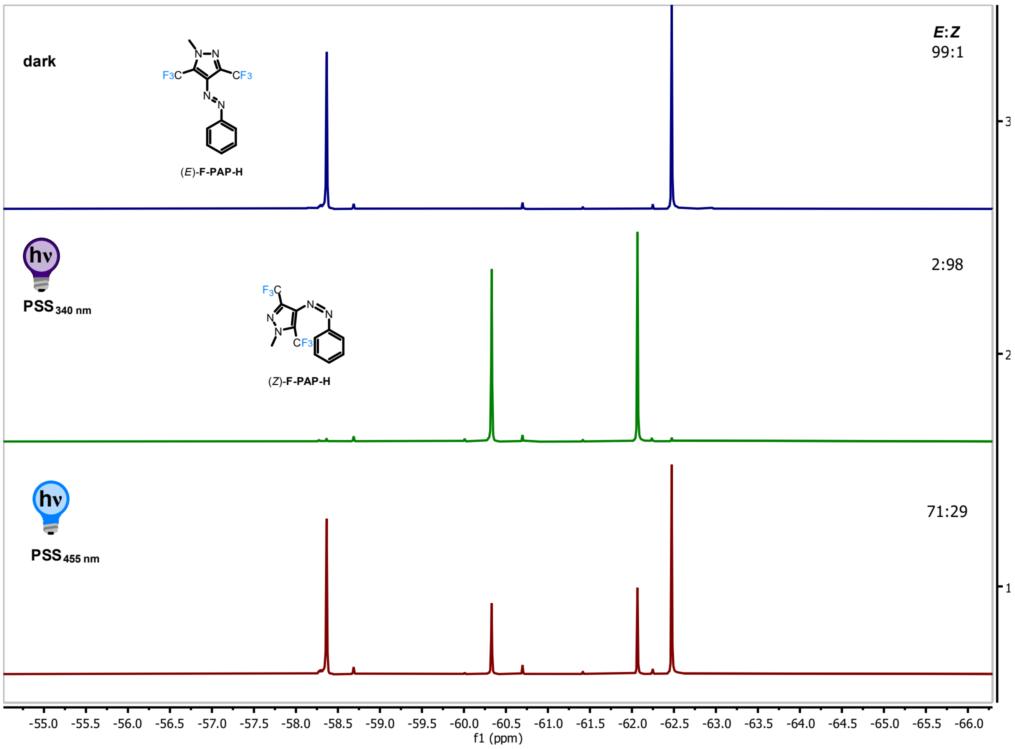

Figure S60: ^{19}F-NMR (376.27 MHz, CD_3CN, 295 K) of **F-PAP-H**. The upper trace shows the spectrum before irradiation, the middle trace shows the spectrum at $PSS_{340\ nm}$, and the lower trace shows the spectrum at $PSS_{455\ nm}$.

F-PAP-Cl: (*E*)-4-((4-Chlorophenyl)diazenyl)-1-methyl-3,5-bis(trifluoromethyl)-1*H*-pyrazole

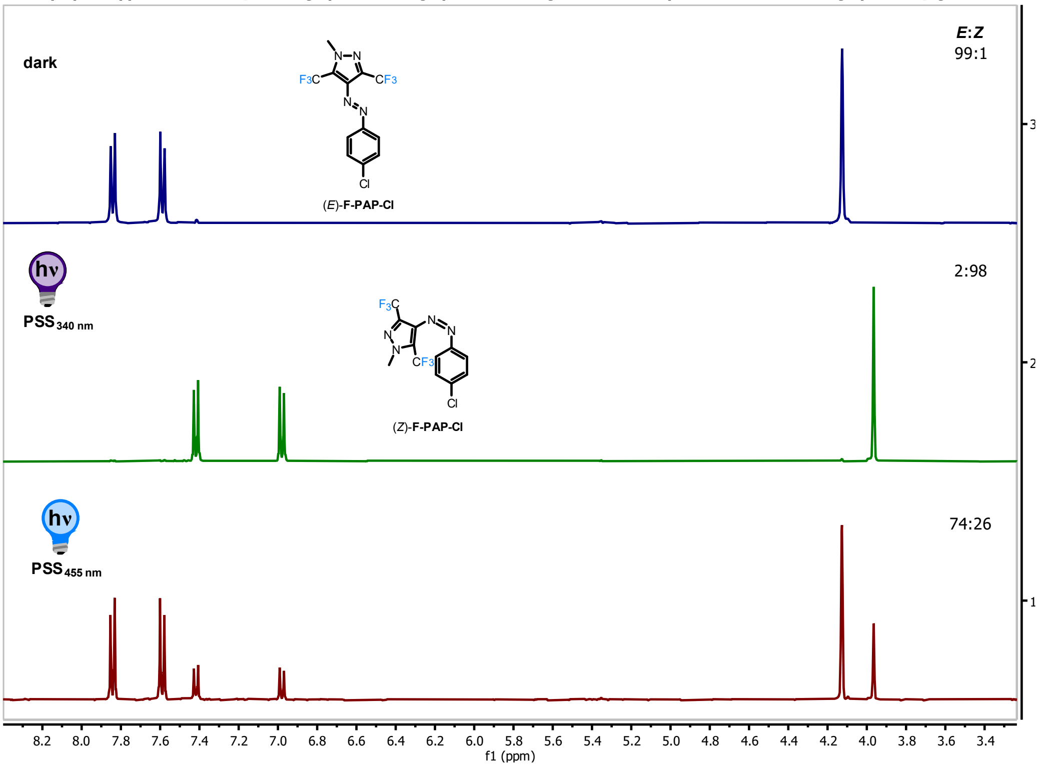

Figure S61: ^{1}H-NMR (399.88 MHz, CD_3CN, 295 K) of **F-PAP-Cl**. The upper trace shows the spectrum before irradiation, the middle trace shows the spectrum at $PSS_{340\ nm}$, and the lower trace shows the spectrum at $PSS_{455\ nm}$.

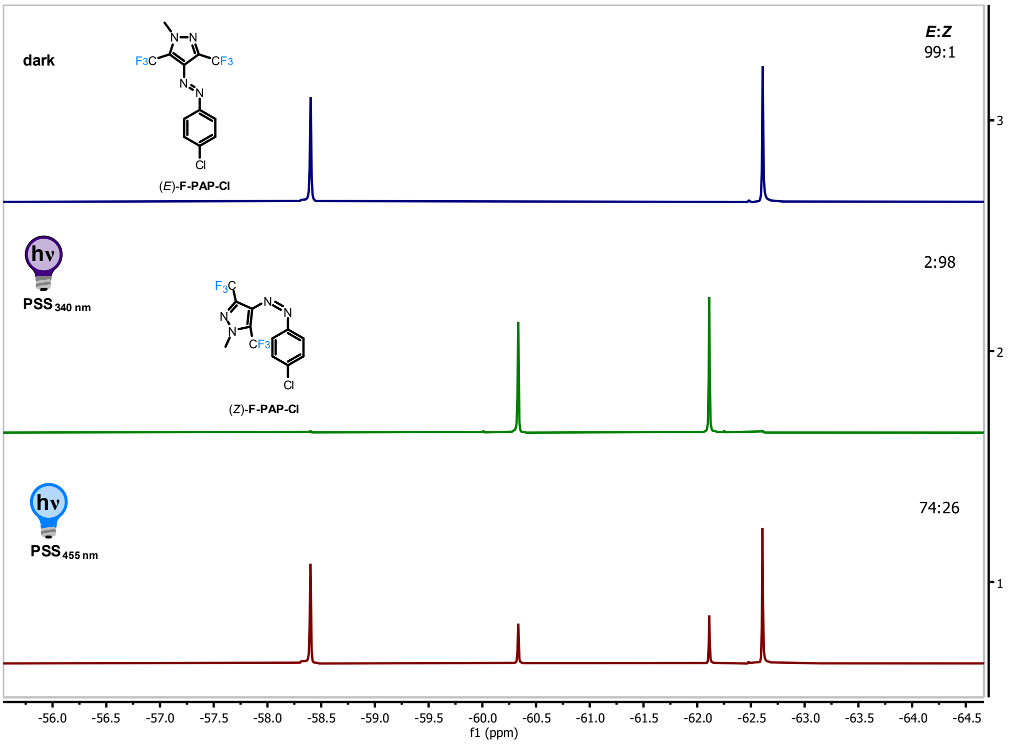

Figure S62: ^{19}F-NMR (376.27 MHz, CD_3CN, 295 K) of **F-PAP-Cl**. The upper trace shows the spectrum before irradiation, the middle trace shows the spectrum at $PSS_{340\ nm}$, and the lower trace shows the spectrum at $PSS_{455\ nm}$.

F-PAP-Br: (*E*)-4-((4-Bromophenyl)diazenyl)-1-methyl-3,5-bis(trifluoromethyl)-1*H*-pyrazole

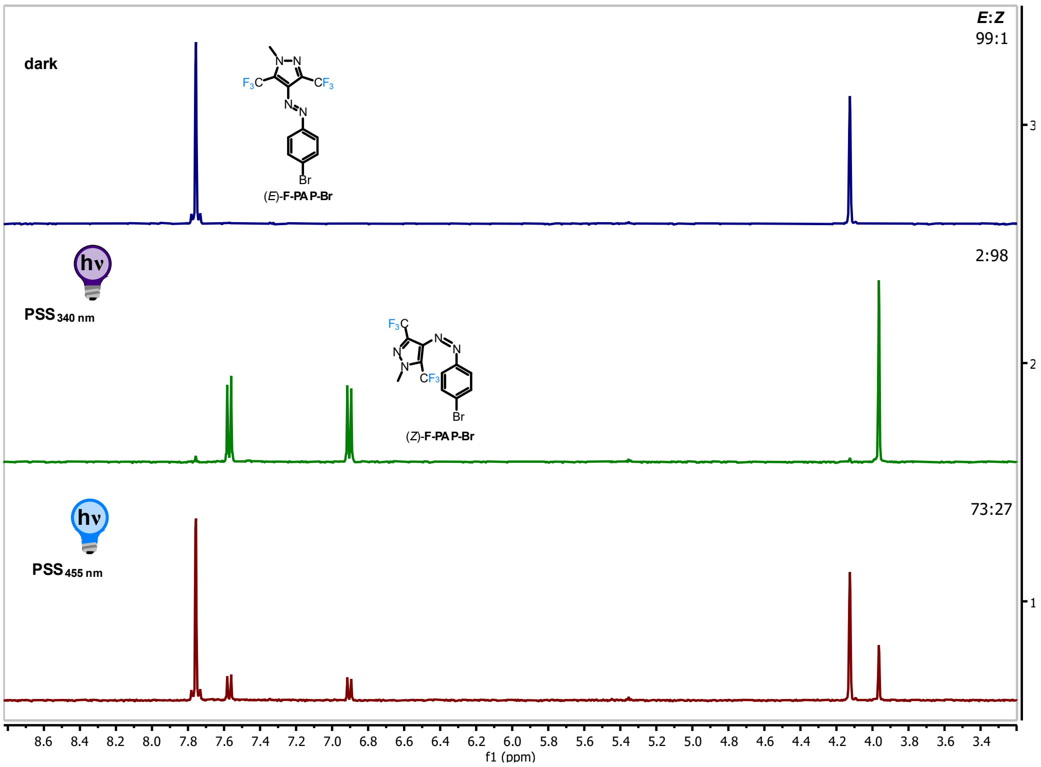

Figure S63: ^{1}H-NMR (399.88 MHz, CD_3CN, 295 K) of **F-PAP-Br**. The upper trace shows the spectrum before irradiation, the middle trace shows the spectrum at $PSS_{340\ nm}$, and the lower trace shows the spectrum at $PSS_{455\ nm}$.

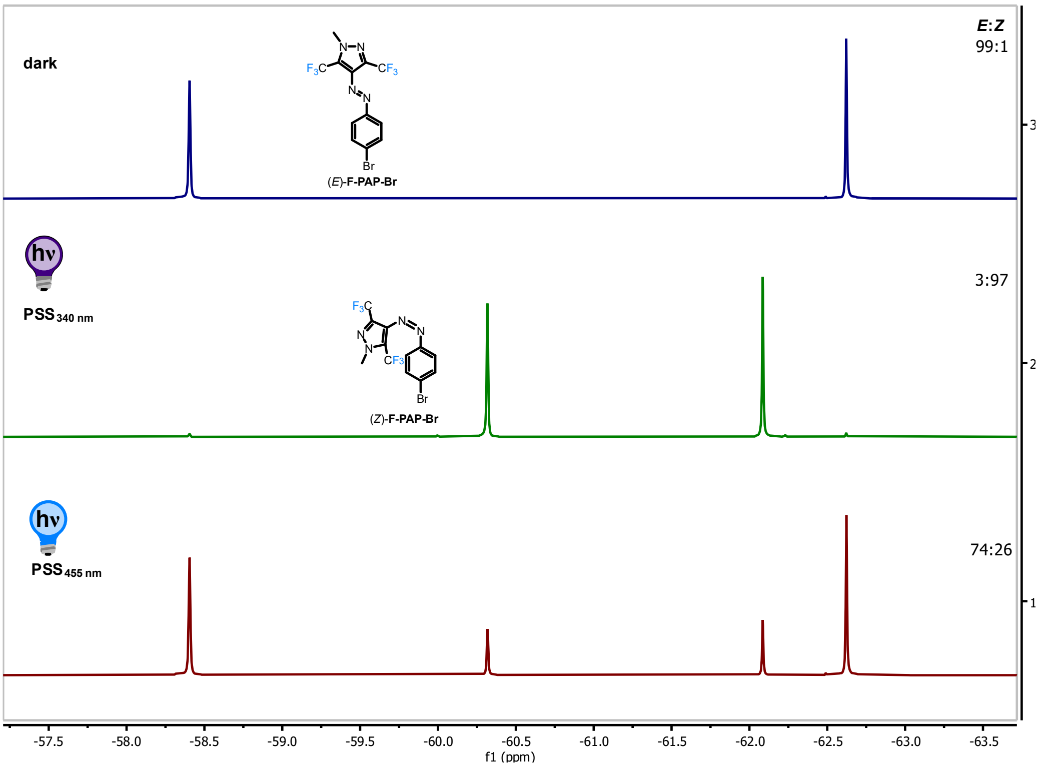

Figure S64: ^{19}F-NMR (376.27 MHz, CD_3CN, 295 K) of **F-PAP-Br**. The upper trace shows the spectrum before irradiation, the middle trace shows the spectrum at $PSS_{340\ nm}$, and the lower trace shows the spectrum at $PSS_{455\ nm}$.

F-PAP-CN: (*E*)-4-((1-Methyl-3,5-bis(trifluoromethyl)-1*H*-pyrazol-4-yl)diazenyl)benzonitrile

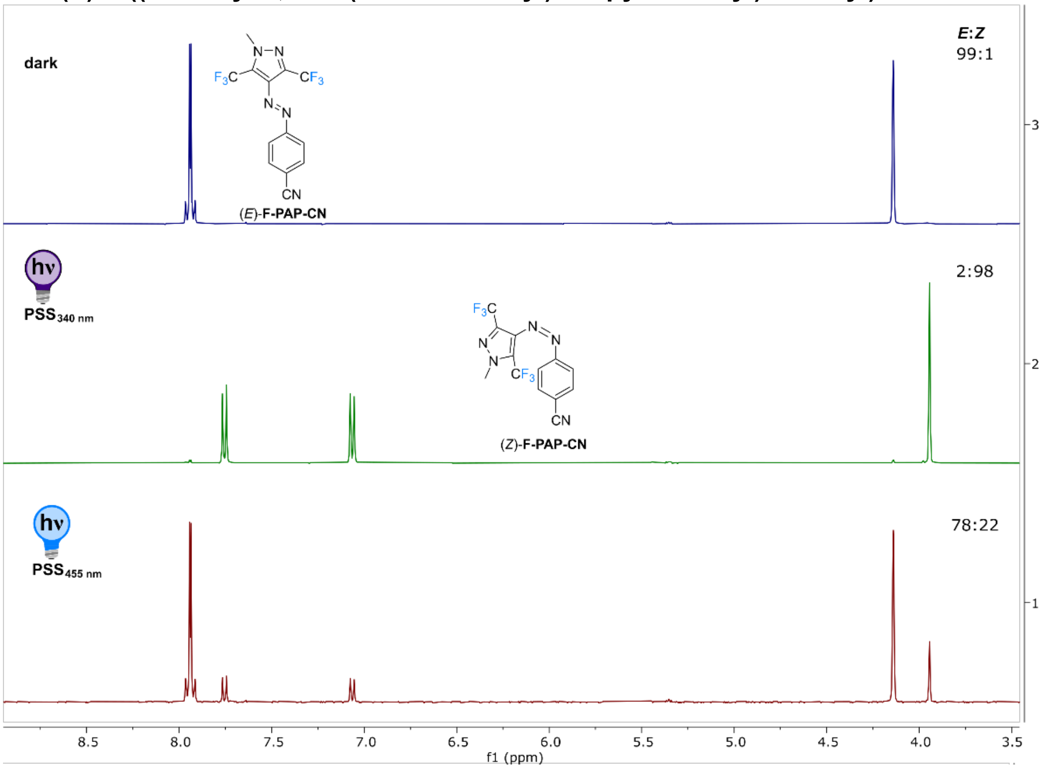

Figure S65: ^{1}H-NMR (399.88 MHz, CD_3CN, 295 K) of **F-PAP-CN**. The upper trace shows the spectrum before irradiation, the middle trace shows the spectrum at $PSS_{340\ nm}$, and the lower trace shows the spectrum at $PSS_{455\ nm}$.

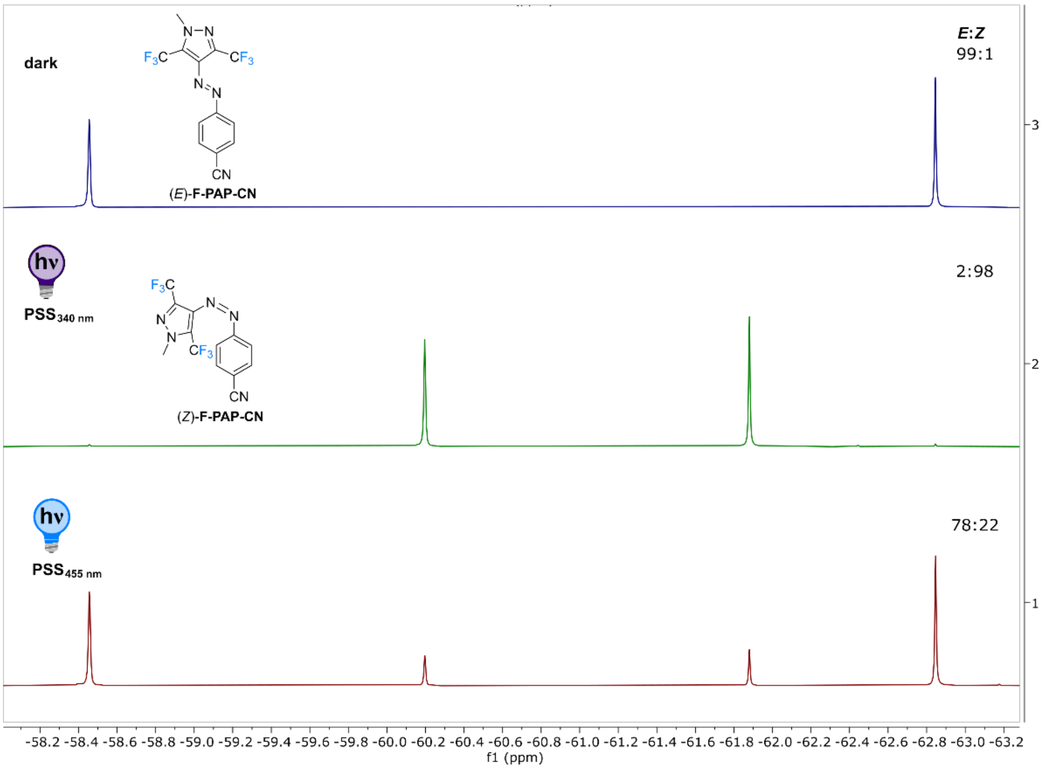

Figure S66: ^{19}F-NMR (376.27 MHz, CD_3CN, 295 K) of **F-PAP-CN**. The upper trace shows the spectrum before irradiation, the middle trace shows the spectrum at $PSS_{340\ nm}$, and the lower trace shows the spectrum at $PSS_{455\ nm}$.

F-PAP-Me: (*E*)-1-Methyl-4-(p-tolyldiazenyl)-3,5-bis(trifluoromethyl)-1*H*-pyrazole

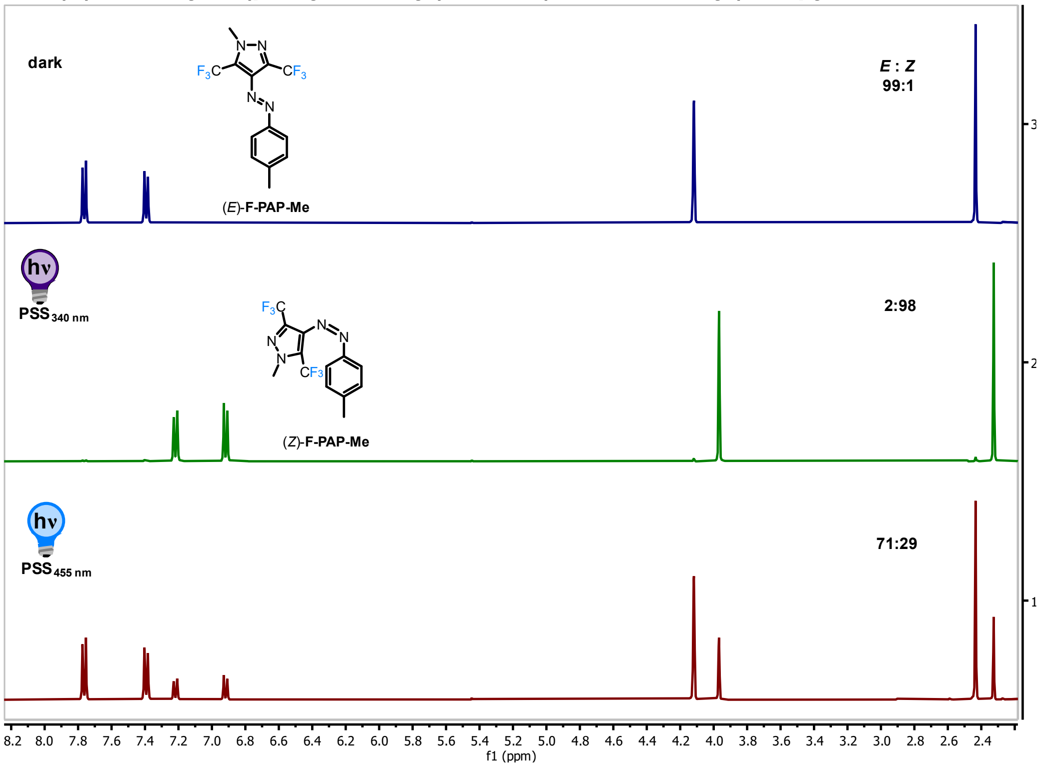

Figure S67: ^{1}H-NMR (399.88 MHz, CD_3CN, 295 K) of **F-PAP-Me**. The upper trace shows the spectrum before irradiation, the middle trace shows the spectrum at $PSS_{340\ nm}$, and the lower trace shows the spectrum at $PSS_{455\ nm}$.

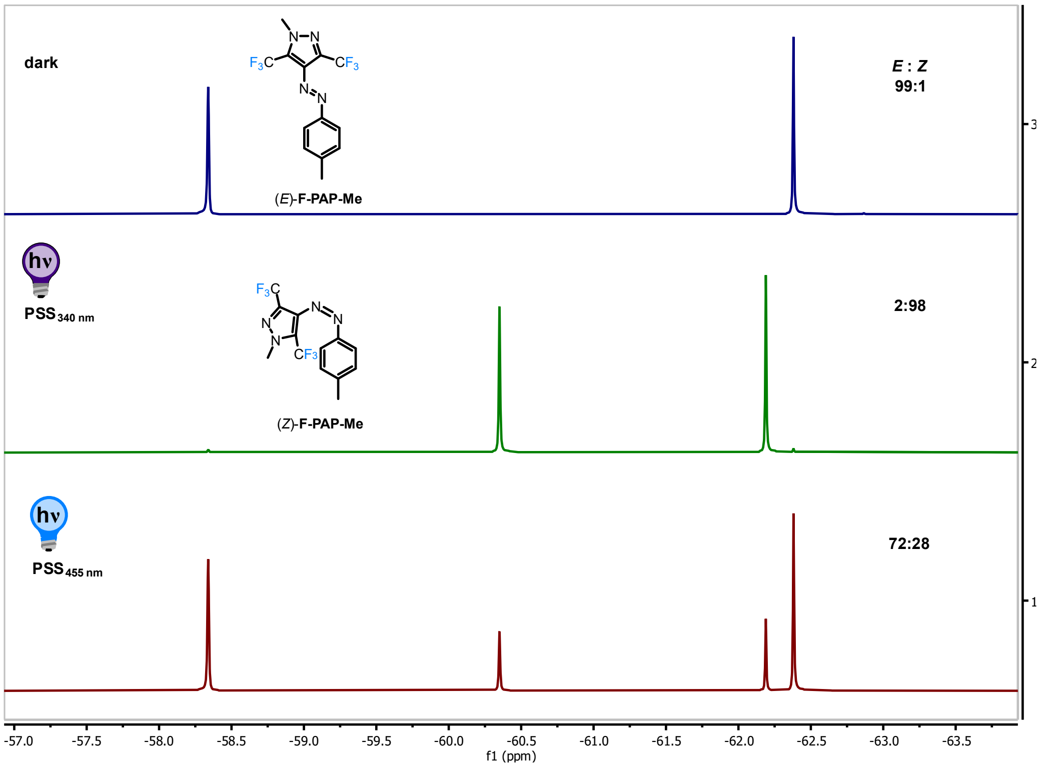

Figure S68: ^{19}F-NMR (376.27 MHz, CD_3CN, 295 K) of **F-PAP-Me**. The upper trace shows the spectrum before irradiation, the middle trace shows the spectrum at $PSS_{340\ nm}$, and the lower trace shows the spectrum at $PSS_{455\ nm}$.

F-PAP-CF$_3$: (*E*)-1-Methyl-3,5-bis(trifluoromethyl)-4-((4 (trifluoromethyl)phenyl)diazenyl)-1*H*-pyrazole

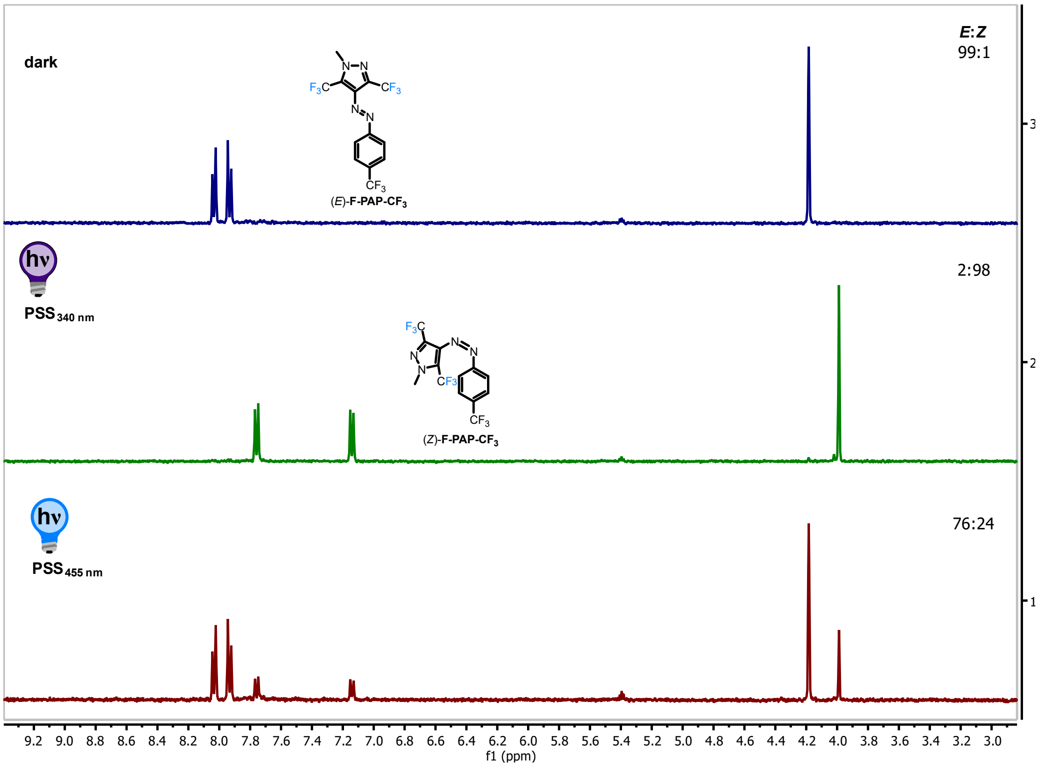

Figure S69: ^{1}H-NMR (399.88 MHz, CD_3CN, 295 K) of **F-PAP-CF$_3$**. The upper trace shows the spectrum before irradiation, the middle trace shows the spectrum at $PSS_{340\ nm}$, and the lower trace shows the spectrum at $PSS_{455\ nm}$.

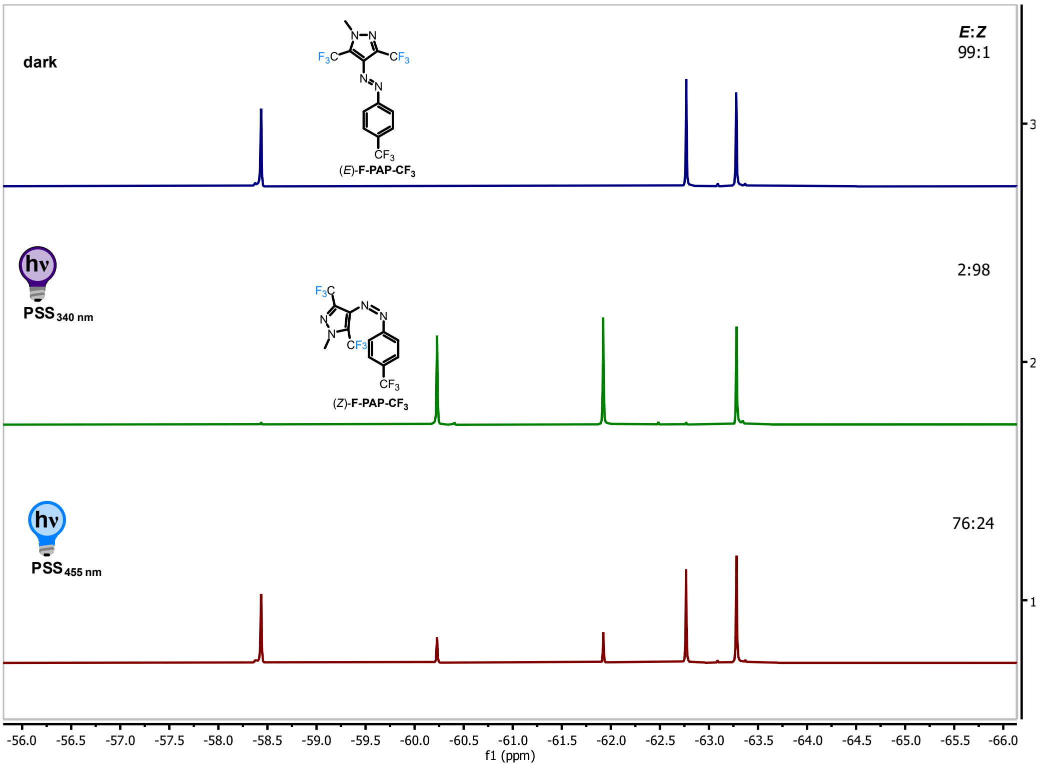

Figure S70: ^{19}F-NMR (376.27 MHz, CD_3CN, 295 K) of **F-PAP-CF$_3$**. The upper trace shows the spectrum before irradiation, the middle trace shows the spectrum at $PSS_{340\ nm}$, and the lower trace shows the spectrum at $PSS_{455\ nm}$.

F-PAP-OMe: (*E*)-4-((4-Methoxyphenyl)diazenyl)-1-methyl-3,5-bis(trifluoromethyl)-1*H*-pyrazole

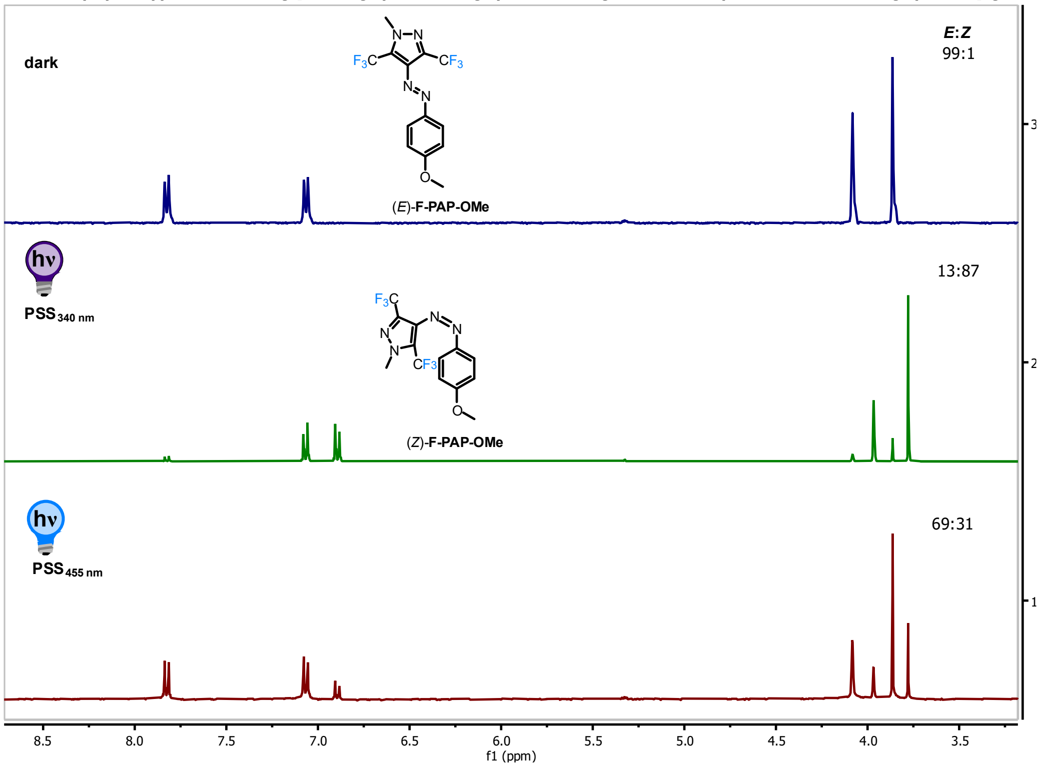

Figure S71:^{1}H-NMR (399.88 MHz, CD_3CN, 295 K) of **F-PAP-OMe**. The upper trace shows the spectrum before irradiation, the middle trace shows the spectrum at $PSS_{340\ nm}$, and the lower trace shows the spectrum at $PSS_{455\ nm}$.

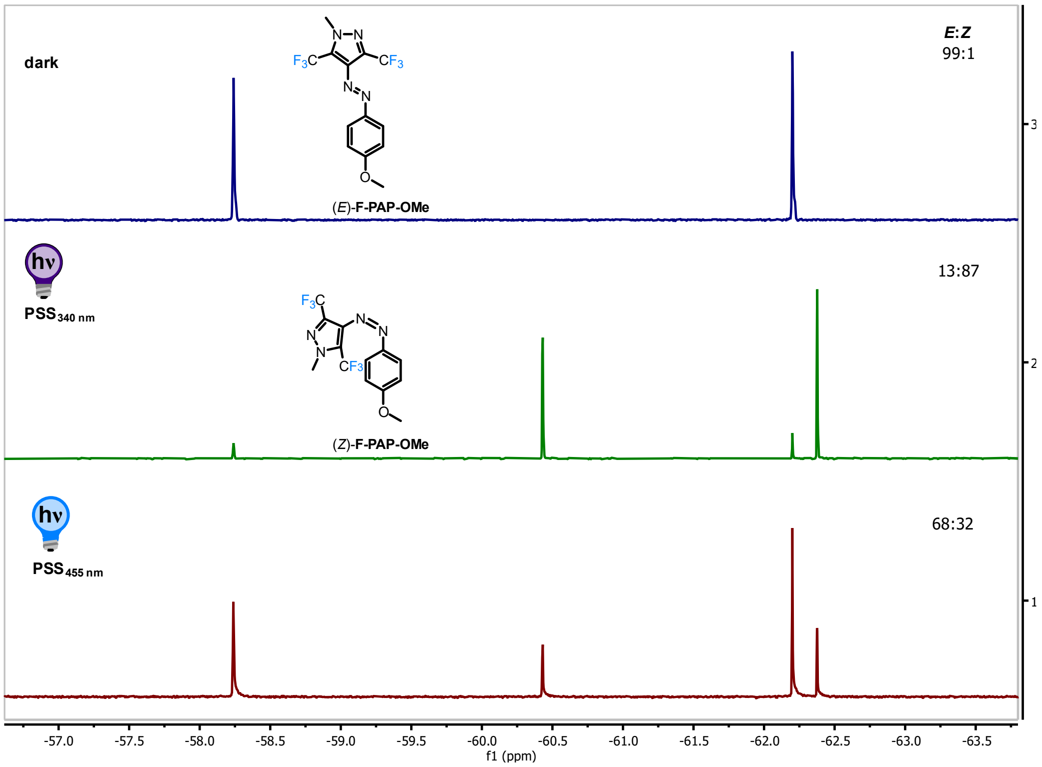

Figure S72:^{19}F-NMR (376.27 MHz, CD_3CN, 295 K) of **F-PAP-OMe**. The upper trace shows the spectrum before irradiation, the middle trace shows the spectrum at $PSS_{340\ nm}$, and the lower trace shows the spectrum at $PSS_{455\ nm}$.

F-PAP-OCF$_3$: (*E*)-1-Methyl-4-((4-(trifluoromethoxy)phenyl)diazenyl)-3,5-bis(trifluoromethyl)-1*H*-pyrazole

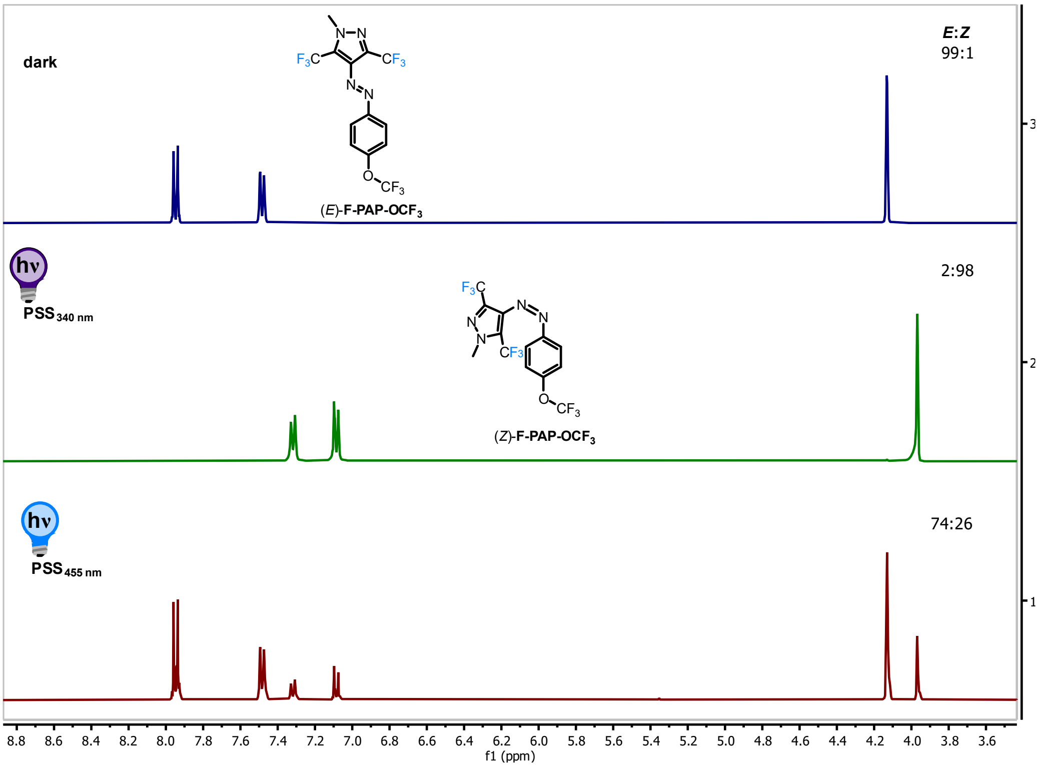

Figure S73: ^{1}H-NMR (399.88 MHz, CD_3CN, 295 K) of **F-PAP-OCF$_3$**. The upper trace shows the spectrum before irradiation, the middle trace shows the spectrum at PSS$_{340\ nm}$, and the lower trace shows the spectrum at PSS$_{455\ nm}$.

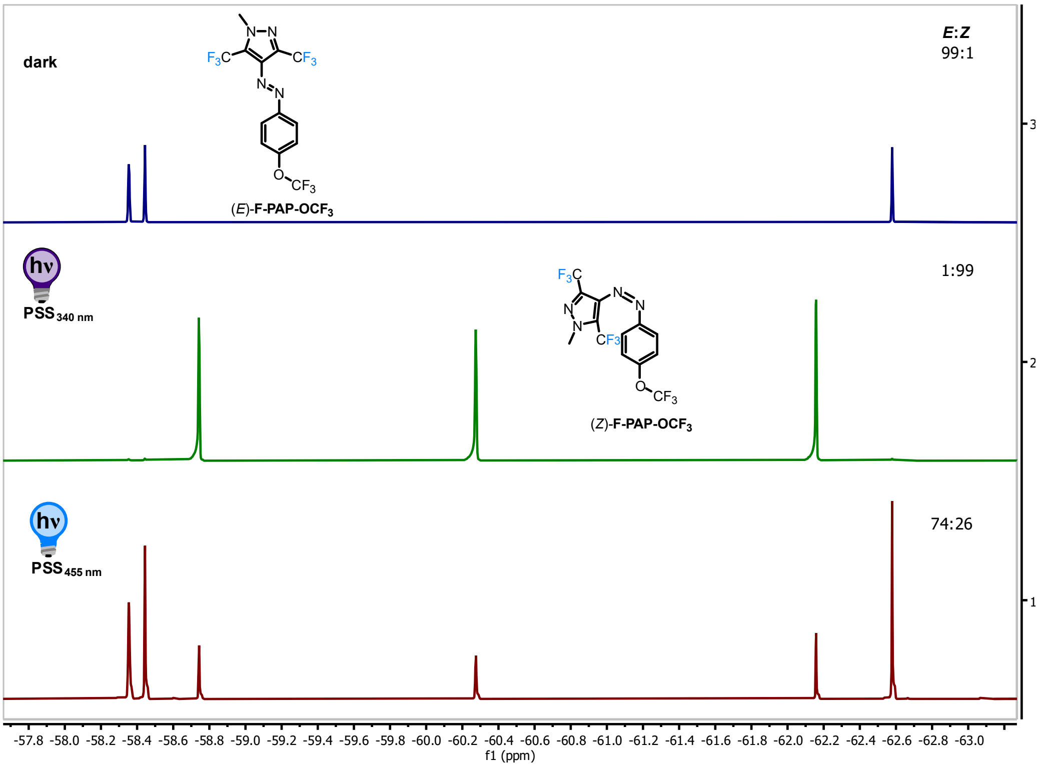

Figure S74: ^{19}F-NMR (376.27 MHz, CD_3CN, 295 K) of **F-PAP-OCF$_3$**. The upper trace shows the spectrum before irradiation, the middle trace shows the spectrum at PSS$_{340\ nm}$, and the lower trace shows the spectrum at PSS$_{455\ nm}$.

F-PAP-CCH: (*E*)-4-((4-Ethynylphenyl)diazenyl)-1-methyl-3,5-bis(trifluoromethyl)-1*H*-pyrazole

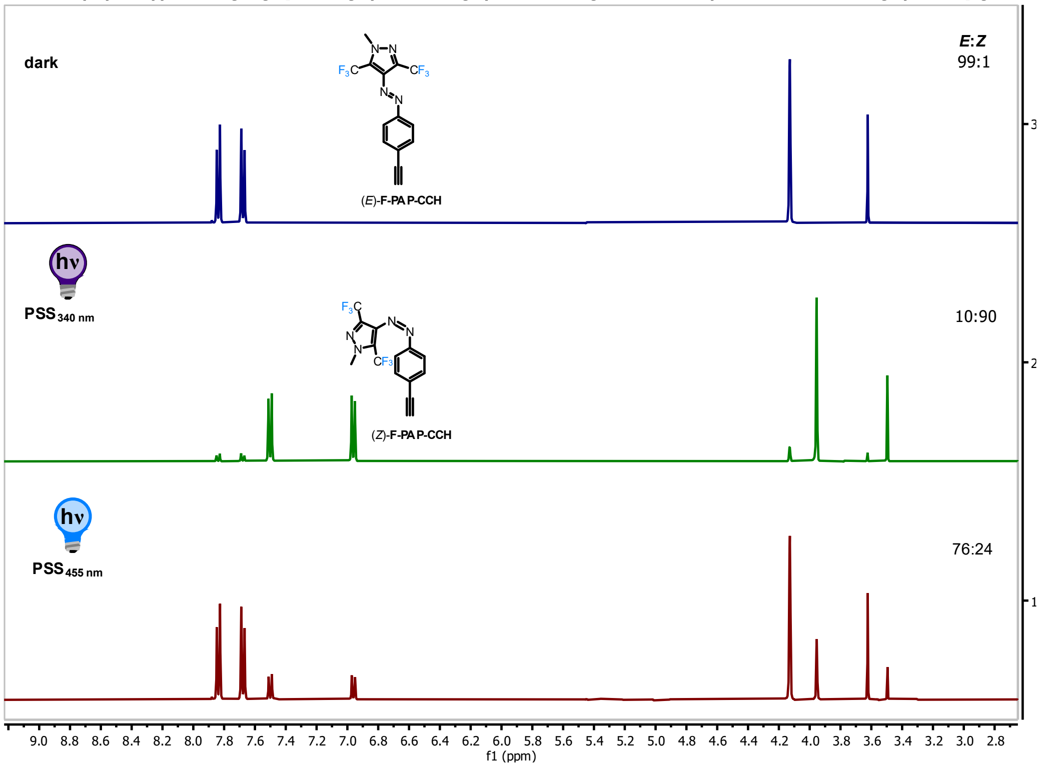

Figure S75: ^{1}H-NMR (399.88 MHz, CD_3CN, 295 K) of **F-PAP-CCH**. The upper trace shows the spectrum before irradiation, the middle trace shows the spectrum at $PSS_{340\ nm}$, and the lower trace shows the spectrum at $PSS_{455\ nm}$.

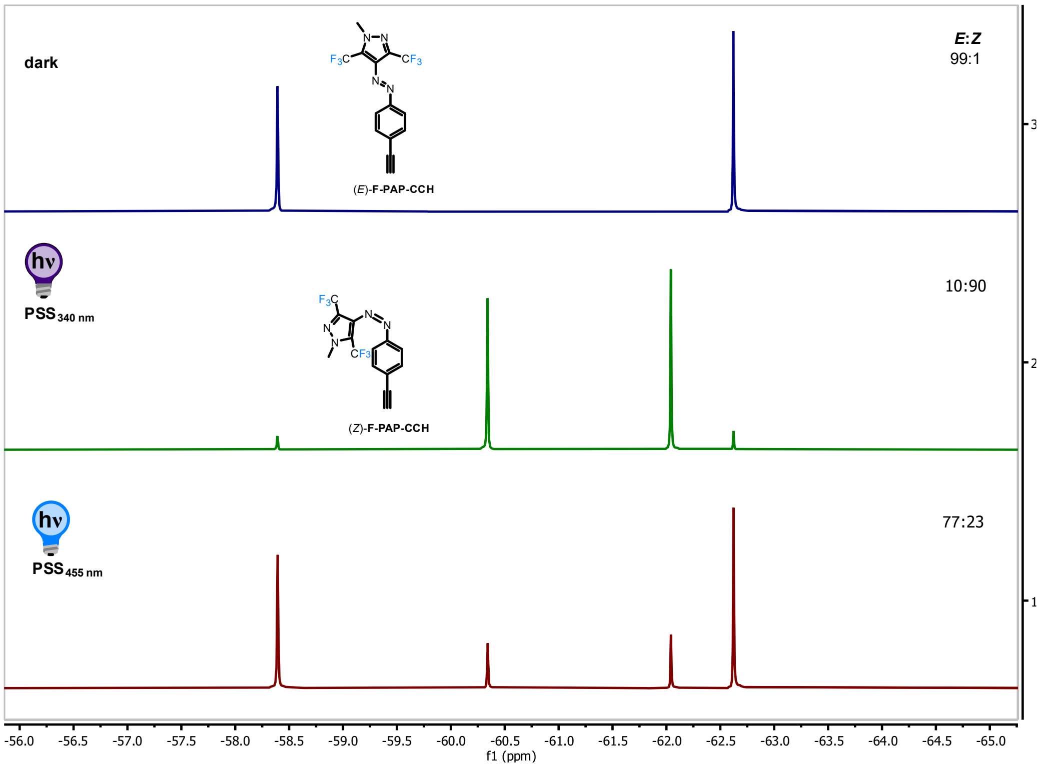

Figure S76: ^{19}F-NMR (376.27 MHz, CD_3CN, 295 K) of **F-PAP-CCH**. The upper trace shows the spectrum before irradiation, the middle trace shows the spectrum at $PSS_{340\ nm}$, and the lower trace shows the spectrum at $PSS_{455\ nm}$.

F-PAP-*n*Bu: (*E*)-4-((4-Butylphenyl)diazenyl)-1-methyl-3,5-bis(trifluoromethyl)-1*H*-pyrazole

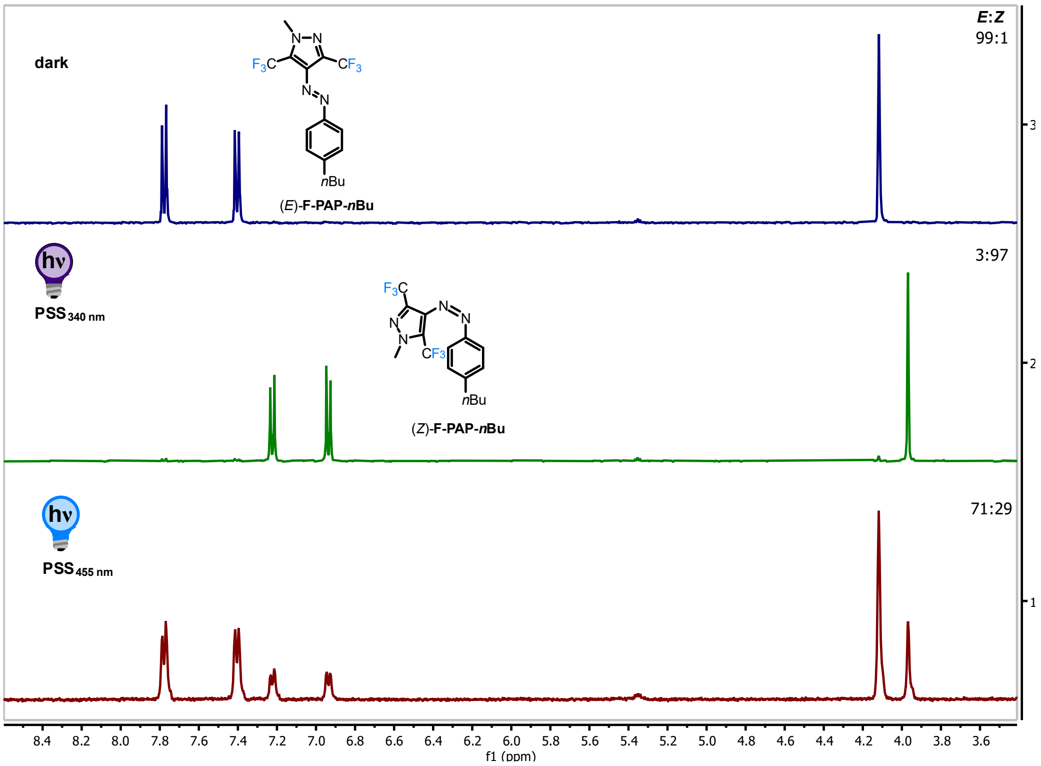

Figure S77: ^{1}H-NMR (399.88 MHz, CD_3CN, 295 K) of **F-PAP-*n*Bu**. The upper trace shows the spectrum before irradiation, the middle trace shows the spectrum at $PSS_{340\ nm}$, and the lower trace shows the spectrum at $PSS_{455\ nm}$.

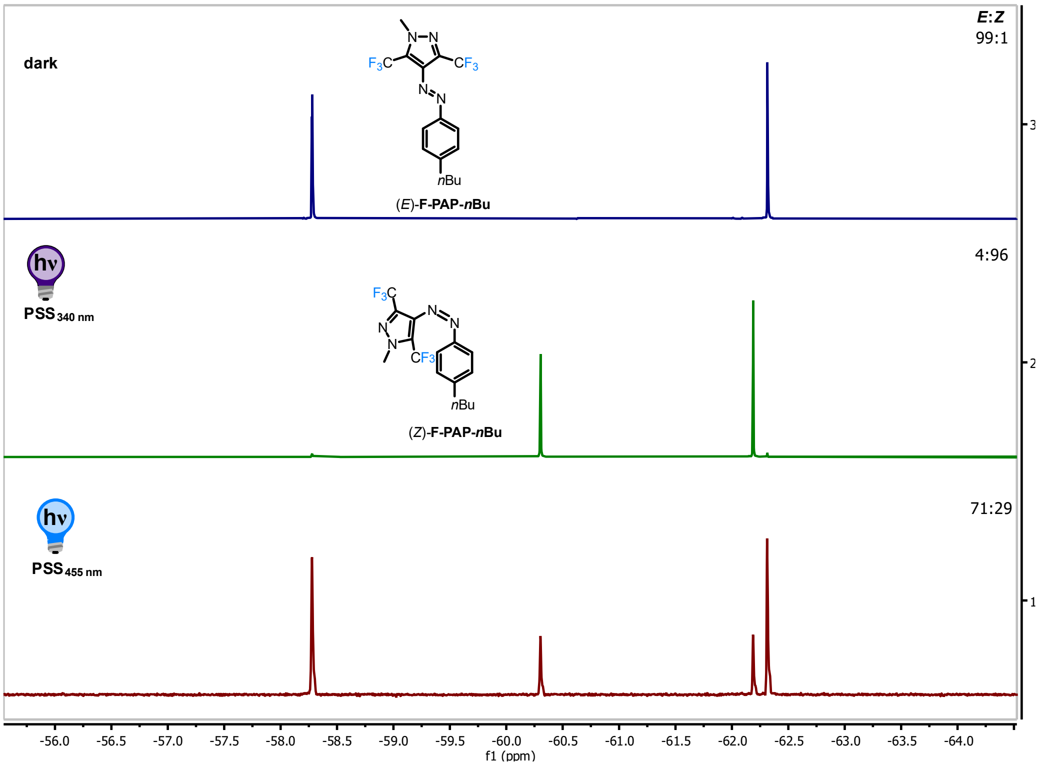

Figure S78: ^{19}F-NMR (376.27 MHz, CD_3CN, 295 K) of **F-PAP-*n*Bu**. The upper trace shows the spectrum before irradiation, the middle trace shows the spectrum at $PSS_{340\ nm}$, and the lower trace shows the spectrum at $PSS_{455\ nm}$.

F-PAP-C_3H_6COOH: (*E*)-4-(4-((1-Methyl-3,5-bis(trifluoromethyl)-1*H*-pyrazol-4-yl)diazenyl)phenyl) butanoic acid

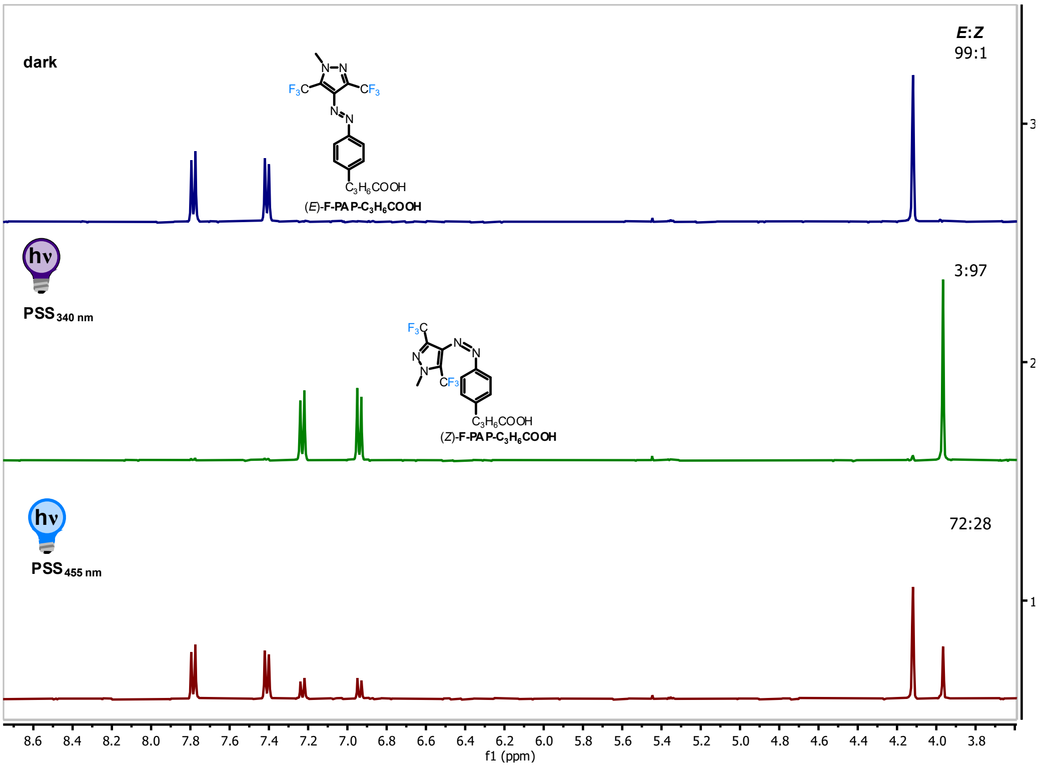

Figure S79: ^{1}H-NMR (399.88 MHz, CD_3CN, 295 K) of **F-PAP-C_3H_6COOH**. The upper trace shows the spectrum before irradiation, the middle trace shows the spectrum at $PSS_{340\ nm}$, and the lower trace shows the spectrum at $PSS_{455\ nm}$.

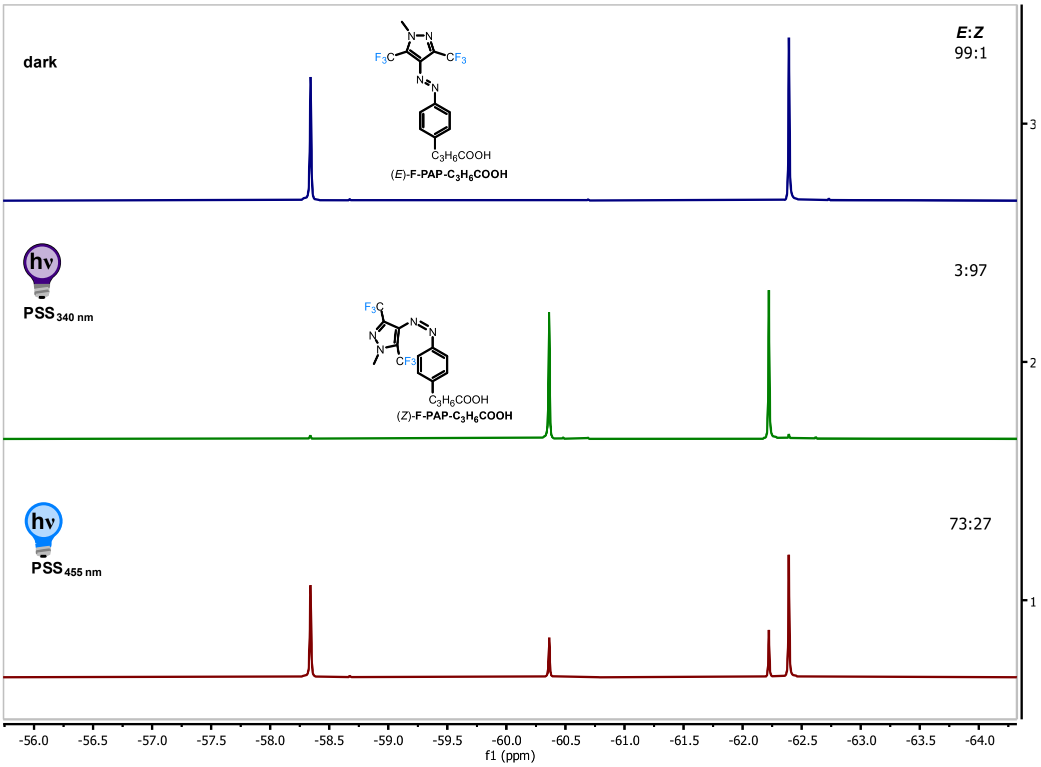

Figure S80: ^{19}F-NMR (376.27 MHz, CD_3CN, 295 K) of **F-PAP- C_3H_6COOH**. The upper trace shows the spectrum before irradiation, the middle trace shows the spectrum at $PSS_{340\ nm}$, and the lower trace shows the spectrum at $PSS_{455\ nm}$.

F-PAP-NO₂: (*E*)-1-Methyl-4-((4-nitrophenyl)diazenyl)-3,5-bis(trifluoromethyl)-1*H*-pyrazole

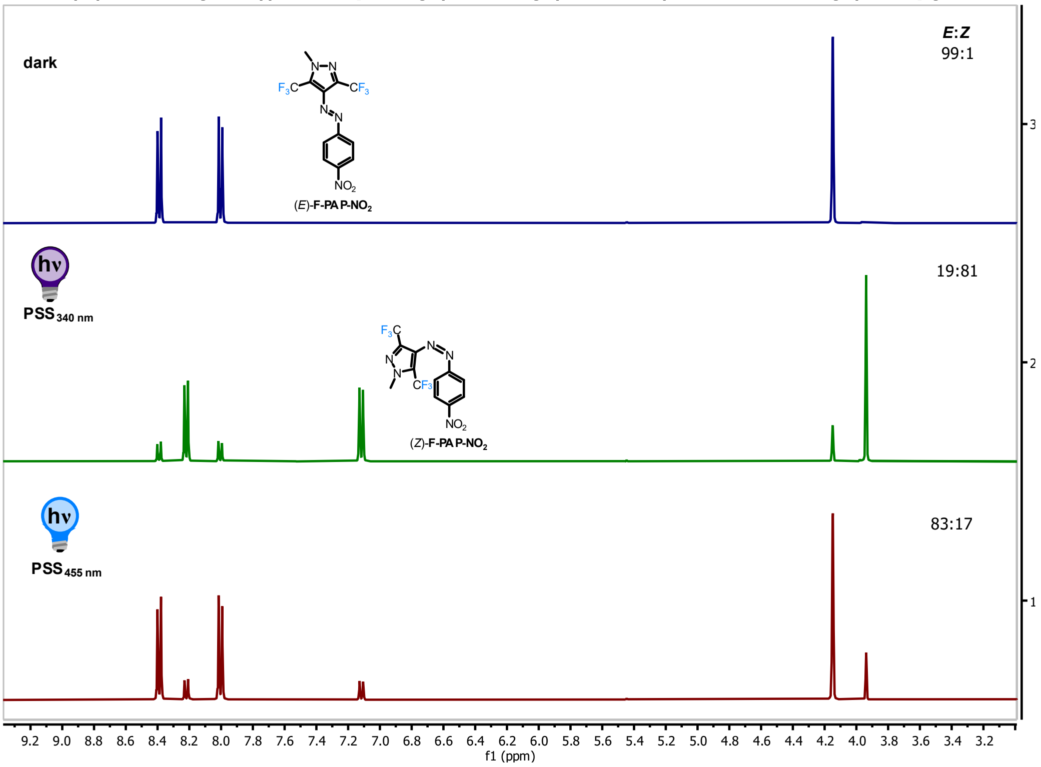

Figure S81: ^{1}H-NMR (399.88 MHz, CD_3CN, 295 K) of **F-PAP-NO₂**. The upper trace shows the spectrum before irradiation, the middle trace shows the spectrum at $PSS_{340\ nm}$, and the lower trace shows the spectrum at $PSS_{455\ nm}$.

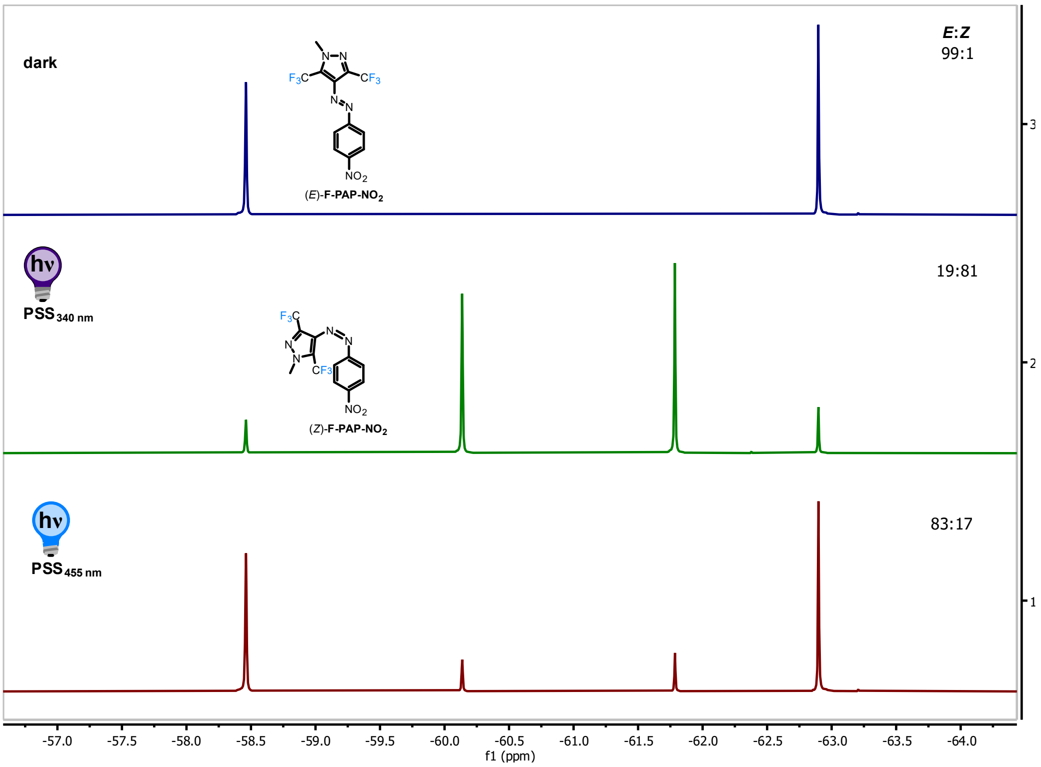

Figure S82: ^{19}F-NMR (376.27 MHz, CD_3CN, 295 K) of **F-PAP-NO₂**. The upper trace shows the spectrum before irradiation, the middle trace shows the spectrum at $PSS_{340\ nm}$, and the lower trace shows the spectrum at $PSS_{455\ nm}$.

F-(*N*Ph)PAP: (*E*)-1-Phenyl-4-(phenyldiazenyl)-3,5-bis(trifluoromethyl)-1*H*-pyrazole

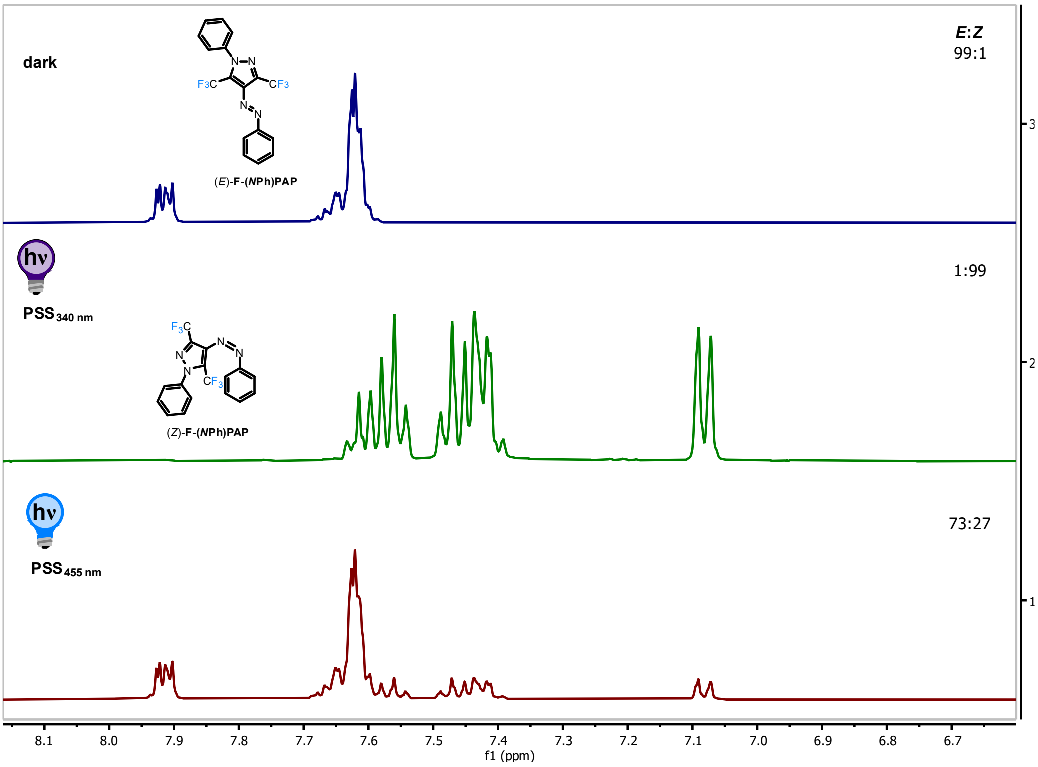

Figure S83: ^{1}H-NMR (399.88 MHz, CD_3CN, 295 K) of **F-(*N*Ph)PAP**. The upper trace shows the spectrum before irradiation, the middle trace shows the spectrum at $PSS_{340\ nm}$, and the lower trace shows the spectrum at $PSS_{455\ nm}$.

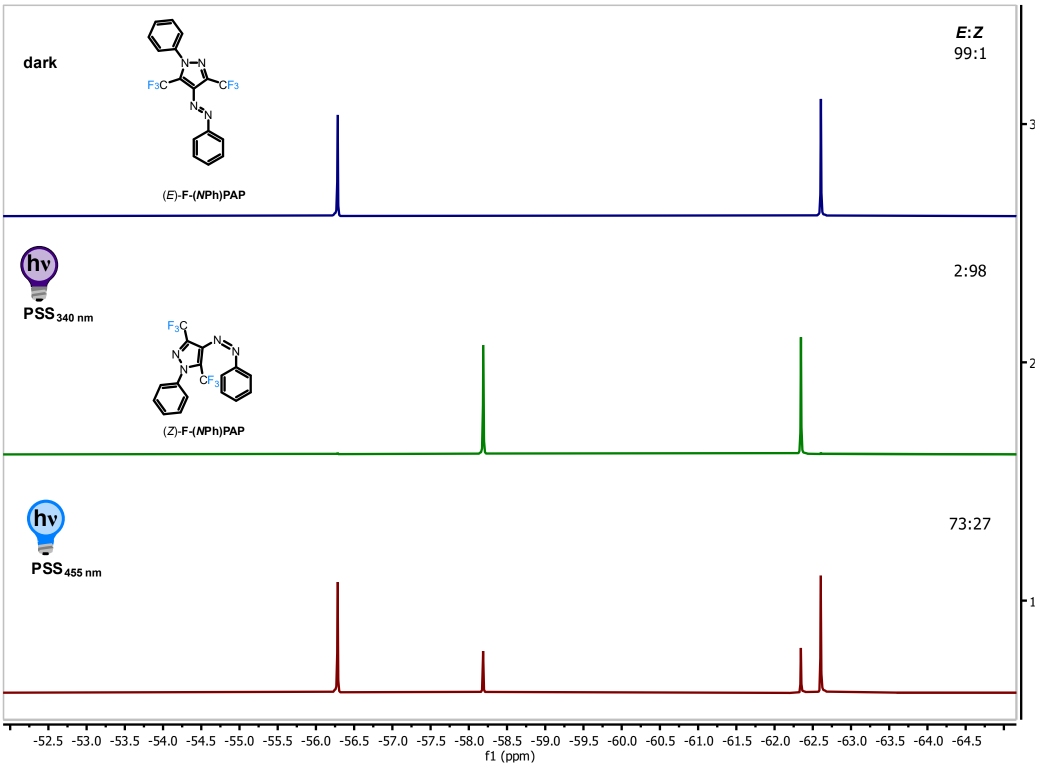

Figure S84: ^{19}F-NMR (376.27 MHz, CD_3CN, 295 K) of **F-(*N*Ph)PAP**. The upper trace shows the spectrum before irradiation, the middle trace shows the spectrum at $PSS_{340\ nm}$, and the lower trace shows the spectrum at $PSS_{455\ nm}$.

3.7. Thermal *Z*-*E* relaxation

Samples were dissolved in toluene and diluted to absorbance 1.2-1.4 (conc. 60 -70 μM). For Me, *n*Bu, Acid and OMe PAPs 0.6-0.7 (conc. 30 -35 μM) was used. Samples were irradiated with 365 nm LED while heated up on 80 °C. The relaxation kinetic was measured via an Avantes UV-Vis spectrometer.

The absorbance dependency on time was plotted and fitted with an exponential decay fit (equation $y = A_1 * e^{(-x/\tau)} + y_0$), where τ is the lifetime.

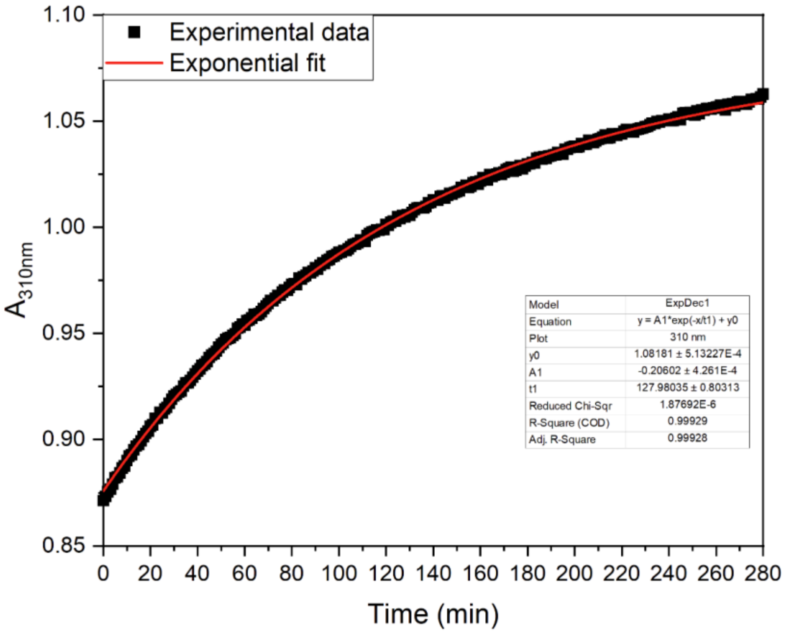

Figure S85: Thermal *Z*-*E* isomerization of **F-PAP-H** at 80 °C in toluene.

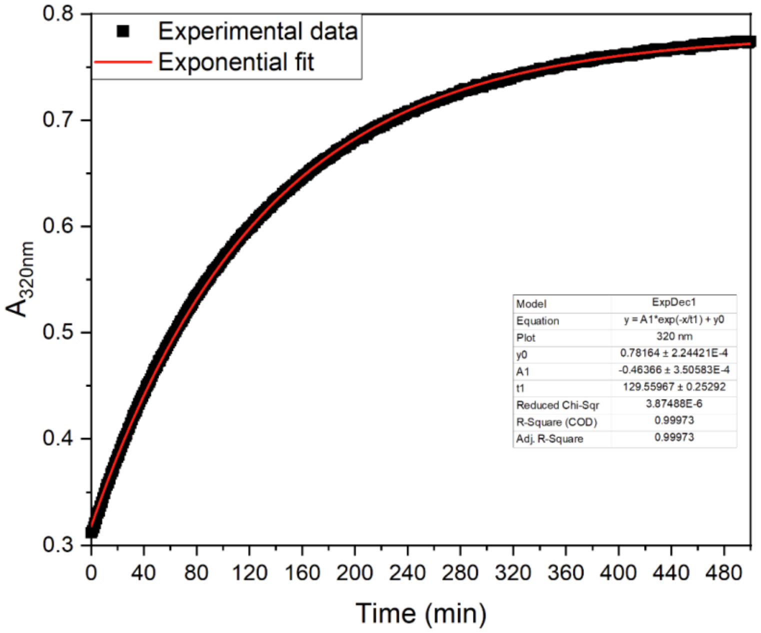

Figure S86: Thermal *Z-E* isomerization of **F-PAP-Cl** at 80 °C in toluene.

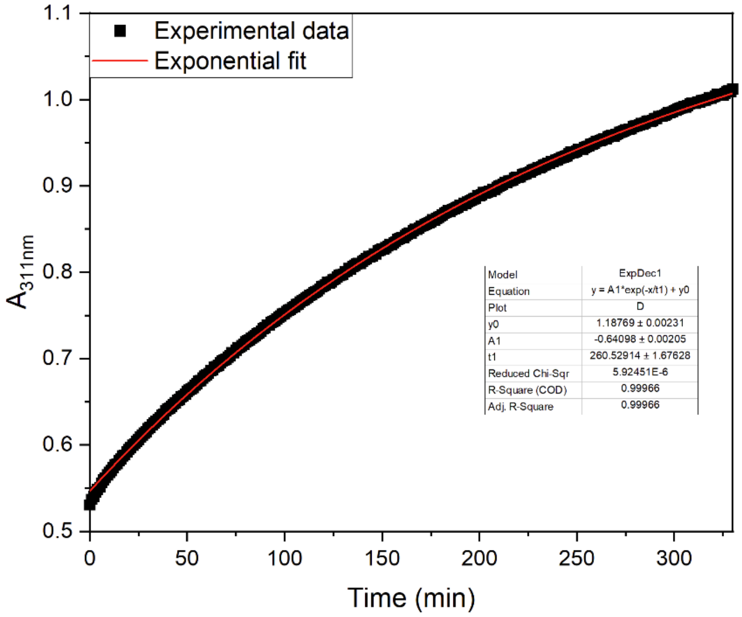

Figure S87: Thermal *Z-E* isomerization of **F-PAP- CF_3** at 80 °C in toluene.

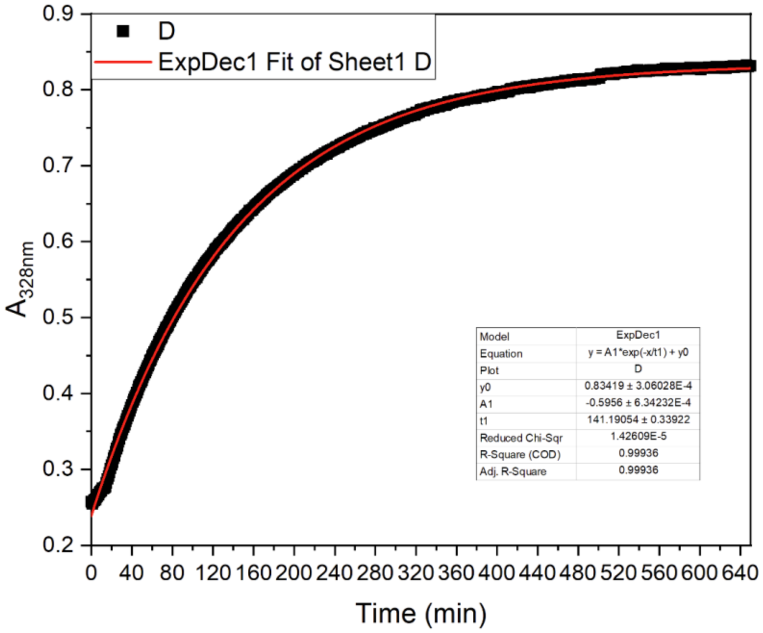

Figure S88: Thermal *Z*-*E* isomerization of **F-PAP-Br** at 80 °C in toluene.

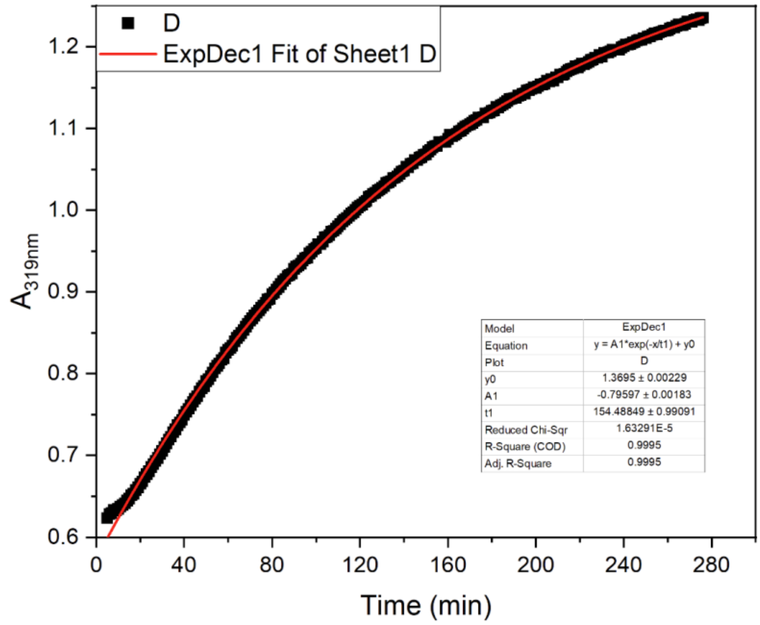

Figure S89: Thermal *Z*-*E* isomerization of **F-PAP-CN** at 80 °C in toluene.

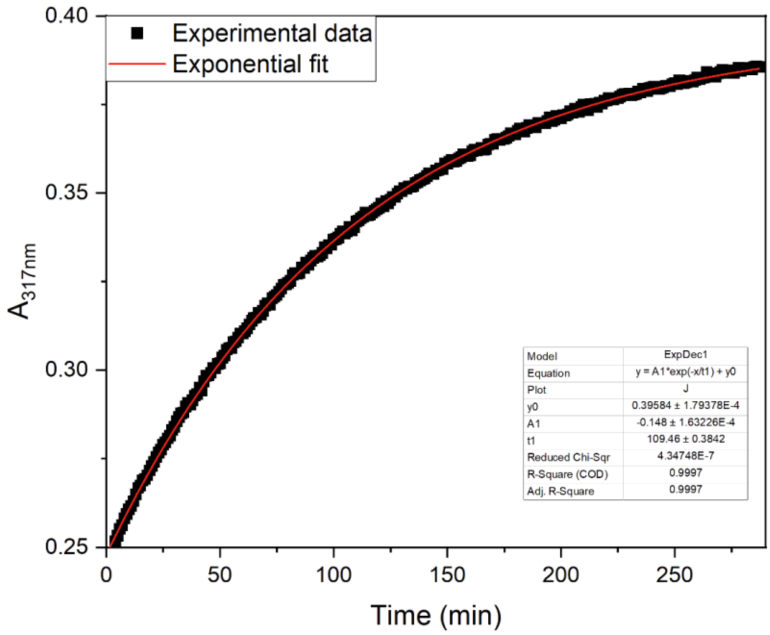

Figure S90: Thermal *Z*-*E* isomerization of **F-PAP-Me** at 80 °C in toluene.

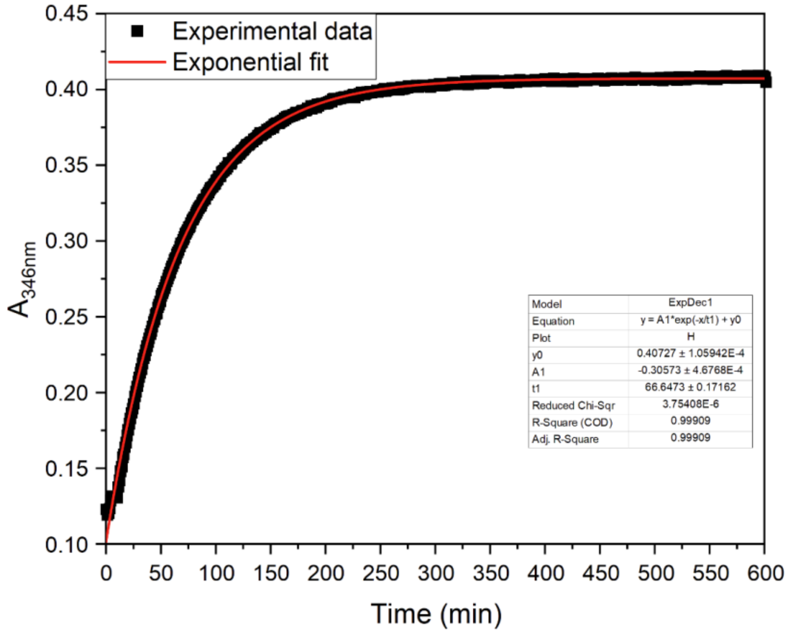

Figure S91: Thermal *Z*-*E* isomerization of **F-PAP-OMe** at 80 °C in toluene.

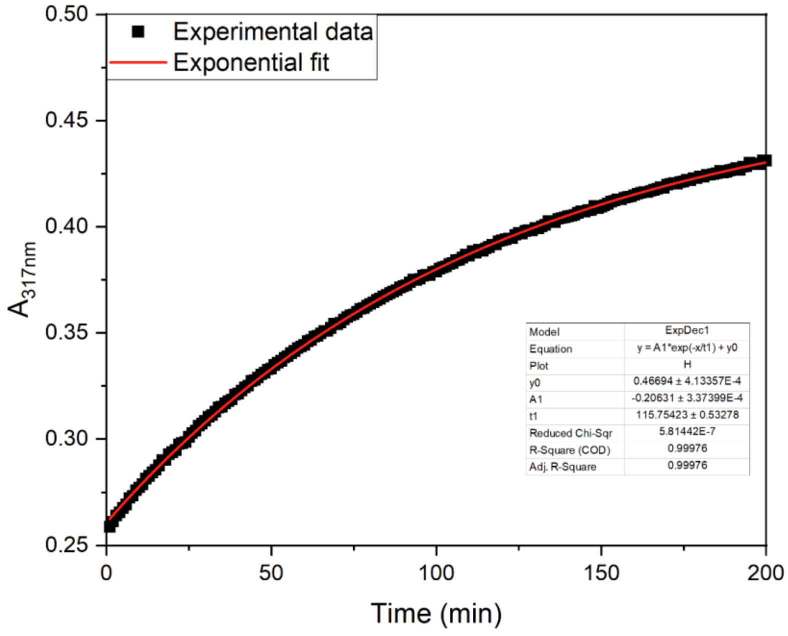

Figure S92: Thermal *Z*-*E* isomerization of **F-PAP-C_3H_6COOH** at 80 °C in toluene.

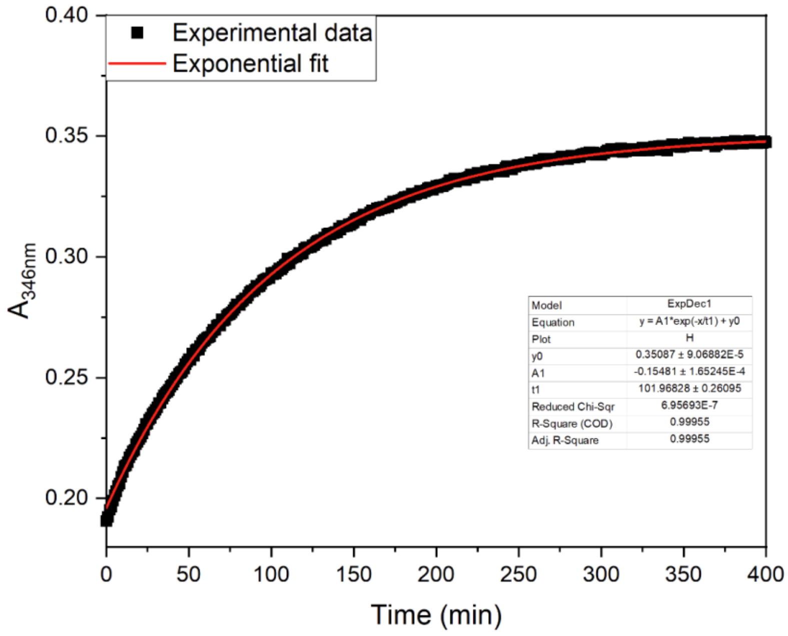

Figure S93: Thermal *Z-E* isomerization of **F-PAP-*n*Bu** at 80 °C in toluene.

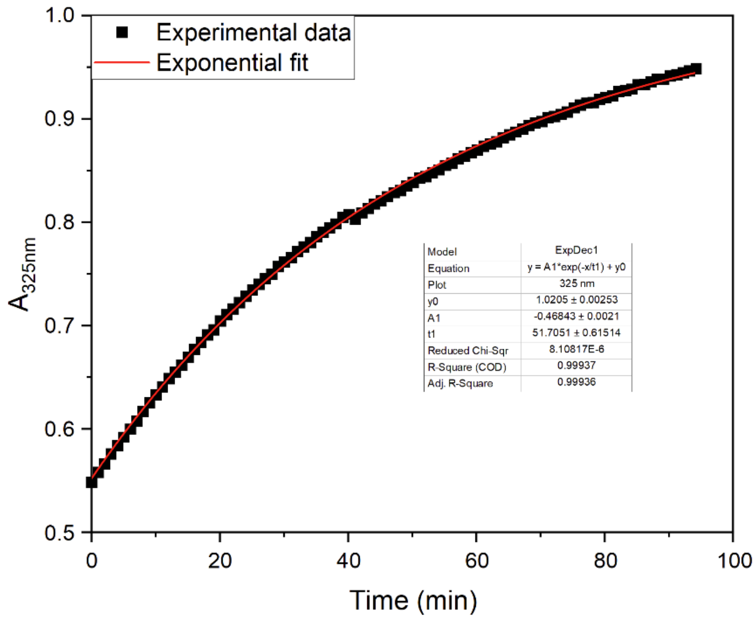

Figure S94: Thermal *Z-E* isomerization of **F-PAP-CCH** at 80 °C in toluene.

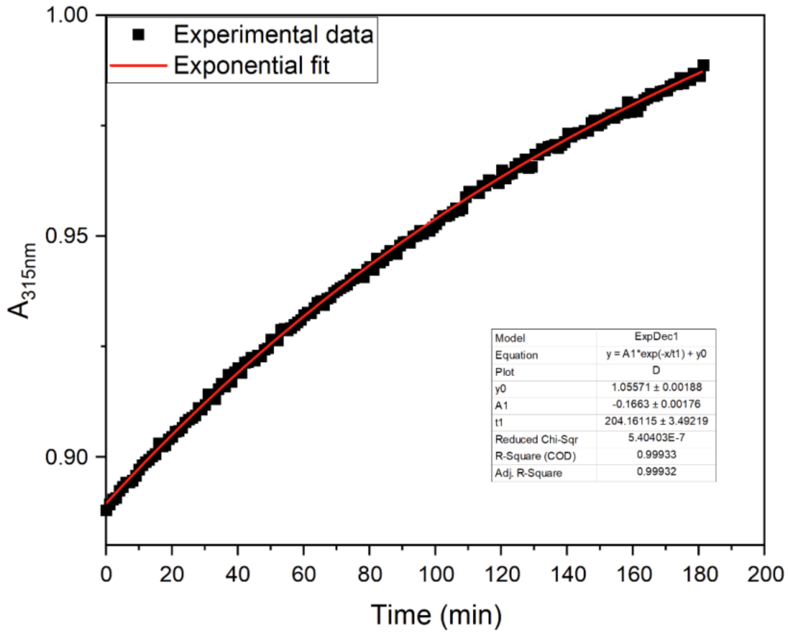

Figure S95: Thermal *Z*-*E* isomerization of **F-PAP-NO_2** at 80 °C in toluene.

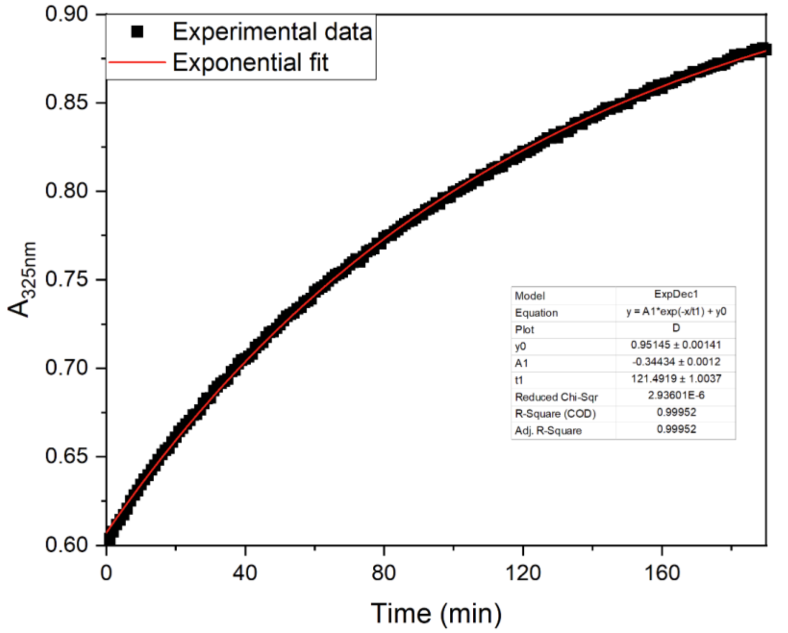

Figure S96: Thermal *Z*-*E* isomerization of **F(*N*Ph)-PAP** at 80 °C in toluene.

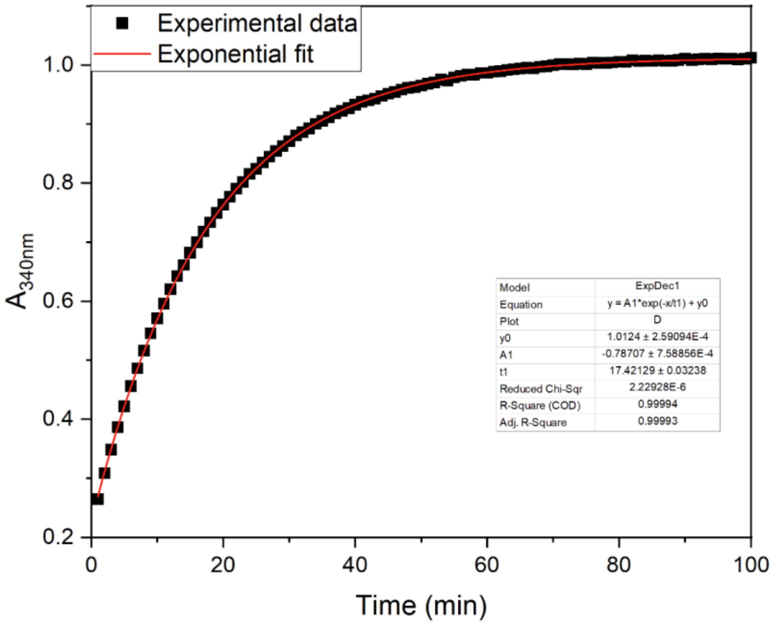

Figure S97: Thermal *Z*-*E* isomerization of **PAP-H** at 80 °C in toluene.

4. Hammett Substituent Parameter Analysis

4.1. λ_{max} Analysis of **F-PAPs**

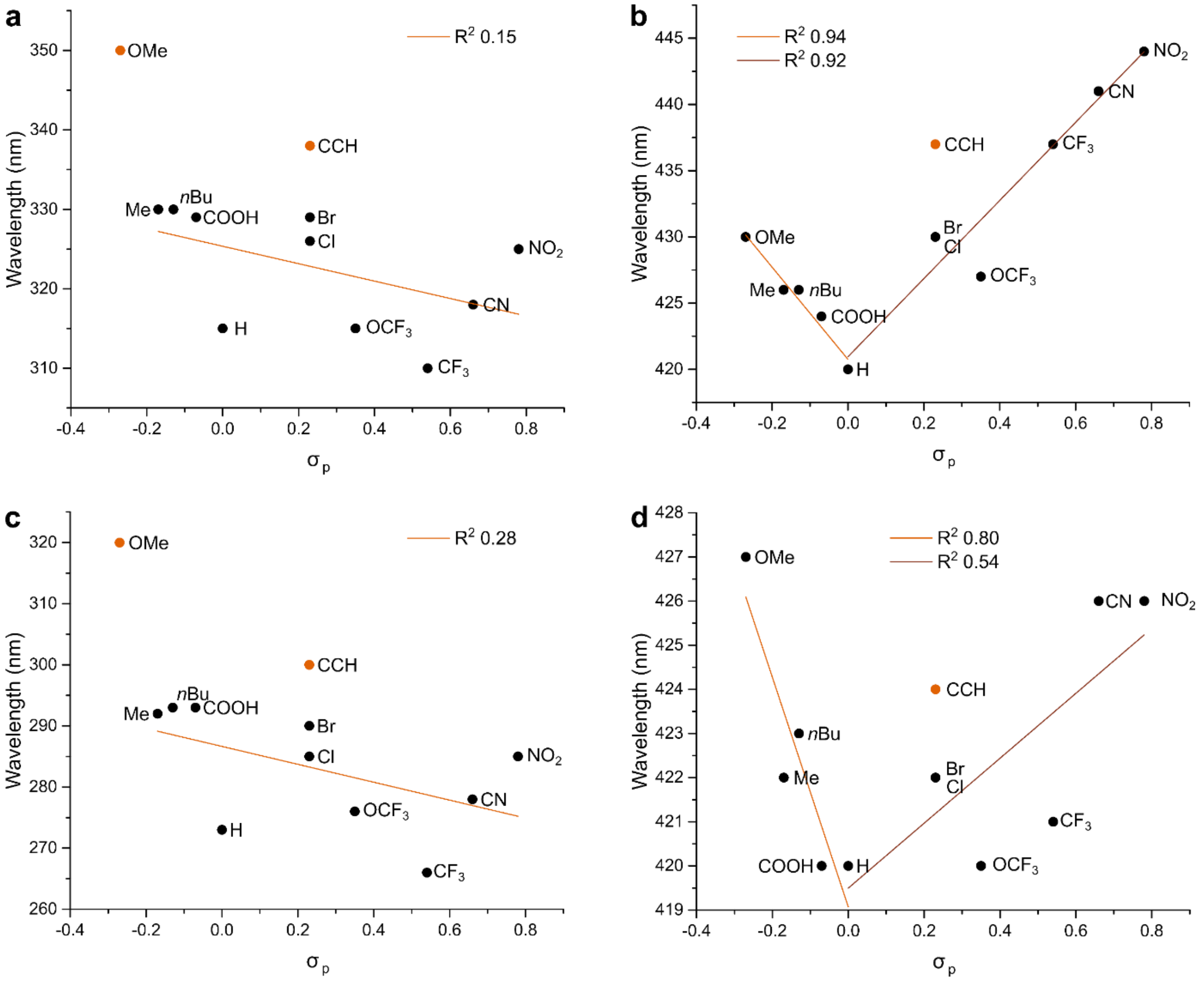

Figure S98: a Shows the λ_{max} of the ππ* band of the *E* isomer of **F-PAPs** as a correlation to the Hammett parameter, where the outliers are marked in orange and the correlation is shown in orange. **b** Shows the λ_{max} of the nπ* band of the *E* isomer of **F-PAPs** as a correlation to the Hammett parameter, where the outliers are marked in orange and the correlations are shown in orange and maroon. **c** Shows the λ_{max} of the ππ* band of the *Z* isomer of **F-PAPs** as a correlation to the Hammett parameter, where the outliers are marked in orange and the correlation is shown in orange. **d** Shows the λ_{max} of the nπ* band of the *Z* isomer of **F-PAPs** as a correlation to the Hammett parameter, where the outliers are marked in orange and the correlations are shown in orange and maroon.

4.2. Photostationary State Distribution

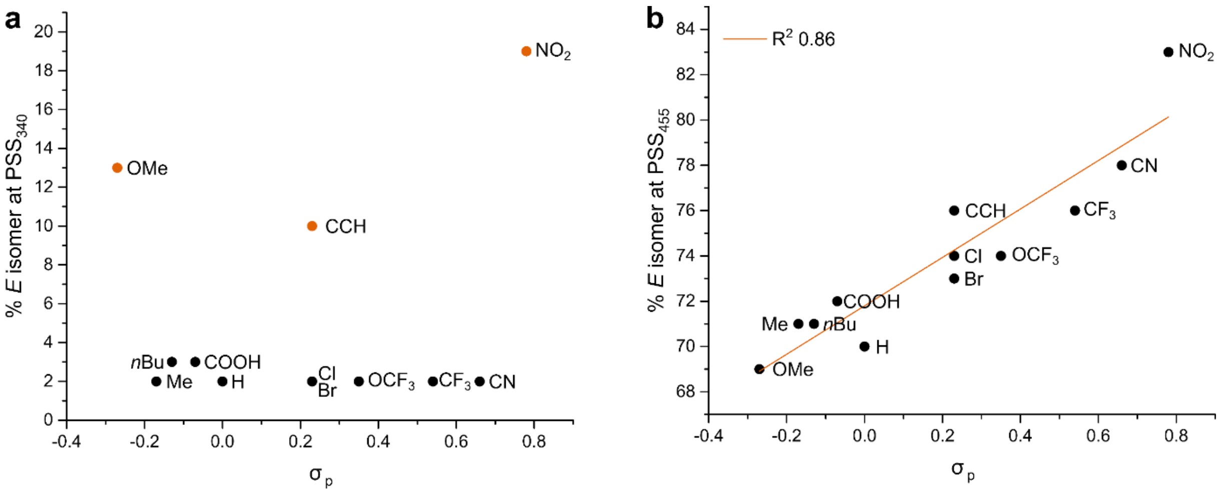

Figure S99: a Shows the percentage of *E* isomer of **F-PAP**s at the $PSS_{340\ nm}$ in correlation to the Hammett parameter (σ_p), where the outliers are marked in orange. **b** Shows the percentage of *E* isomer of **F-PAP**s at the $PSS_{455\ nm}$, with the correlation shown in orange.

4.3. Quantum Yields

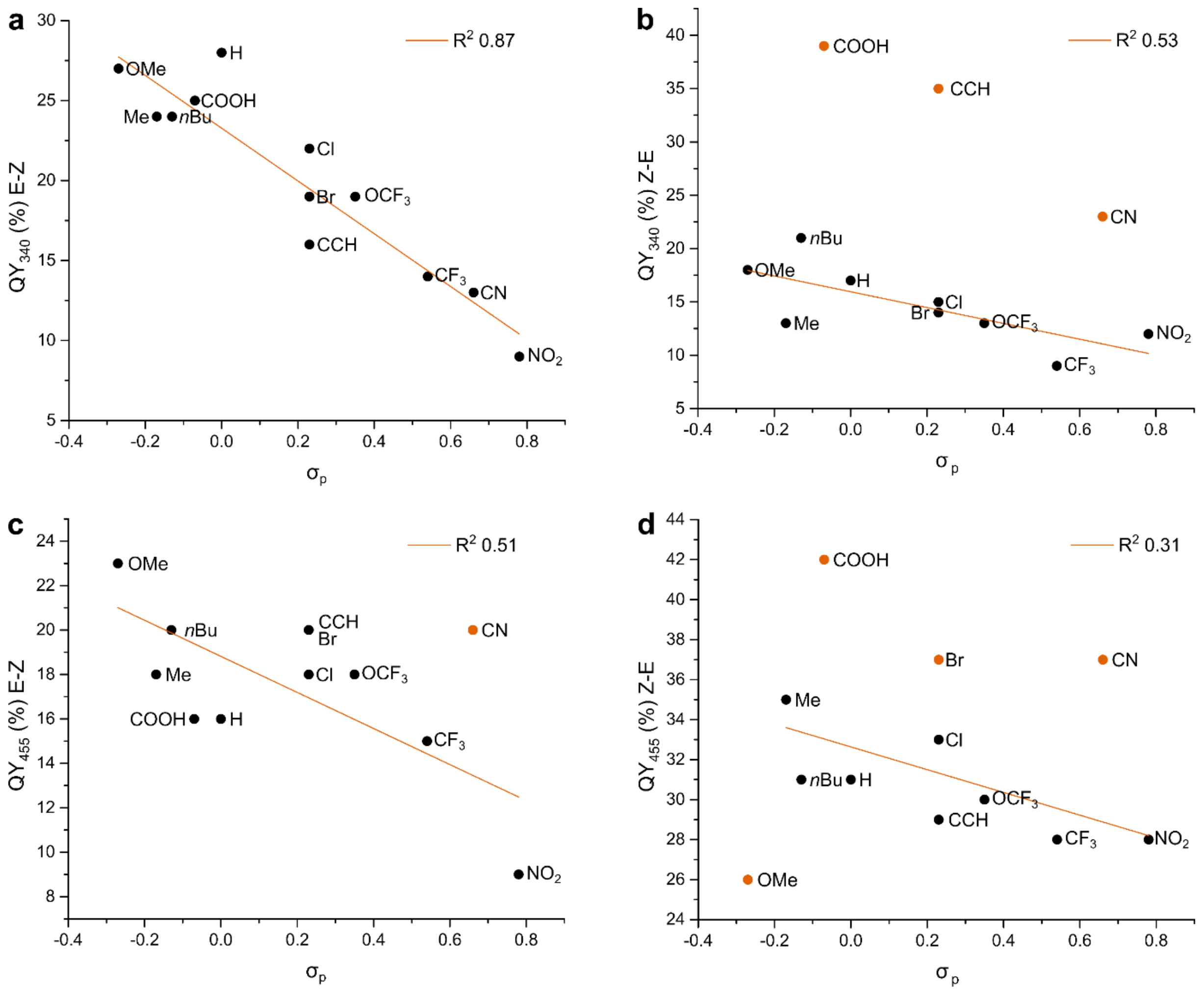

Figure S100: a Shows the percentage of quantum yield$_{340\ nm}$ *E*→*Z* of **F-PAPs** in correlation to the Hammett parameter (σ_p), with the correlation shown in orange. **b** Shows the percentage of quantum yield$_{340\ nm}$ Z→E of **F-PAPs** in correlation to the Hammett parameter (σ_p), where the outliers are marked in orange and the correlation is shown in orange. **c** Shows the percentage of quantum yield$_{455\ nm}$ *E*→*Z* of **F-PAPs** in correlation to the Hammett parameter (σ_p), where the outliers are marked in orange and the correlation is shown in orange. **d** Shows the percentage of quantum yield$_{455\ nm}$ *Z*→*E* of **F-PAPs** in correlation to the Hammett parameter (σ_p), where the outliers are marked in orange and the correlation is shown in orange.

4.4. Lifetimes

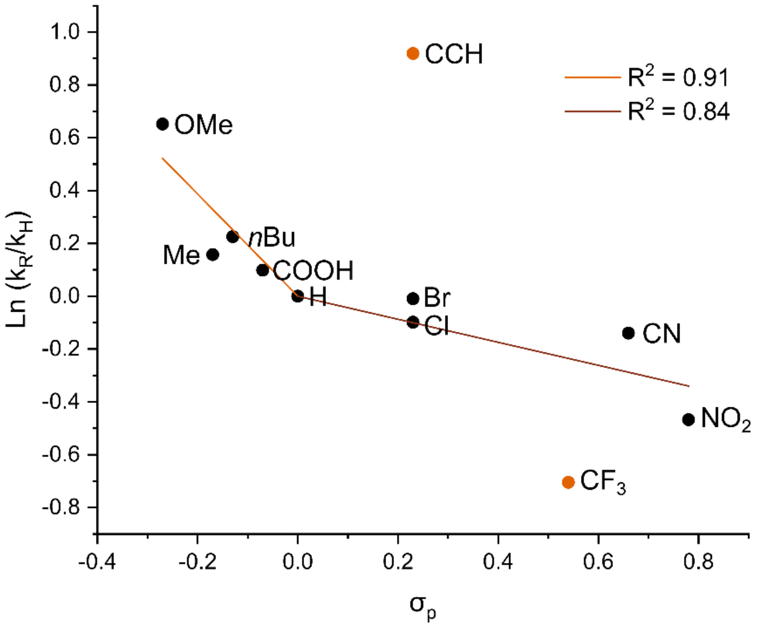

Figure S101: Shows the lifetimes of **F-PAP**s in correlation to the Hammett parameter (σ_p), with the correlations shown in orange and maroon. We observe a slight change in slope, hinting at a different mechanism, where outliers are marked in orange.

5. Vesicle Formation and Manipulation

Stock solutions (4 mg/mL in chloroform) of F-(*Nn*PrCOOH)PAP-*n*Bu, DOPC, and cholesterol were prepared. Aliquots of each (150 µL, 325 µL, and 25 µL, respectively) were combined in a 5 mL glass test tube. The solvent was evaporated under a gentle stream of nitrogen, yielding a thin lipid film on the tube wall. This film was further dried under vacuum at 50 °C for 1 hour to remove residual solvent. Hydration was initiated by adding 0.5 mL of D_2O to the dried film. After a 10-minute incubation period at room temperature, the suspension was vortexed to aid dispersion. This hydration–vortex cycle was repeated twice to enhance vesicle formation. Subsequently, 10 µL of a saturated $NaHCO_3$ solution in D_2O was added to facilitate the detachment of the film from the glass surface. The resulting suspension was transferred to an NMR tube and used directly for subsequent experiments.

The sample was irradiated using a 340 nm LED light source (see *General Information* for specifications) in successive intervals: initially for 5 minutes, followed by additional irradiation periods of 5, 10, and 60 minutes (cumulative irradiation times: 5, 10, 20, and 80 minutes). After irradiation, both ^{1}H NMR (128 scans) and ^{19}F NMR (512 scans) spectra were recorded to monitor compositional changes.

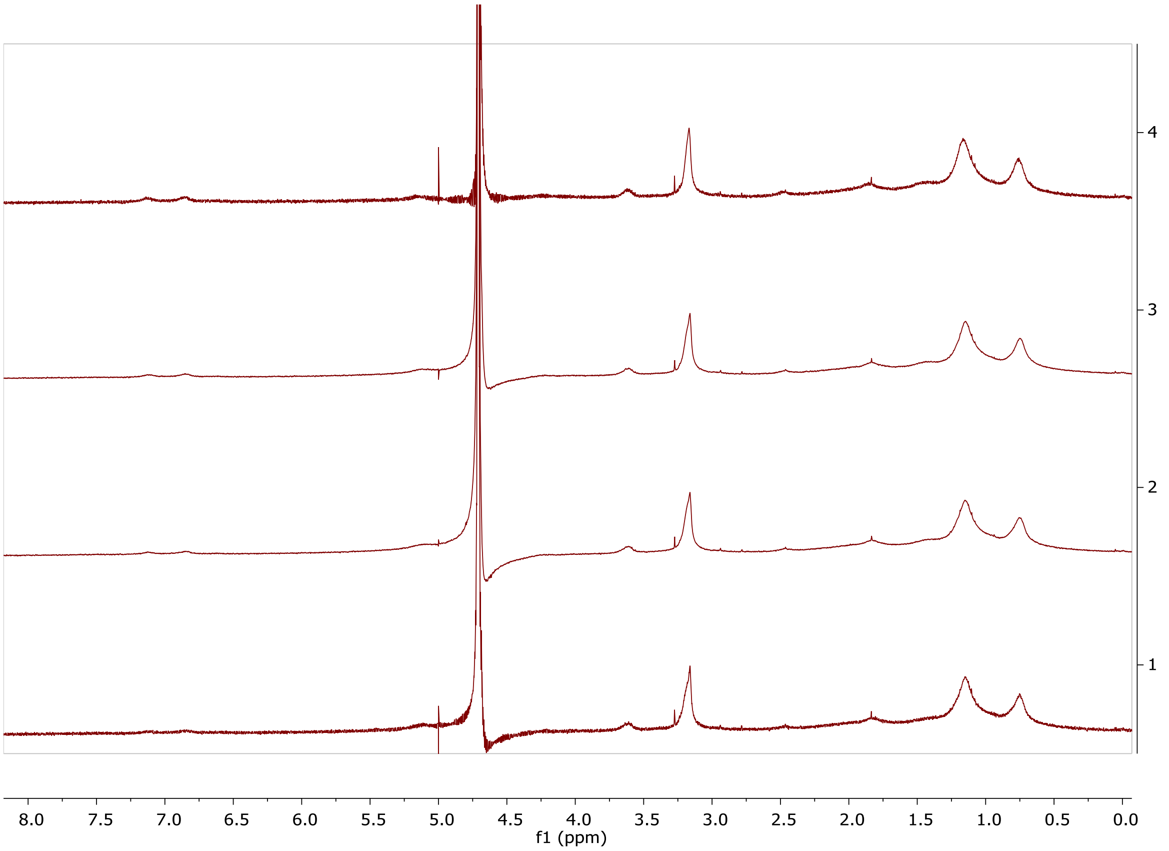

Figure S102: ^{1}H-NMR (300 MHz, D_2O, 295 K) of **11** in vesicles after 5, 10, 20, and 80 min (bottom to top) irradiation with 340 nm *ex situ* LED light.

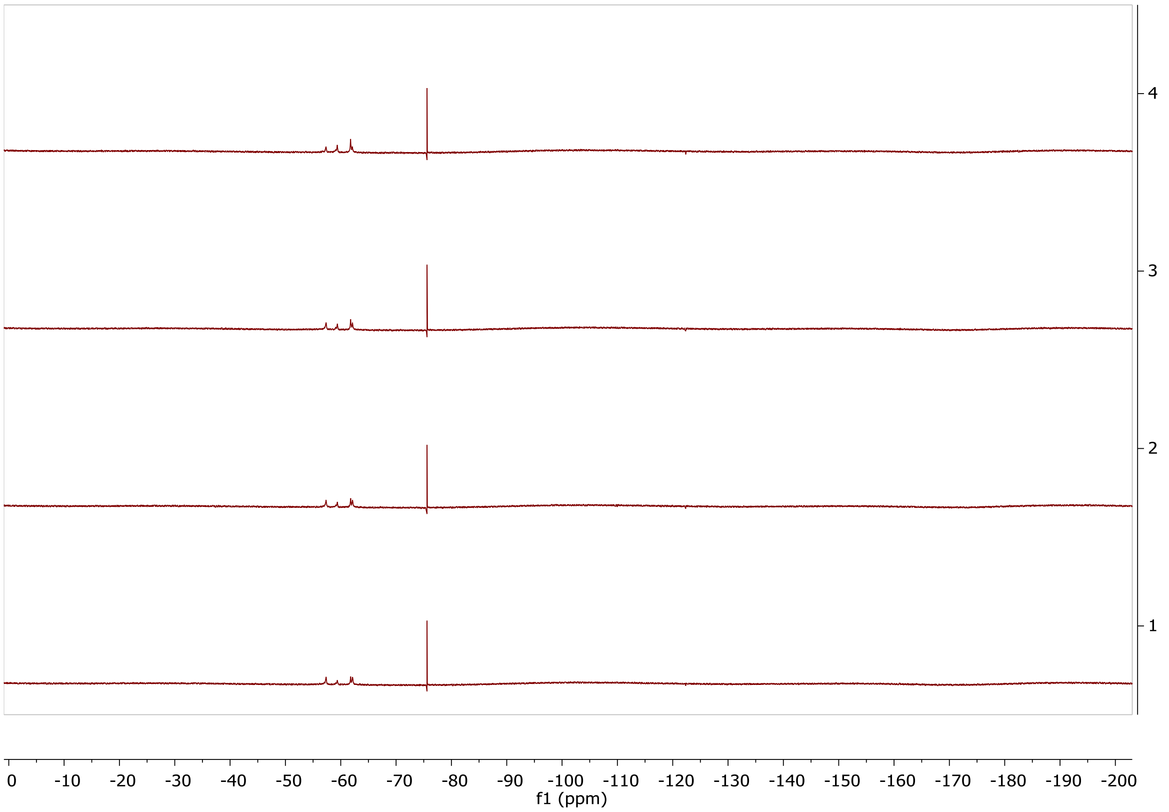

Figure S103: ^{19}F-NMR (282.37 MHz, D_2O, 295 K) of **11** in vesicles after 5, 10, 20, and 80 min (top to bottom) irradiation with 340 nm *ex situ* LED light.

6. NMR Spectra

F-PAP-H: (*E*)-1-Methyl-4-(phenyldiazenyl)-3,5-bis(trifluoromethyl)-1*H*-pyrazole

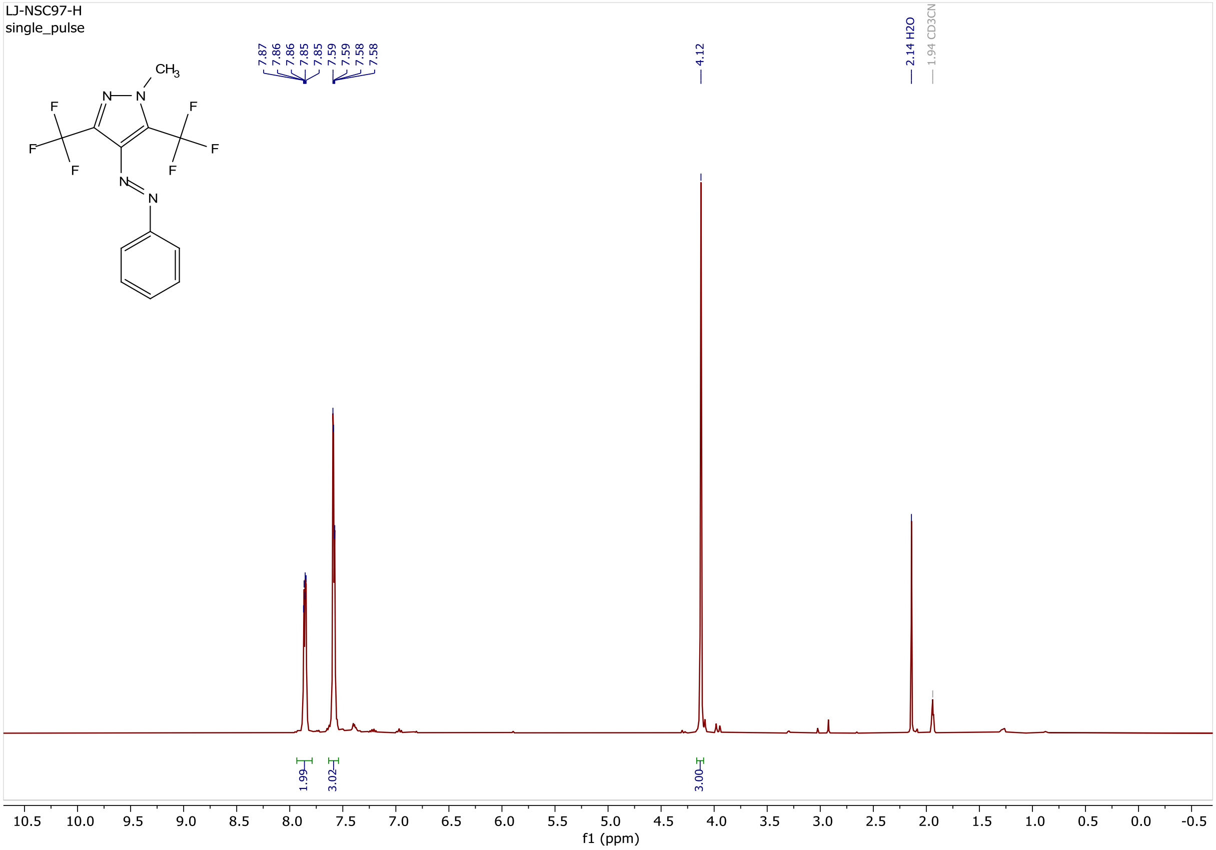

Figure S104: ^{1}H-NMR (399.78 MHz, CD_3CN, 295 K) of **F-PAP-H**.

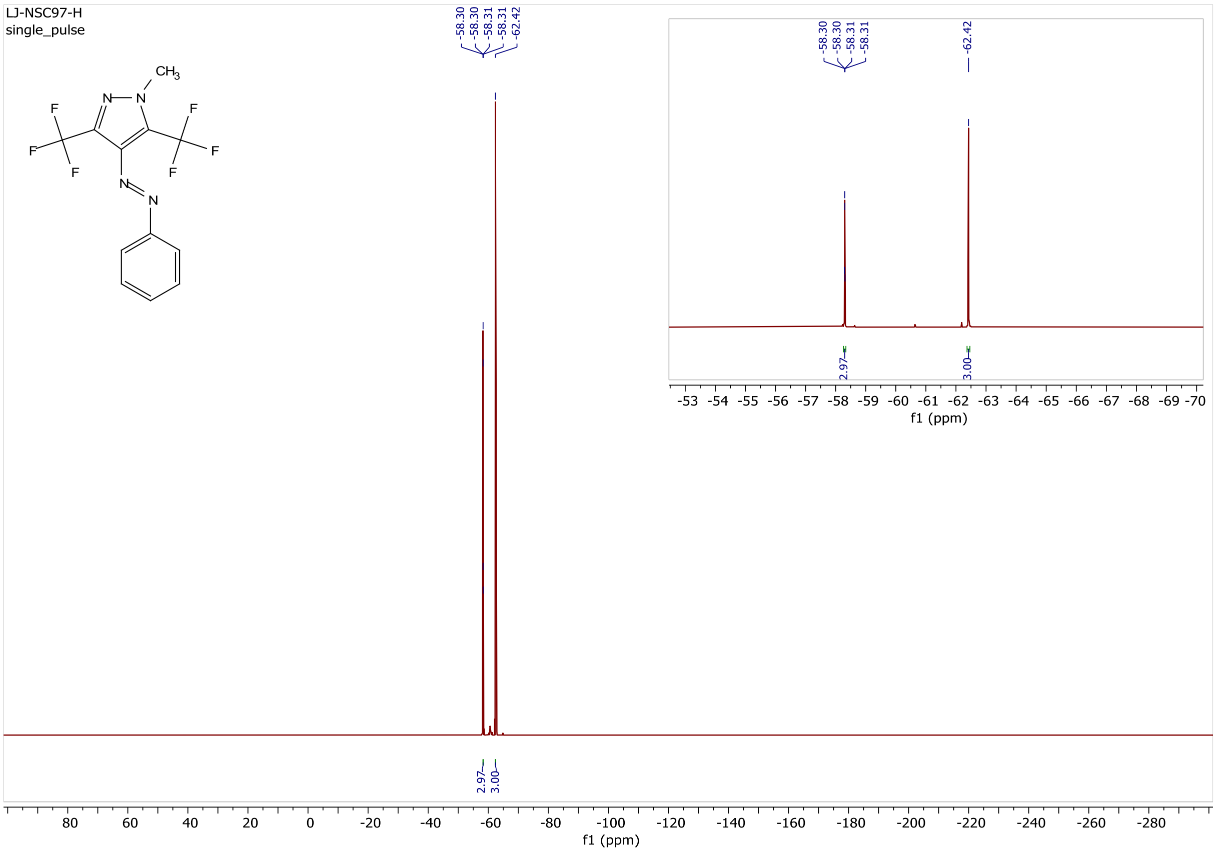

Figure S105: ^{19}F-NMR (376.13 MHz, CD_3CN, 295 K) of **F-PAP-H**.

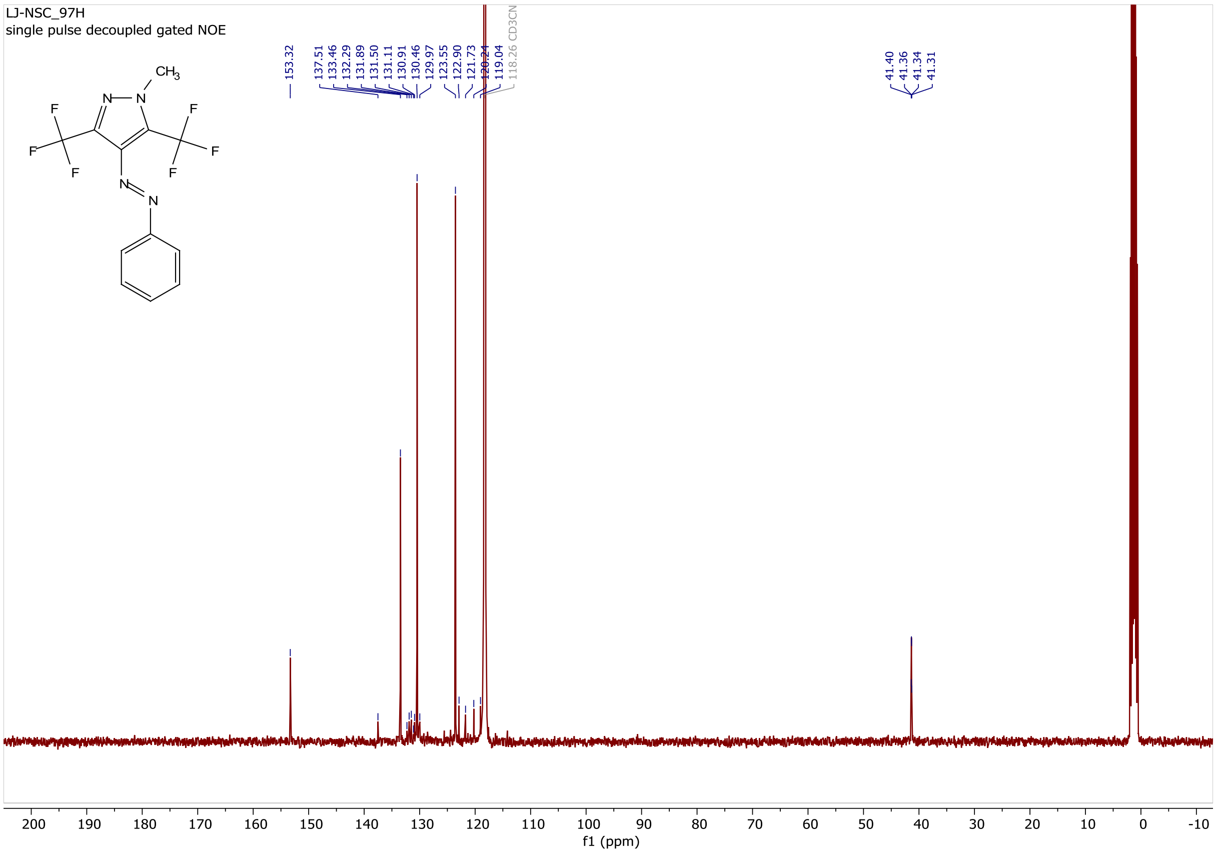

Figure S106: ^{13}C-NMR {^{1}H} (100.54 MHz, CD_3CN, 295 K) of **F-PAP-H**.

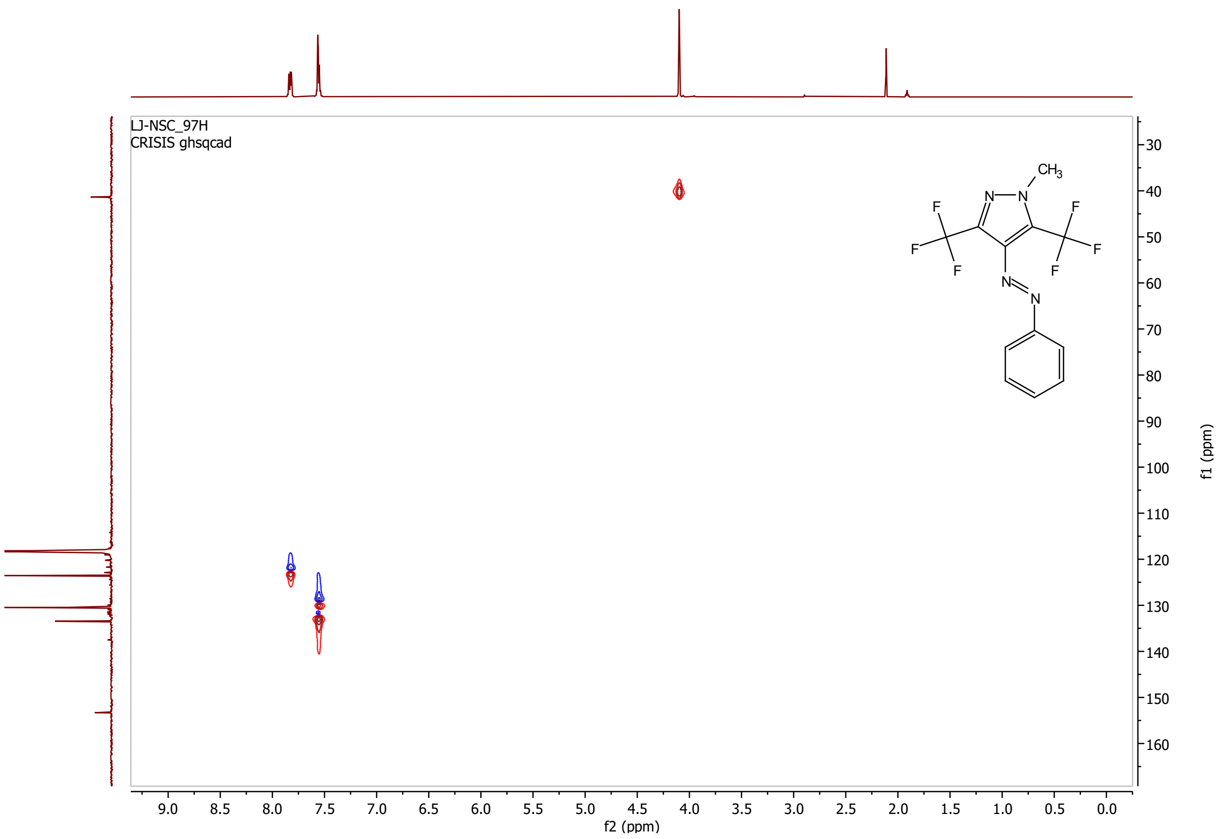

Figure S107: HSQC [$^1H \leftrightarrow ^{13}C$, (399.78 MHz /100.54 MHz, CD_3CN, 295 K) of **F-PAP-H**.

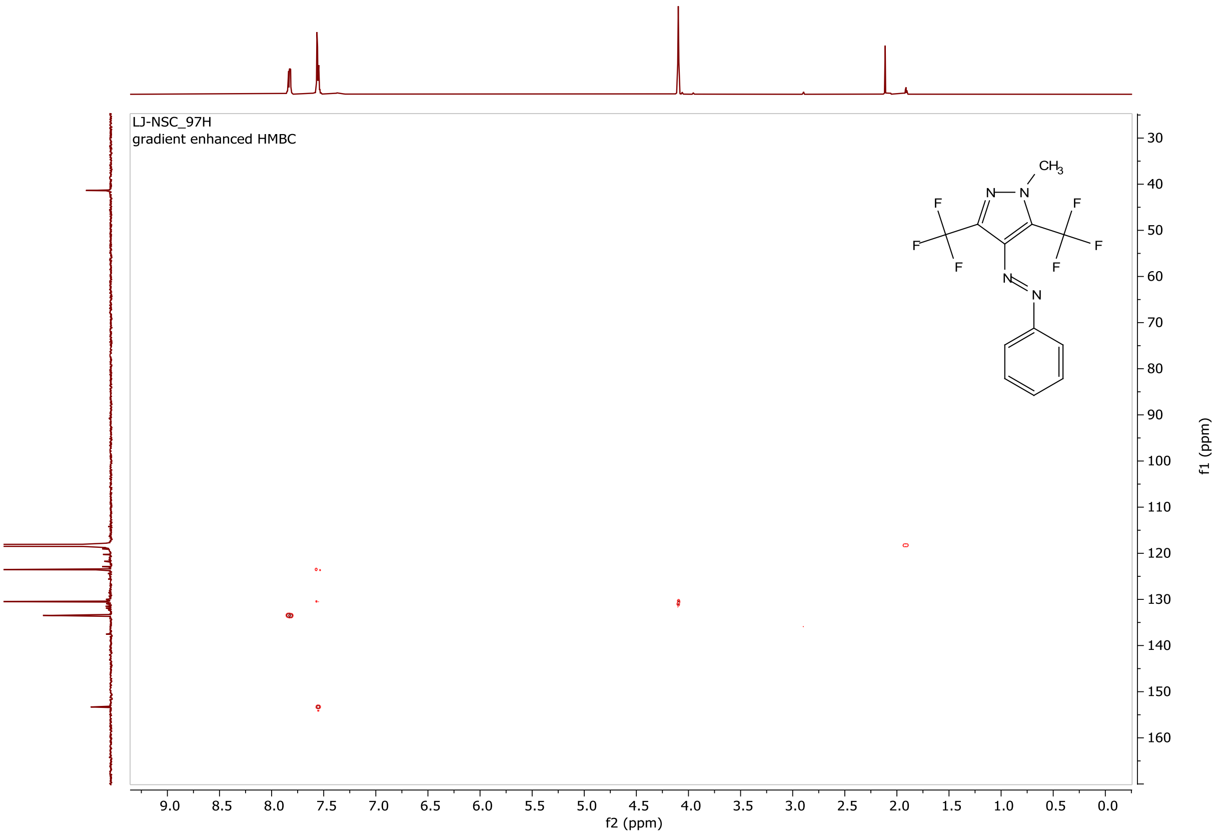

Figure S108: HMBC [$^1H \leftrightarrow ^{13}C$, (399.78 MHz /100.54 MHz, CD_3CN, 295 K) of **F-PAP-H**.

F-PAP-Cl: (*E*)-4-((4-Chlorophenyl)diazenyl)-1-methyl-3,5-bis(trifluoromethyl)-1*H*-pyrazole

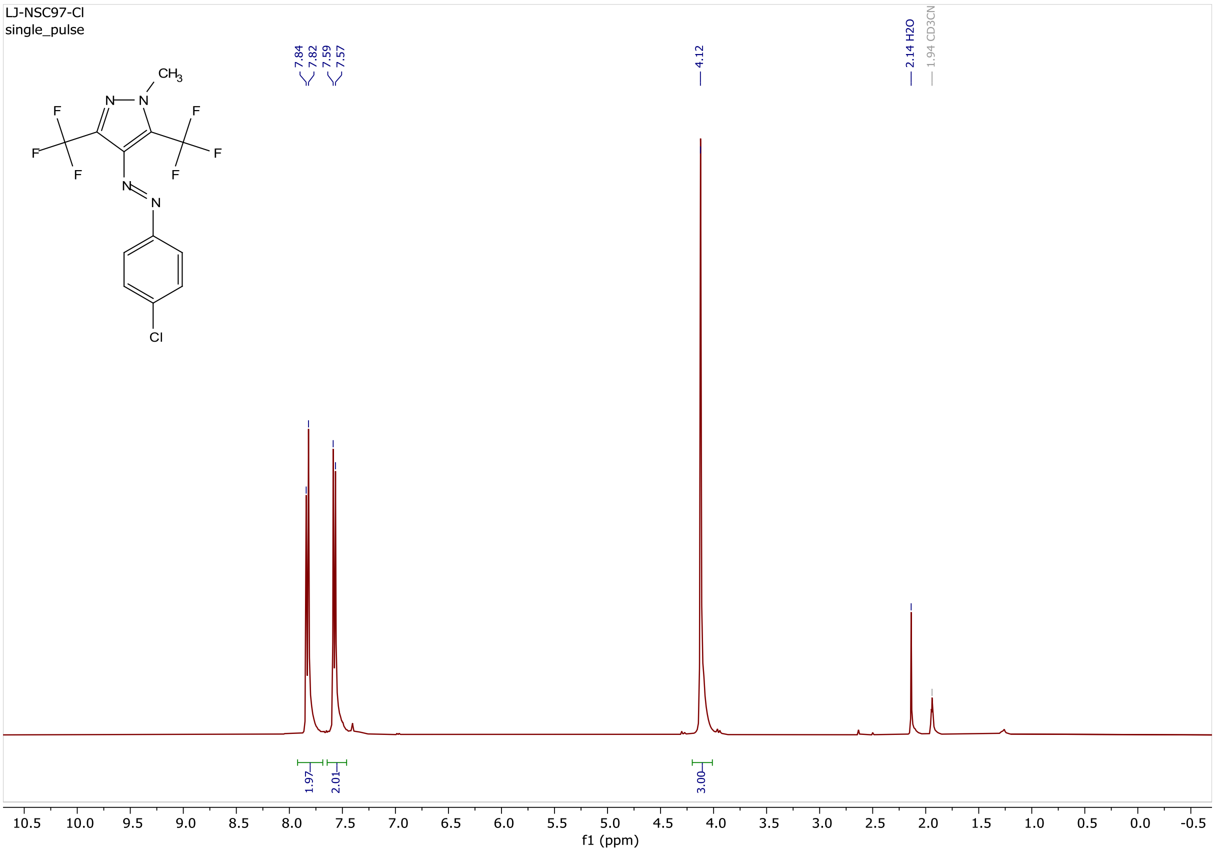

Figure S109: ^{1}H-NMR (399.78 MHz, CD_3CN, 295 K) of **F-PAP-Cl**.

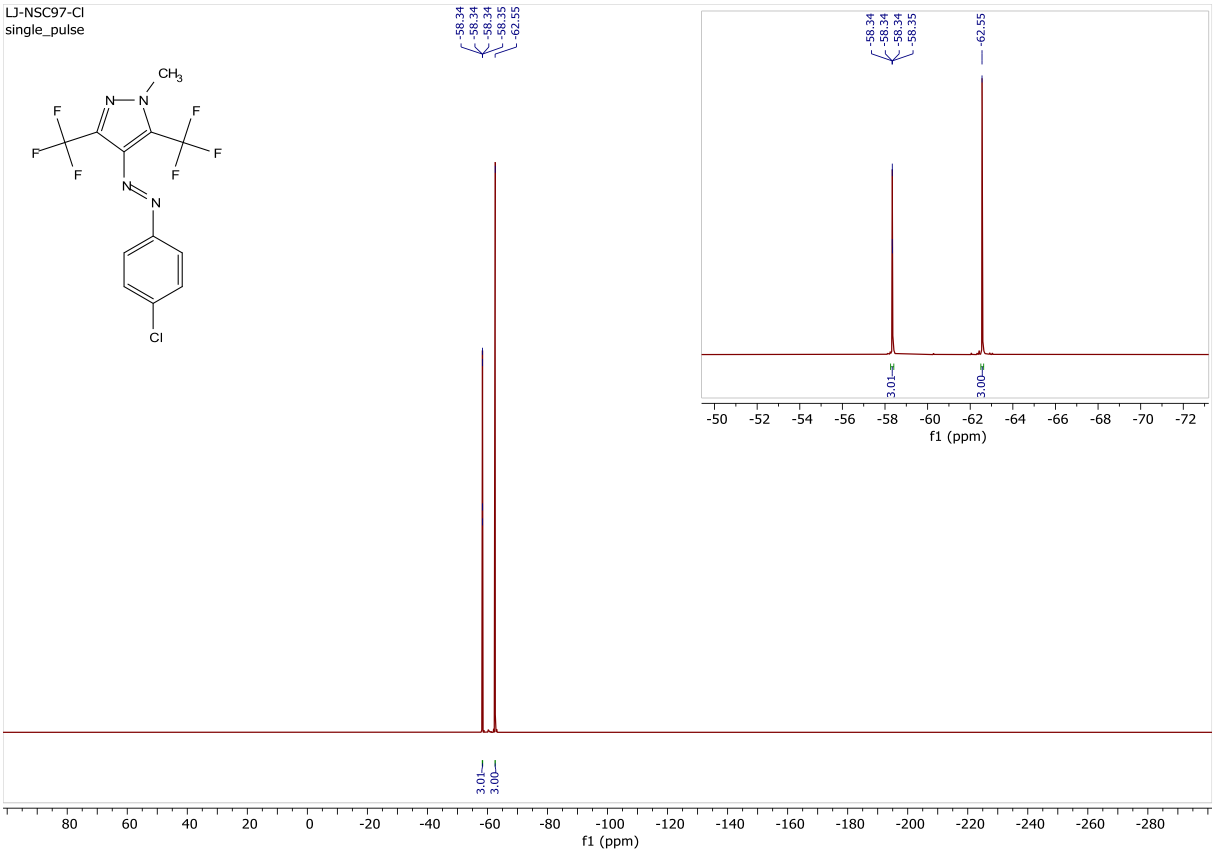

Figure S110: ^{19}F-NMR (376.13 MHz, CD_3CN, 295 K) of **F-PAP-Cl**.

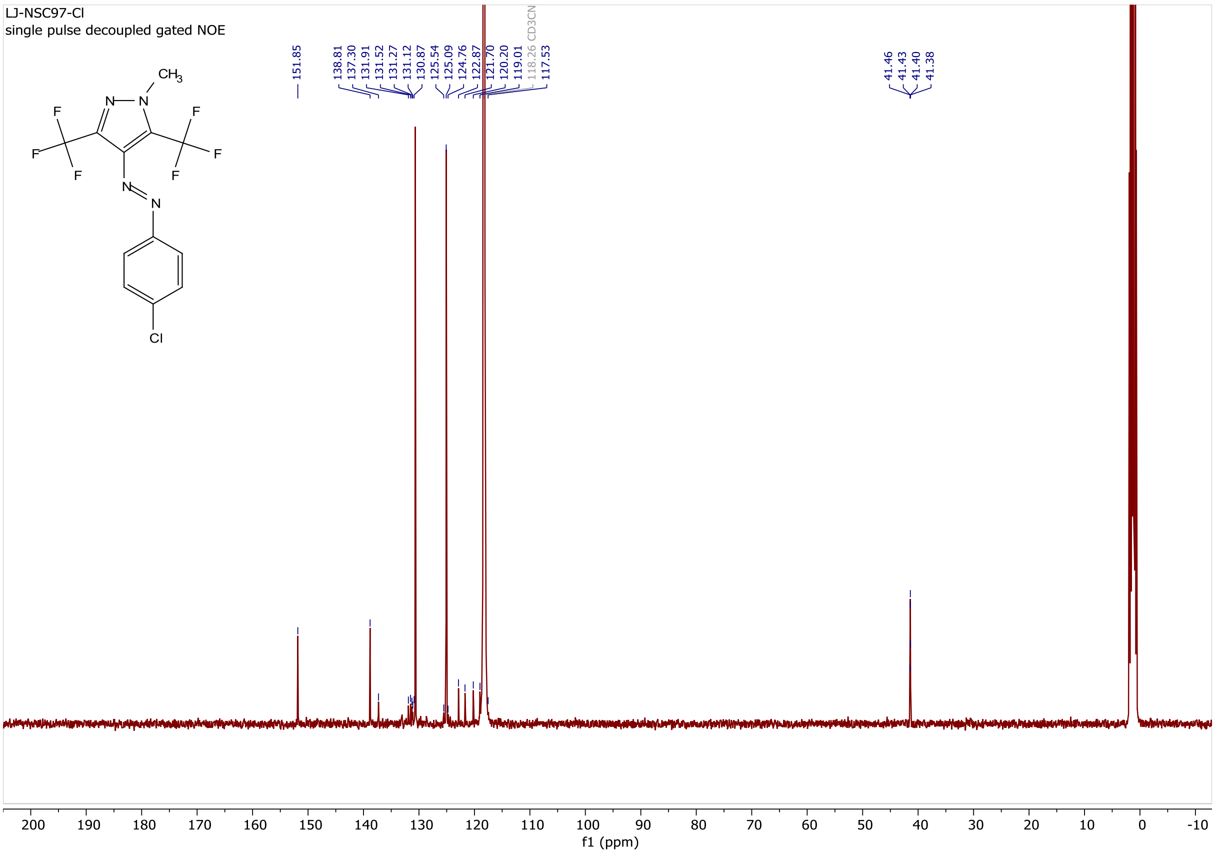

Figure S111: ^{13}C-NMR {^{1}H} (100.54 MHz, CD_3CN, 295 K) of **F-PAP-Cl**.

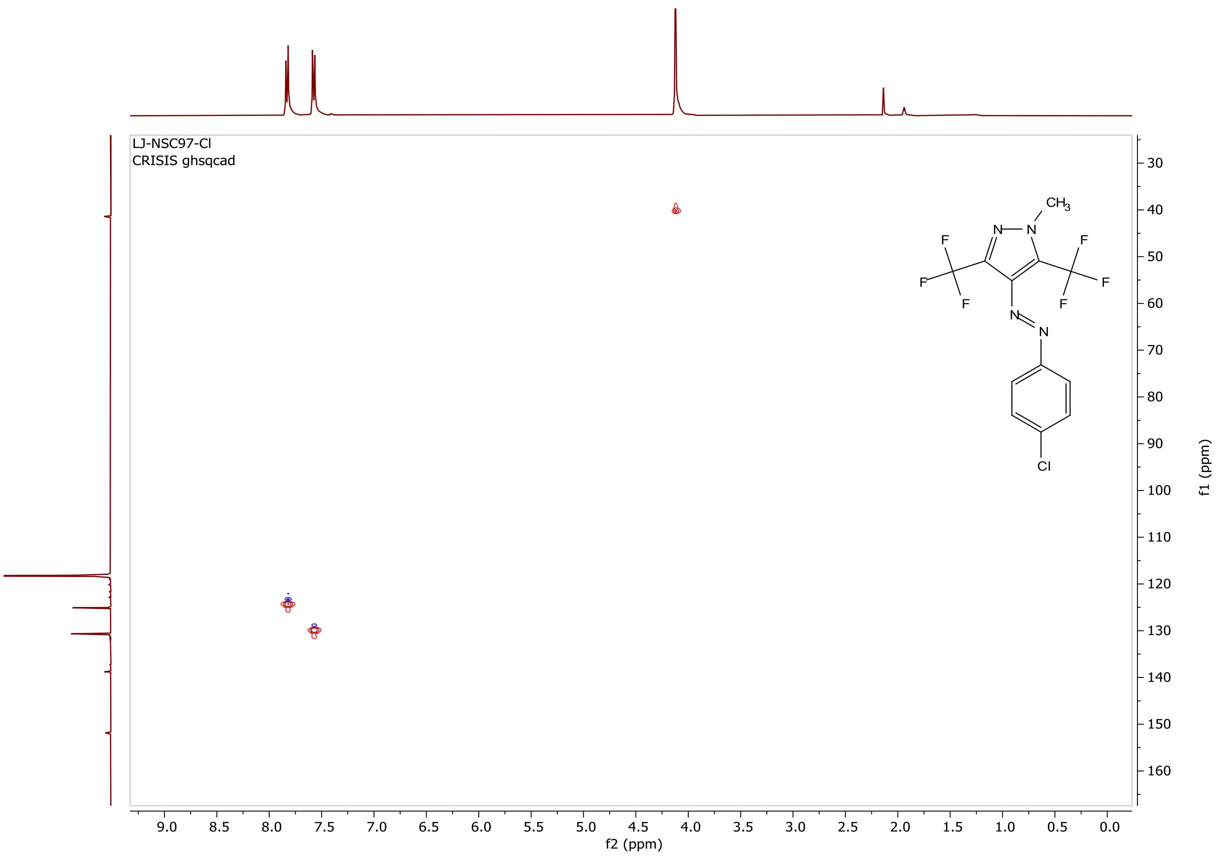

Figure S112: HSQC [¹H ↔¹³C, (399.78 MHz /100.54 MHz, CD_3CN, 295 K) of **F-PAP-Cl**.

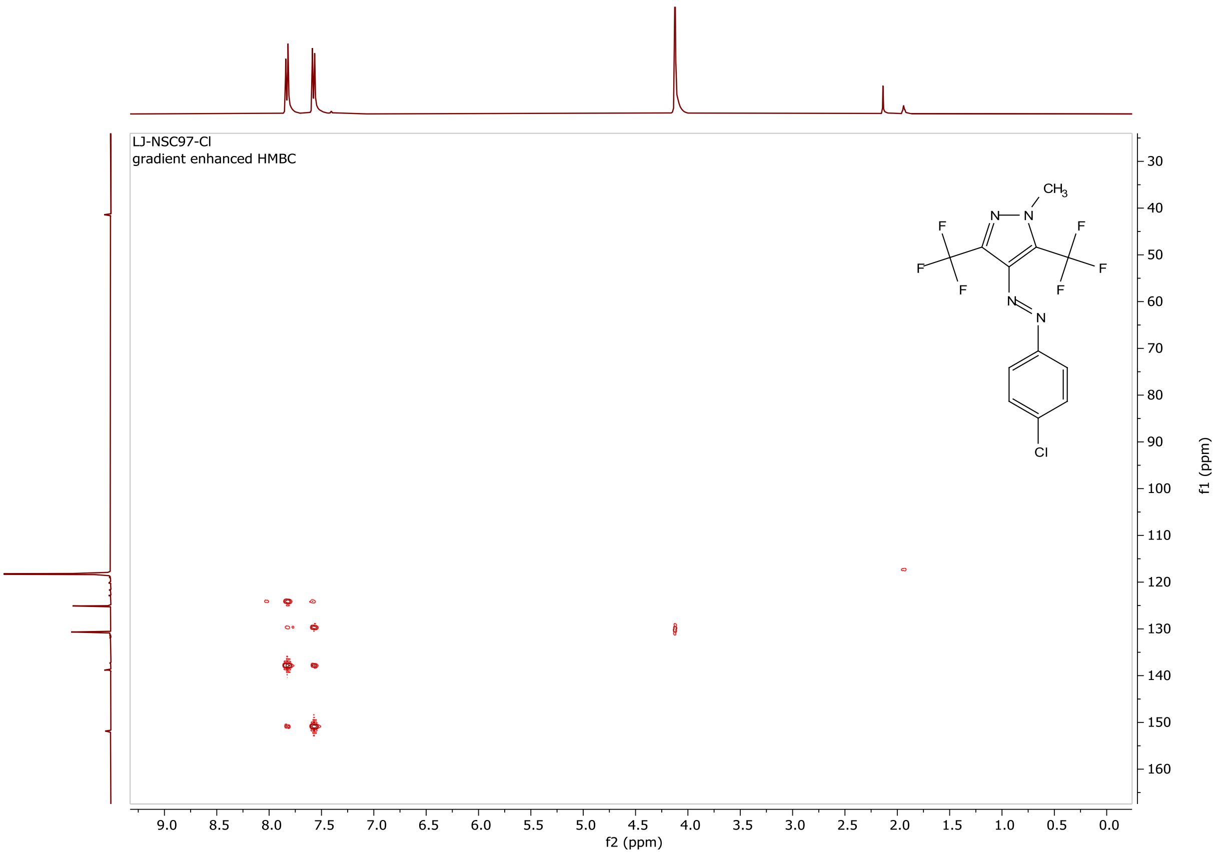

Figure S113: HMBC [¹H ↔¹³C, (399.78 MHz /100.54 MHz, CD_3CN, 295 K) of **F-PAP-Cl**.

F-PAP-Br: (*E*)-4-((4-Bromophenyl)diazenyl)-1-methyl-3,5-bis(trifluoromethyl)-1*H*-pyrazole

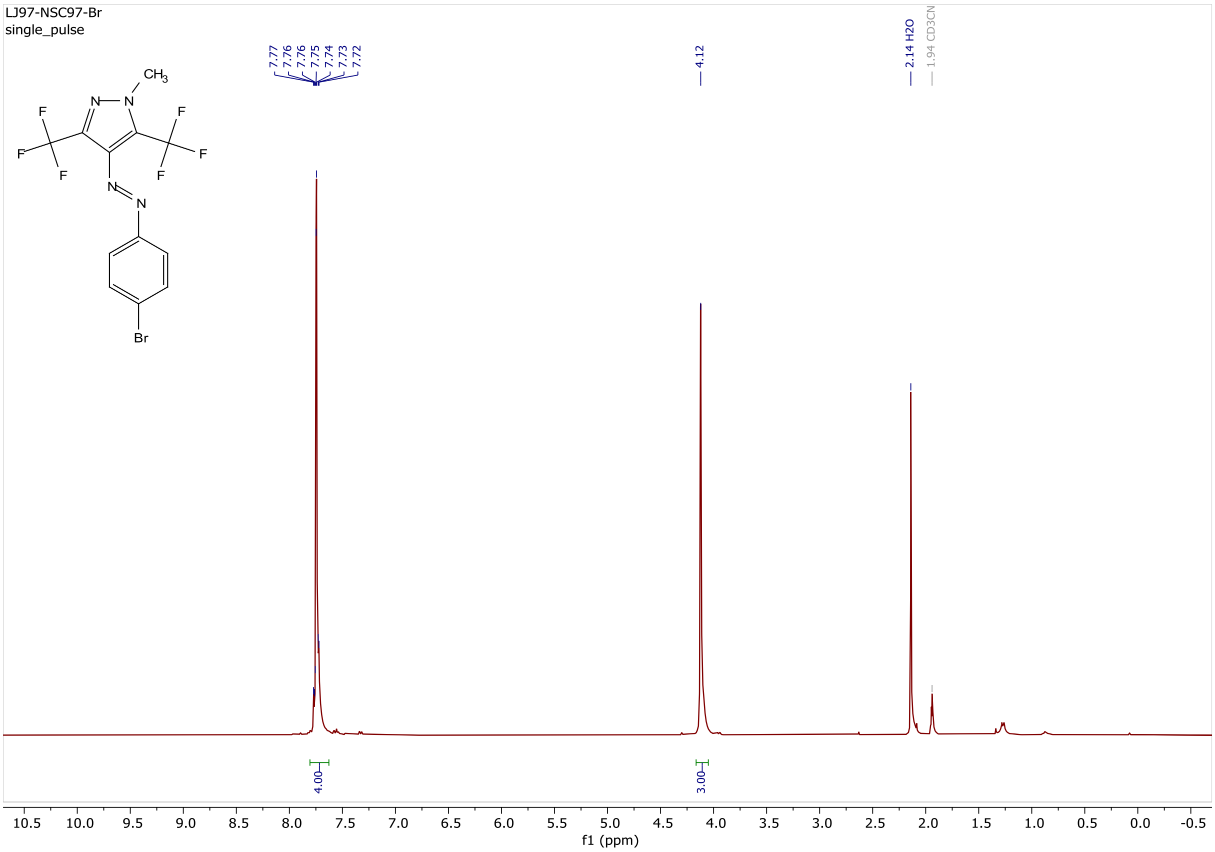

Figure S114: ^{1}H-NMR (399.78 MHz, CD_3CN, 295 K) of **F-PAP-Br**.

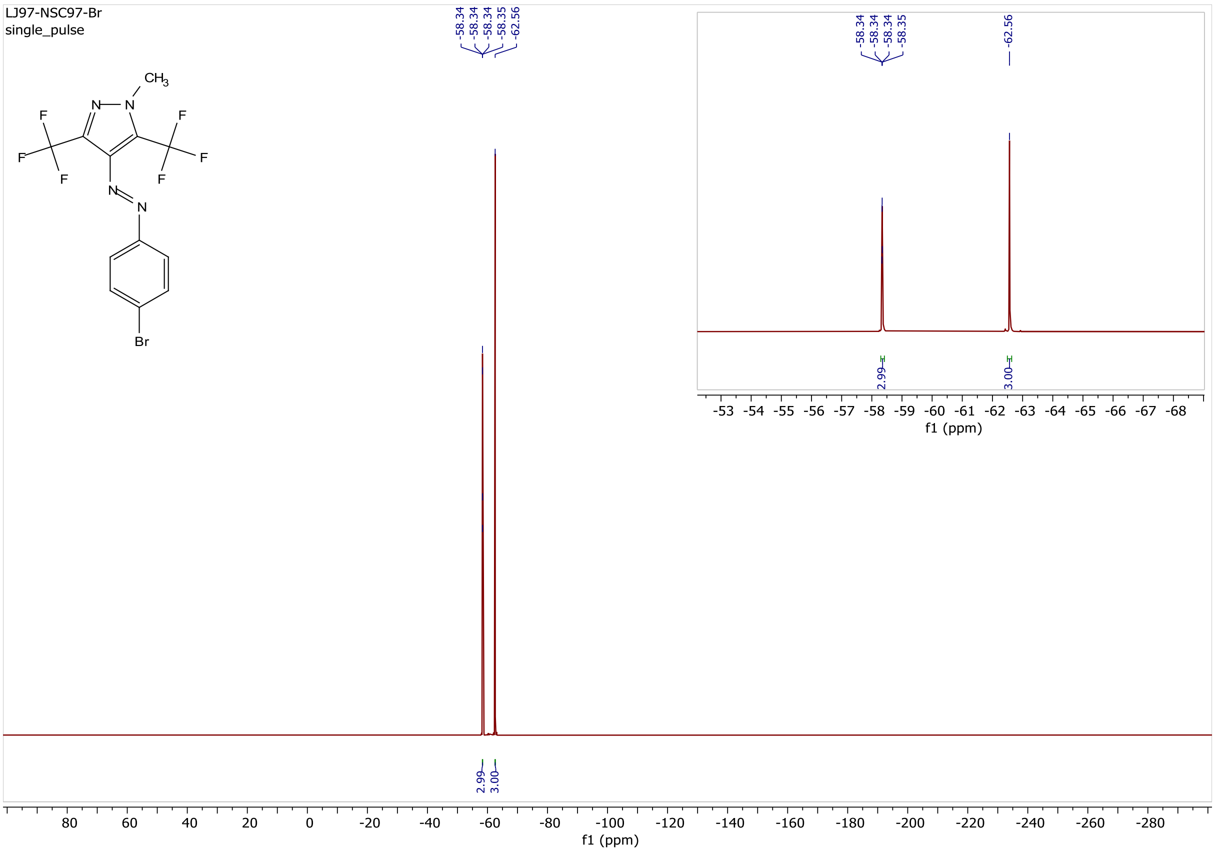

Figure S115: ^{19}F-NMR (376.13 MHz, CD_3CN, 295 K) of **F-PAP-Br**.

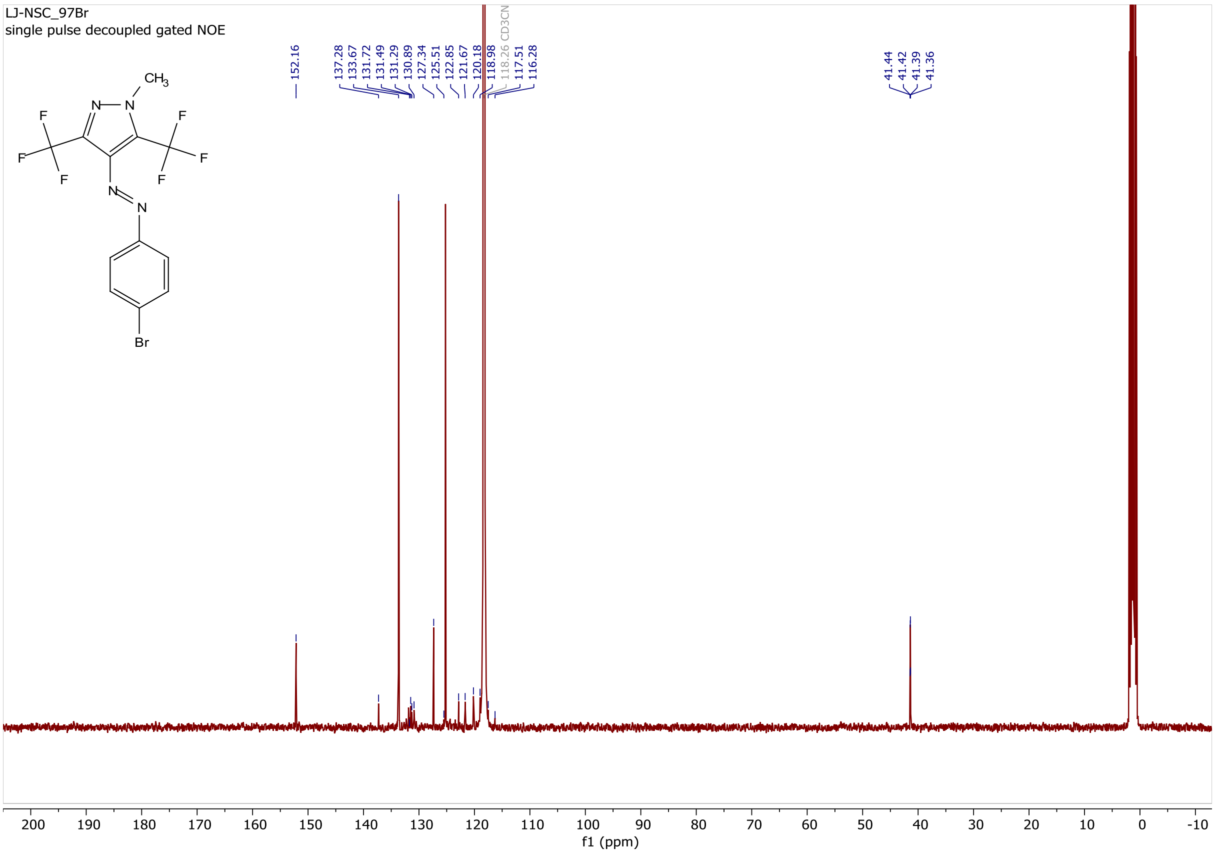

Figure S116: ^{13}C-NMR {^1H} (100.54 MHz, CD_3CN, 295 K) of **F-PAP-Br**.

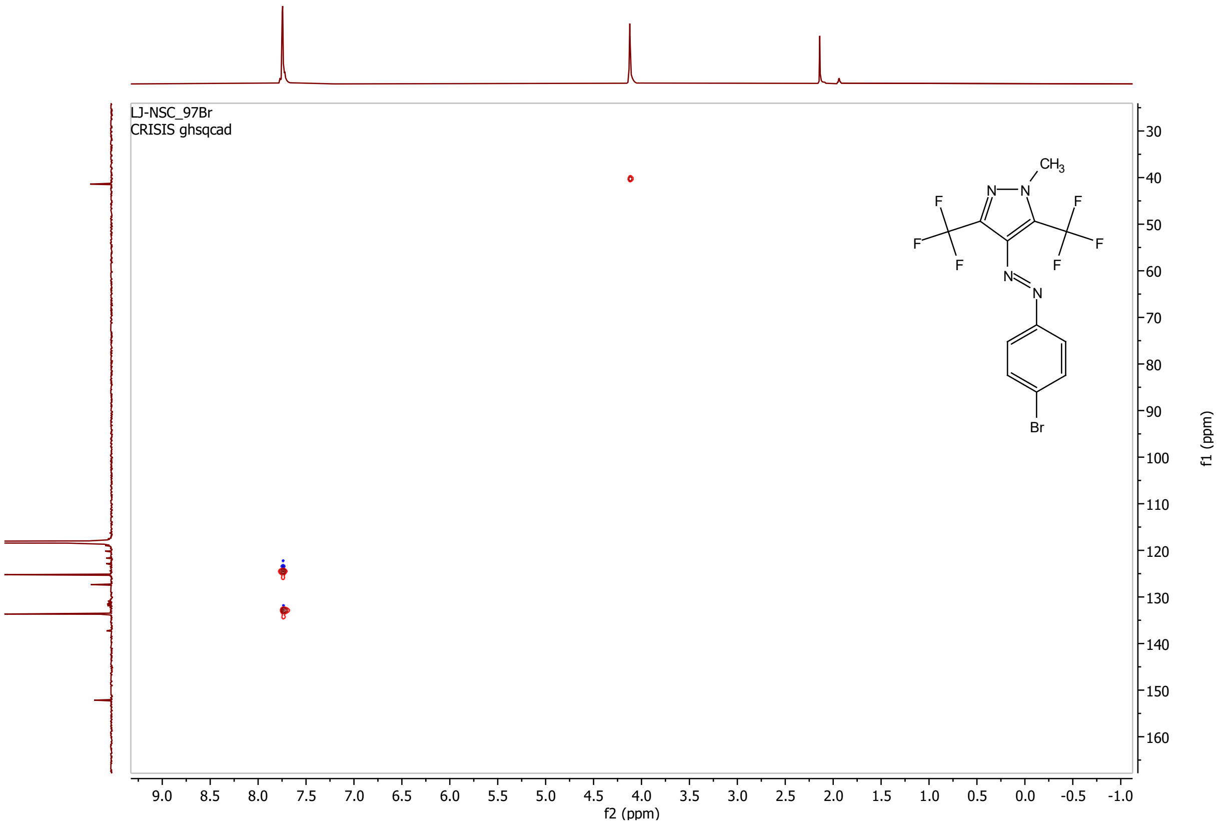

Figure S117: HSQC [$^{1}H \leftrightarrow ^{13}C$, (399.78 MHz /100.54 MHz, CD_3CN, 295 K) of **F-PAP-Br**.

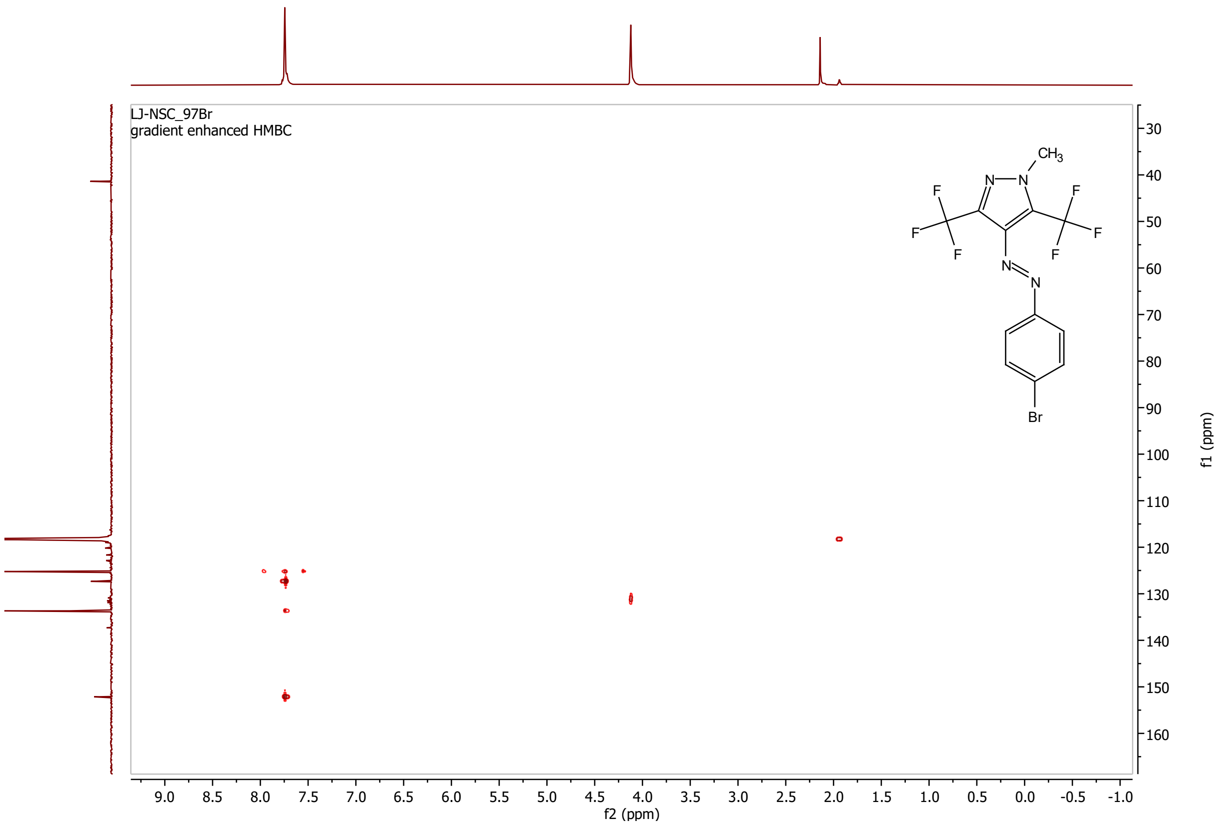

Figure S118: HMBC [$^{1}H \leftrightarrow ^{13}C$, (399.78 MHz /100.54 MHz, CD_3CN, 295 K) of **F-PAP-Br**.

F-PAP-CN: (*E*)-4-((1-Methyl-3,5-bis(trifluoromethyl)-1*H*-pyrazol-4-yl)diazenyl)benzonitrile

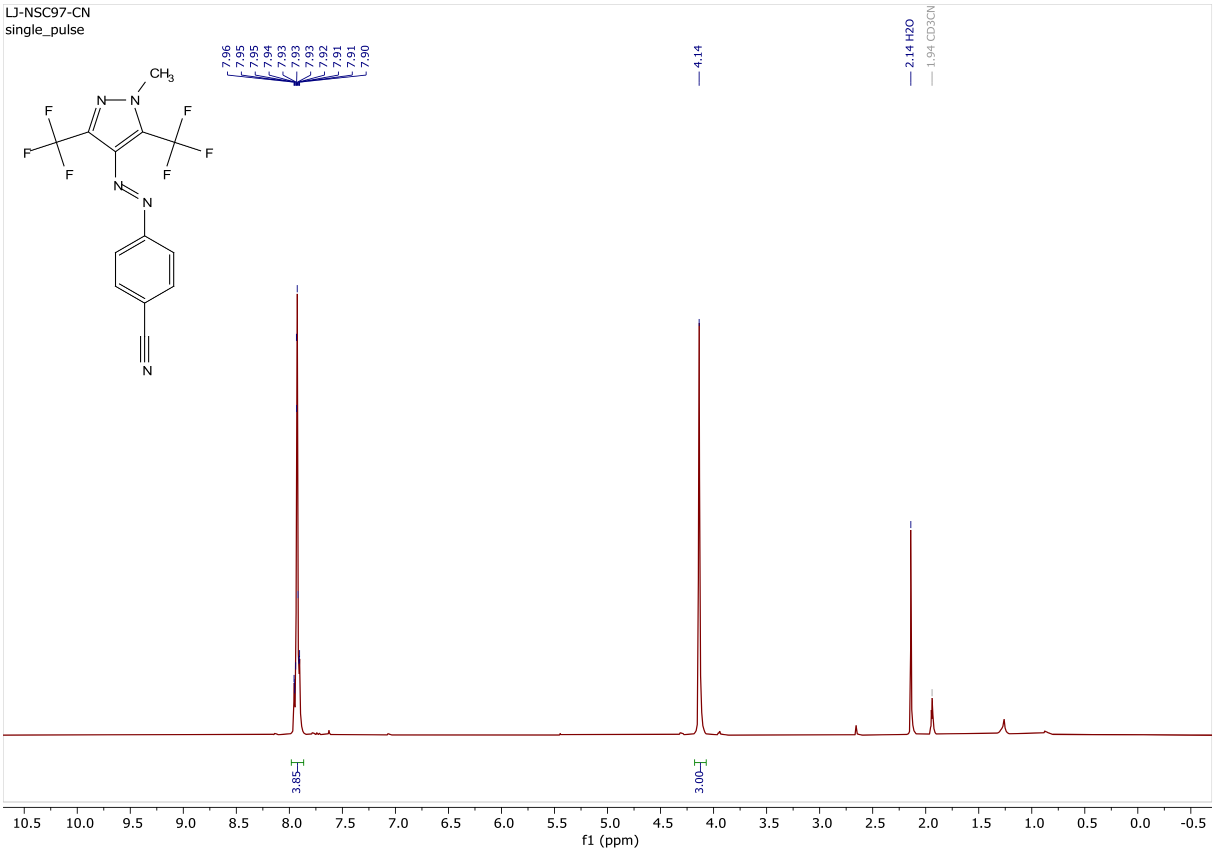

Figure S119: ^{1}H-NMR (399.78 MHz, CD_3CN, 295 K) of **F-PAP-CN**.

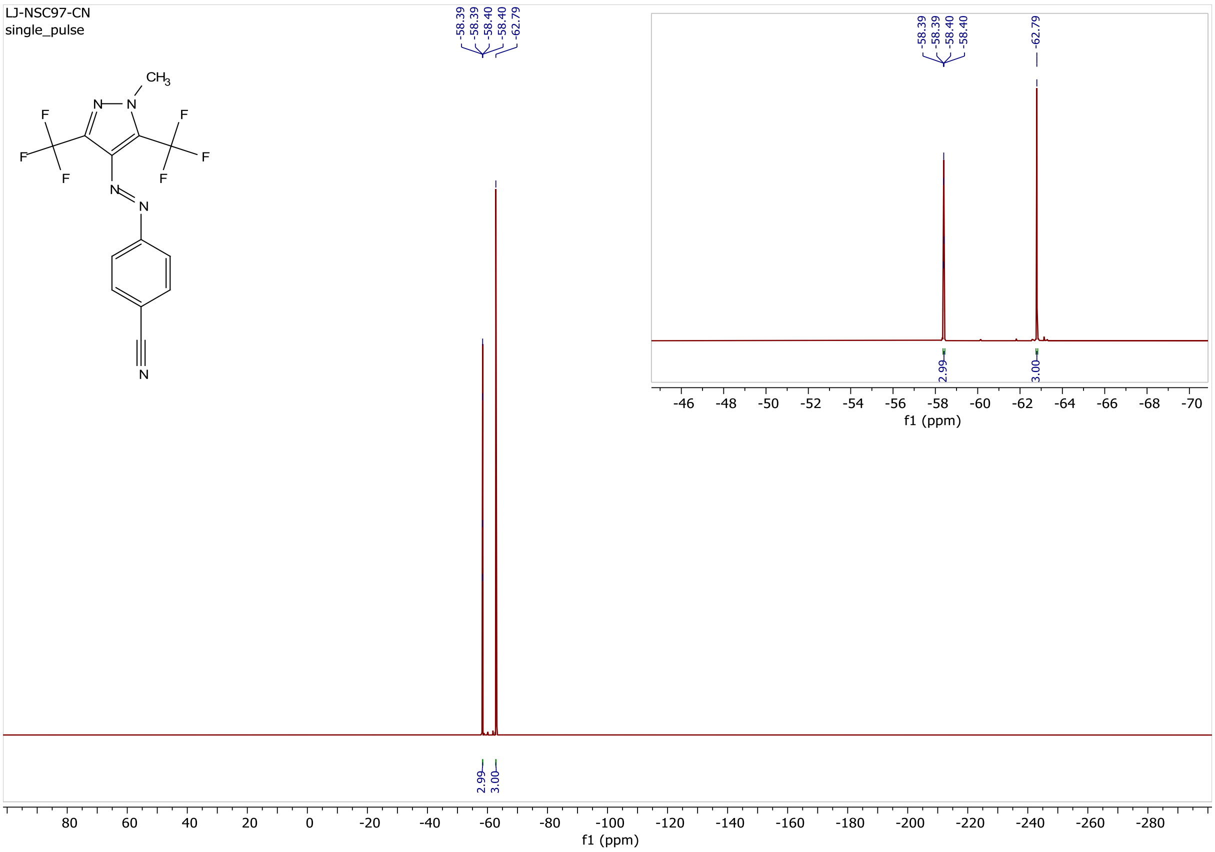

Figure S120: ^{19}F-NMR (376.13 MHz, CD_3CN, 295 K) of **F-PAP-CN**.

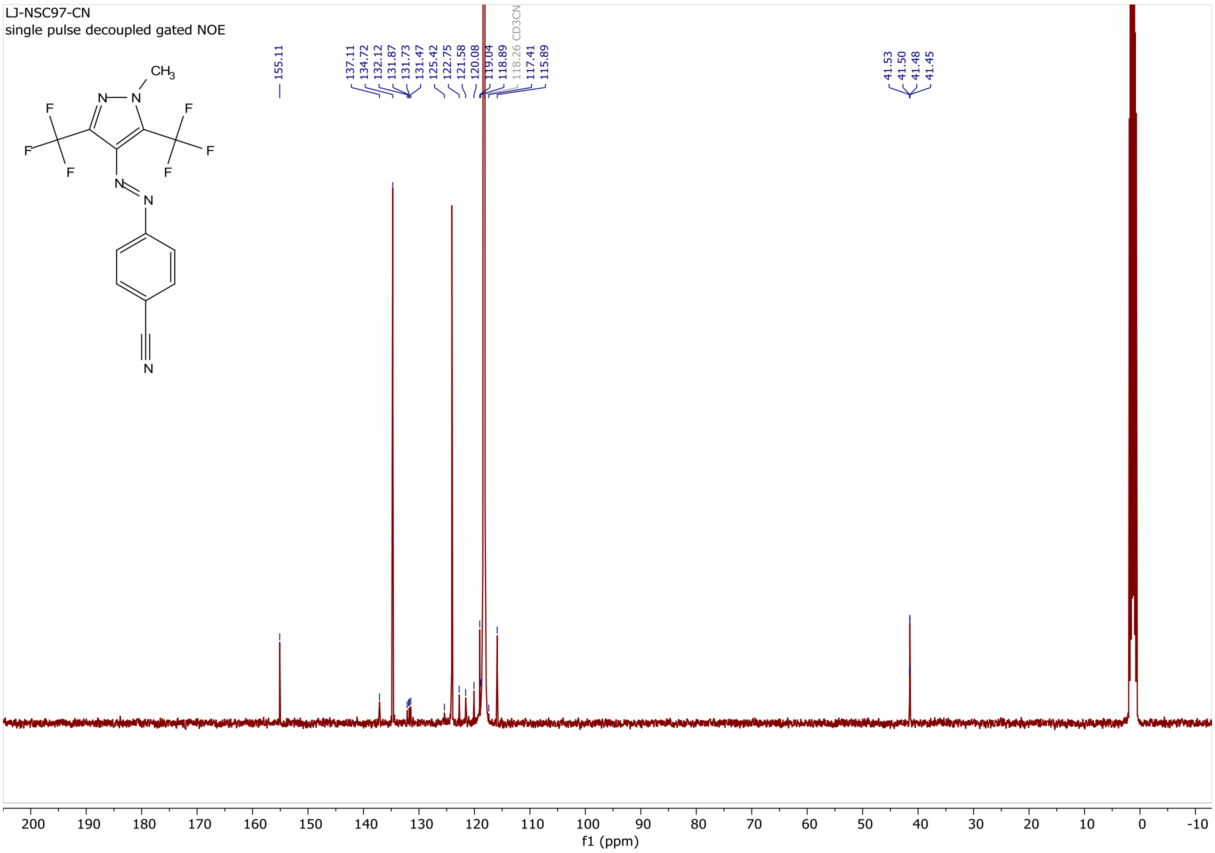

Figure S121: ^{13}C-NMR {^{1}H} (100.54 MHz, CD_3CN, 295 K) of **F-PAP-CN**.

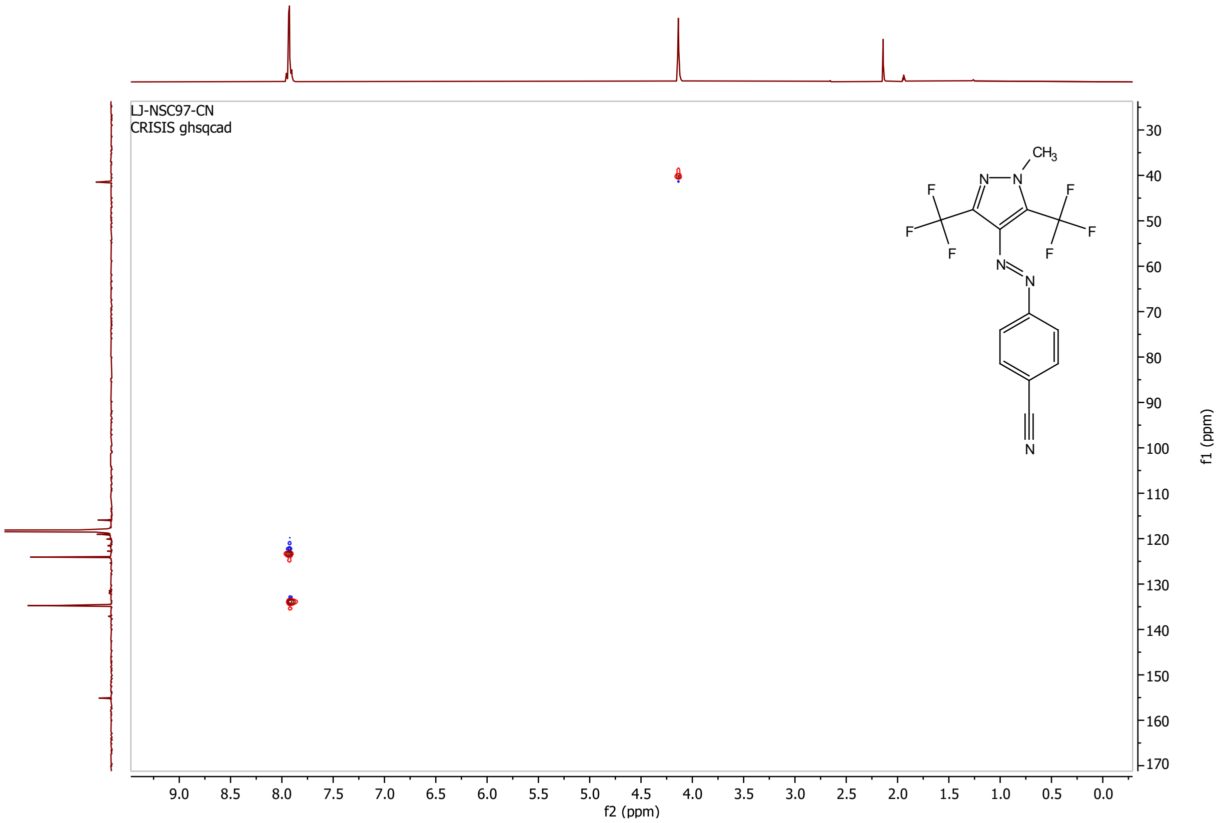

Figure S122: HSQC [$^{1}H \leftrightarrow {}^{13}C$, (399.78 MHz /100.54 MHz, CD_3CN, 295 K) of **F-PAP-CN**.

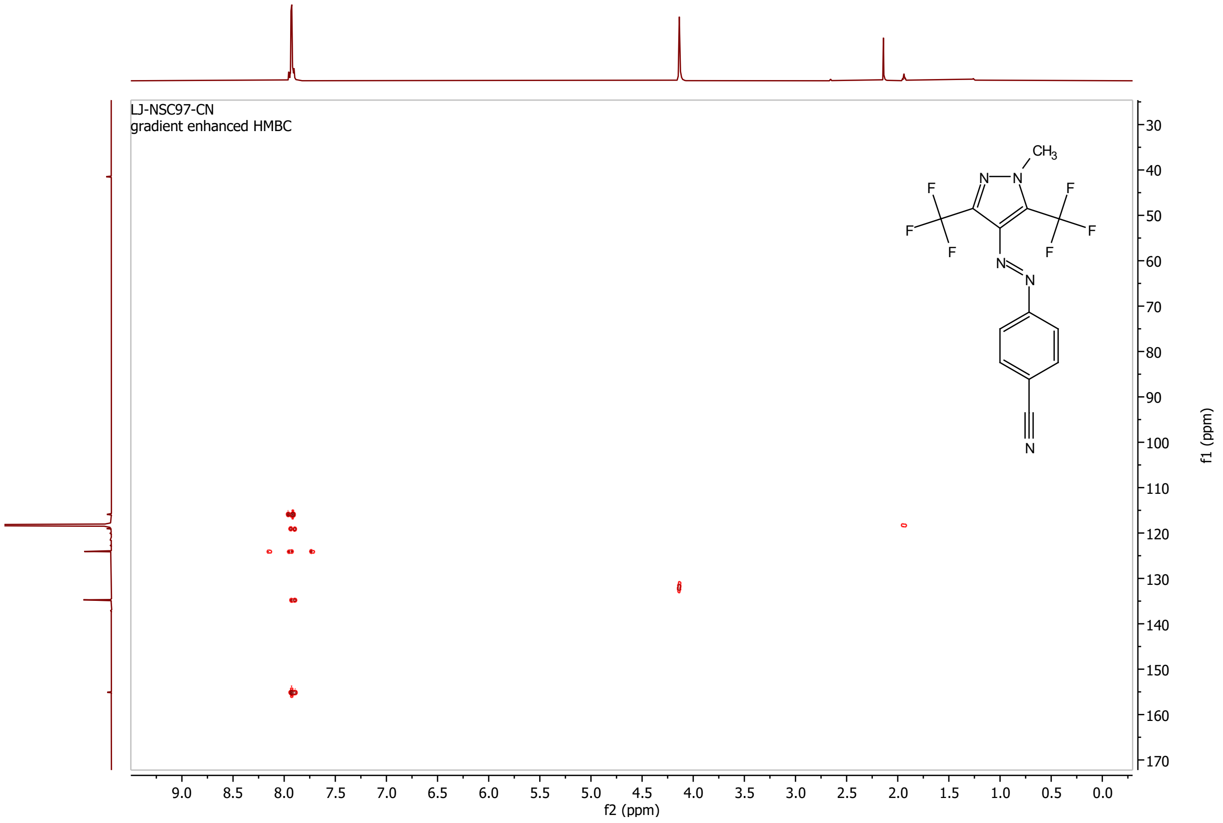

Figure S123: HMBC [$^{1}H \leftrightarrow {}^{13}C$, (399.78 MHz /100.54 MHz, CD_3CN, 295 K) of **F-PAP-CN**.

F-PAP-Me: (*E*)-1-Methyl-4-(*p*-tolyldiazenyl)-3,5-bis(trifluoromethyl)-1*H*-pyrazole

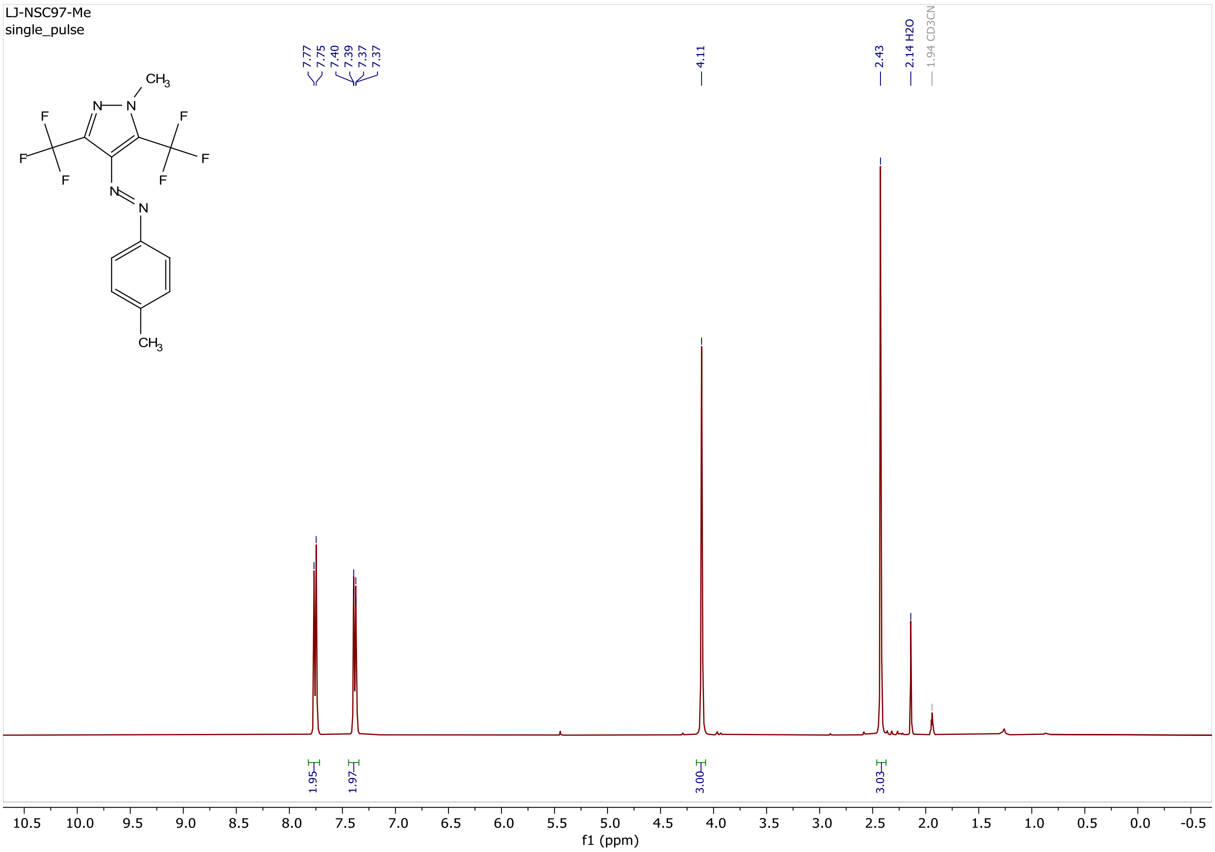

Figure S124: ^{1}H-NMR (399.78 MHz, CD_3CN, 295 K) of **F-PAP-Me**.

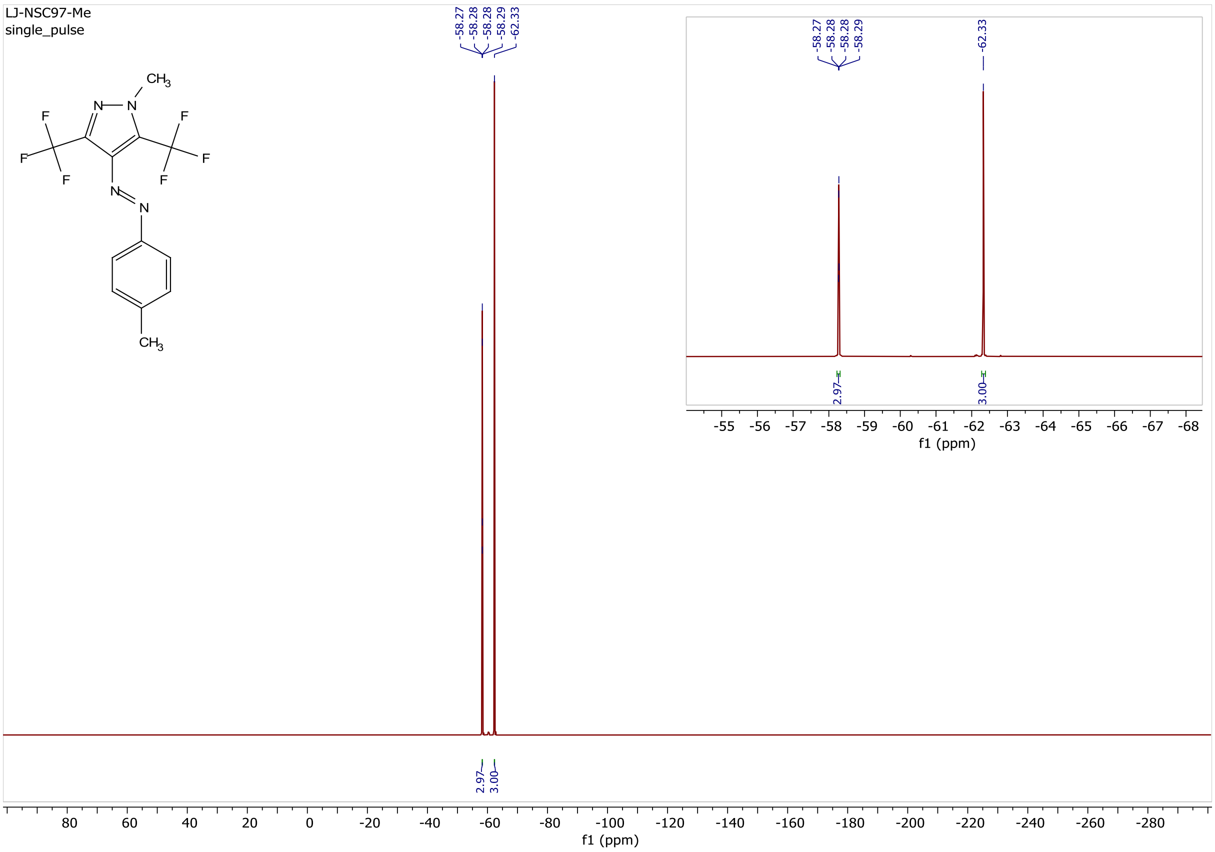

Figure S125: ^{19}F-NMR (376.13 MHz, CD_3CN, 295 K) of **F-PAP-Me**.

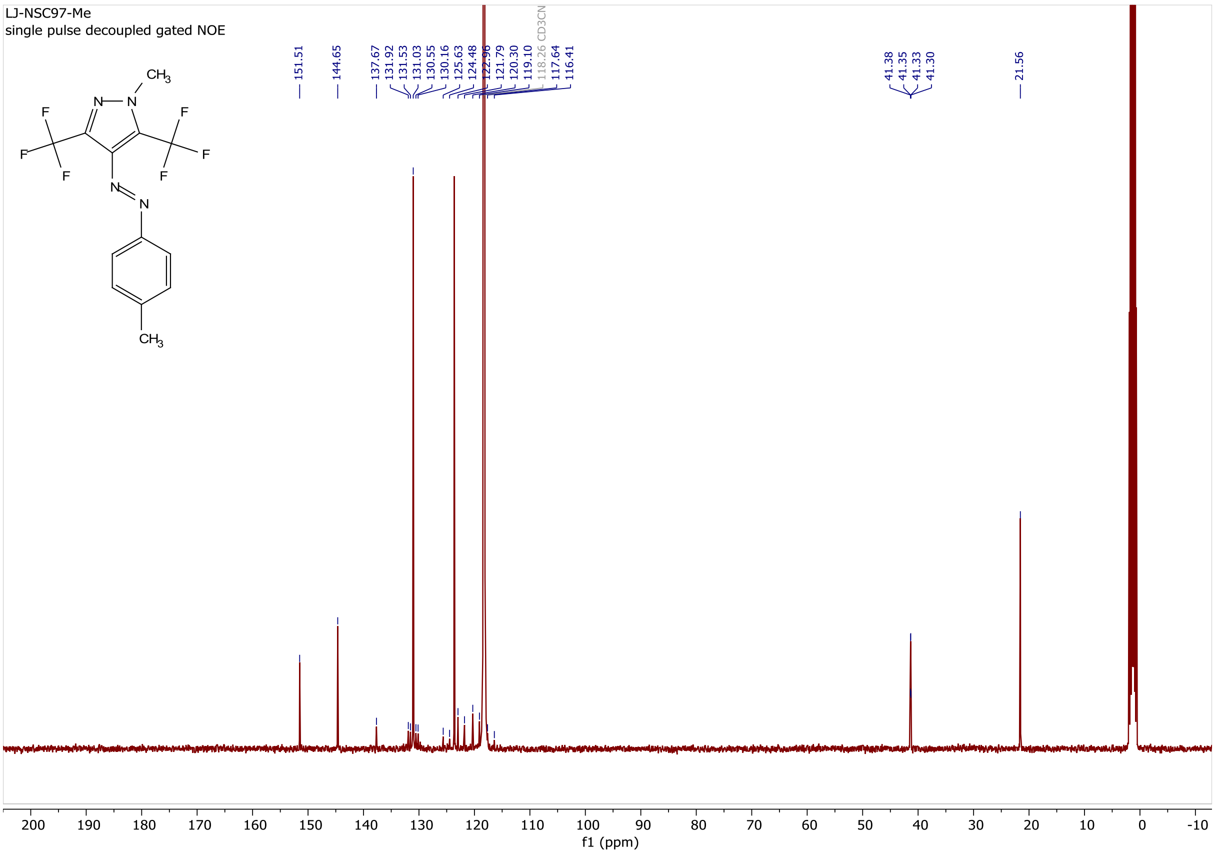

Figure S126: ^{13}C-NMR {^{1}H} (100.54 MHz, CD_3CN, 295 K) of **F-PAP-Me**.

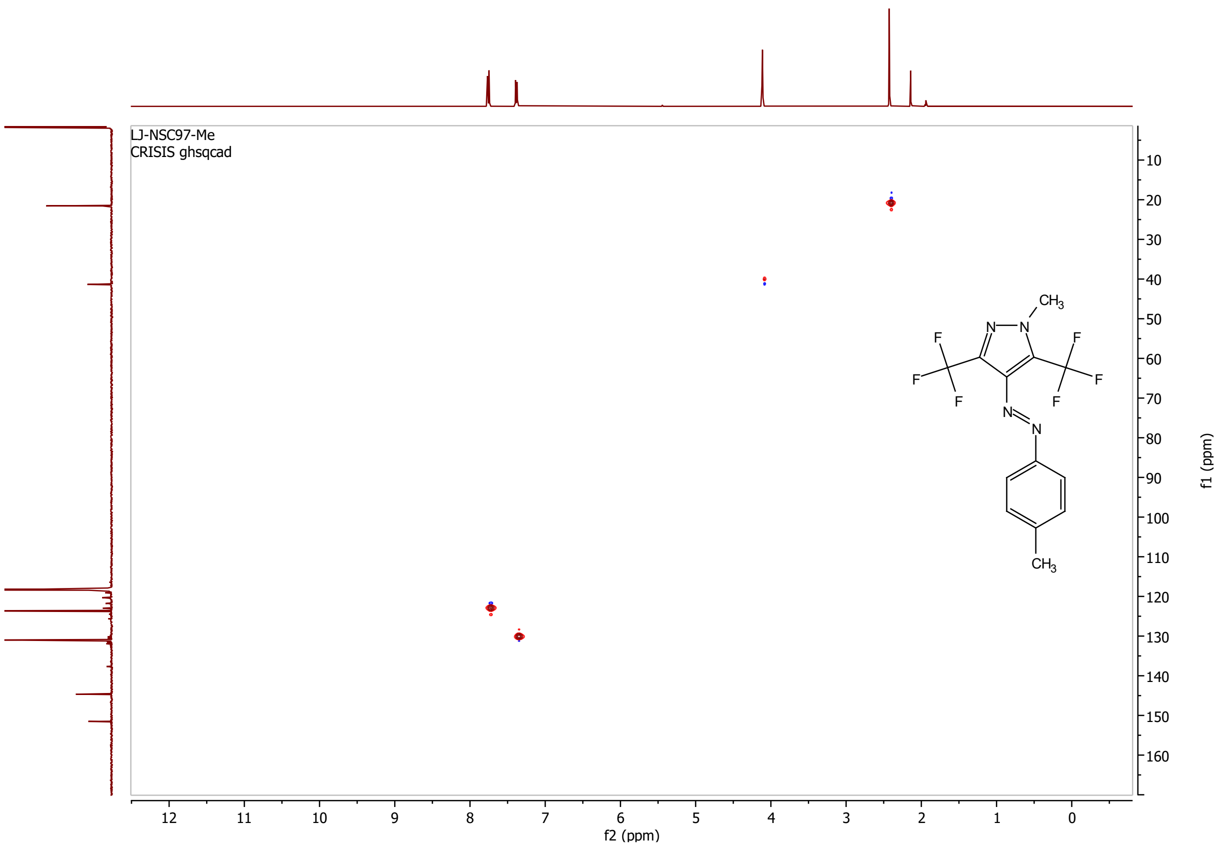

Figure S127: HSQC [^{1}H ↔^{13}C, (399.78 MHz /100.54 MHz, CD_3CN, 295 K) of **F-PAP-Me**.

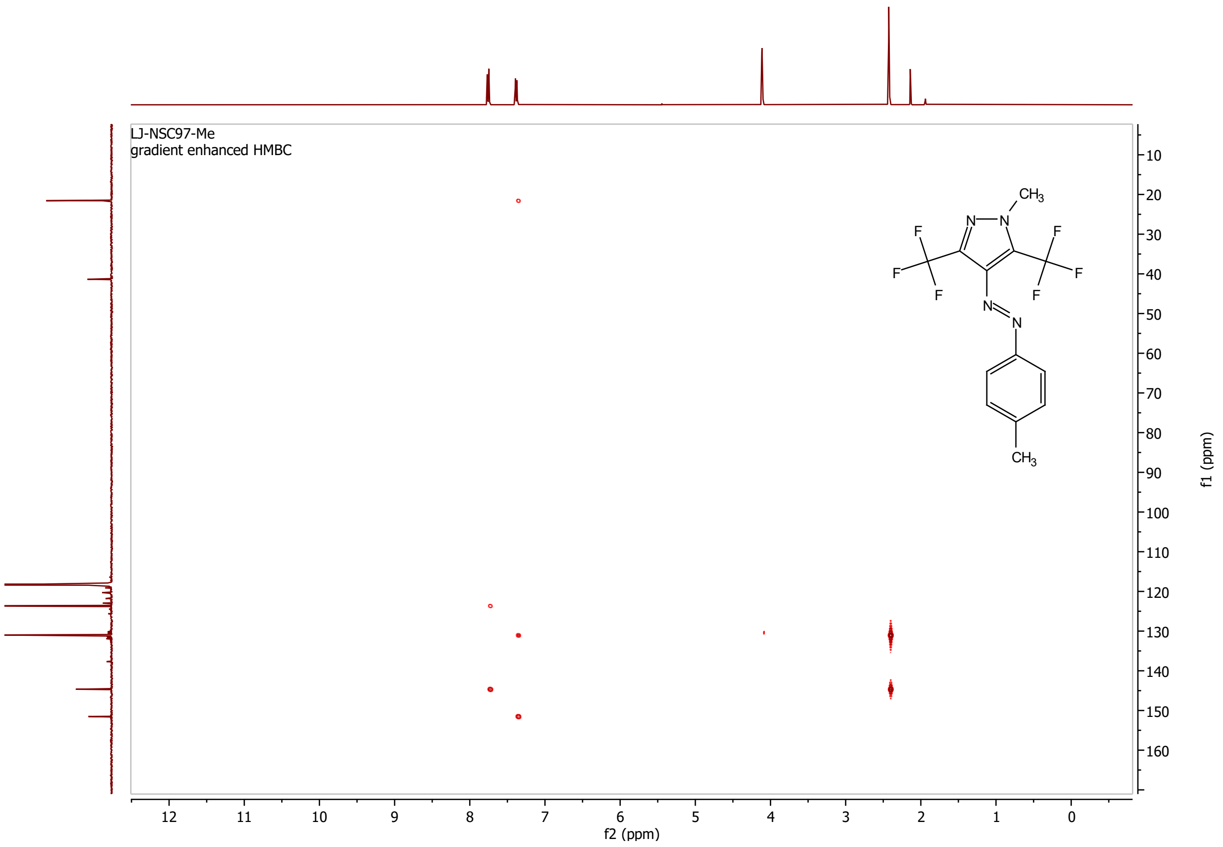

Figure S128: HMBC [^{1}H ↔^{13}C, (399.78 MHz /100.54 MHz, CD_3CN, 295 K) of **F-PAP-Me**.

F-PAP-CF$_3$: (*E*)-1-Methyl-3,5-bis(trifluoromethyl)-4-((4 (trifluoromethyl)phenyl)diazenyl)-1*H*-pyrazole

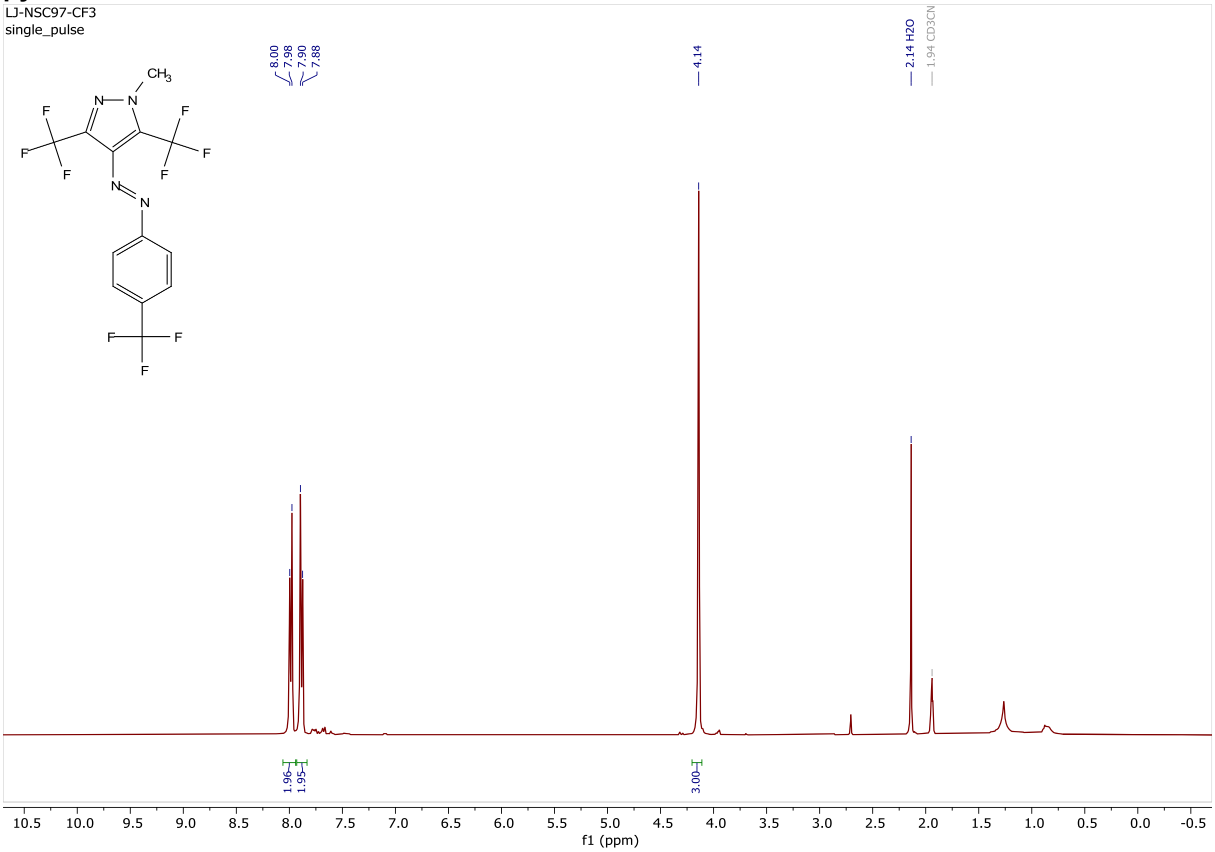

Figure S129: ^{1}H-NMR (399.78 MHz, CD_3CN, 295 K) of **F-PAP-CF$_3$**.

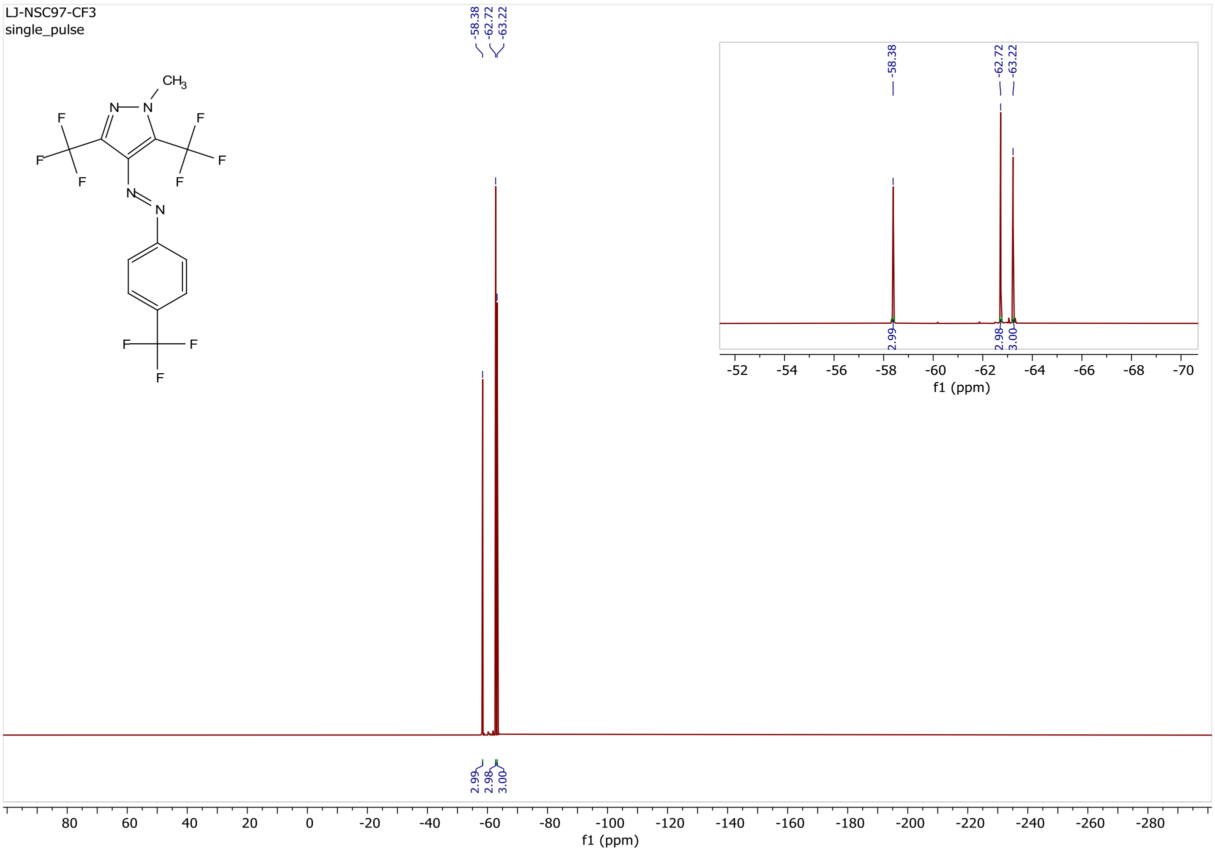

Figure S130: ^{19}F-NMR (376.13 MHz, CD_3CN, 295 K) of **F-PAP-CF$_3$**.

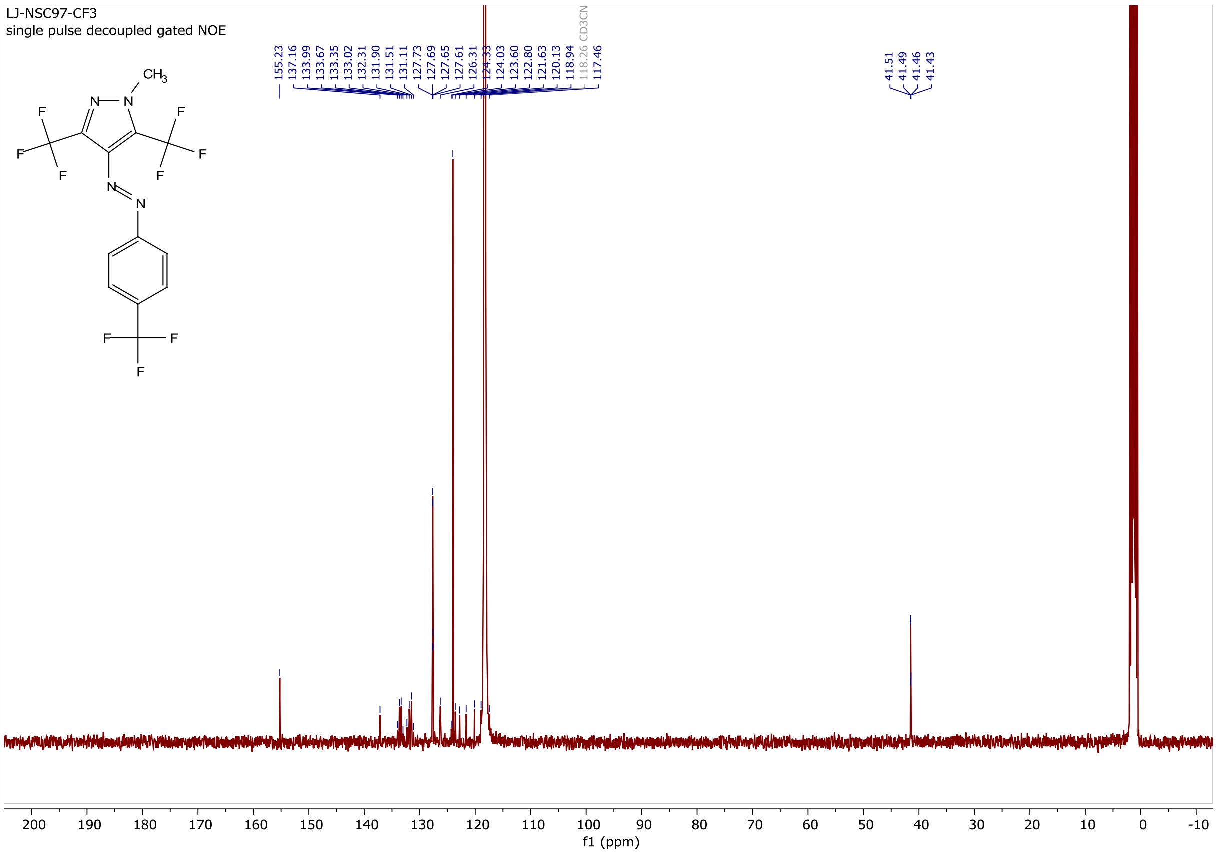

Figure S131: ^{13}C-NMR {^{1}H} (100.54 MHz, CD_3CN, 295 K) of **F-PAP-CF$_3$**.

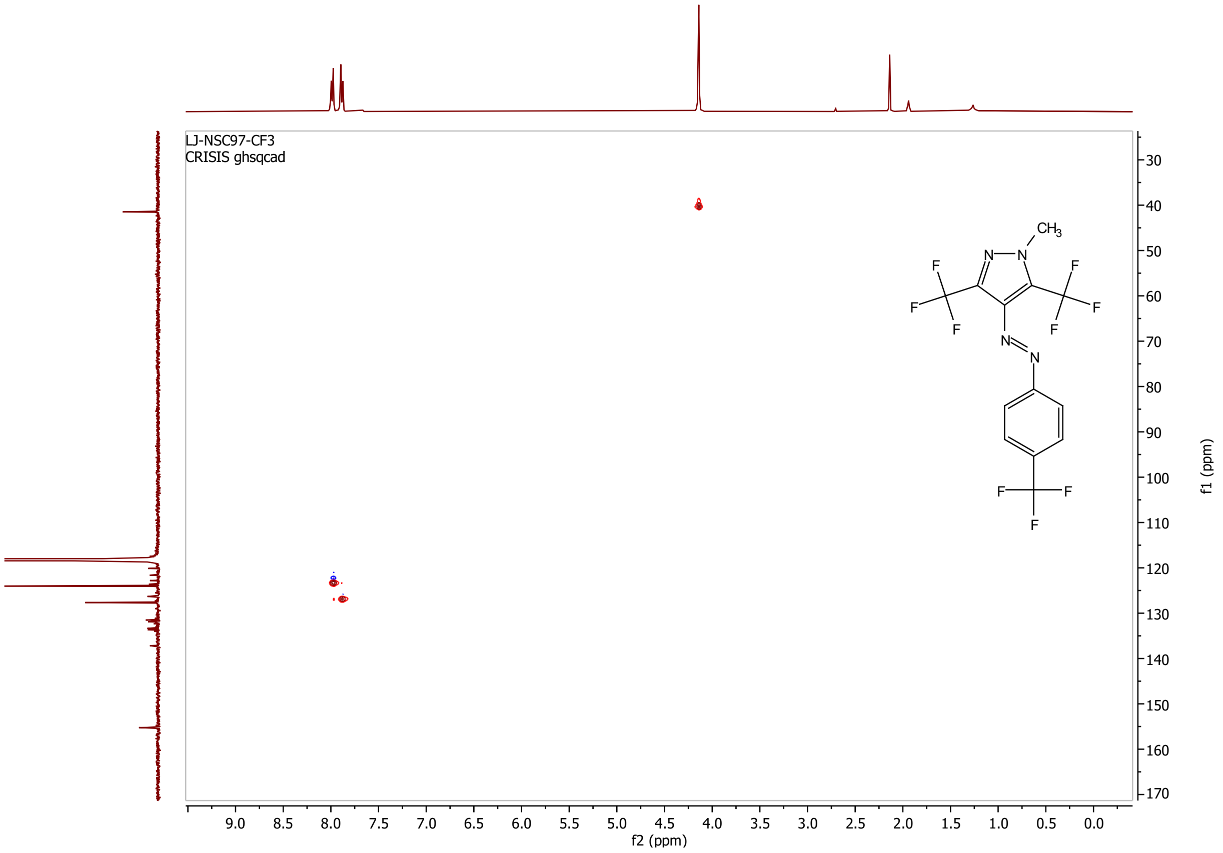

Figure S132: HSQC [^{1}H ↔^{13}C, (399.78 MHz /100.54 MHz, CD_3CN, 295 K) of **F-PAP-CF$_3$**.

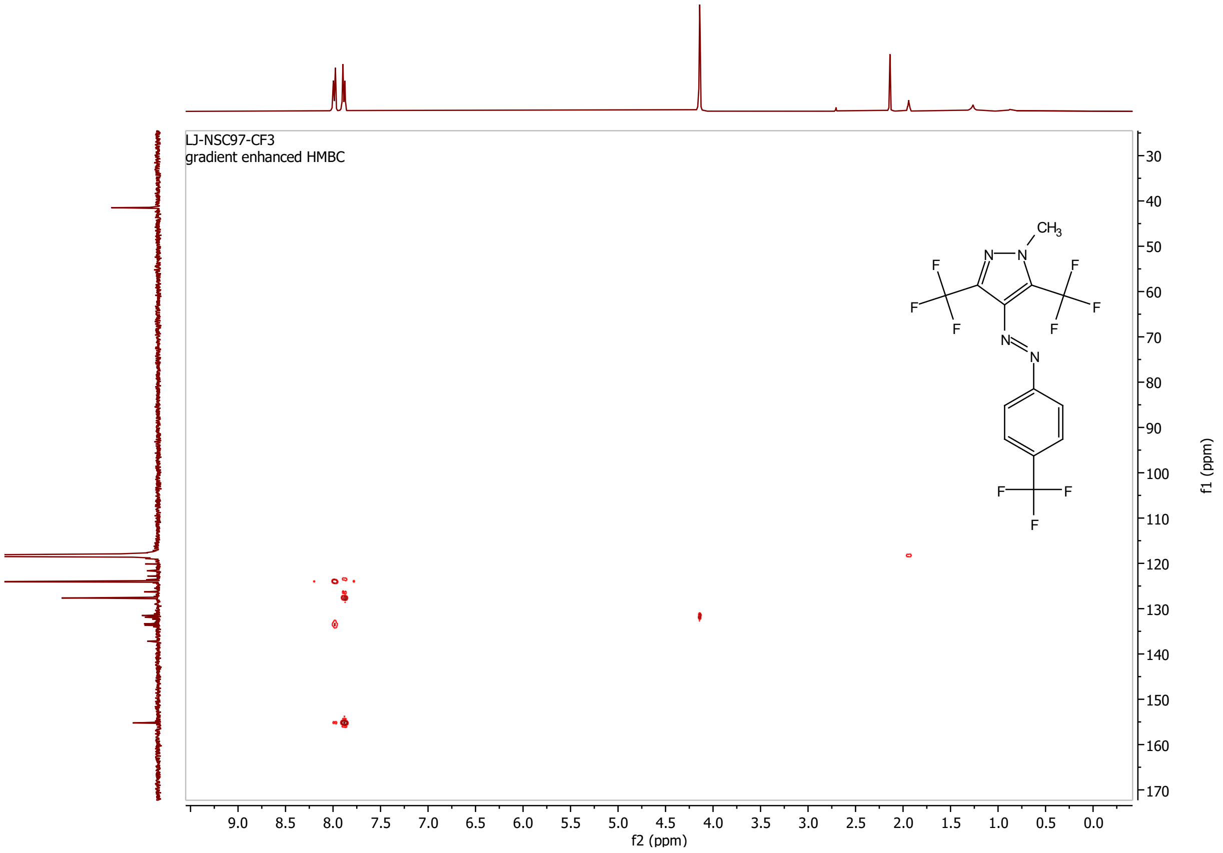

Figure S133: HMBC [$^1H \leftrightarrow ^{13}C$, (399.78 MHz /100.54 MHz, CD_3CN, 295 K) of **F-PAP-CF$_3$**.

F-PAP-OMe: (*E*)-4-((4-Methoxyphenyl)diazenyl)-1-methyl-3,5-bis(trifluoromethyl)-1*H*-pyrazole

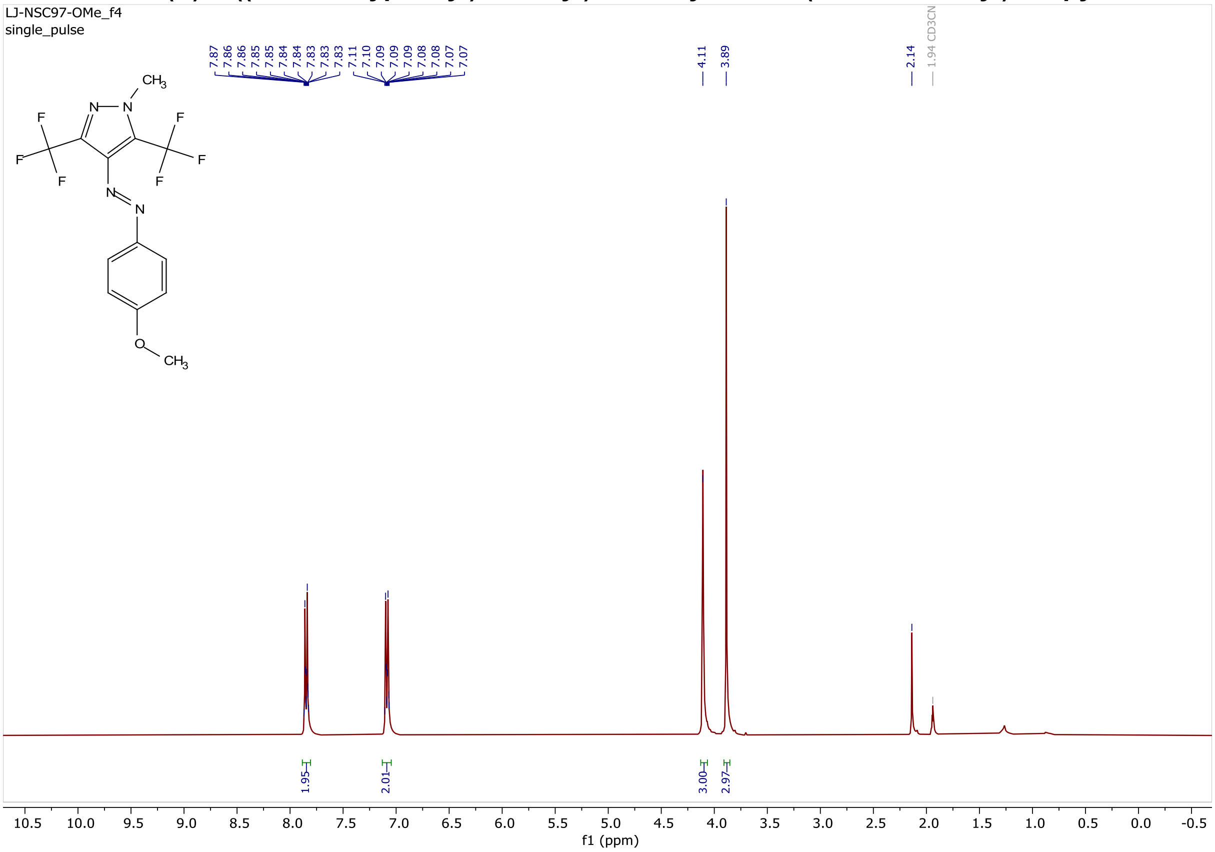

Figure S134: ^{1}H-NMR (399.78 MHz, CD_3CN, 295 K) of **F-PAP-OMe**.

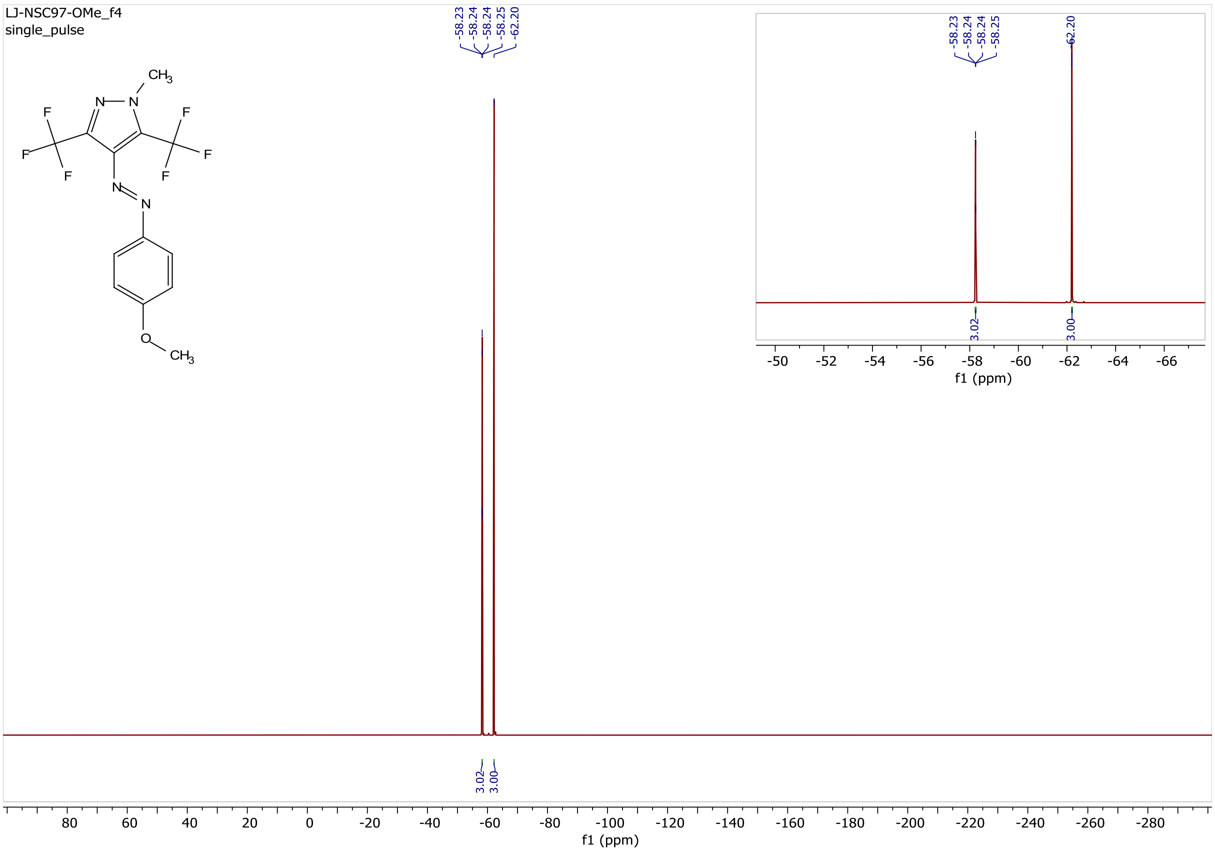

Figure S135: ^{19}F-NMR (376.13 MHz, CD_3CN, 295 K) of **F-PAP-OMe**.

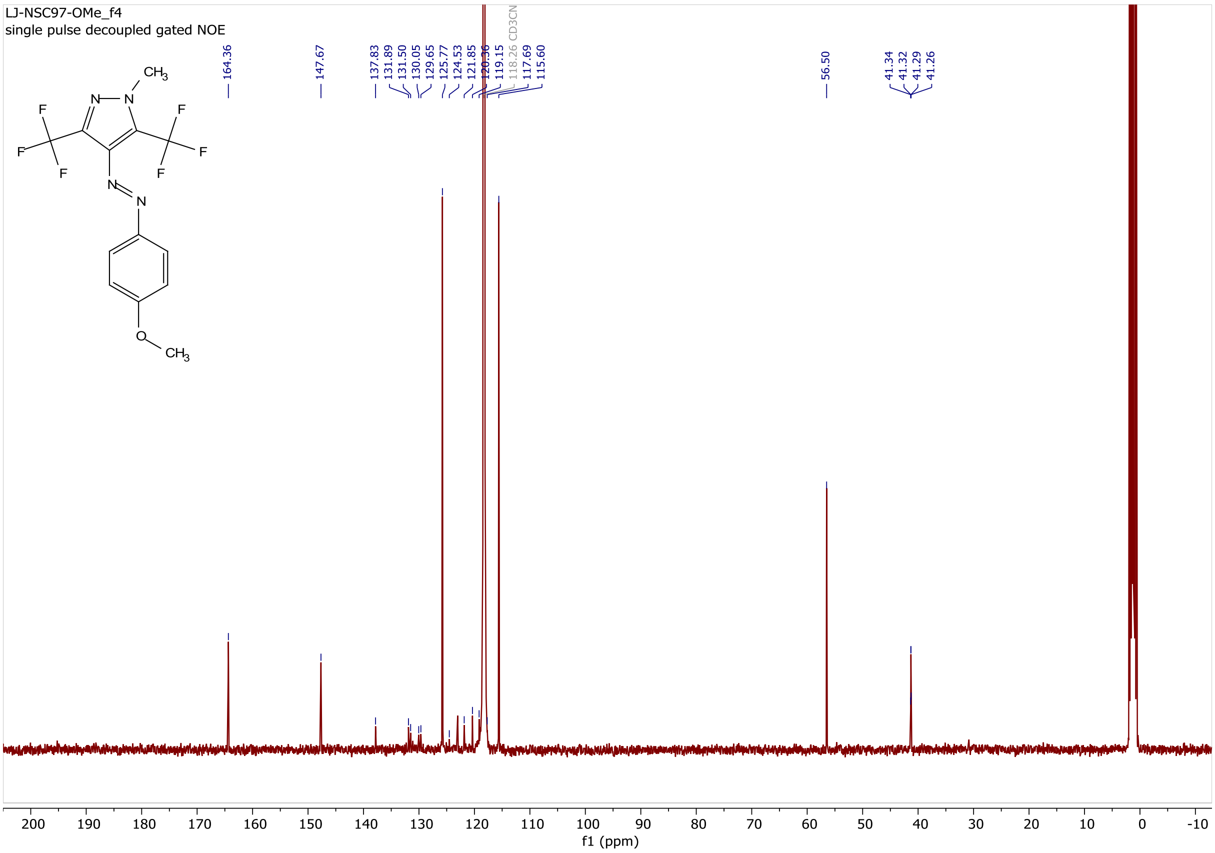

Figure S136: ^{13}C-NMR {^{1}H} (100.54 MHz, CD_3CN, 295 K) of **F-PAP-OMe**.

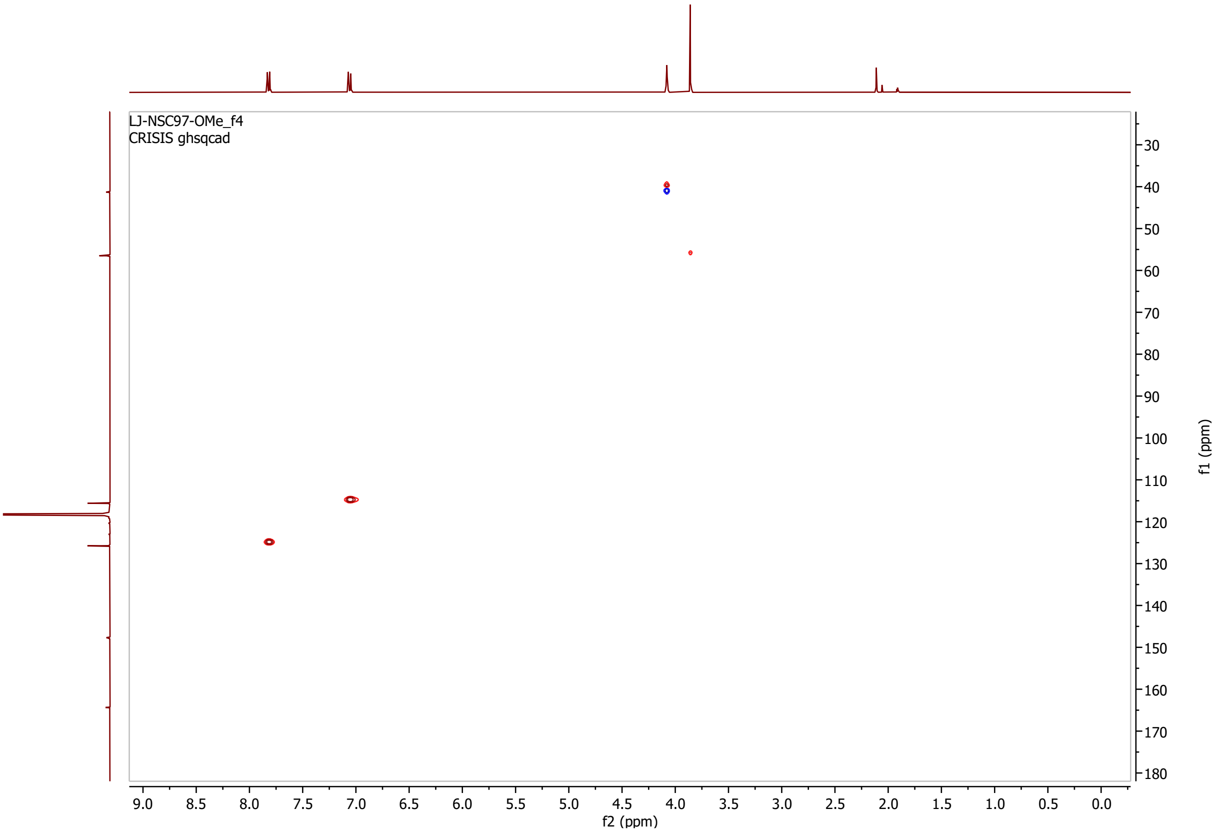

Figure S137: HSQC [$^{1}H \leftrightarrow ^{13}C$, (399.78 MHz /100.54 MHz, CD_3CN, 295 K) of **F-PAP-OMe**.

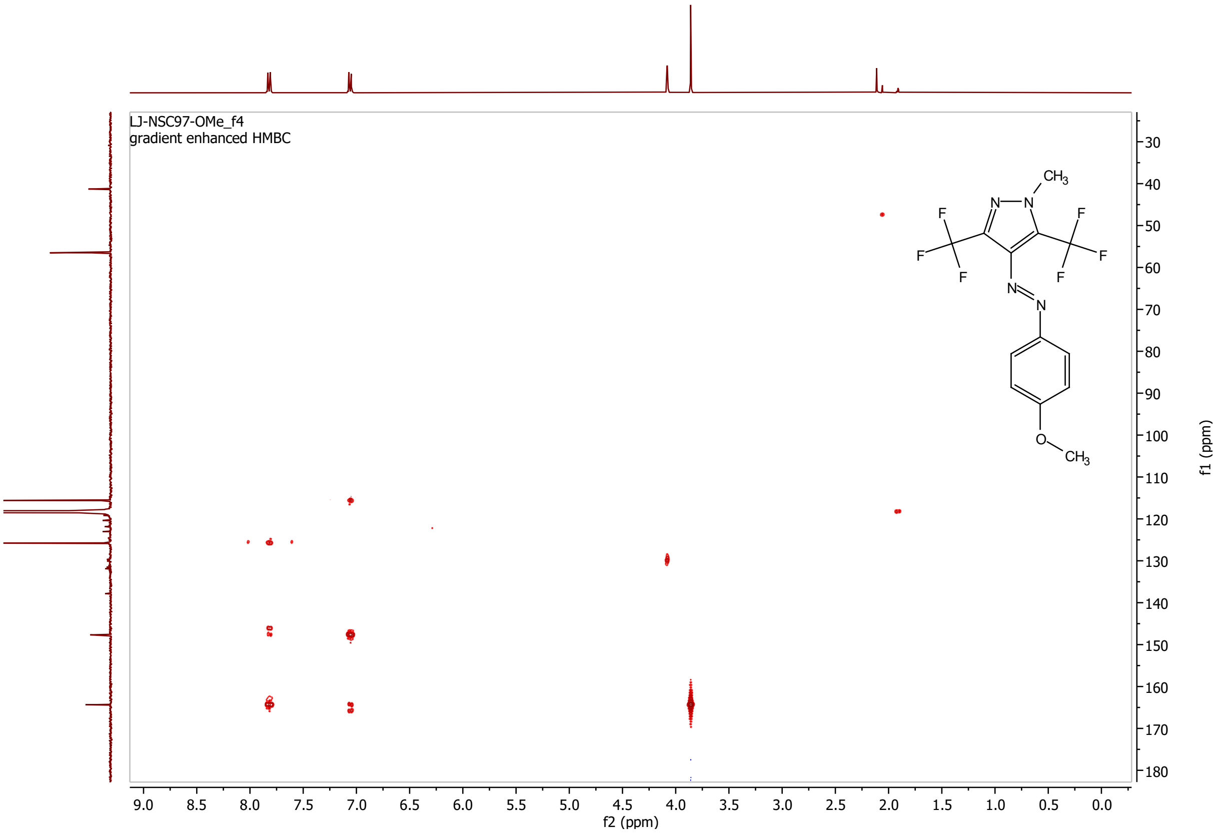

Figure S138: HMBC [$^{1}H \leftrightarrow ^{13}C$, (399.78 MHz /100.54 MHz, CD_3CN, 295 K) of **F-PAP-OMe**.

F-PAP-OCF$_3$: (*E*)-1-Methyl-4-((4-(trifluoromethoxy)phenyl)diazenyl)-3,5-bis(trifluoromethyl)-1*H*-pyrazole

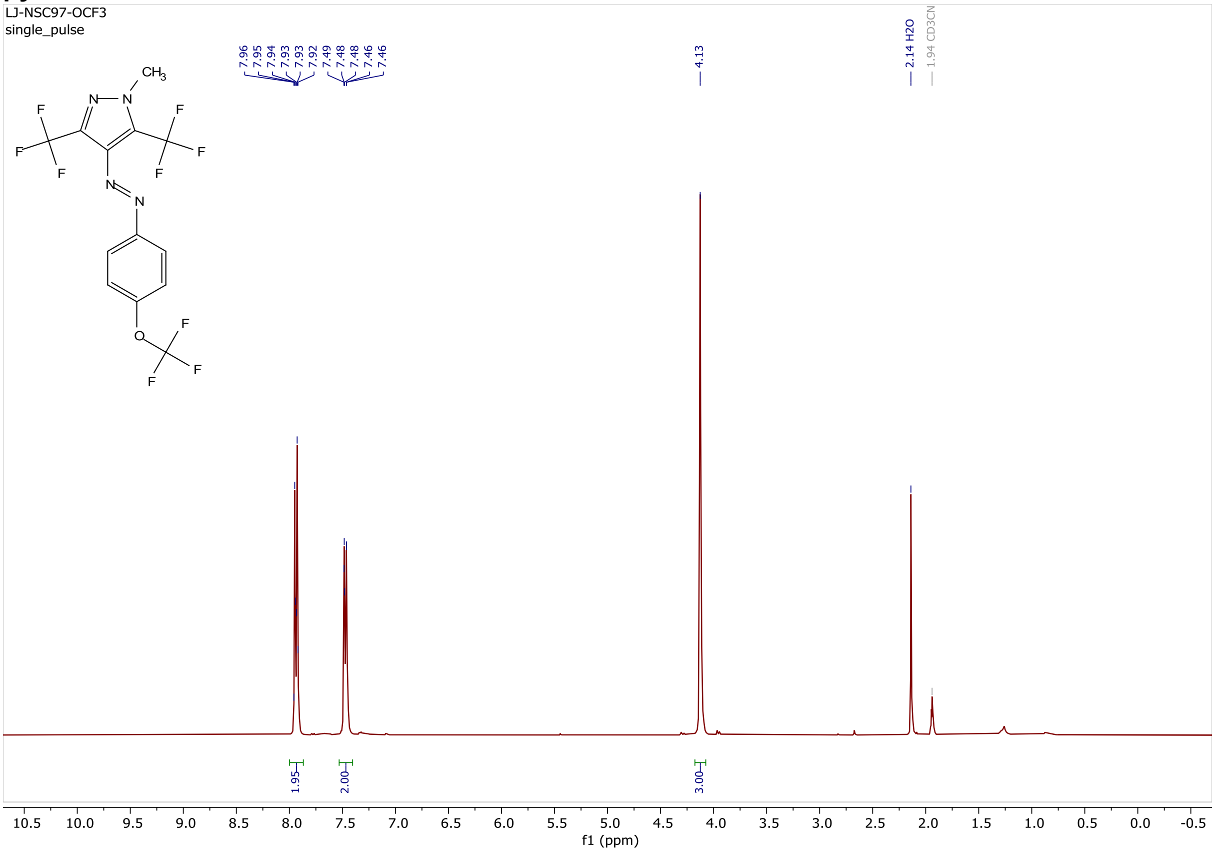

Figure S139: ^{1}H-NMR (399.78 MHz, CD_3CN, 295 K) of **F-PAP-OCF$_3$**.

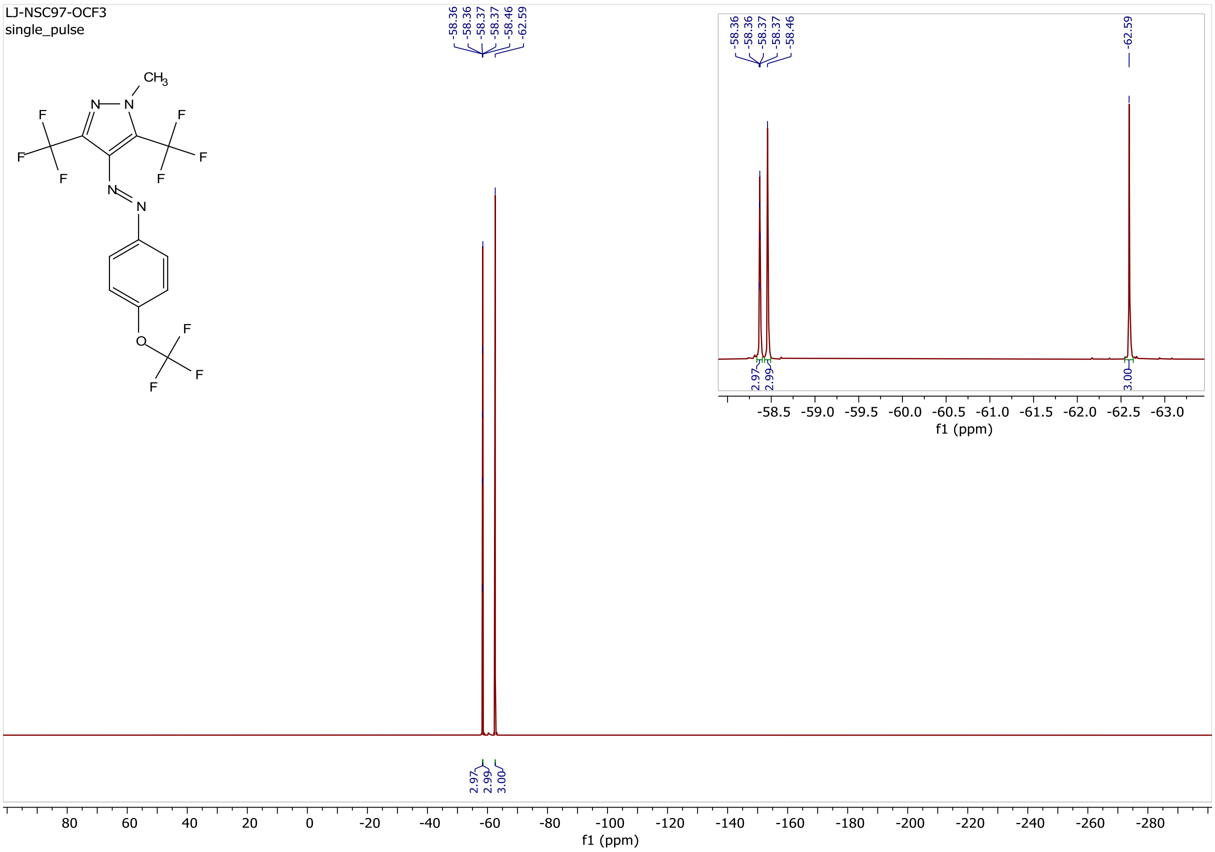

Figure S140: ^{19}F-NMR (376.13 MHz, CD_3CN, 295 K) of **F-PAP-OCF$_3$**.

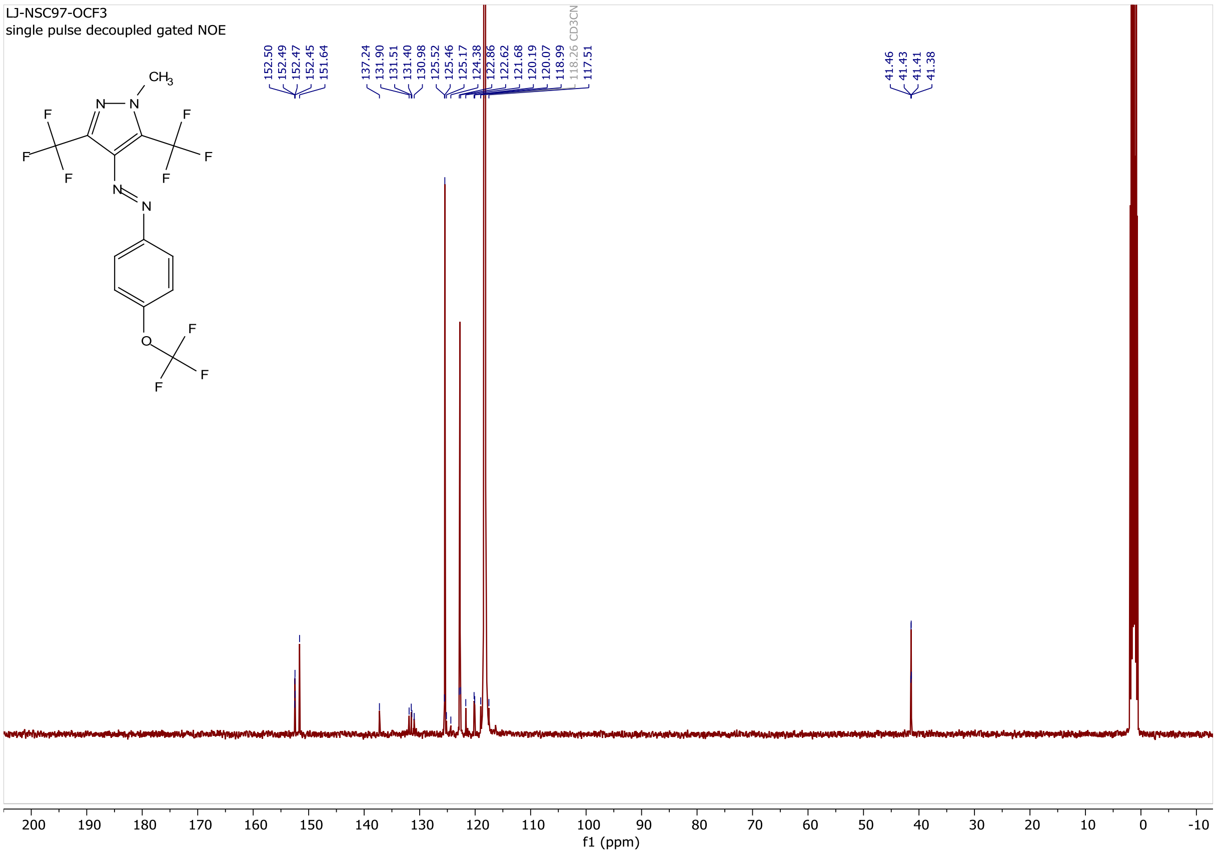

Figure S141: ^{13}C-NMR {^{1}H} (100.54 MHz, CD_3CN, 295 K) of **F-PAP-OCF$_3$**.

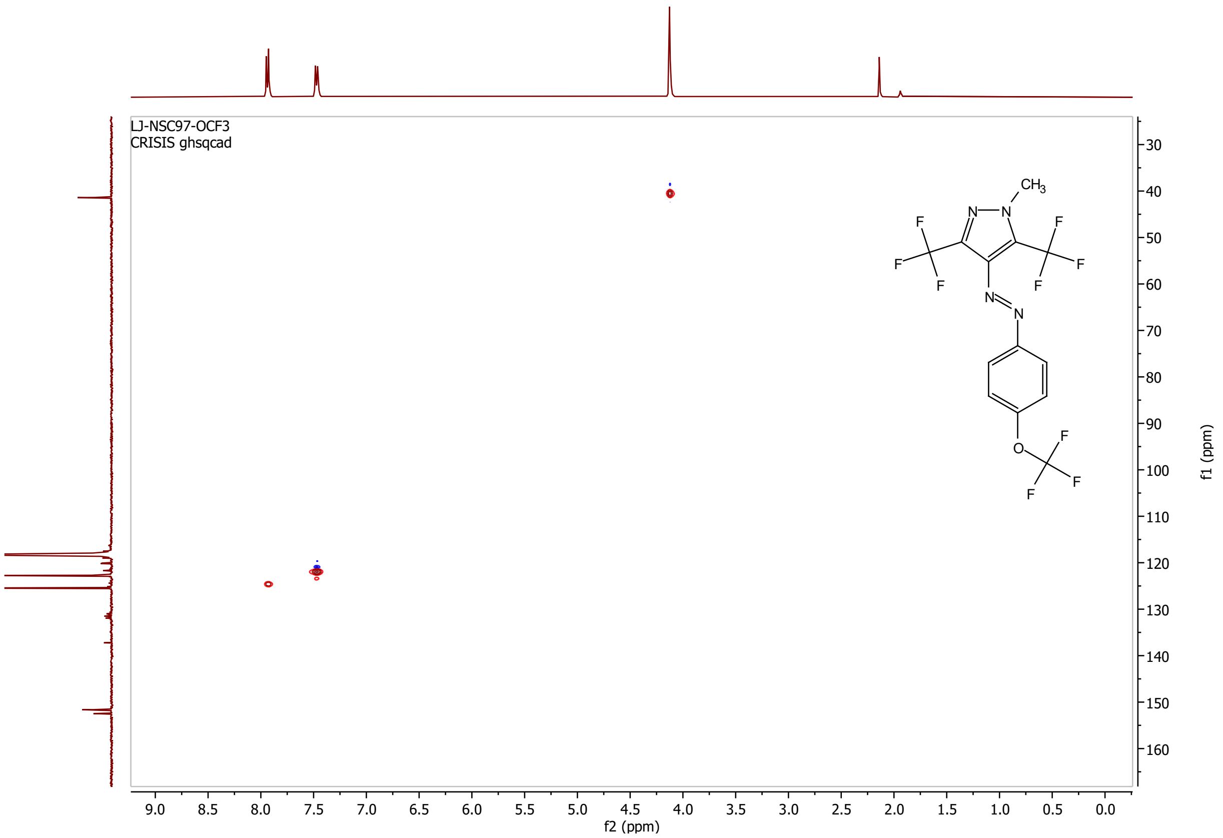

Figure S142: HSQC [^{1}H ↔^{13}C, (399.78 MHz /100.54 MHz, CD_3CN, 295 K) of **F-PAP-OCF$_3$**.

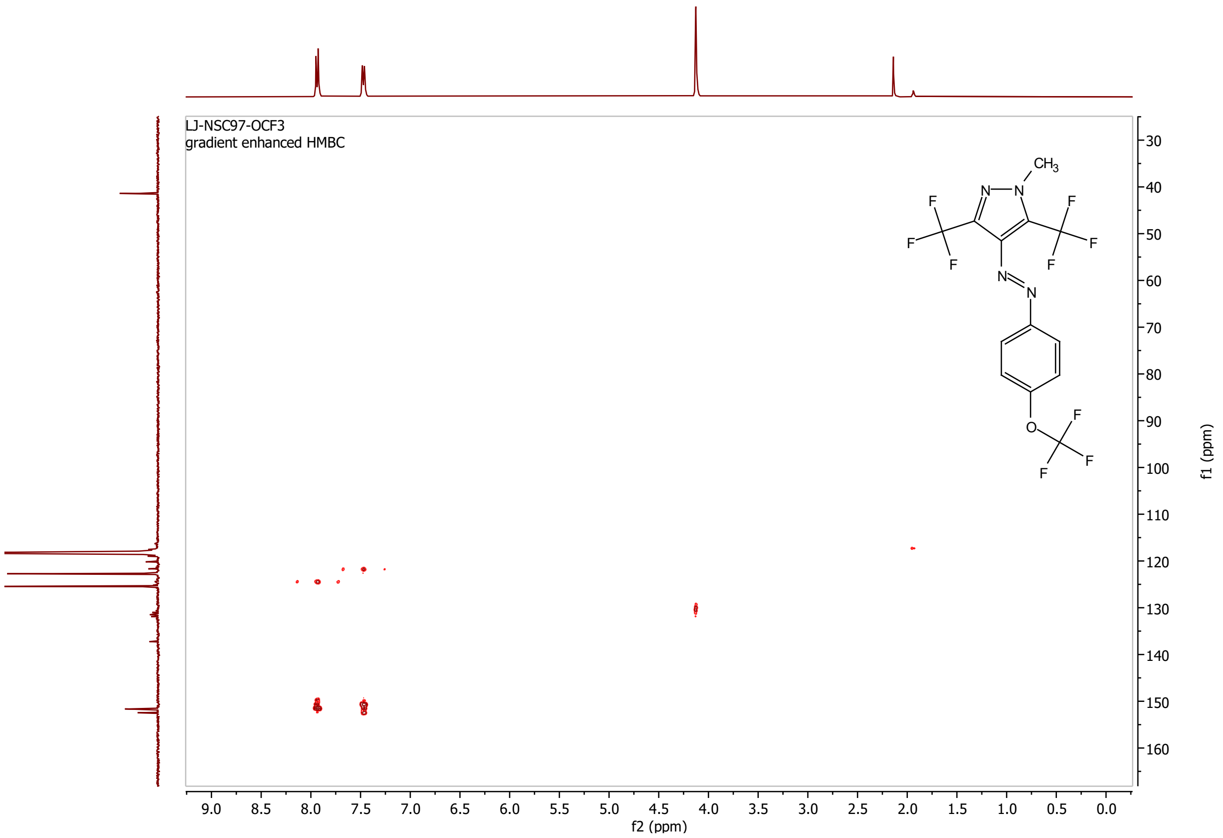

Figure S143: HMBC [^{1}H ↔ ^{13}C, (399.78 MHz /100.54 MHz, CD_3CN, 295 K) of **F-PAP-OCF$_3$**.

F-PAP-CCH: (*E*)-4-((4-Ethynylphenyl)diazenyl)-1-methyl-3,5-bis(trifluoromethyl)-1*H*-pyrazole

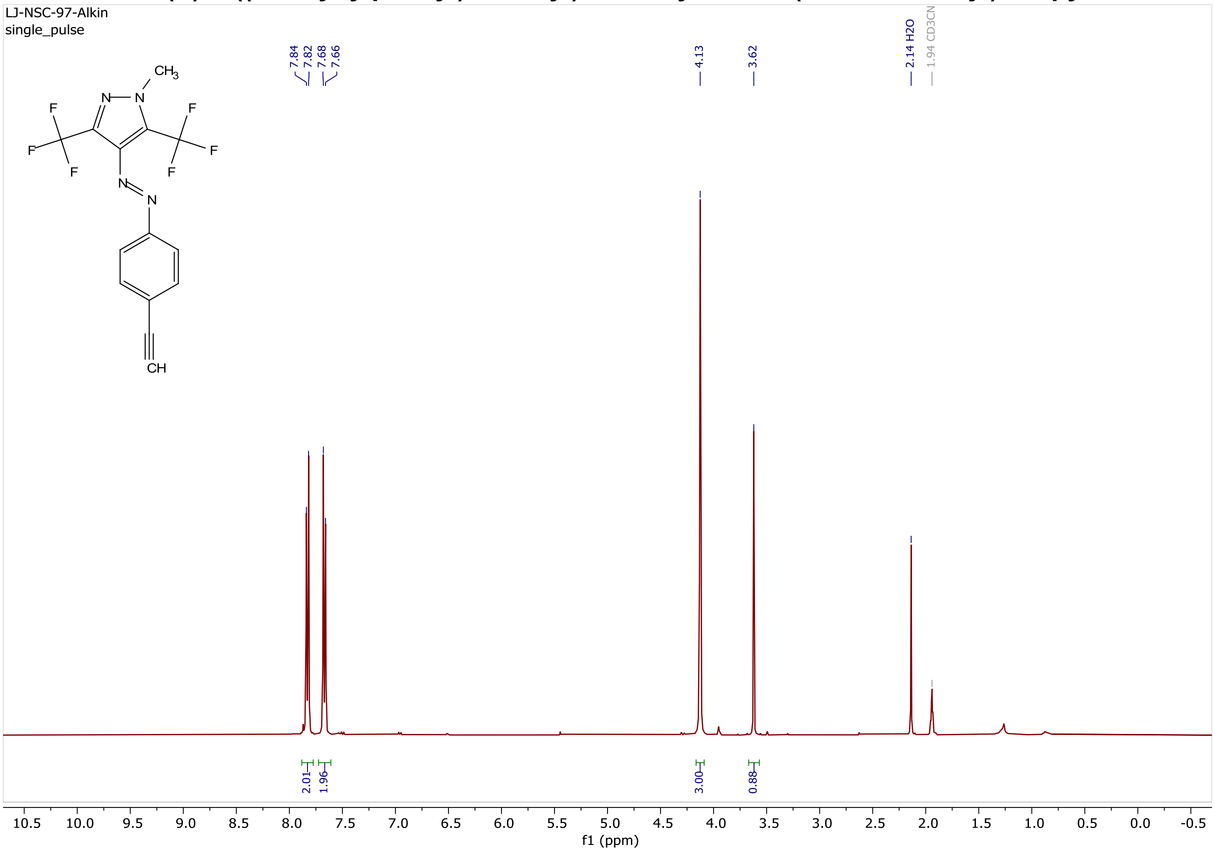

Figure S144: ^{1}H-NMR (399.78 MHz, CD_3CN, 295 K) of F-PAP-CCH.

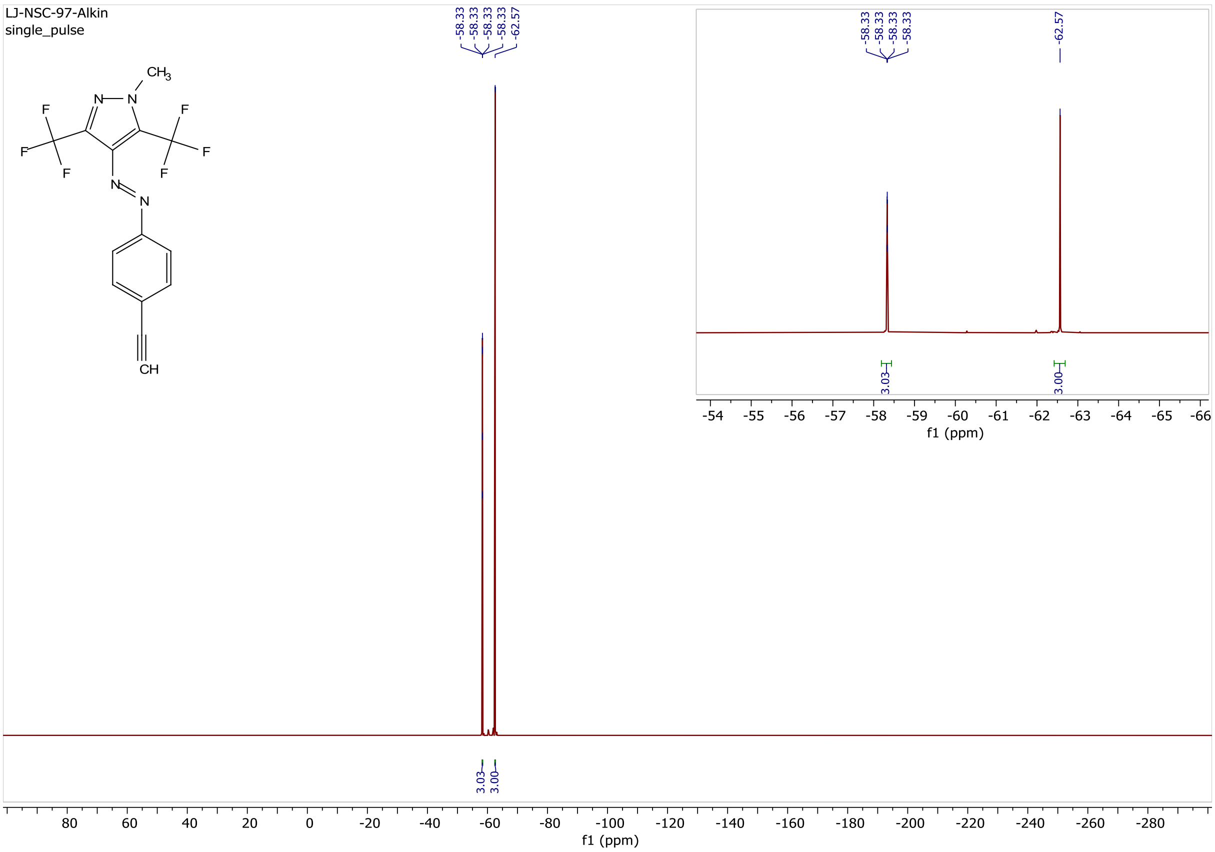

Figure S145: ^{19}F-NMR (376.13 MHz, CD_3CN, 295 K) of F-PAP-CCH.

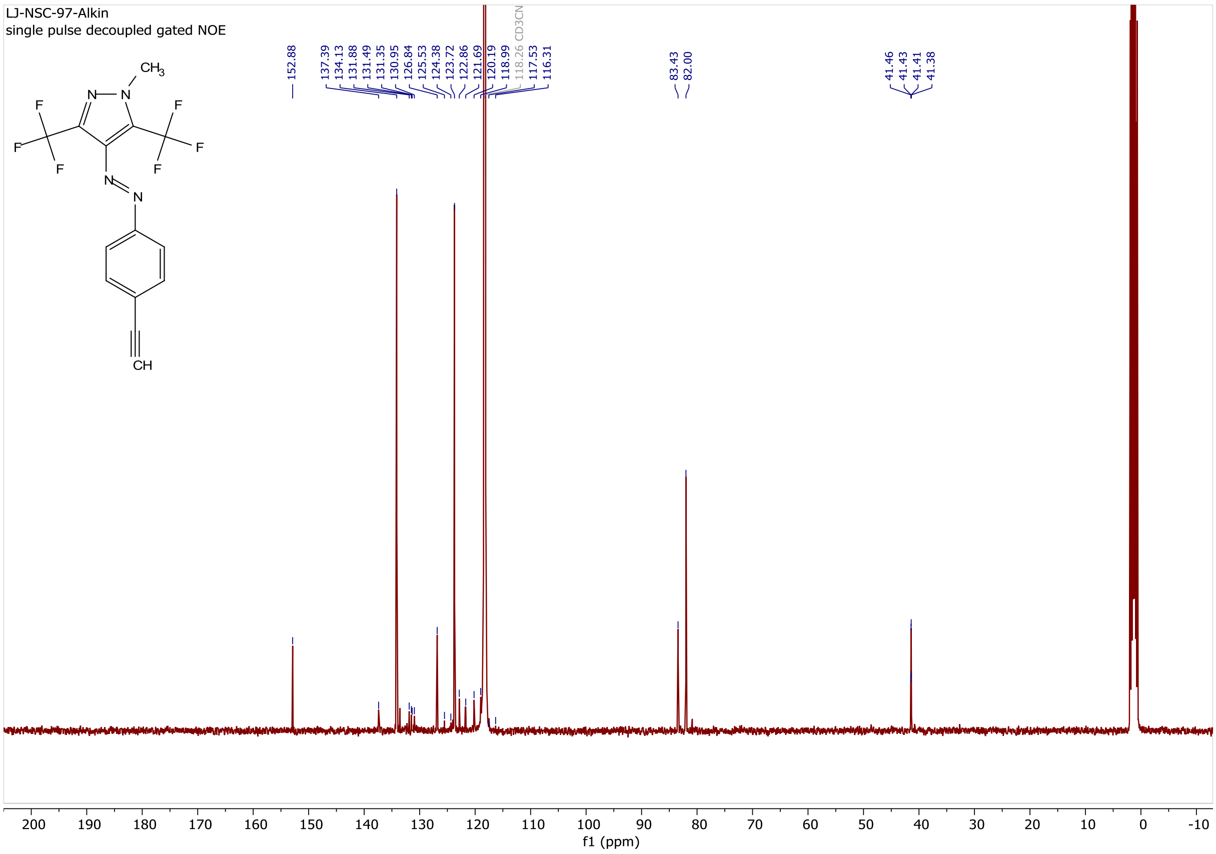

Figure S146: ^{13}C-NMR {^{1}H} (100.54 MHz, CD_3CN, 295 K) of F-PAP-CCH

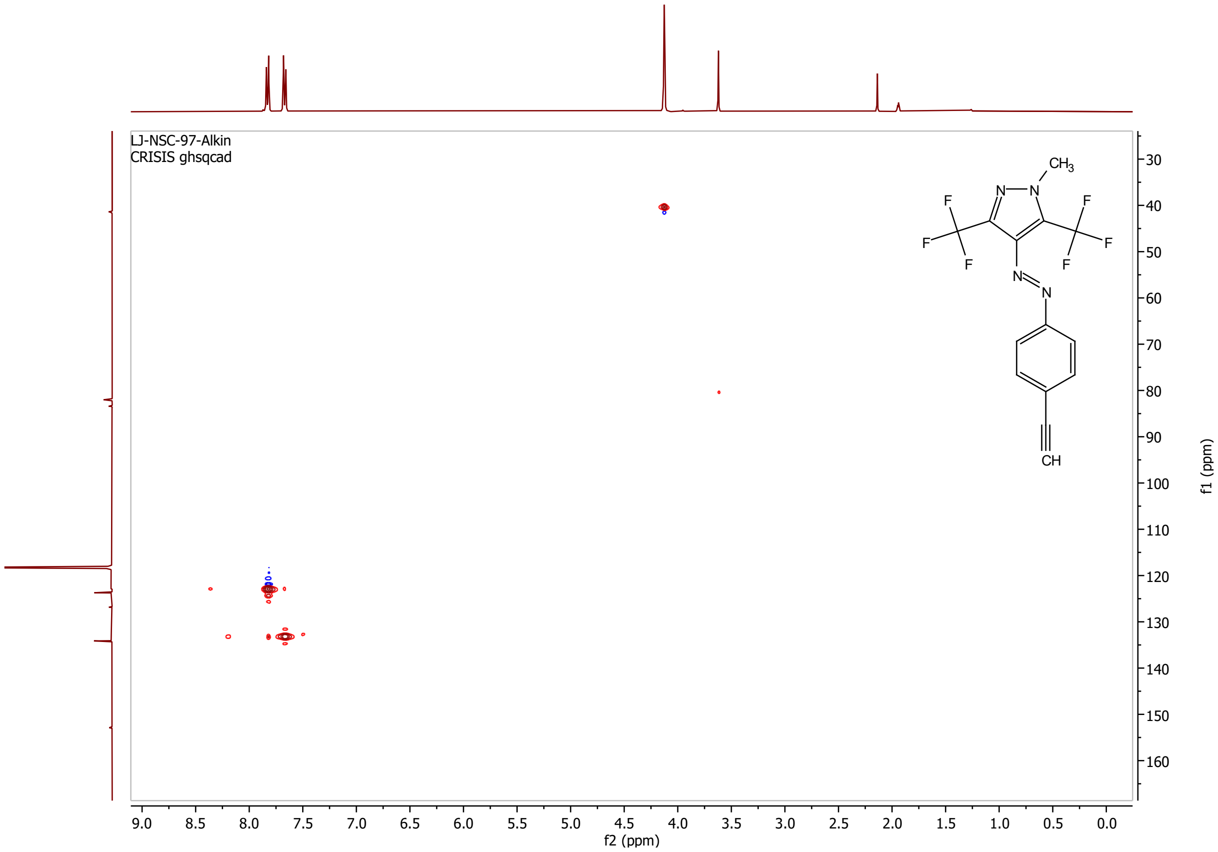

Figure S147: HSQC [$^{1}H \leftrightarrow ^{13}C$, (399.78 MHz /100.54 MHz, CD_3CN, 295 K) of F-PAP-CCH.

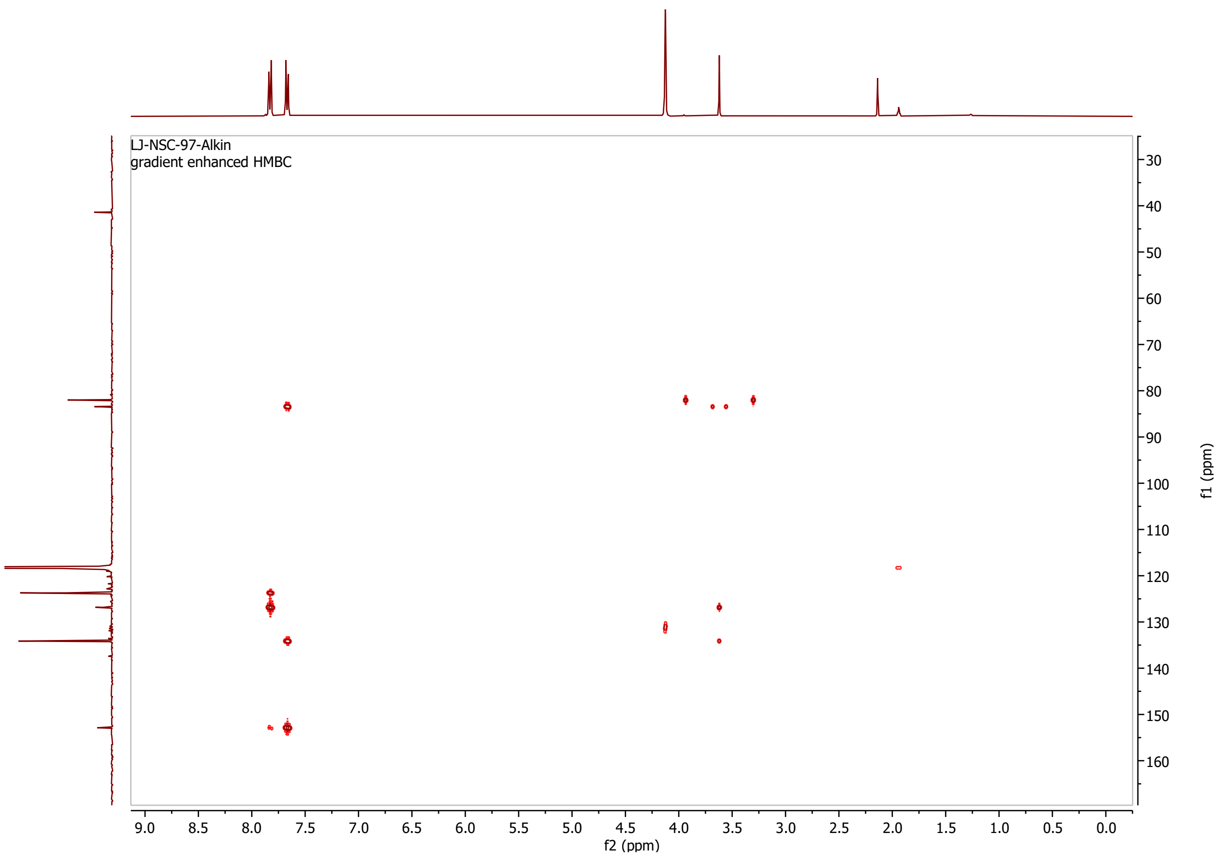

Figure S148: HMBC [$^{1}H \leftrightarrow {}^{13}C$, (399.78 MHz /100.54 MHz, CD_3CN, 295 K) of F-PAP-CCH.

F-PAP-*n*Bu: (*E*)-4-((4-Butylphenyl)diazenyl)-1-methyl-3,5-bis(trifluoromethyl)-1*H*-pyrazole

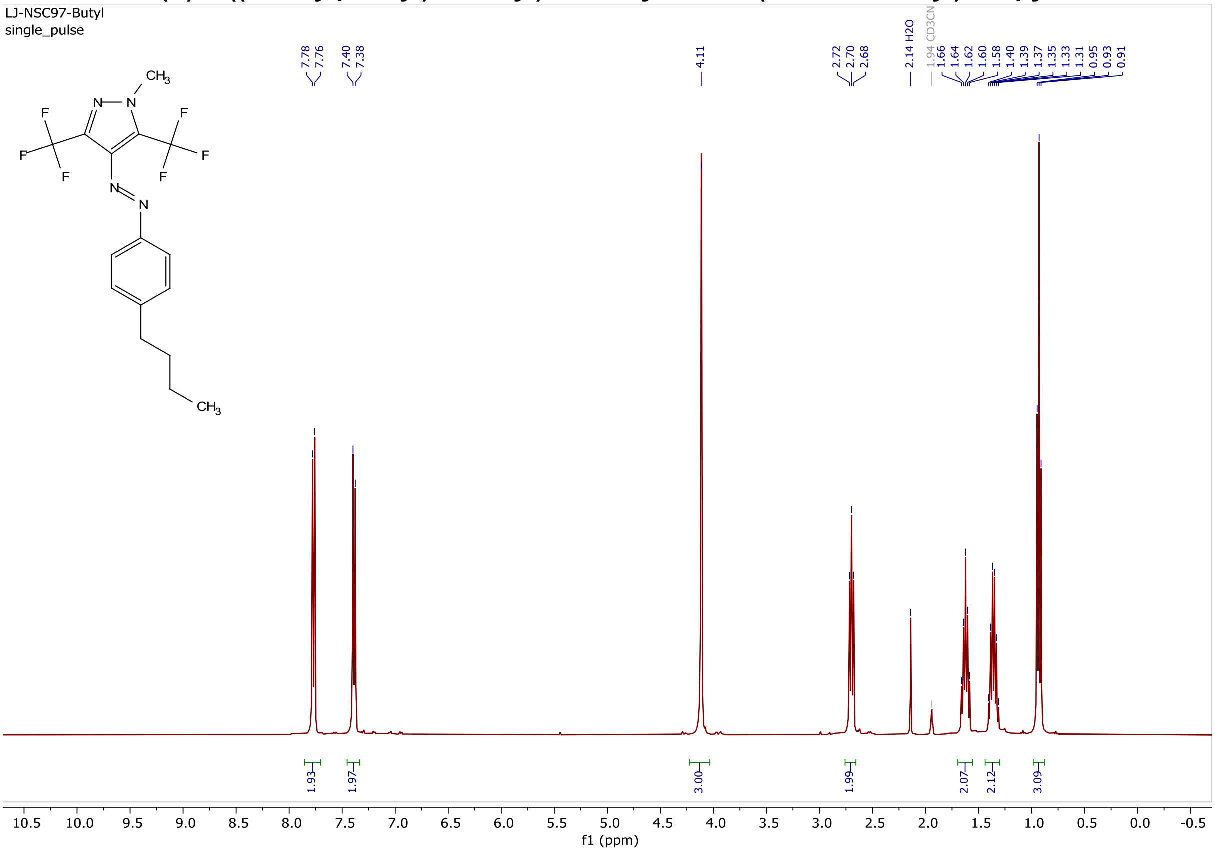

Figure S149: ^{1}H-NMR (399.78 MHz, CD_3CN, 295 K) of **F-PAP-*n*Bu**.

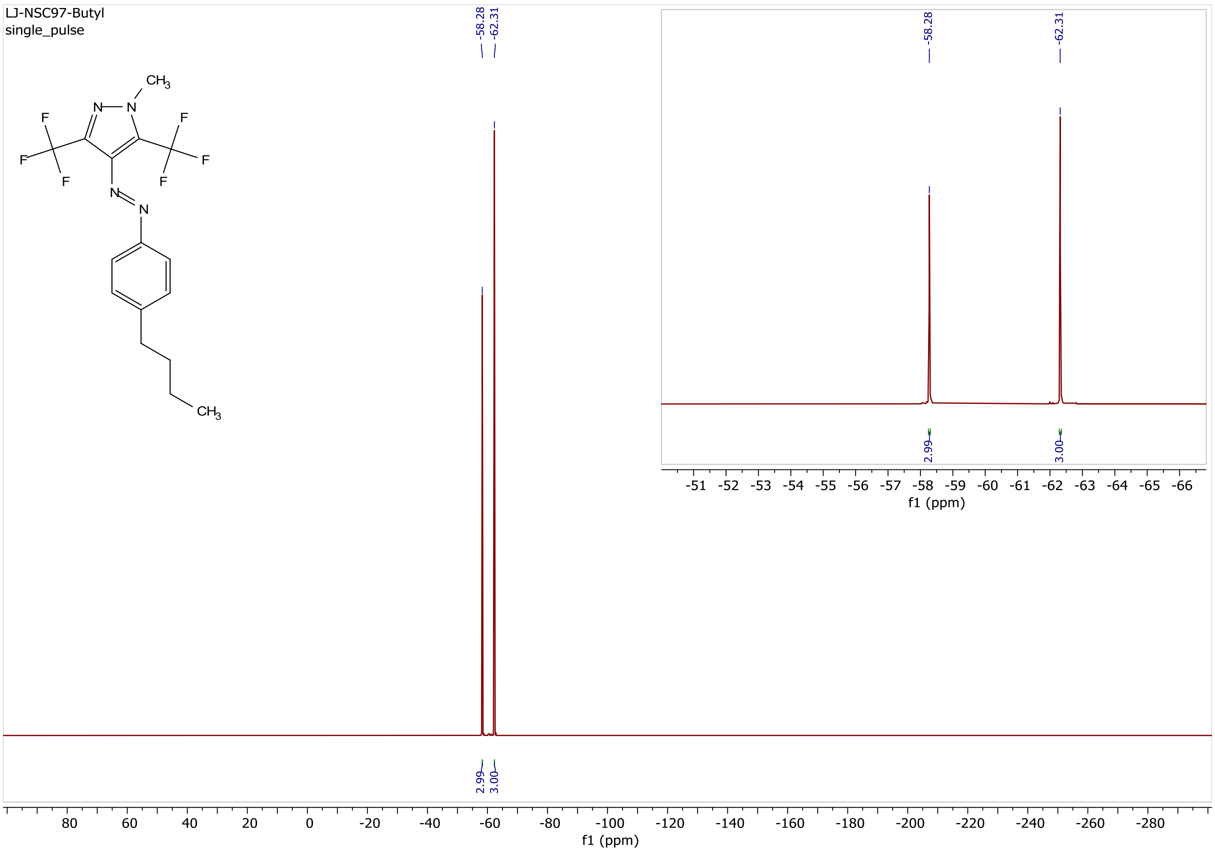

Figure S150: ^{19}F-NMR (376.13 MHz, CD_3CN, 295 K) of of **F-PAP-*n*Bu**.

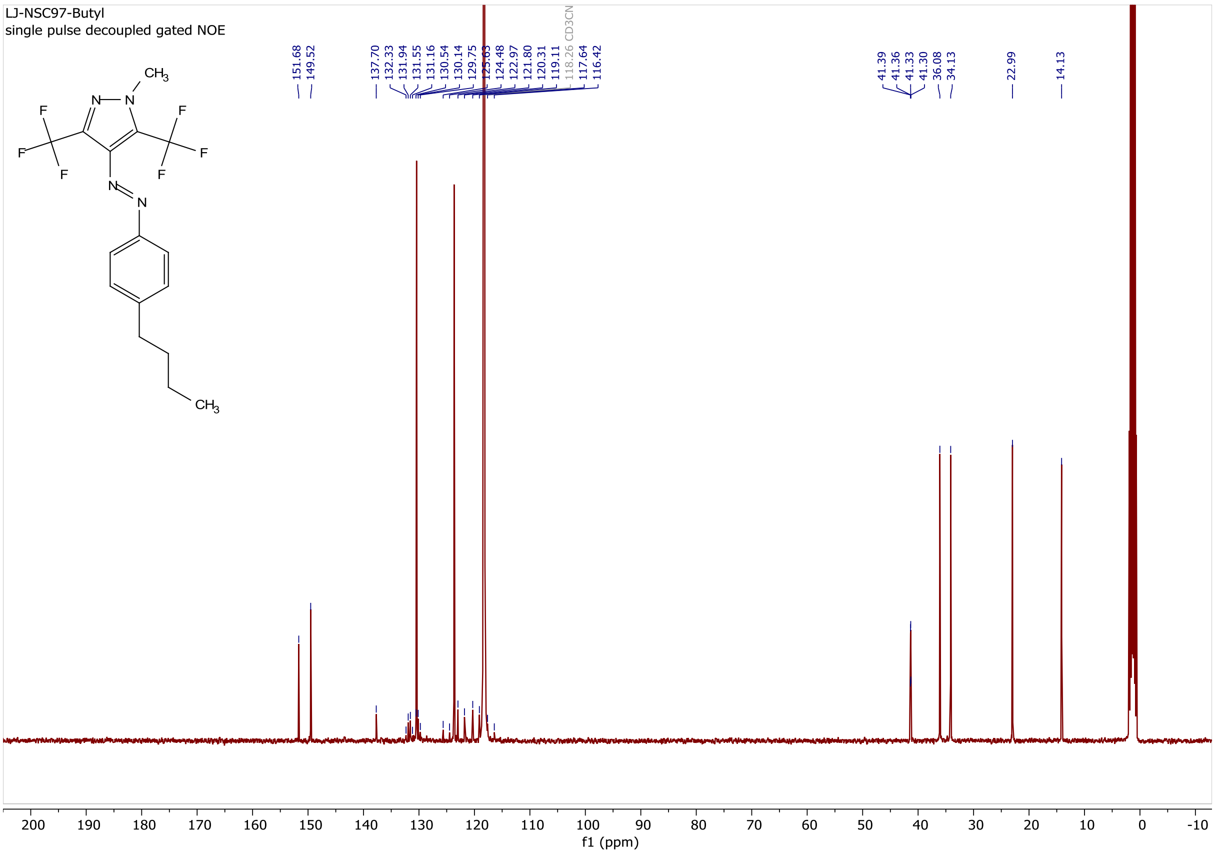

Figure S151: ^{13}C-NMR {^{1}H} (100.54 MHz, CD_3CN, 295 K) of of **F-PAP-*n*Bu**.

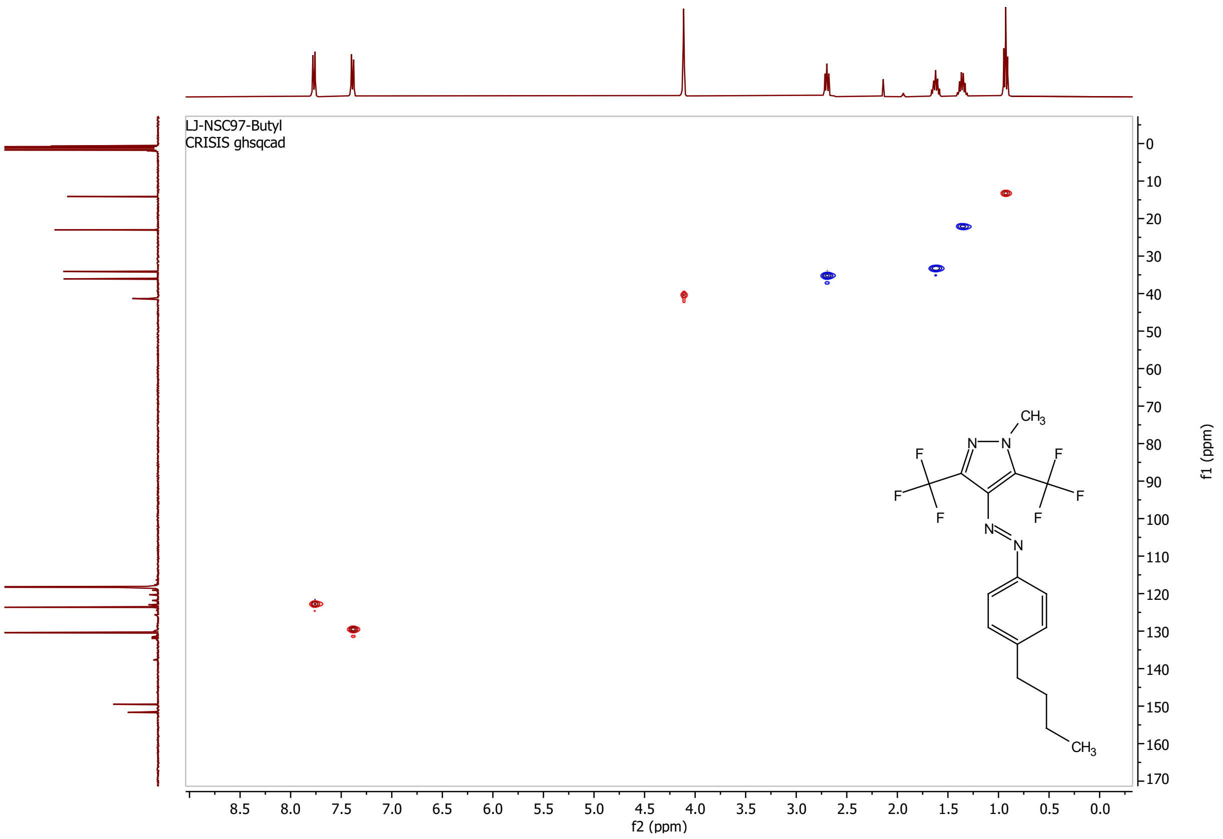

Figure S152: HSQC [$^{1}H \leftrightarrow ^{13}C$, (399.78 MHz /100.54 MHz, CD_3CN, 295 K) of of **F-PAP-*n*Bu**.

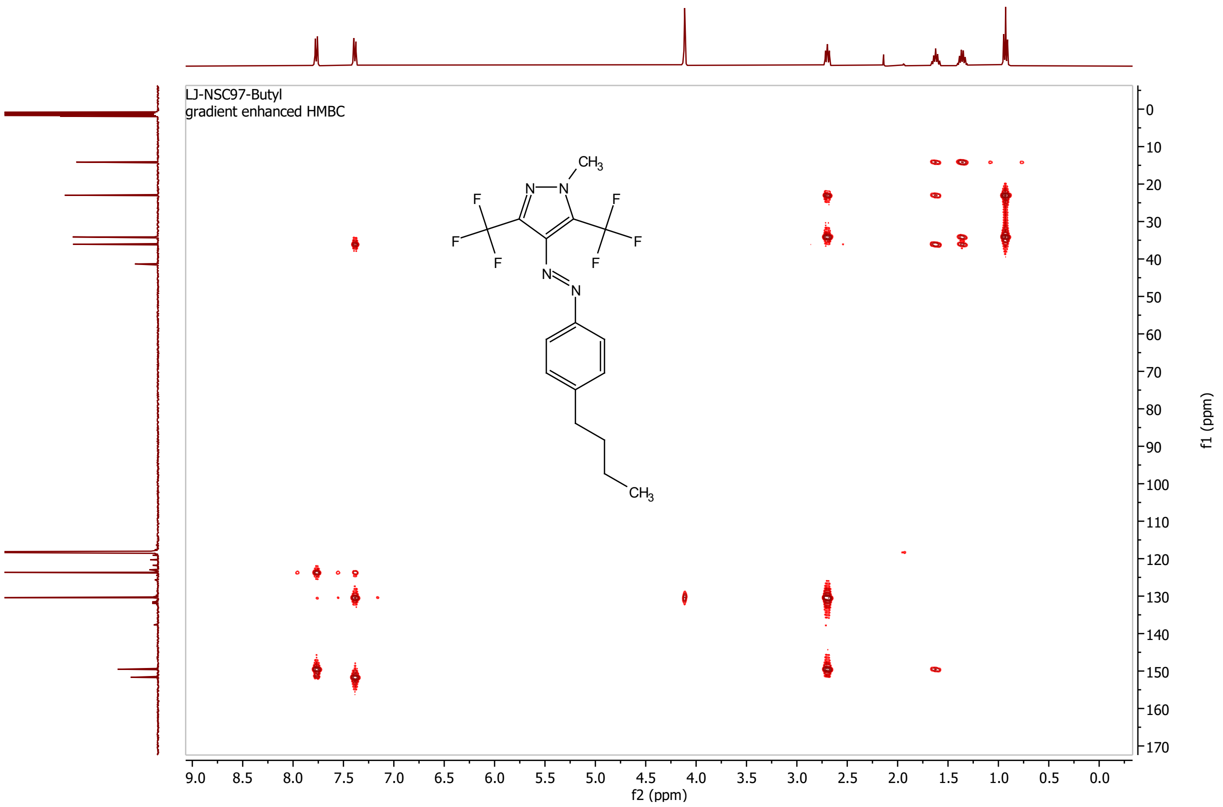

Figure S153: HMBC [$^{1}H \leftrightarrow ^{13}C$, (399.78 MHz /100.54 MHz, CD_3CN, 295 K) of of **F-PAP-*n*Bu**.

F-PAP-C$_3$H$_6$COOH: (*E*)-4-(4-((1-Methyl-3,5-bis(trifluoromethyl)-1*H*-pyrazol-4-yl)diazenyl)phenyl) butanoic acid

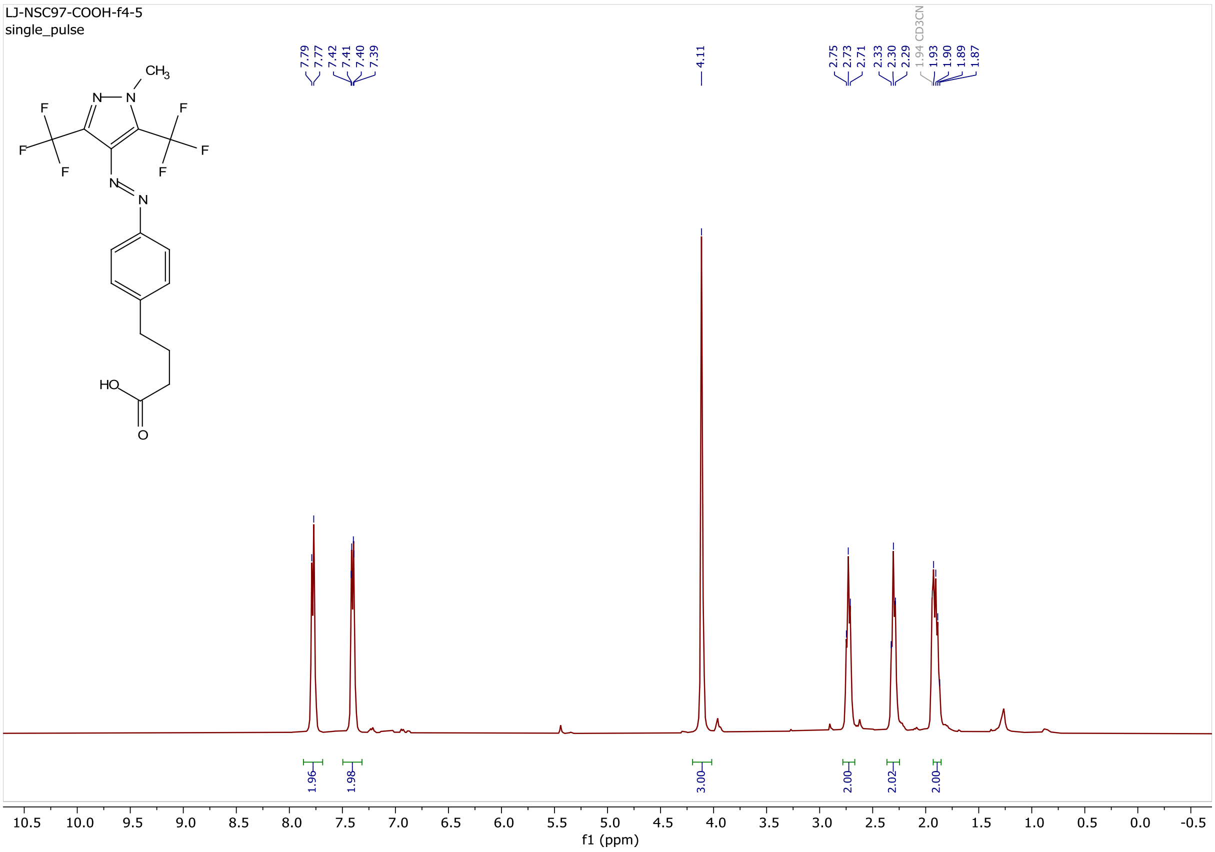

Figure S154: ^{1}H-NMR (399.78 MHz, CD_3CN, 295 K) of **F-PAP-C$_3$H$_6$COOH**.

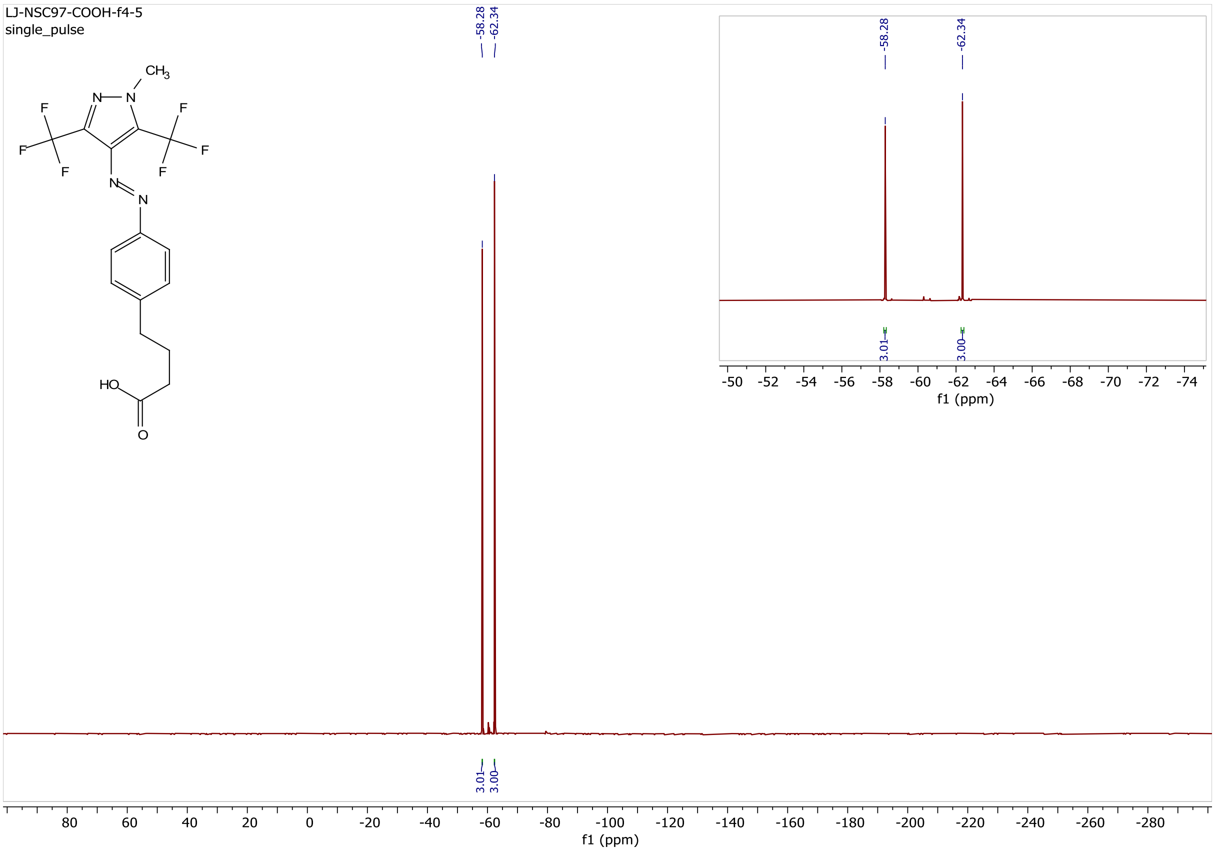

Figure S155: ^{19}F-NMR (376.13 MHz, CD_3CN, 295 K) of **F-PAP-C$_3$H$_6$COOH**.

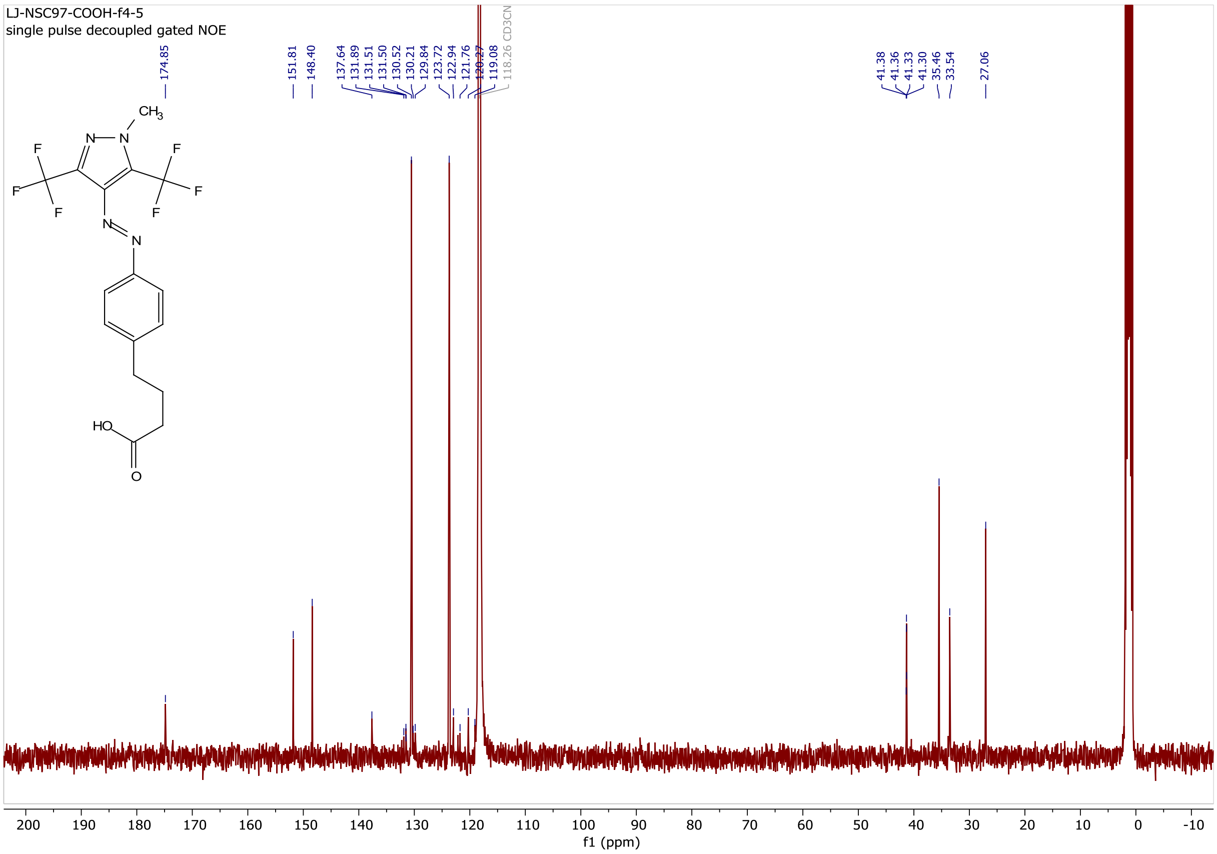

Figure S156: ^{13}C-NMR {^{1}H} (100.54 MHz, CD_3CN, 295 K) of

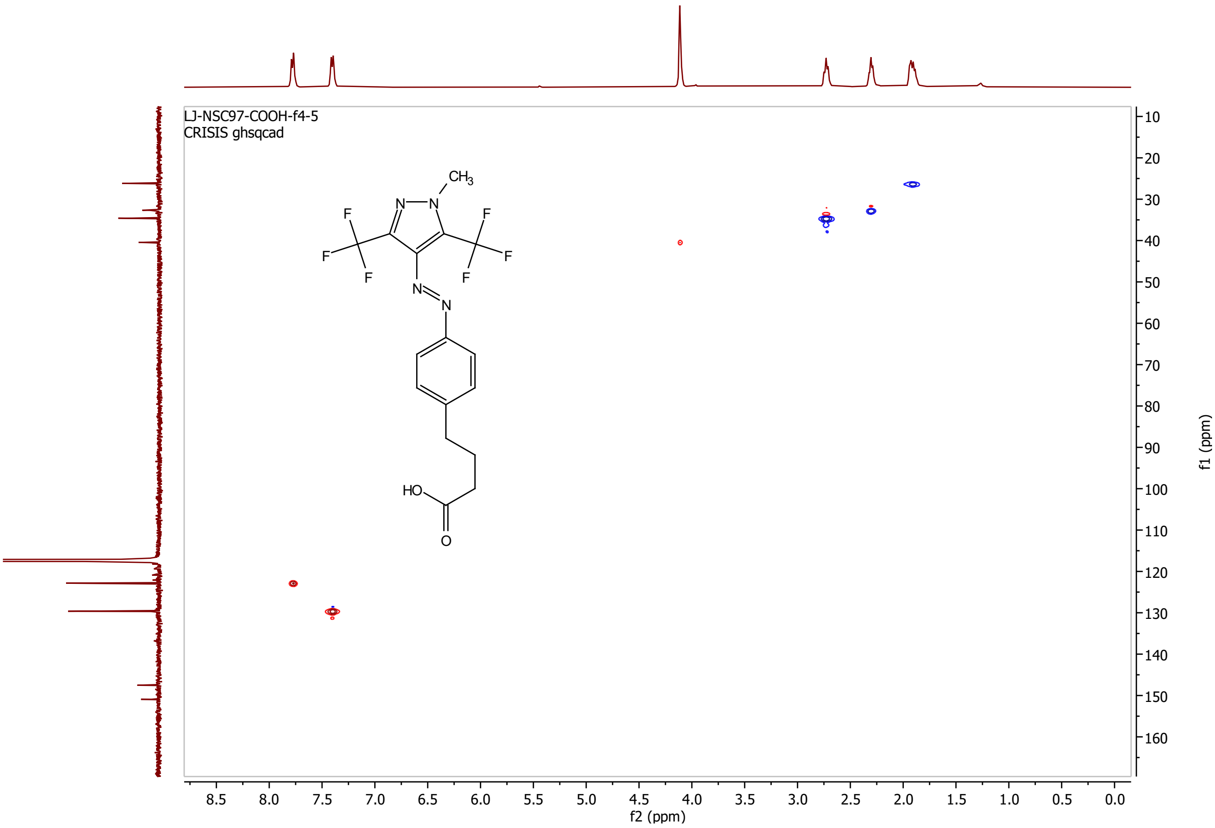

Figure S157: HSQC [$^{1}H \leftrightarrow ^{13}C$, (399.78 MHz /100.54 MHz, CD_3CN, 295 K) of **F-PAP-C_3H_6COOH**.

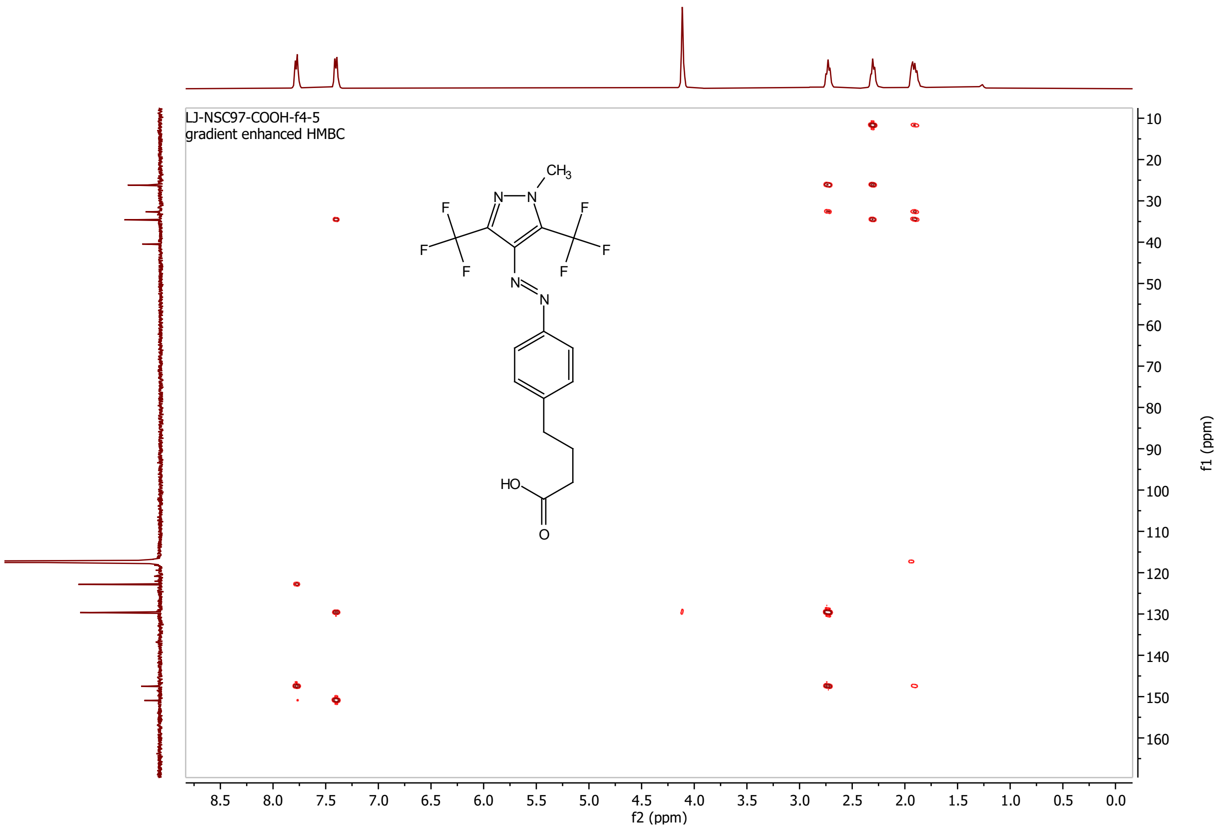

Figure S158: HMBC [$^{1}H \leftrightarrow ^{13}C$, (399.78 MHz /100.54 MHz, CD_3CN, 295 K) of **F-PAP-C_3H_6COOH**.

F-PAP-NO$_2$: (*E*)-1-Methyl-4-((4-nitrophenyl)diazenyl)-3,5-bis(trifluoromethyl)-1*H*-pyrazole

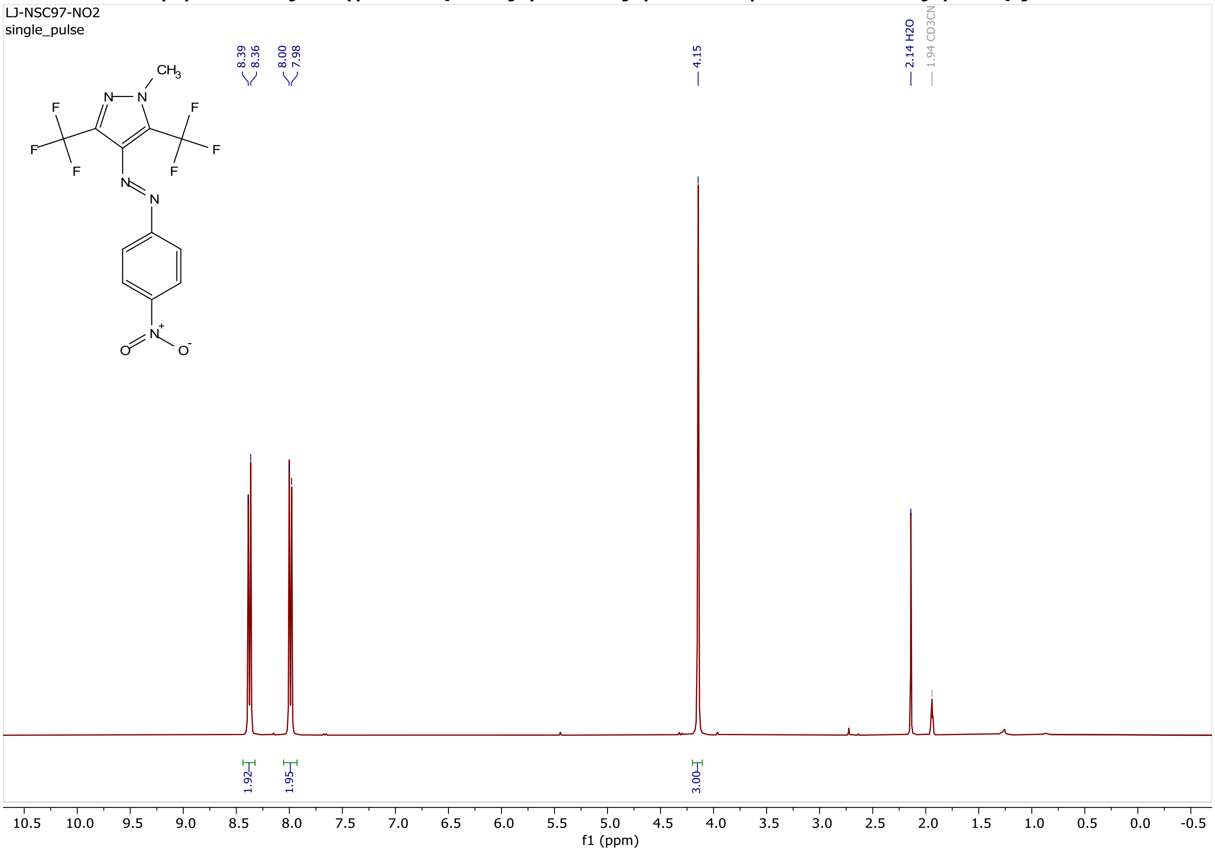

Figure S159: ^{1}H-NMR (399.78 MHz, CD_3CN, 295 K) of **F-PAP-NO$_2$**.

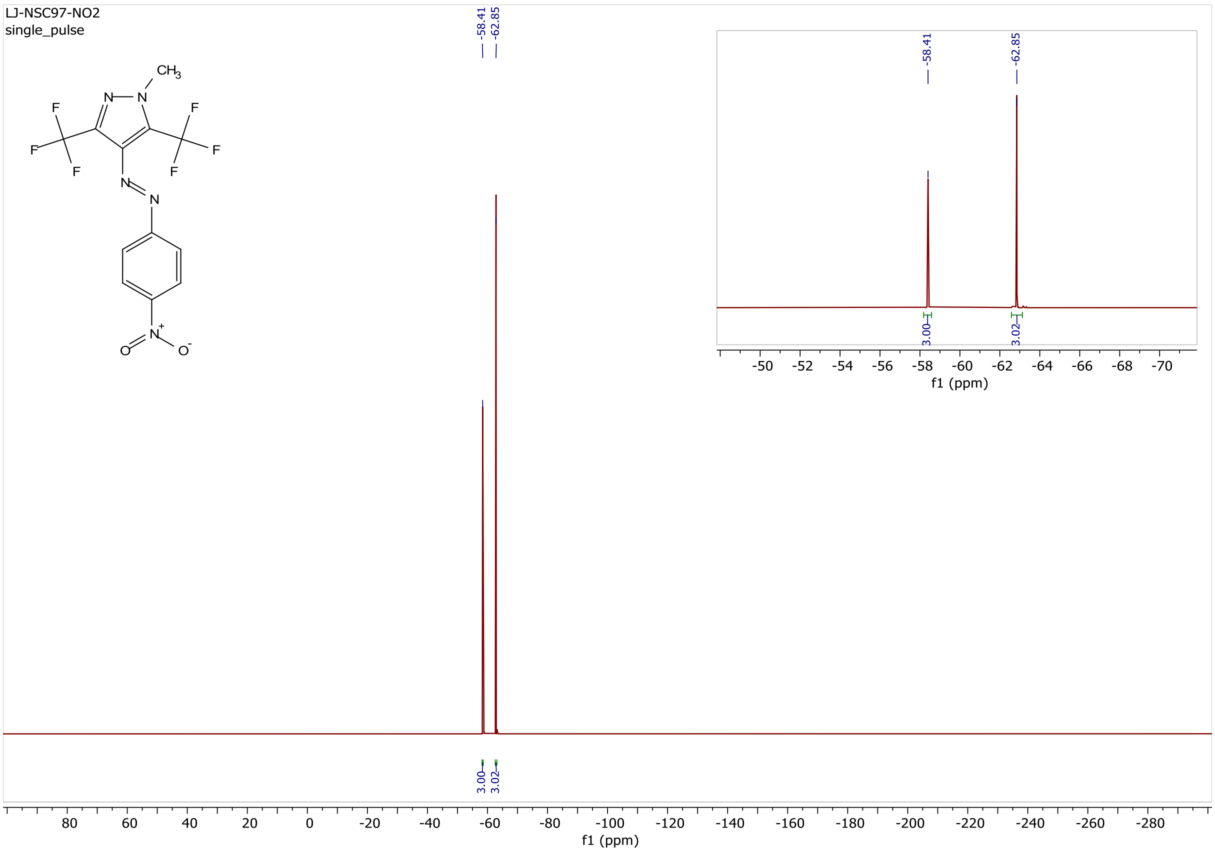

Figure S160: ^{19}F-NMR (376.13 MHz, CD_3CN, 295 K) of **F-PAP-NO$_2$**.

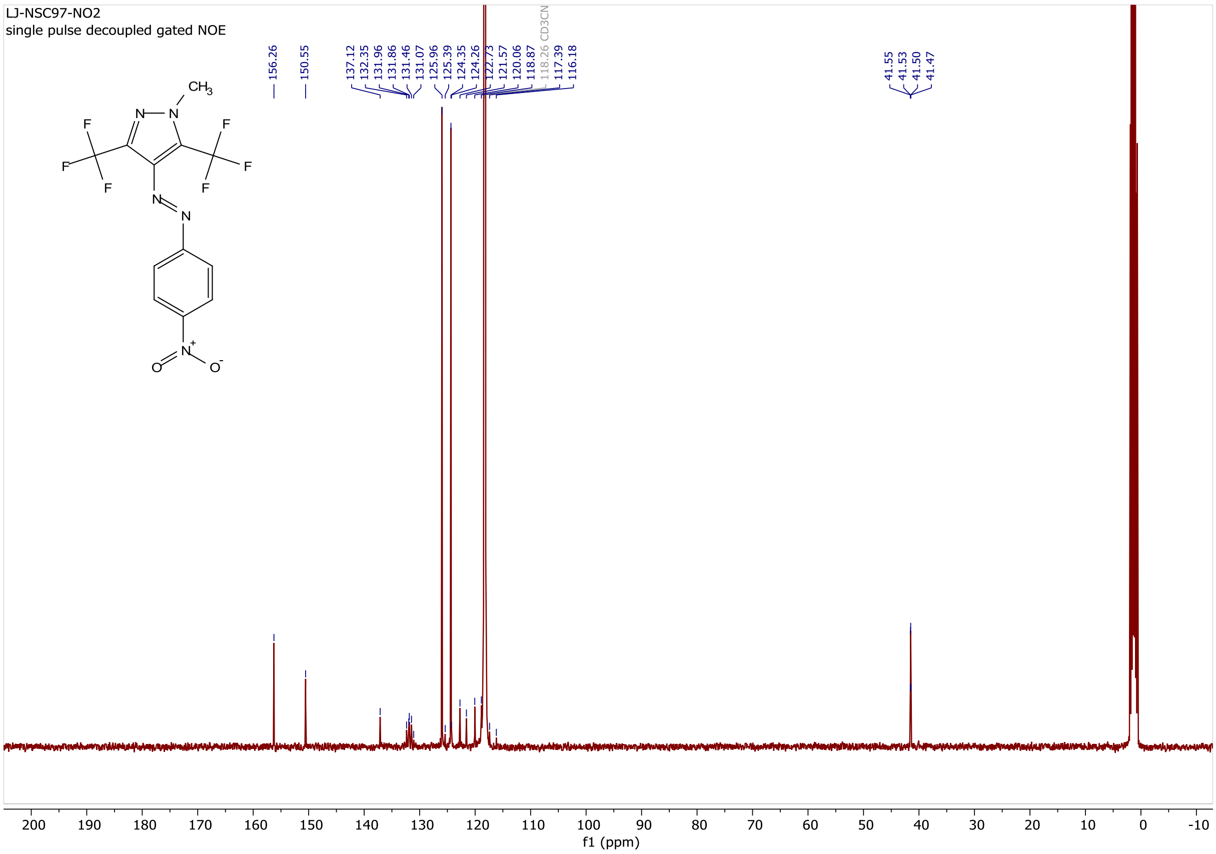

Figure S161: ^{13}C-NMR {^{1}H} (100.54 MHz, CD_3CN, 295 K) of **F-PAP-NO$_2$**.

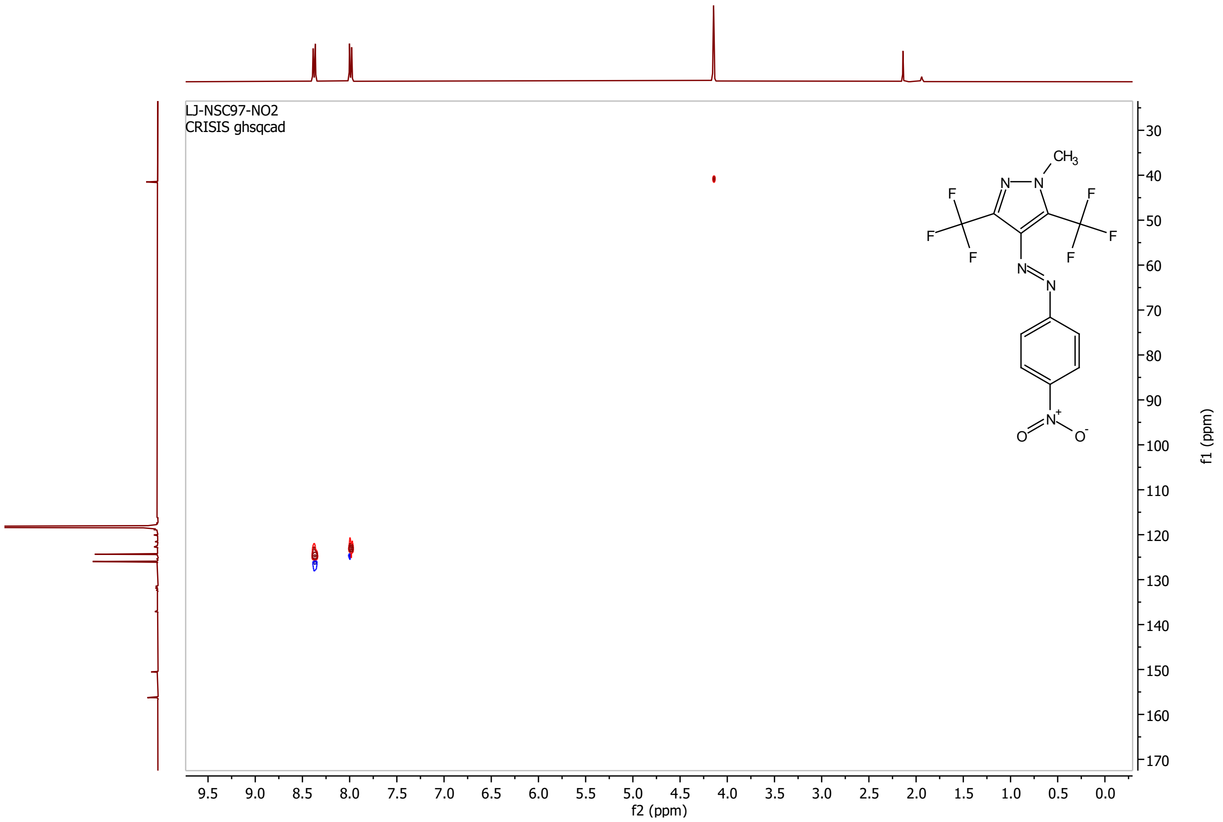

Figure S162: HSQC [$^{1}H \leftrightarrow ^{13}C$, (399.78 MHz /100.54 MHz, CD_3CN, 295 K) of **F-PAP-NO$_2$**.

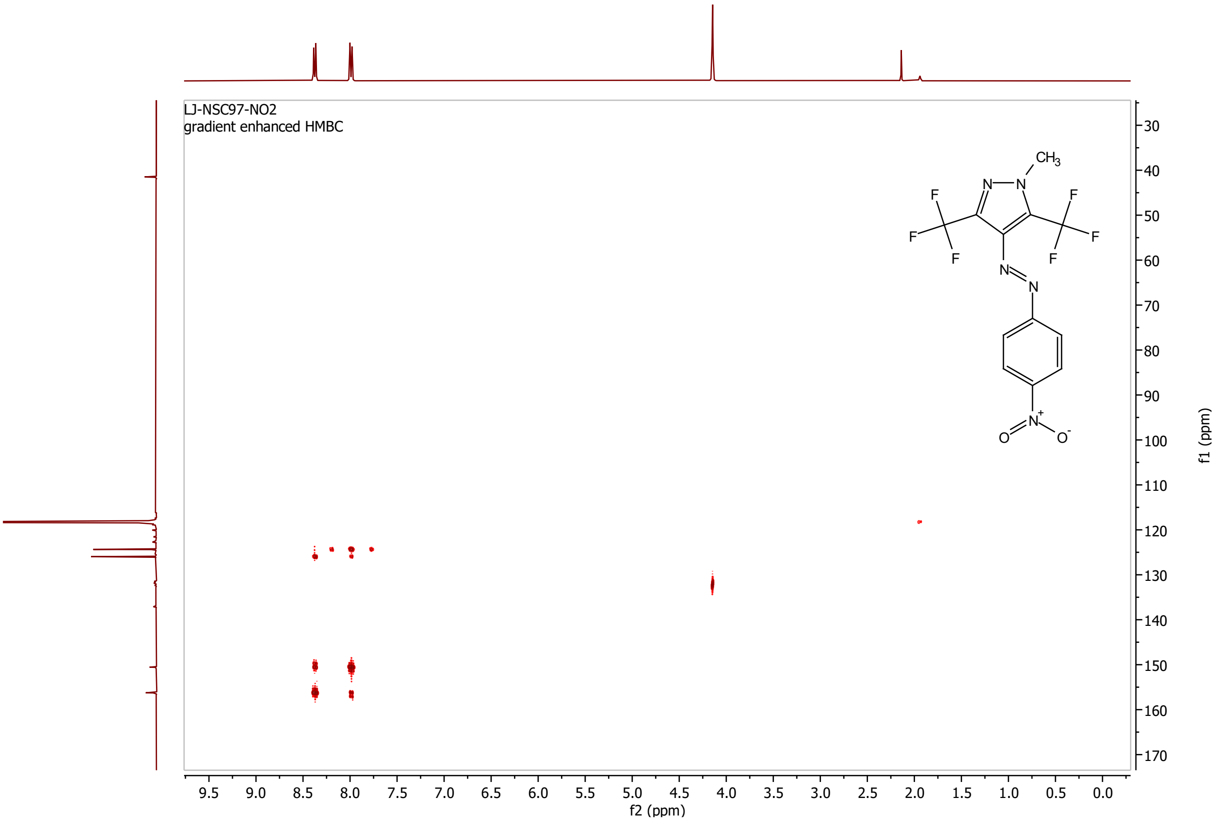

Figure S163: HMBC [$^{1}H \leftrightarrow ^{13}C$, (399.78 MHz /100.54 MHz, CD_3CN, 295 K) of **F-PAP-NO$_2$**.

F-(*N*Ph)PAP: (*E*)-1-Phenyl-4-(phenyldiazenyl)-3,5-bis(trifluoromethyl)-1*H*-pyrazole

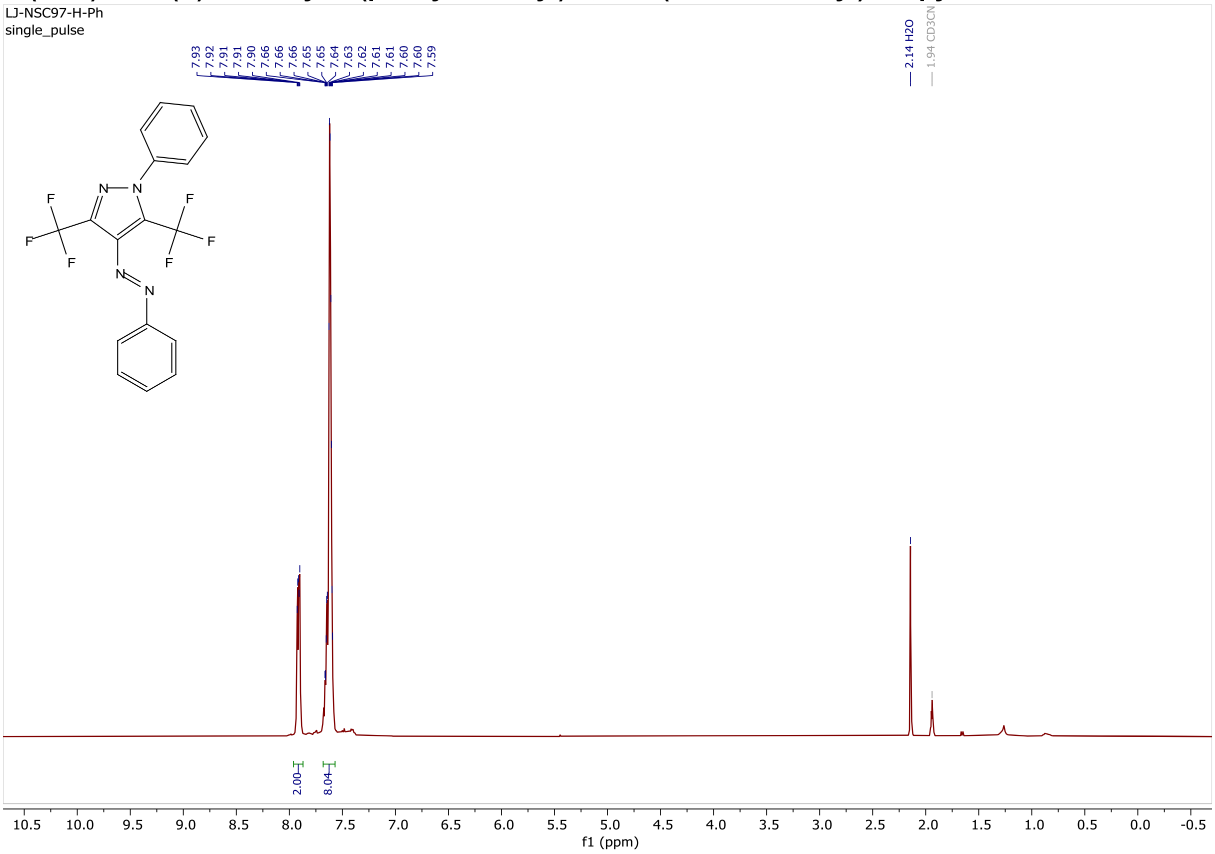

Figure S164: ^{1}H-NMR (399.78 MHz, CD_3CN, 295 K) of **F-(*N*Ph)PAP**.

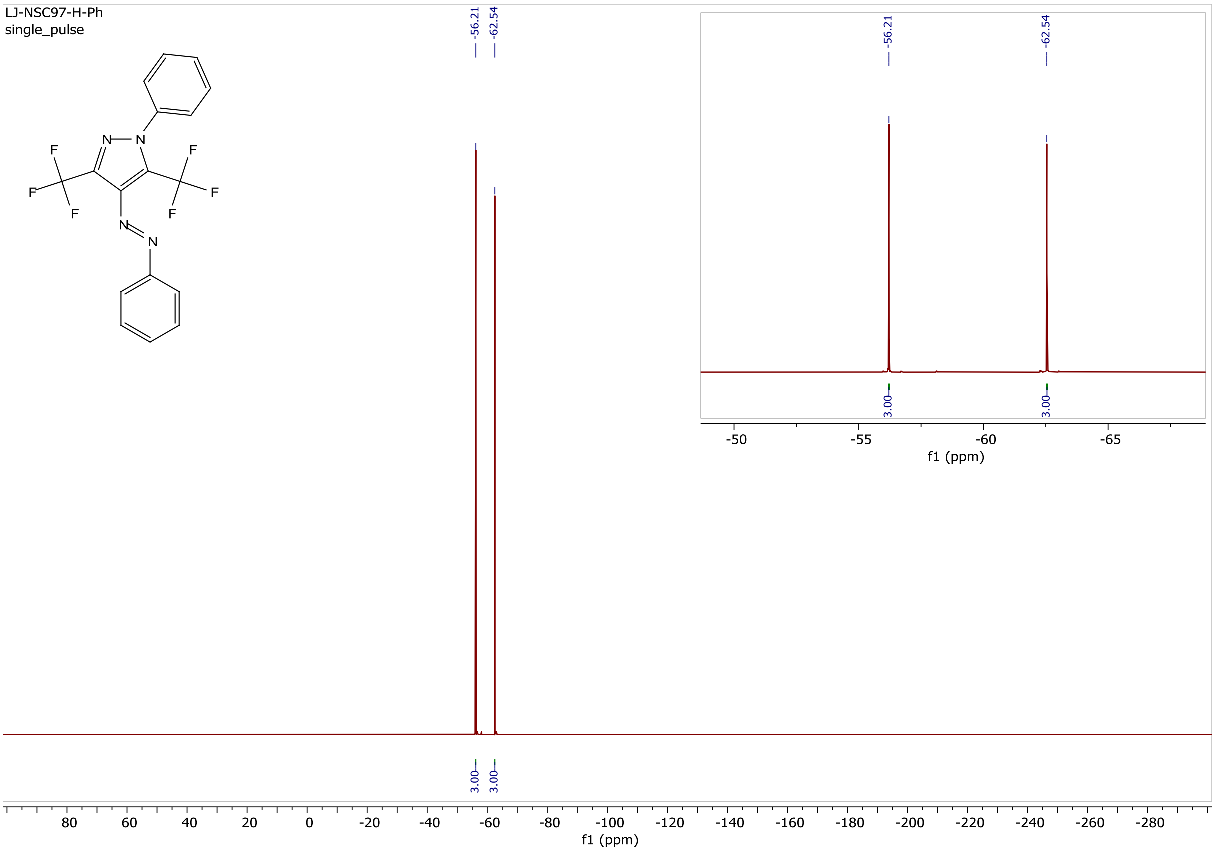

Figure S165: ^{19}F-NMR (376.13 MHz, CD_3CN, 295 K) of **F-(*N*Ph)PAP**.

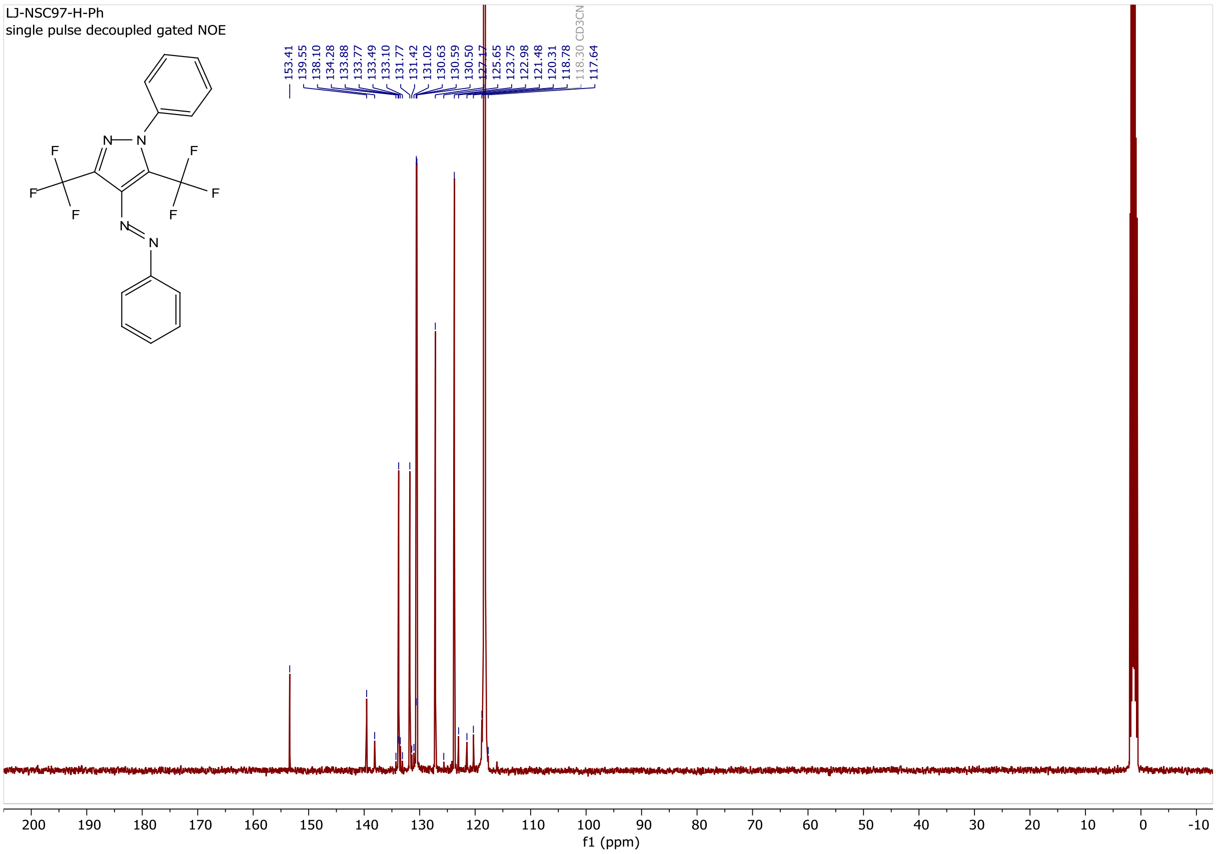

Figure S166: ^{13}C-NMR {^{1}H} (100.54 MHz, CD_3CN, 295 K) of **F-(*N*Ph)PAP**.

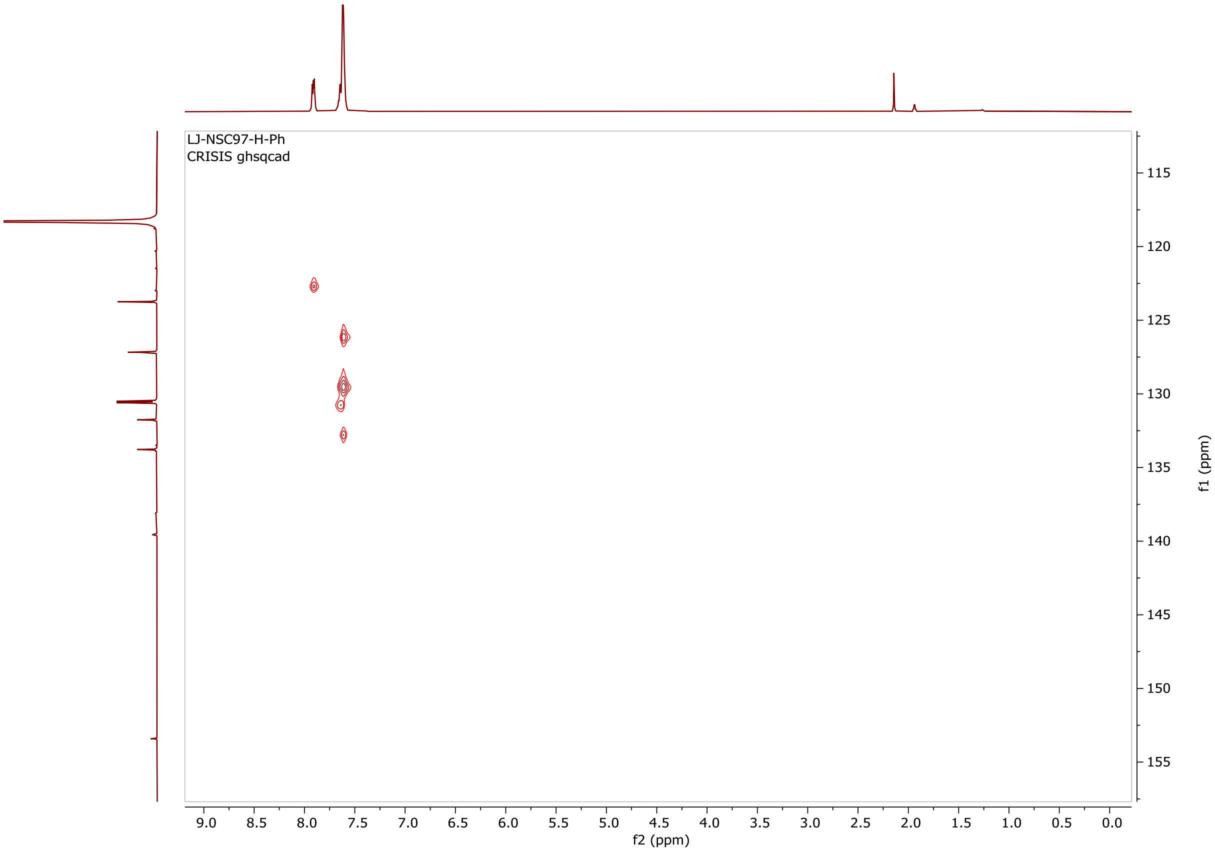

Figure S167: HSQC [^{1}H ↔^{13}C, (399.78 MHz /100.54 MHz, CD_3CN, 295 K) of **F-(*N*Ph)PAP**.

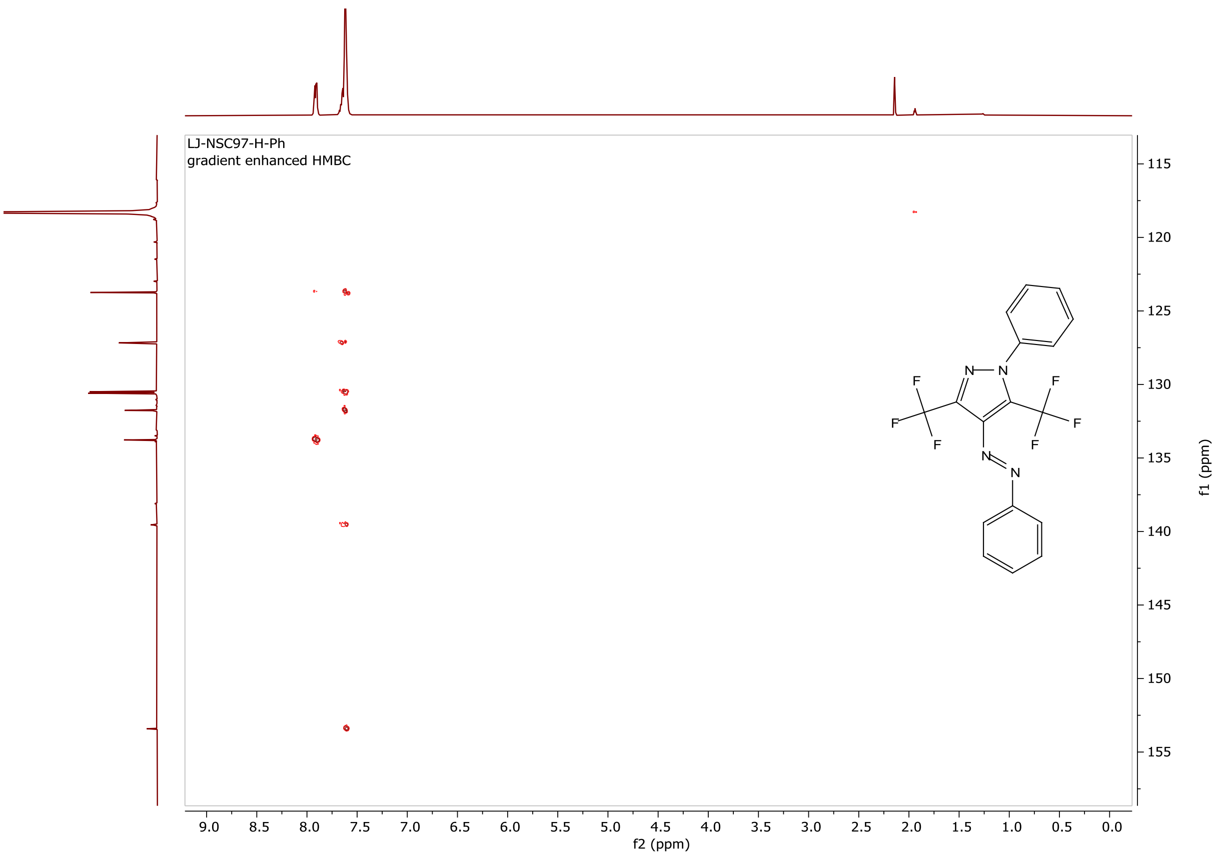

Figure S168: HMBC [^{1}H ↔ ^{13}C, (399.78 MHz /100.54 MHz, CD_3CN, 295 K) of **F-(*N*Ph)PAP**.

F-(*N*)PAP-*n*Bu: (*E*)-4-((4-Butylphenyl)diazenyl-3,5-bis(trifluoromethyl)-1*H*-pyrazole

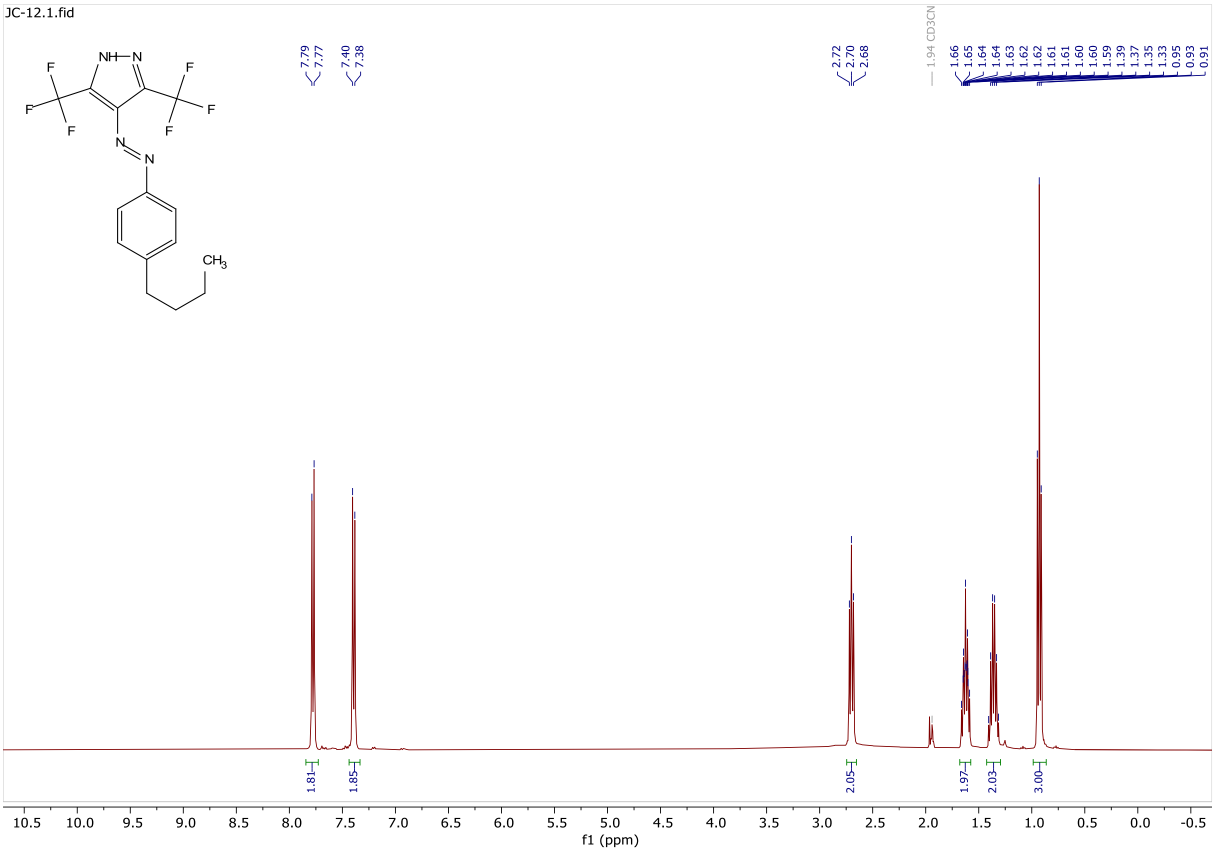

Figure S169: ^{1}H-NMR (400.25 MHz, CD_3CN, 295 K) of **F-(*N*)PAP-*n*Bu**.

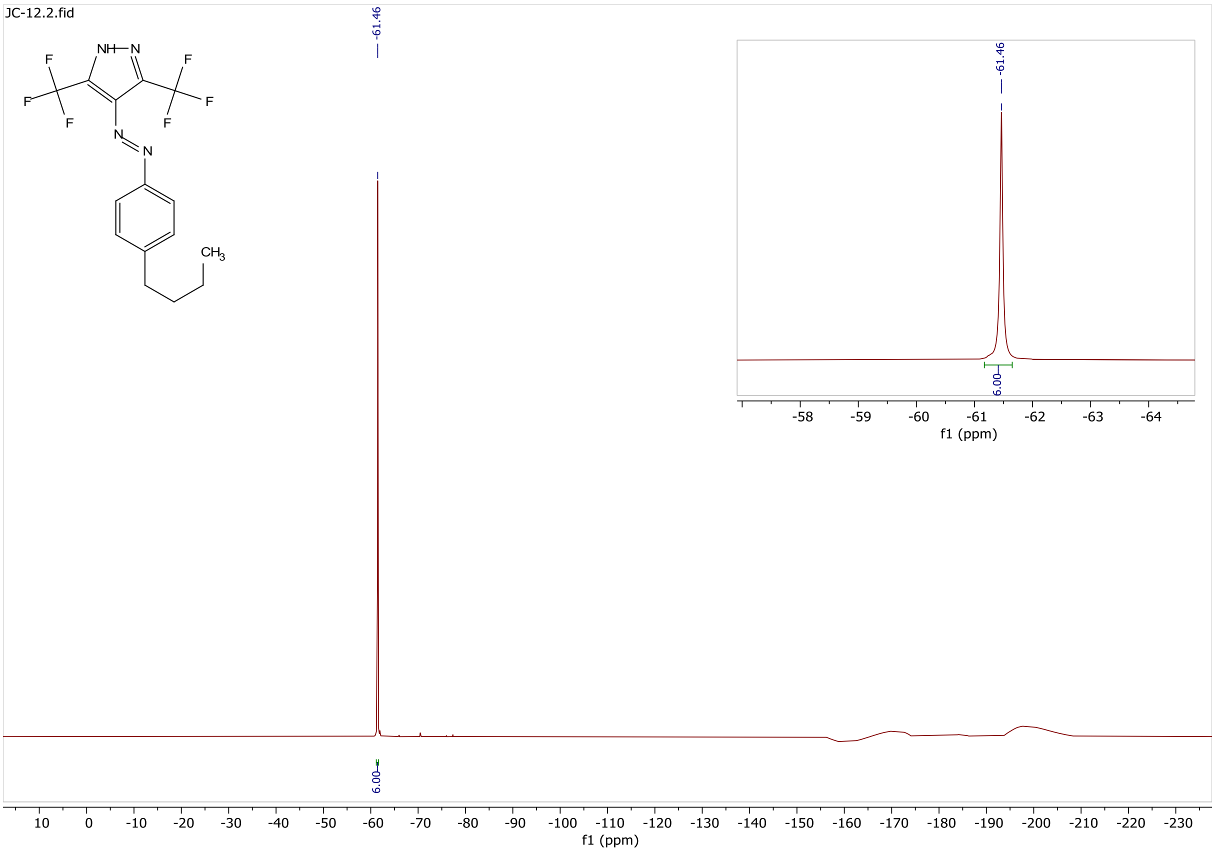

Figure S170: ^{19}F-NMR (376.57 MHz, CD_3CN, 295 K) of **F-(*N*)PAP-*n*Bu**.

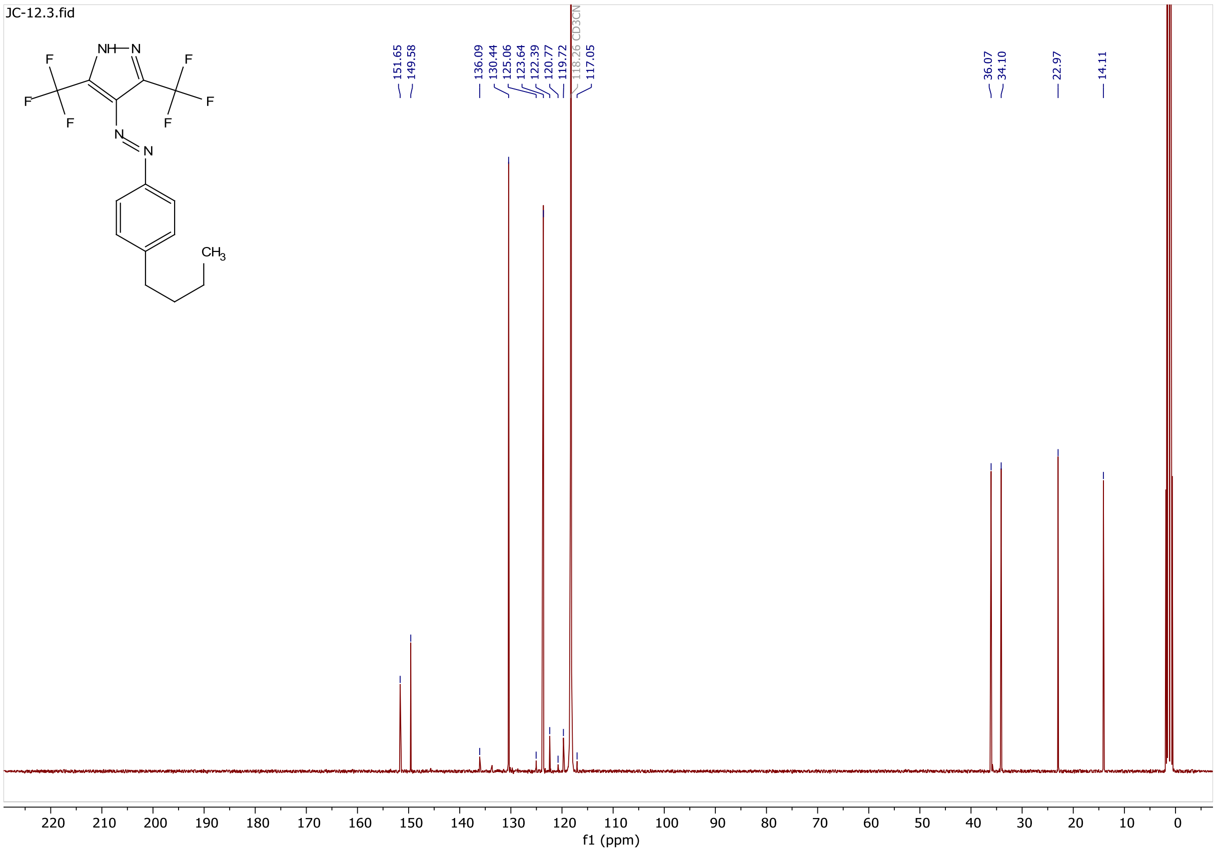

Figure S171: ^{13}C-NMR {^{1}H} (100.65 MHz, CD_3CN, 295 K) of **F-(*N*)PAP-*n*Bu**.

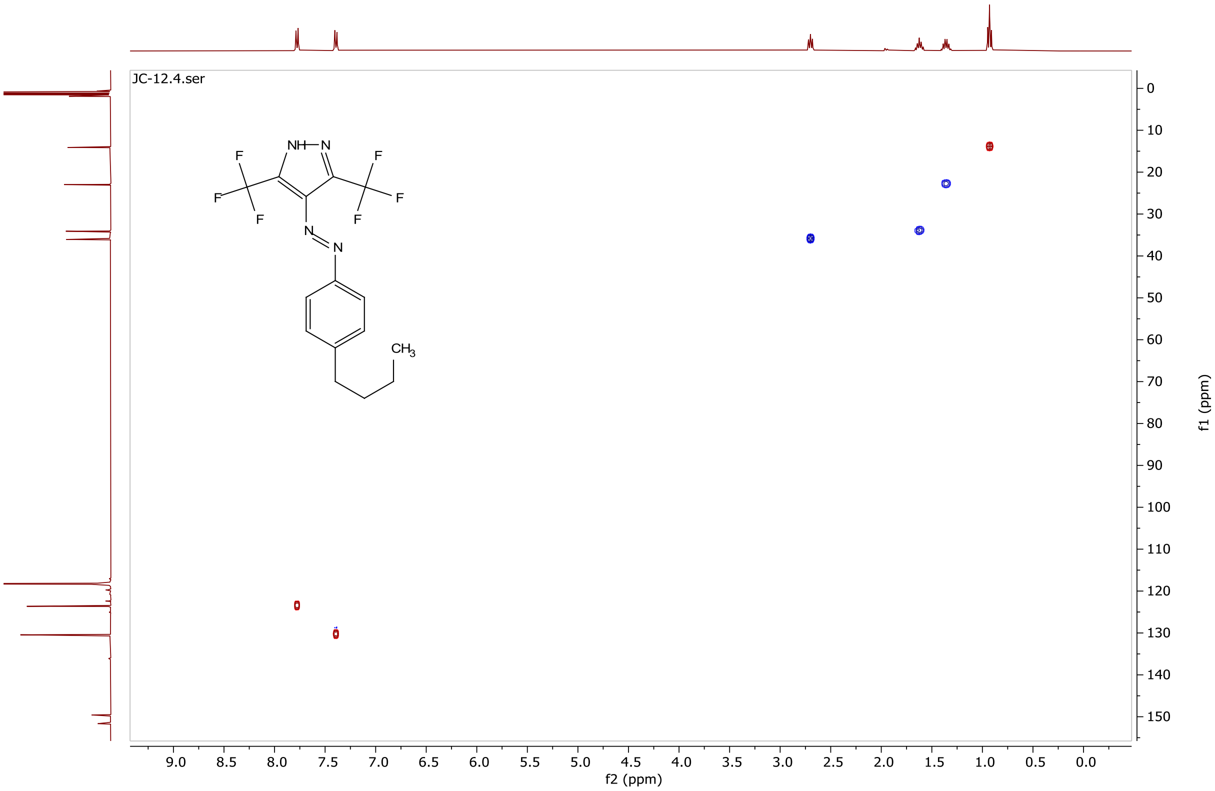

Figure S172: HSQC [^{1}H ↔^{13}C, (400.25 MHz /100.65 MHz, CD_3CN, 295 K) of **F-(*N*)PAP-*n*Bu**.

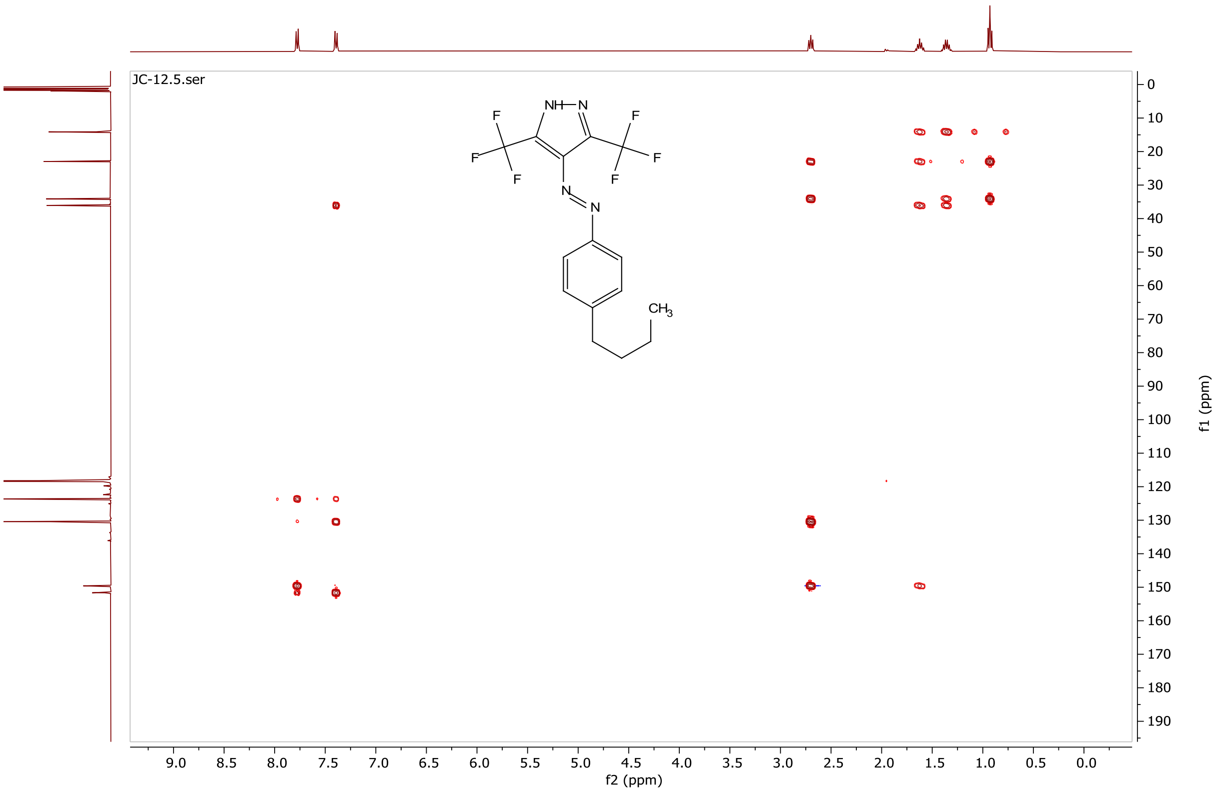

Figure S173: HMBC [$^1H \leftrightarrow ^{13}C$, (400.25 MHz /100.65 MHz, CD_3CN, 295 K) of **F-(*N*)PAP-*n*Bu**.

F-(N*n*PrCOOH)PAP-*n*Bu: (*E*)-4-(4-((4-Butylphenyl)diazenyl-3,5-bis(trifluoromethyl)-1*H*-pyrazolyl)butanoic acid

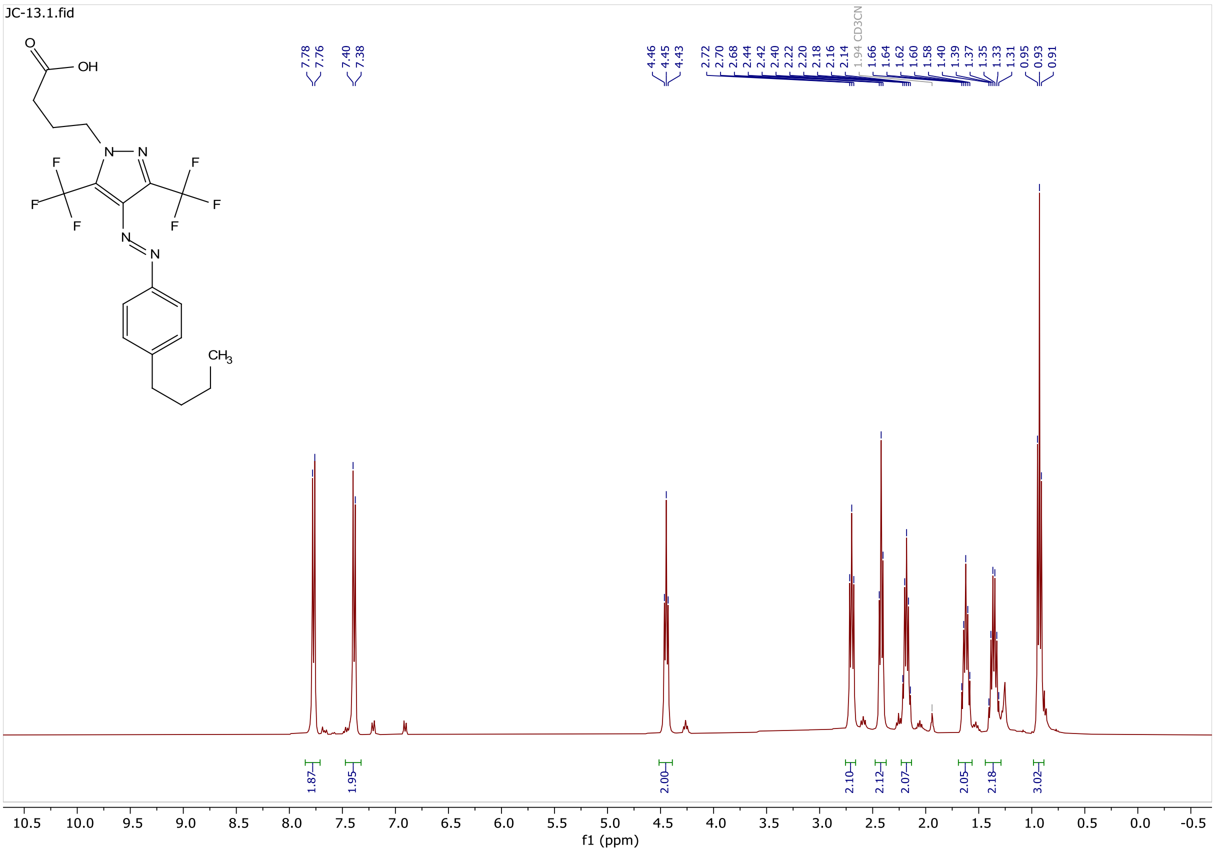

Figure S174: ^{1}H-NMR (400.25 MHz, CD_3CN, 295 K) of **F-(N*n*PrCOOH)PAP-*n*Bu**.

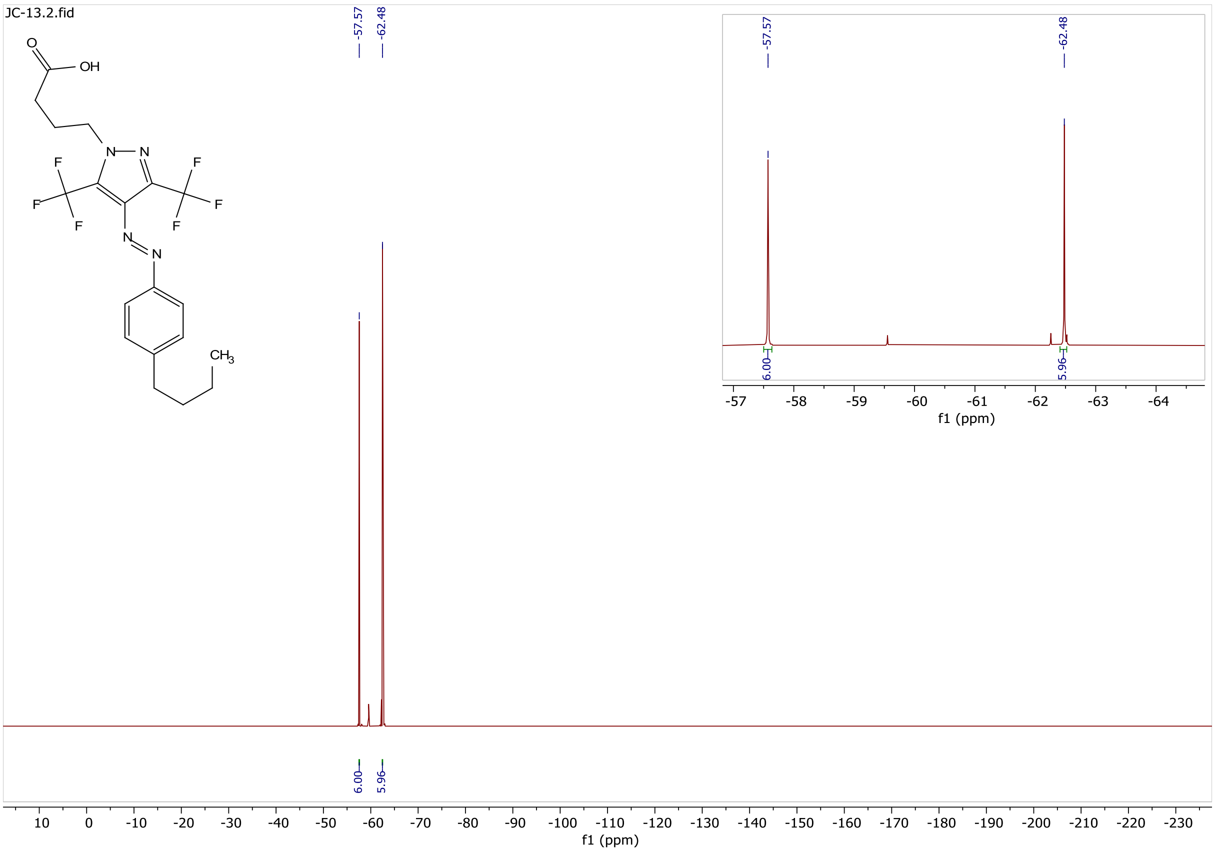

Figure S175: ^{19}F-NMR (376.57 MHz, CD_3CN, 295 K) of **F-(N*n*PrCOOH)PAP-*n*Bu**.

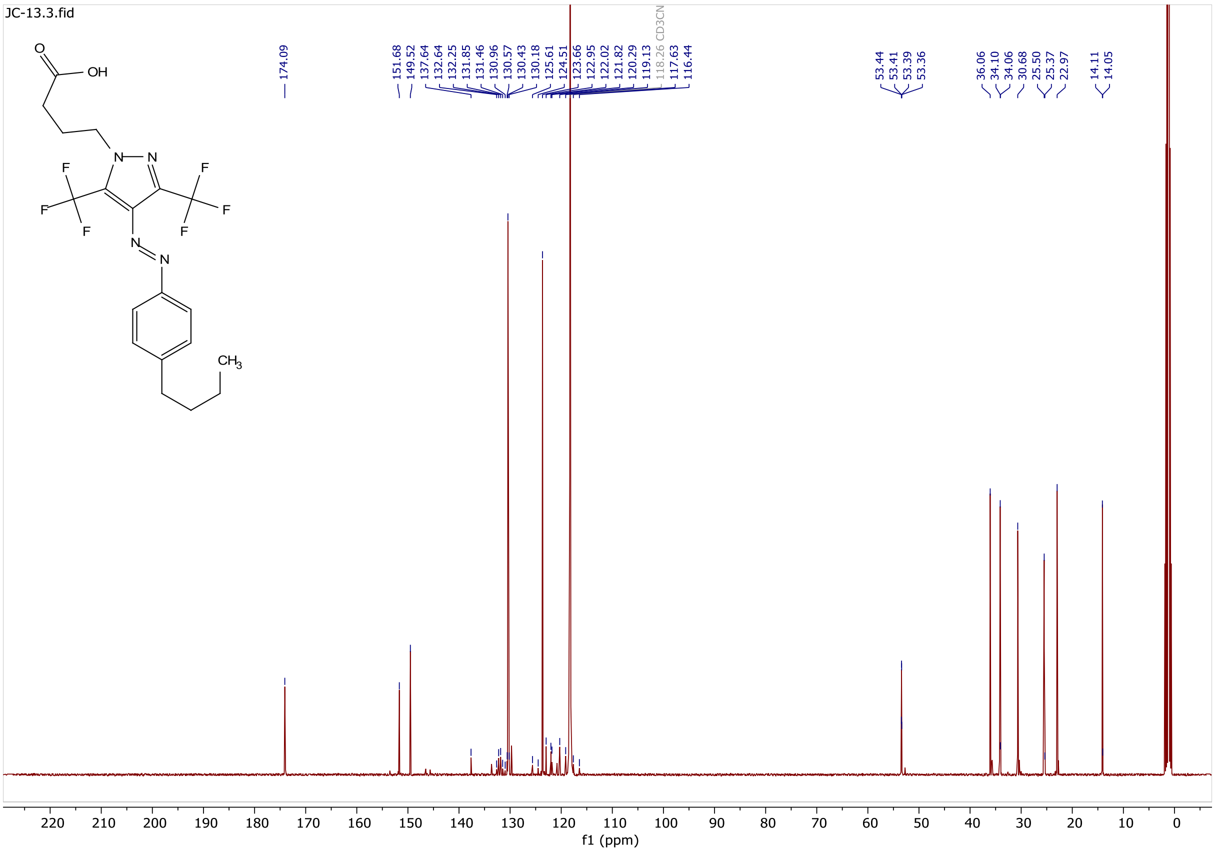

Figure S176: ^{13}C-NMR {^{1}H} (100.65 MHz, CD_3CN, 295 K) of **F-(N*n*PrCOOH)PAP-*n*Bu**.

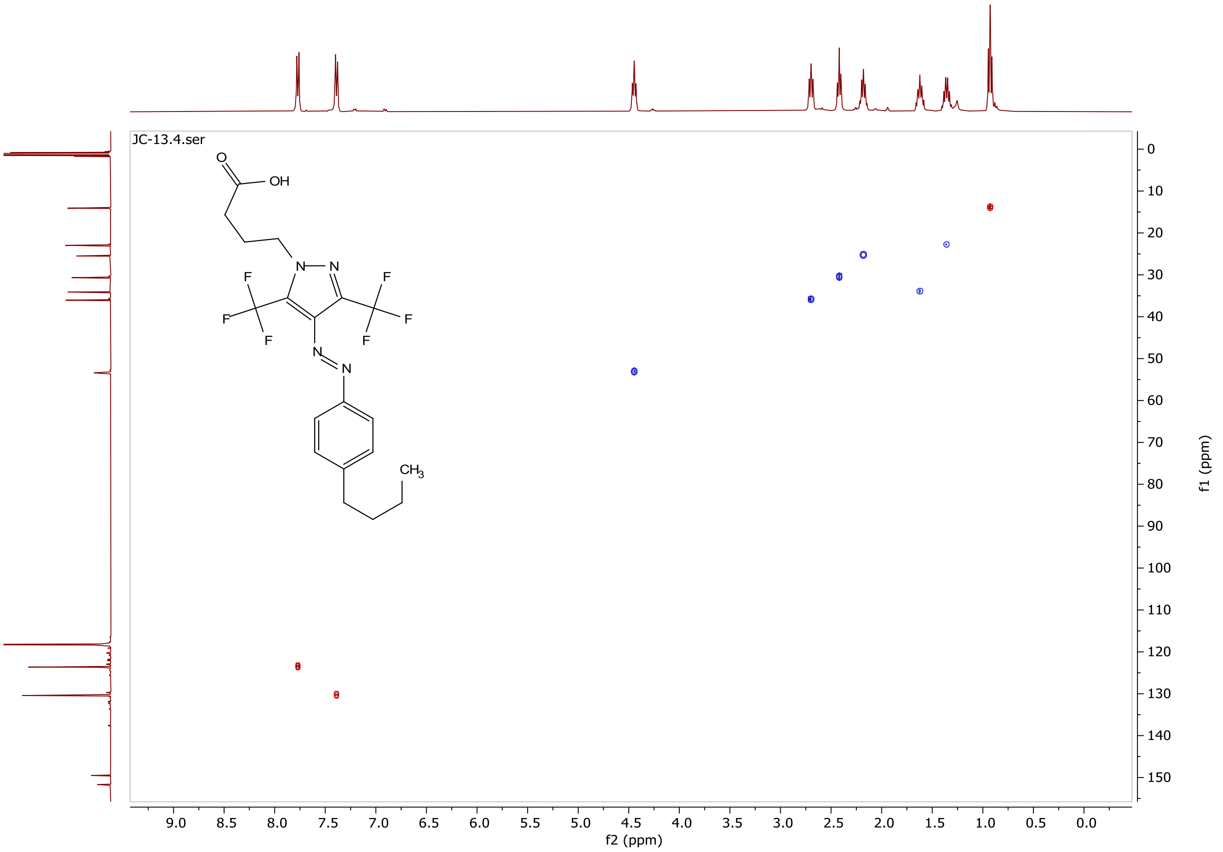

Figure S177: HSQC [$^1H \leftrightarrow ^{13}C$, (400.25 MHz /100.65 MHz, CD_3CN, 295 K) of **F-(N*n*PrCOOH)PAP-*n*Bu**.

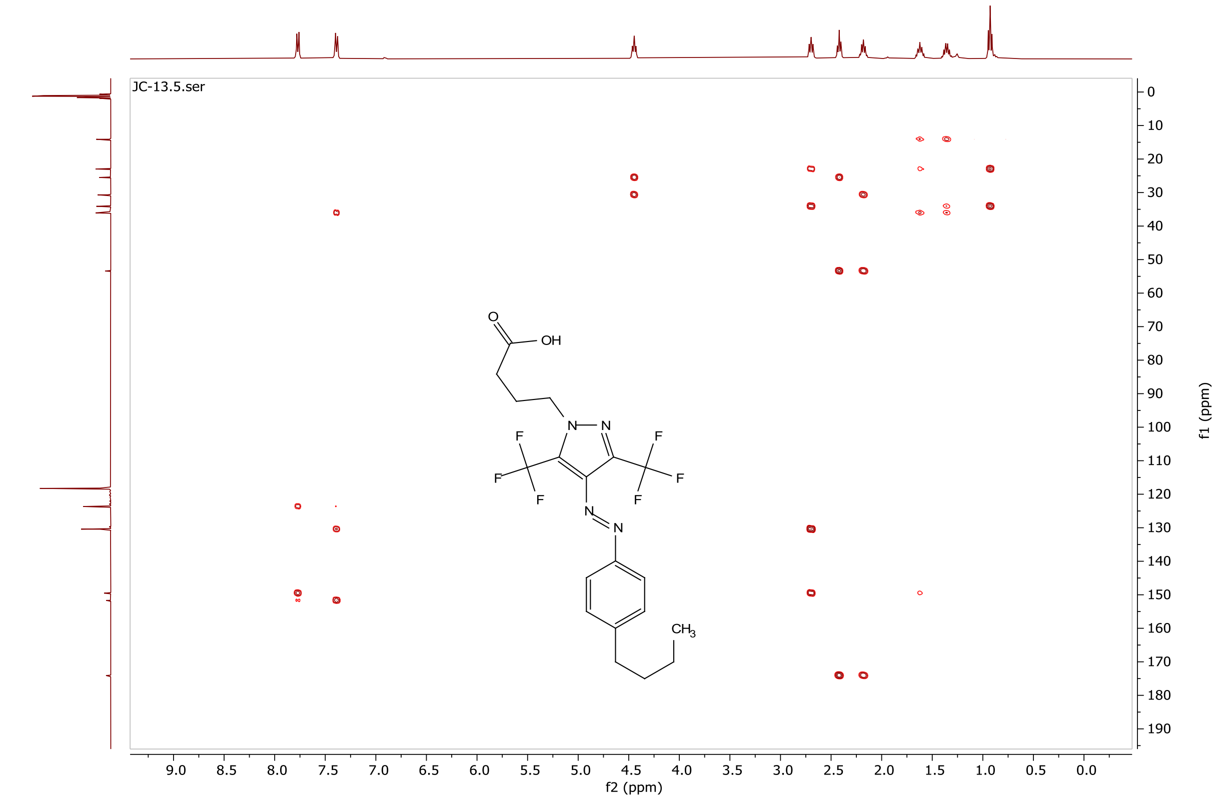

Figure S178: HMBC [$^1H \leftrightarrow ^{13}C$, (400.25 MHz /100.65 MHz, CD_3CN, 295 K) of **F-(N*n*PrCOOH)PAP-*n*Bu**

7. MS Data

F-PAP-H: (*E*)-1-Methyl-4-(phenyldiazenyl)-3,5-bis(trifluoromethyl)-1*H*-pyrazole

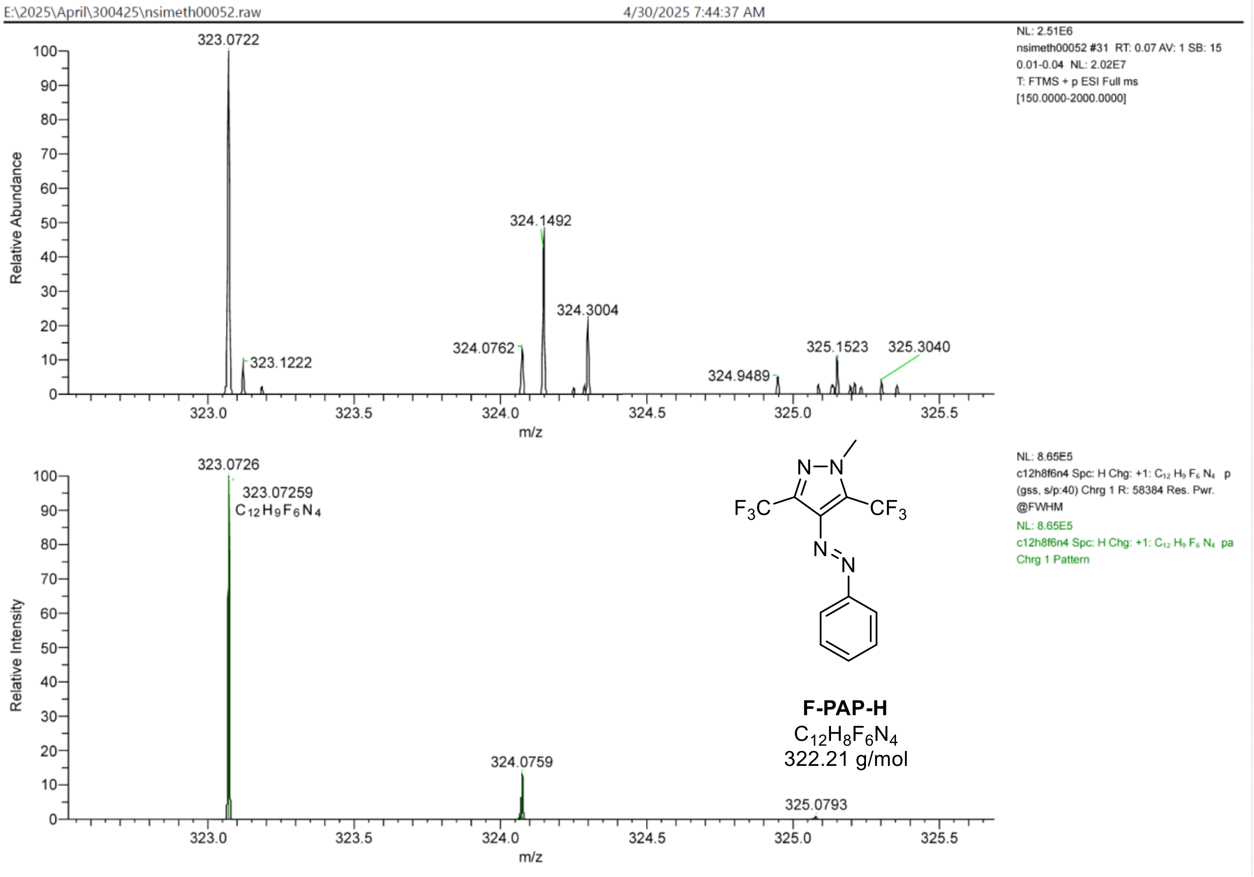

Figure S179: HRMS of **F-PAP-H**. The upper trace shows the measured spectrum, the lower trace a simulated spectrum for the specified molecular formula.

F-PAP-Cl: (*E*)-4-((4-Chlorophenyl)diazenyl)-1-methyl-3,5-bis(trifluoromethyl)-1*H*-pyrazole

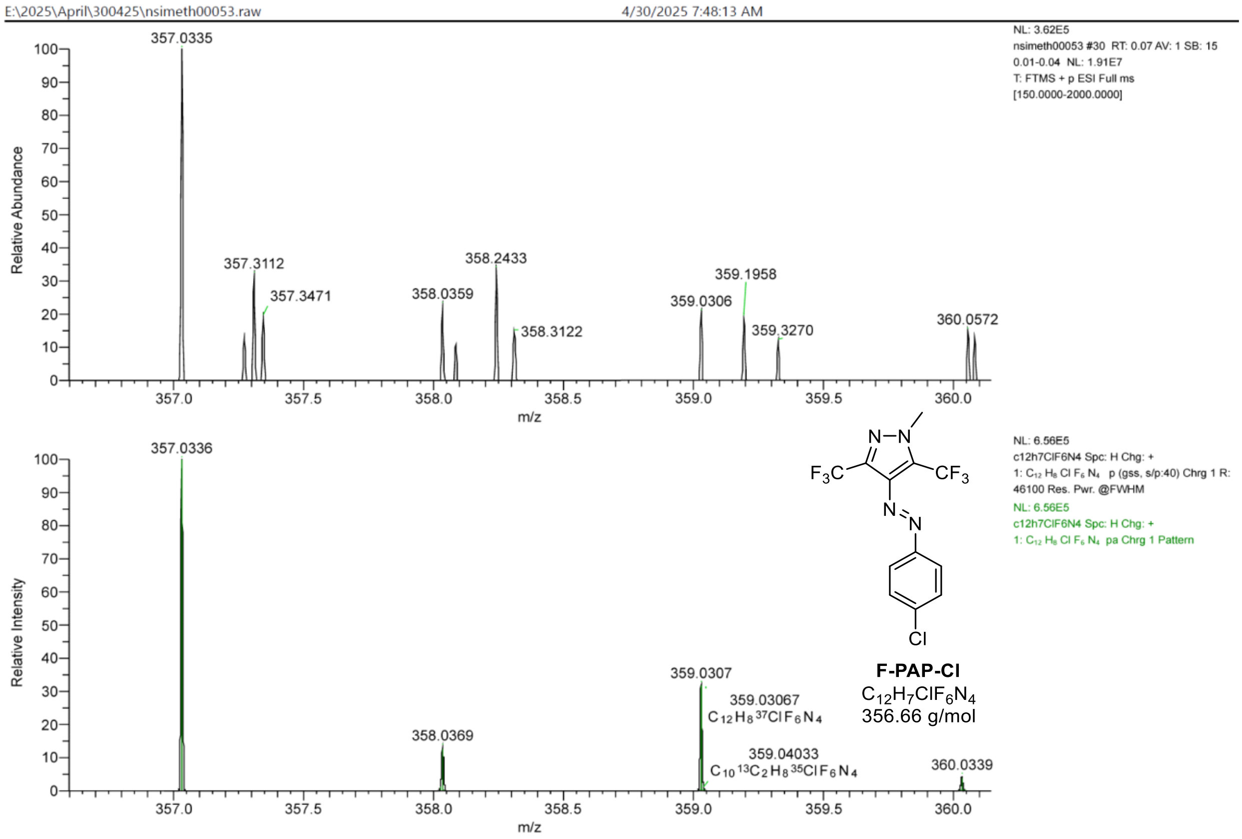

Figure S180: HRMS of **F-PAP-Cl**. The upper trace shows the measured spectrum, the lower trace a simulated spectrum for the specified molecular formula.

F-PAP-Br: (*E*)-4-((4-Bromophenyl)diazenyl)-1-methyl-3,5-bis(trifluoromethyl)-1*H*-pyrazole

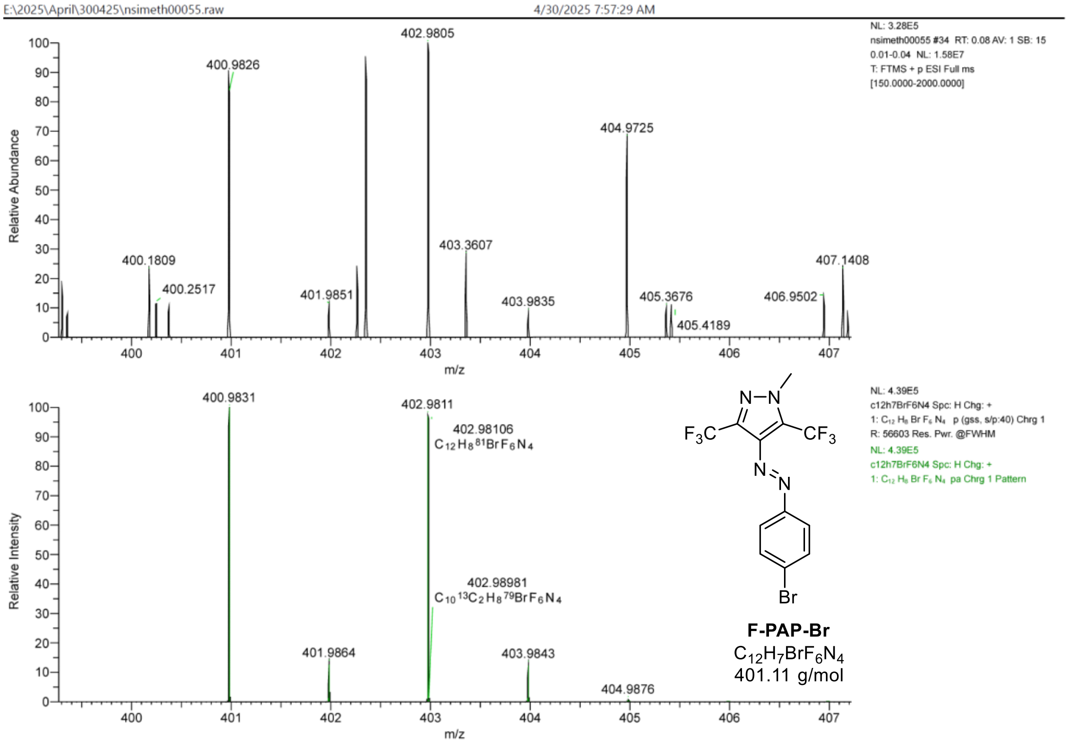

Figure S181: HRMS of **F-PAP-Br**. The upper trace shows the measured spectrum, the lower trace a simulated spectrum for the specified molecular formula.

F-PAP-CN: (*E*)-4-((1-Methyl-3,5-bis(trifluoromethyl)-1*H*-pyrazol-4-yl)diazenyl)benzonitrile

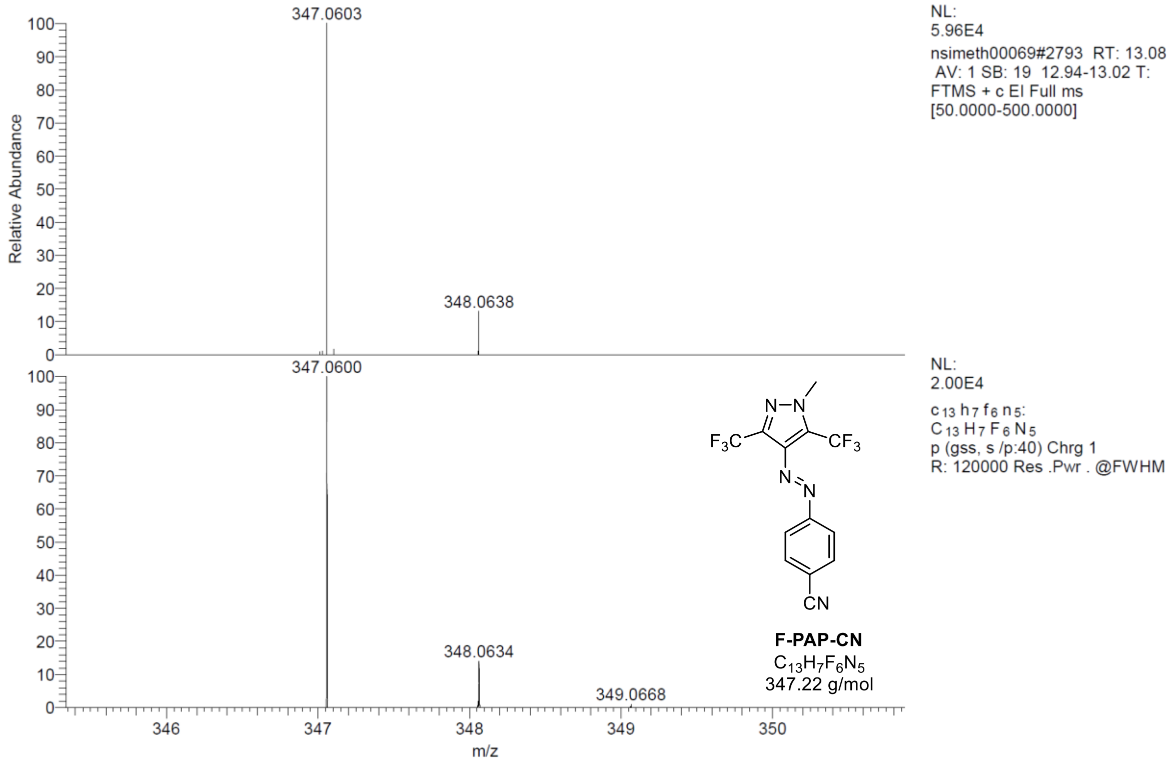

Figure S182: HRMS of **F-PAP-CN**. The upper trace shows the measured spectrum, the lower trace a simulated spectrum for the specified molecular formula.

F-PAP-Me: (*E*)-1-Methyl-4-(p-tolyldiazenyl)-3,5-bis(trifluoromethyl)-1*H*-pyrazole

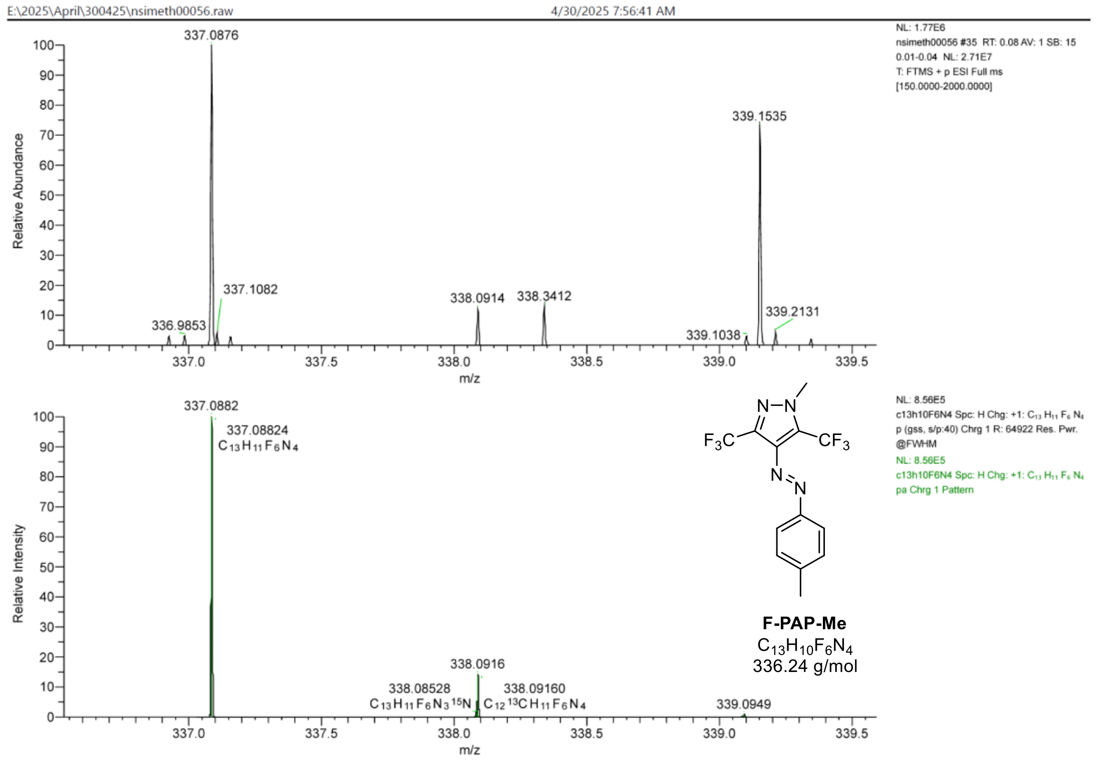

Figure S183: HRMS of **F-PAP-Me**. The upper trace shows the measured spectrum, the lower trace a simulated spectrum for the specified molecular formula.

F-PAP-CF₃: (*E*)-1-Methyl-3,5-bis(trifluoromethyl)-4-((4 (trifluoromethyl)phenyl)diazenyl)-1*H*-pyrazole

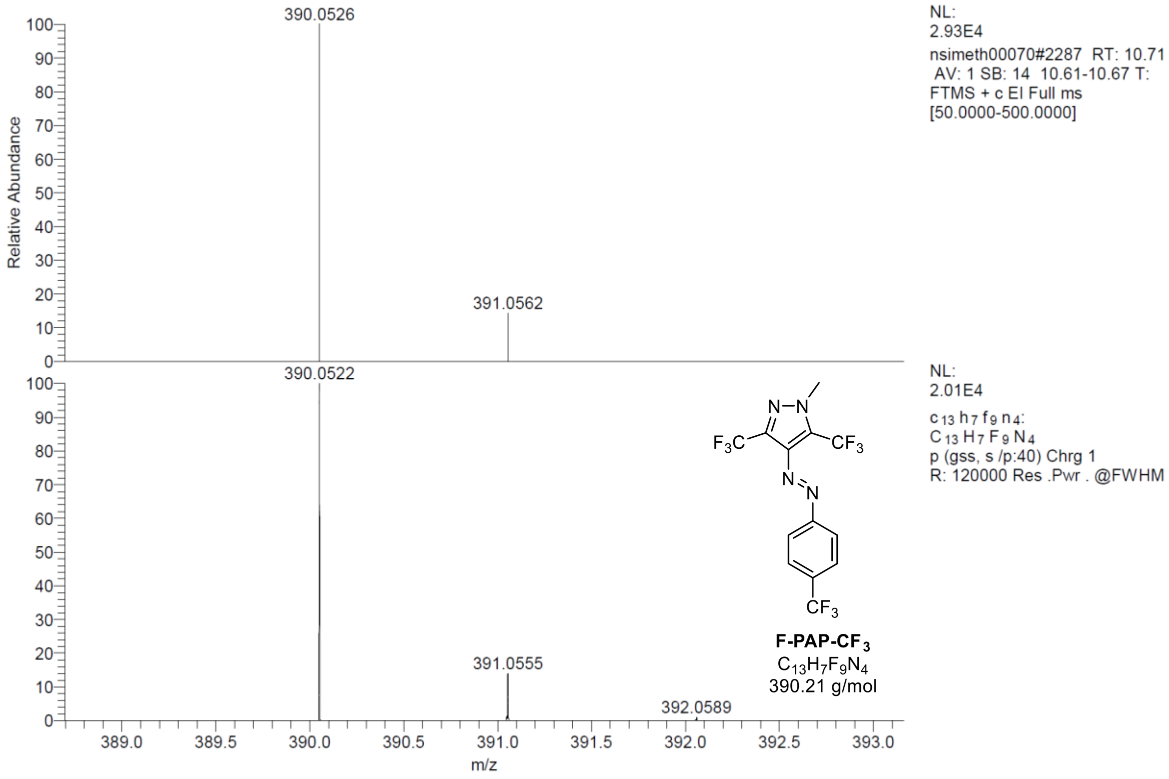

Figure S184: HRMS of **F-PAP-CF$_3$**. The upper trace shows the measured spectrum, the lower trace a simulated spectrum for the specified molecular formula.

F-PAP-OMe: (*E*)-4-((4-Methoxyphenyl)diazenyl)-1-methyl-3,5-bis(trifluoromethyl)-1*H*-pyrazole

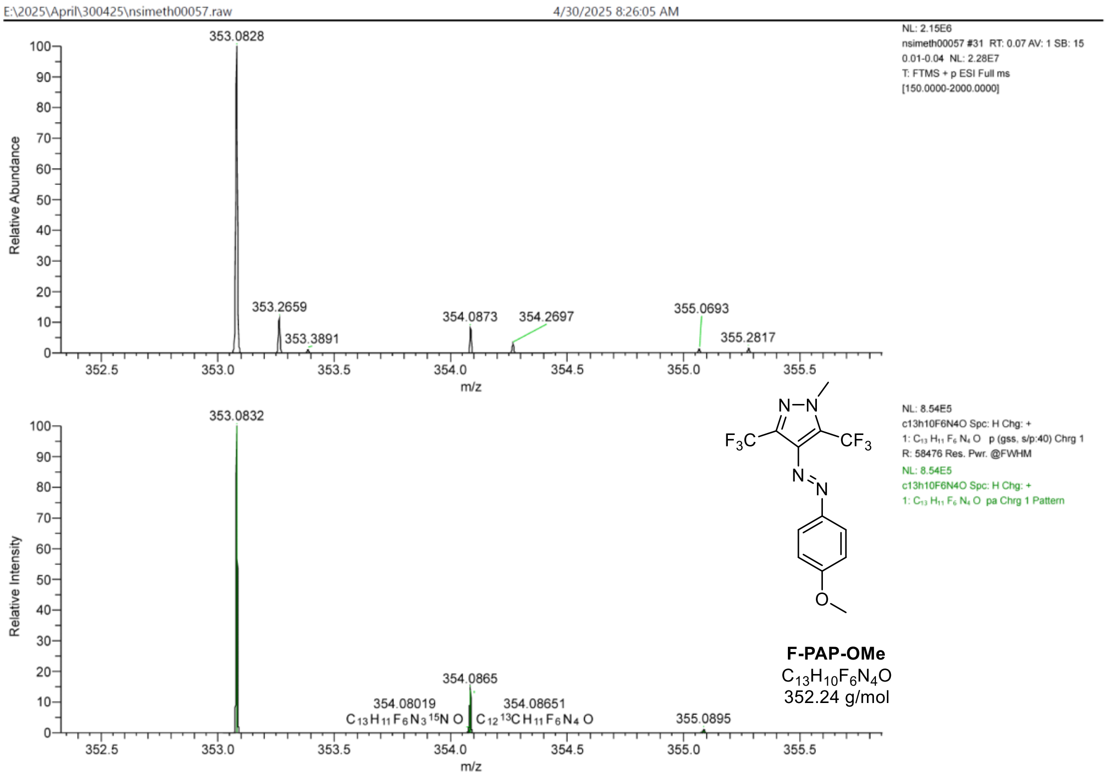

Figure S185: HRMS of **F-PAP-OMe**. The upper trace shows the measured spectrum, the lower trace a simulated spectrum for the specified molecular formula.

F-PAP-OCF$_3$: (*E*)-1-Methyl-4-((4-(trifluoromethoxy)phenyl)diazenyl)-3,5-bis(trifluoromethyl)-1*H*-pyrazole

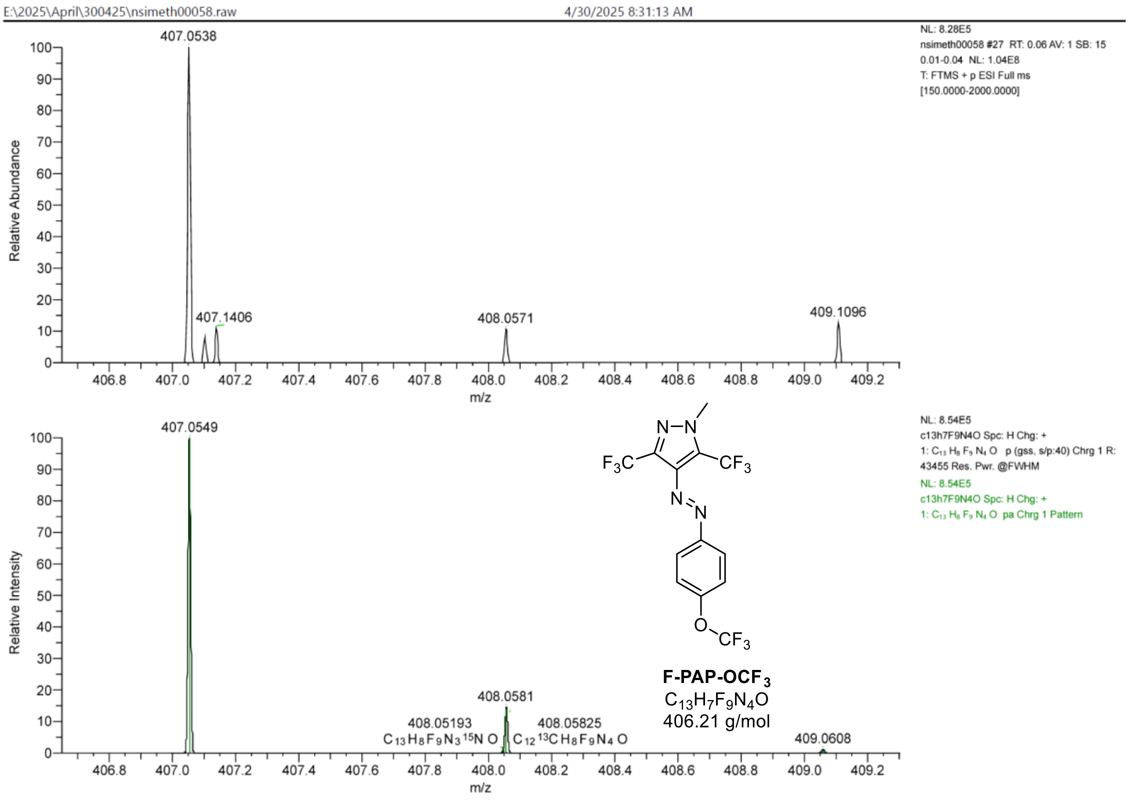

Figure S186: HRMS of **F-PAP-OCF$_3$**. The upper trace shows the measured spectrum, the lower trace shows a simulated spectrum for the indicated mass.

F-PAP-CCH: (*E*)-4-((4-Ethynylphenyl)diazenyl)-1-methyl-3,5-bis(trifluoromethyl)-1*H*-pyrazole

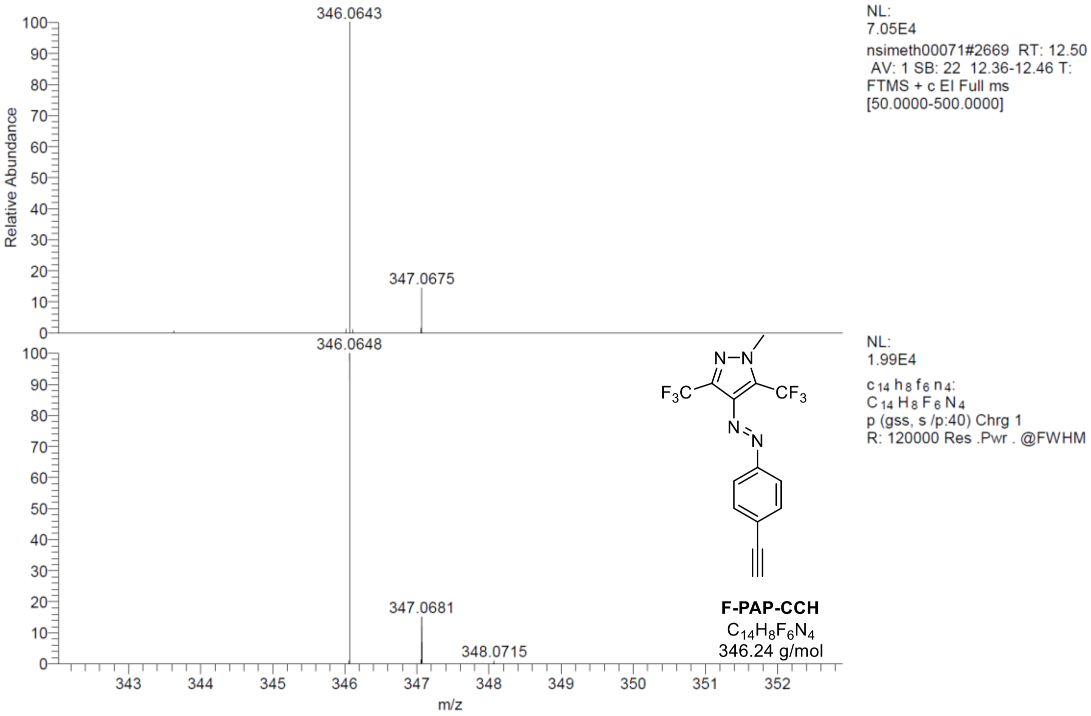

Figure S187: HRMS of **F-PAP-CCH**. The upper trace shows the measured spectrum, the lower trace shows a simulated spectrum for the indicated mass.

F-PAP-*n*Bu: (*E*)-4-((4-Butylphenyl)diazenyl)-1-methyl-3,5-bis(trifluoromethyl)-1*H*-pyrazole

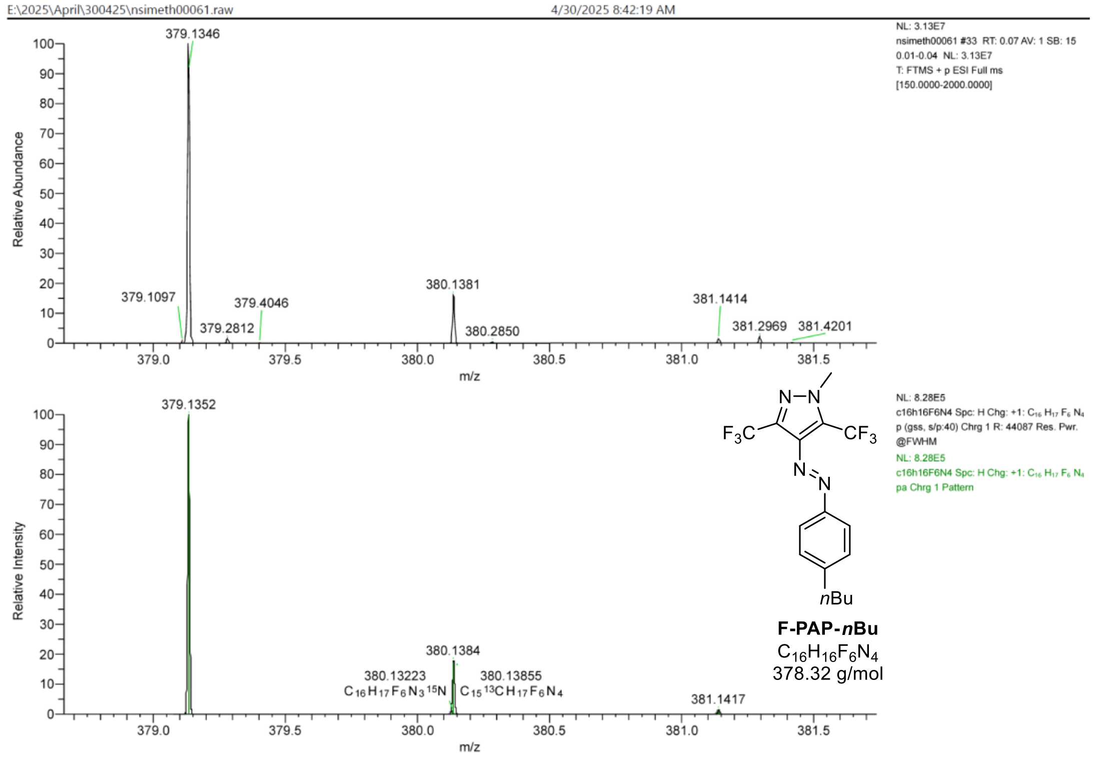

Figure S188: HRMS of **F-PAP-*n*Bu**. The upper trace shows the measured spectrum, the lower trace shows a simulated spectrum for the indicated mass.

F-PAP-C_3H_6COOH: (*E*)-4-(4-((1-Methyl-3,5-bis(trifluoromethyl)-1*H*-pyrazol-4-yl)diazenyl)phenyl) butanoic acid

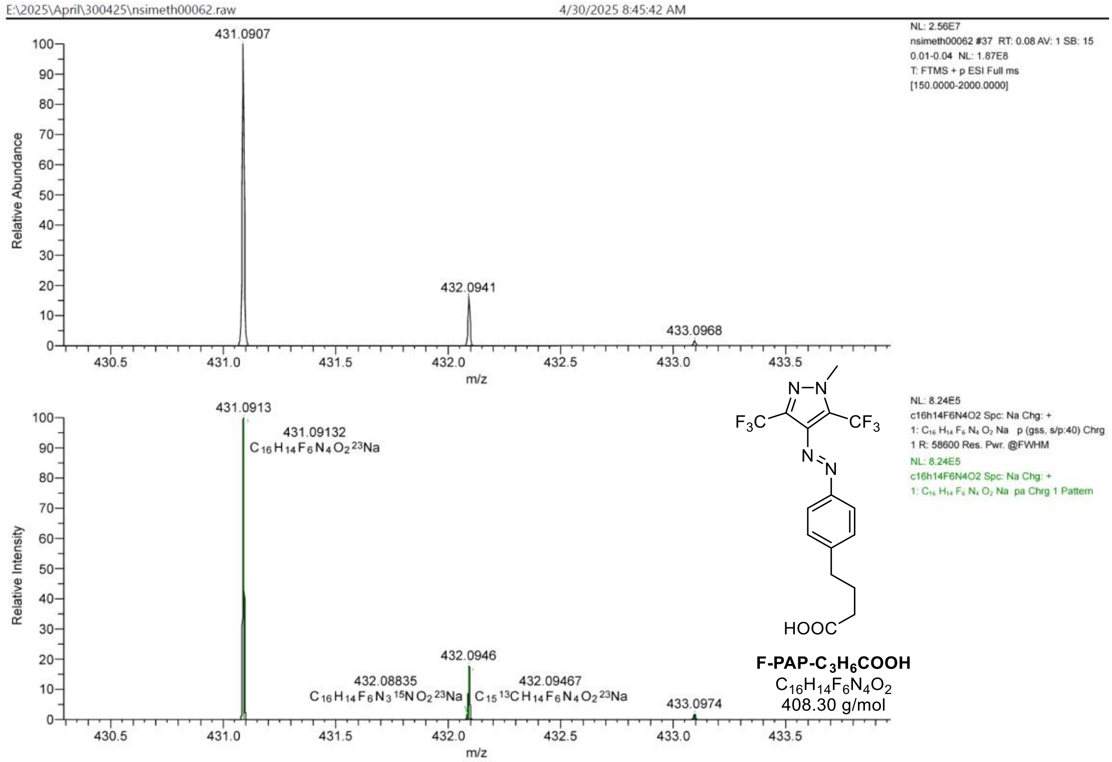

Figure S189: HRMS of **F-PAP-C_3H_6COOH**. The upper trace shows the measured spectrum, the lower trace shows a simulated spectrum for the indicated mass.

F-PAP-NO$_2$: (*E*)-1-Methyl-4-((4-nitrophenyl)diazenyl)-3,5-bis(trifluoromethyl)-1*H*-pyrazole

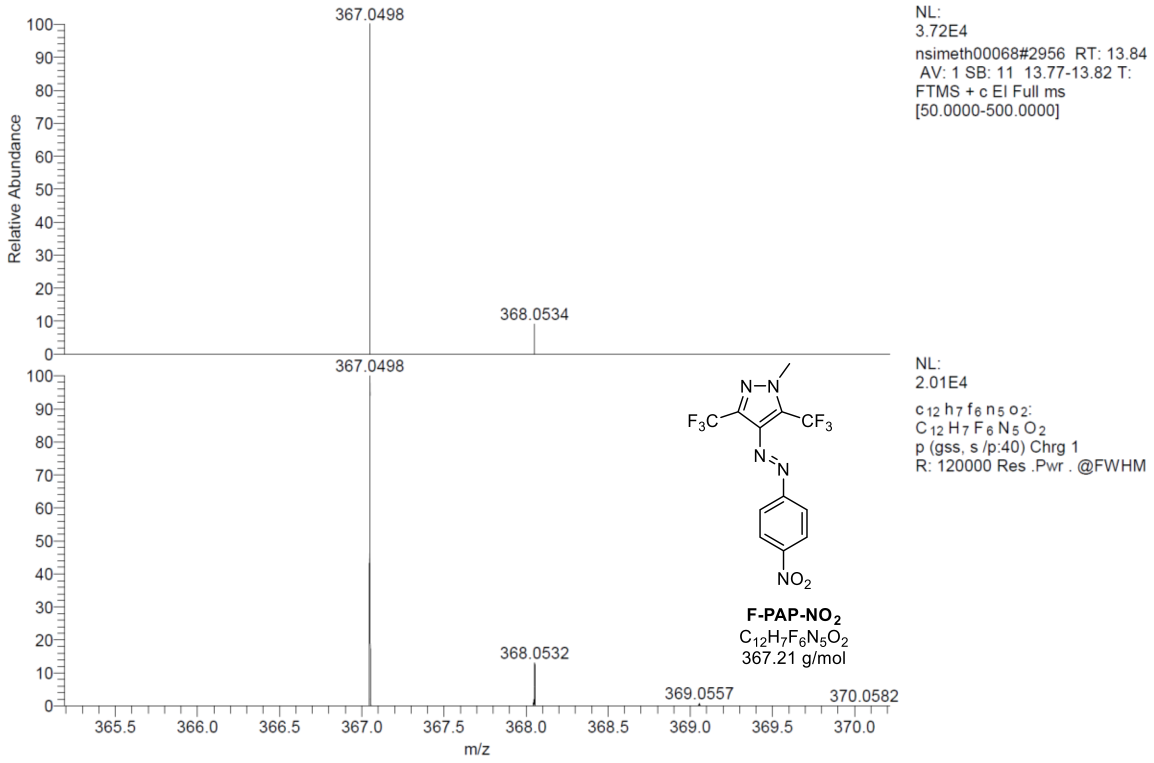

Figure S190: HRMS of **F-PAP-NO$_2$**. The upper trace shows the measured spectrum, the lower trace shows a simulated spectrum for the indicated mass.

F-(*N*Ph)PAP: (*E*)-1-Phenyl-4-(phenyldiazenyl)-3,5-bis(trifluoromethyl)-1*H*-pyrazole

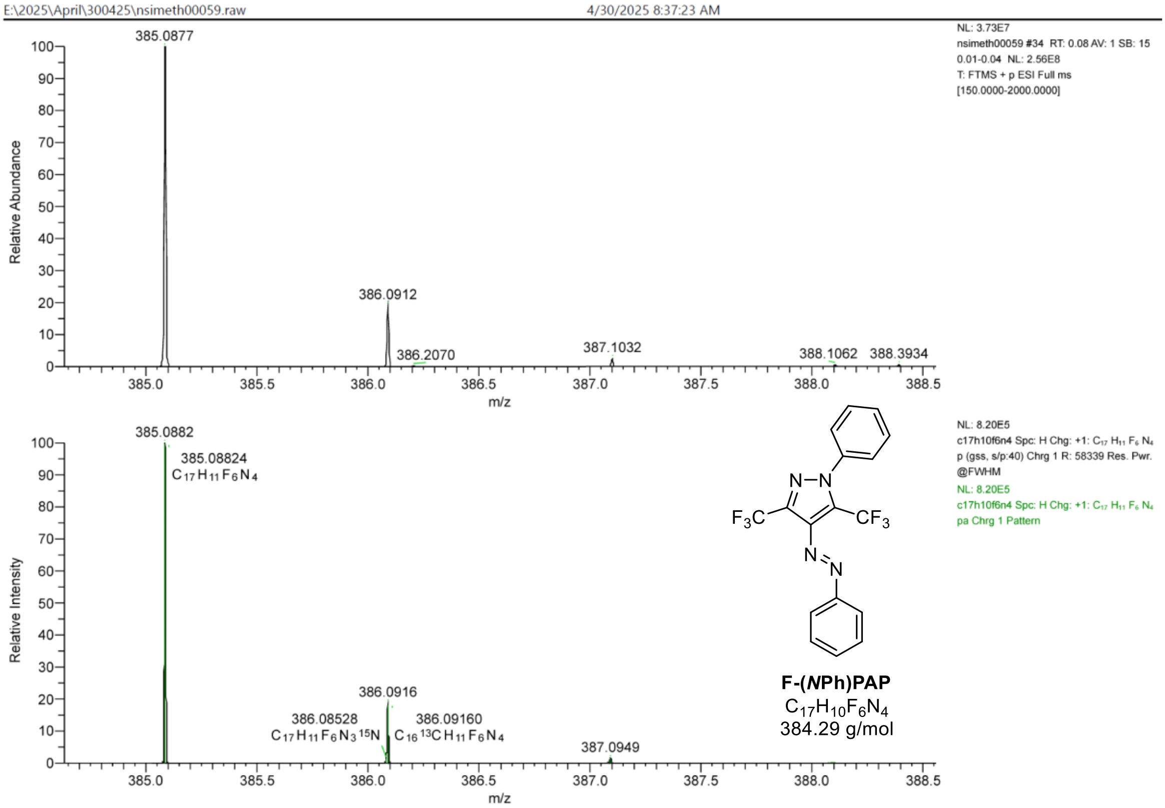

Figure S191: HRMS of **F-(*N*Ph)PAP**. The upper trace shows the measured spectrum, the lower trace shows a simulated spectrum for the indicated mass.

F-(*N*)PAP-*n*Bu: (*E*)-4-((4-Butylphenyl)diazenyl-3,5-bis(trifluoromethyl)-1*H*-pyrazole

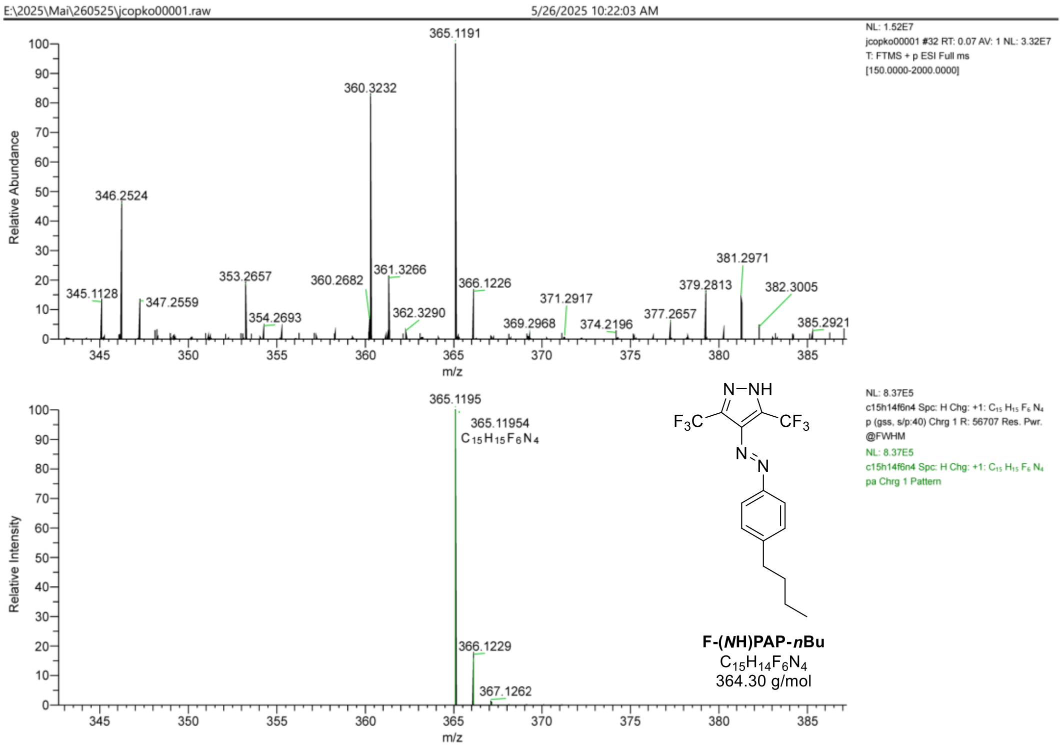

Figure S192: HRMS of **F-(N*H*)PAP-*n*Bu**. The upper trace shows the measured spectrum, the lower trace shows a simulated spectrum for the indicated mass.

F-(N*n*PrCOOH)PAP-*n*Bu: (*E*)-4-(4-((4-Butylphenyl)diazenyl-3,5-bis(trifluoromethyl)-1*H*-pyrazolyl)butanoic acid

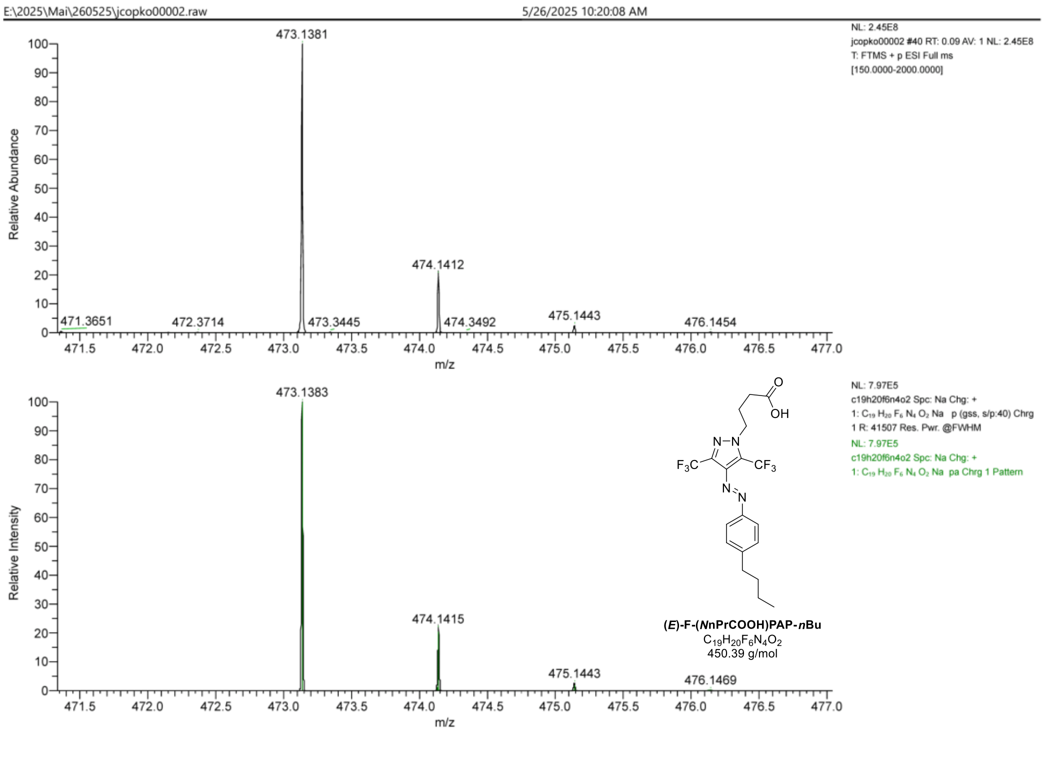

Figure S193: HRMS of **F-(N*n*PrCOOH)PAP-*n*Bu**. The upper trace shows the measured spectrum, the lower trace shows a simulated spectrum for the indicated mass.

8. X-Ray Data

Single crystals were mounted on a fiber loop and fixated using Fomblin oil. The data was collected on an XtaLAB Synergy, with CuK_α (λ = 1.54184) radiation and a HyPix detector. The crystal was kept at 274(19) K during data collection. Using Olex2,[8] the structure was solved with the SHELXT[9] structure solution program using Intrinsic Phasing and refined with the SHELXL[10] refinement package using Least Squares minimization. All the non-hydrogen atoms were refined using an anisotropic model, and all the hydrogen atoms were constrained in geometrical positions to their parent atom. Crystallographic data are presented in Table S1 and Table S2. Deposition numbers CCDC 2453602 and 2453603 contain the supplementary crystallographic data for this paper. These data can be obtained free of charge via www.ccdc.cam.ac.uk/data_request/cif, or by emailing data_request@ccdc.cam.ac.uk, or by contacting The Cambridge Crystallographic Data Centre, 12 Union Road, Cambridge CB21EZ, UK; fax: +441223336033.

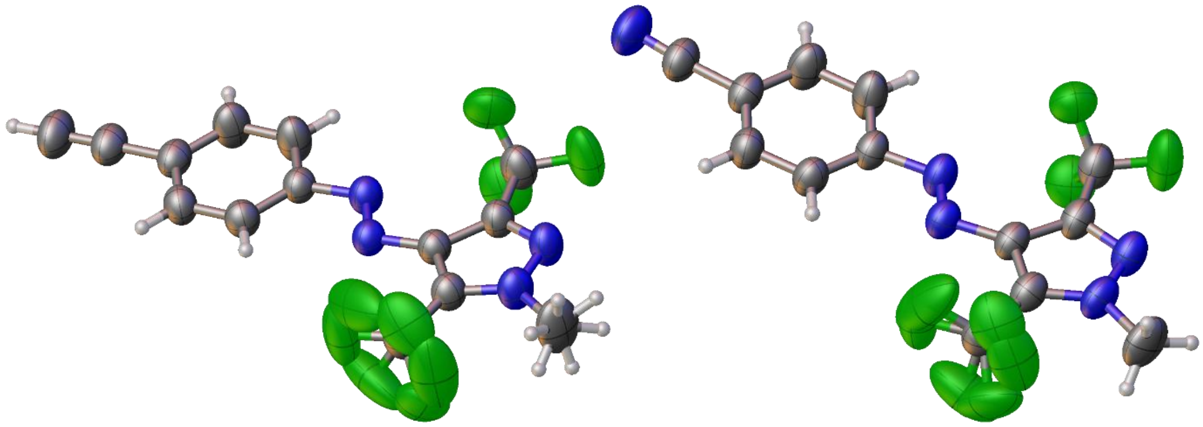

Figure S194: Crystal structure **F-PAP-CCH** (left) and **F-PAP-CN** (right). The thermal ellipsoids are drawn at the 50% probability level.

Table S1: Crystal data and structure refinement for F-PAP-CCH.

CCDC	2453602
Empirical formula	$C_{14}H_{8}F_{6}N_{4}$
Formula weight	346.24
Temperature/K	274(19)
Crystal system	monoclinic
Space group	C2/*m*
a/Å	16.5357(3)
b/Å	6.91550(10)
c/Å	14.0544(3)
α/°	90
β/°	109.314(2)
γ/°	90
Volume/Å^3	1516.71(5)
Z	4
ρ_{calc}g/cm^3	1.516
μ/mm^{-1}	1.304
F(000)	696.0
Crystal size/mm^3	0.39 × 0.31 × 0.08
Radiation	Cu K_{α} (λ = 1.54184)
2Θ range for data collection/°	6.664 to 156.94
Index ranges	-17 ≤ h ≤ 20, -8 ≤ k ≤ 8, -17 ≤ l ≤ 17
Reflections collected	18435
Independent reflections	1735 [R_{int} = 0.0286, R_{sigma} = 0.0123]
Data/restraints/parameters	1735/0/156
Goodness-of-fit on F^2	1.101
Final R indexes [I>=2σ (I)]	R_1 = 0.0444, wR_2 = 0.1320
Final R indexes [all data]	R_1 = 0.0486, wR_2 = 0.1356
Largest diff. peak/hole / e Å^{-3}	0.17/-0.18

Table S2 Crystal data and structure refinement for F-PAP-CN.

CCDC	2453603
Empirical formula	$C_{13}H_7F_6N_5$
Formula weight	347.24
Temperature/K	301.14(15)
Crystal system	triclinic
Space group	*P*-1
a/Å	8.5808(3)
b/Å	8.8989(3)
c/Å	11.0095(6)
α/°	84.394(4)
β/°	75.131(4)
γ/°	62.210(4)
Volume/Å^3	718.58(6)
Z	2
ρ_{calc}g/cm^3	1.605
μ/mm^{-1}	1.399
F(000)	348.0
Crystal size/mm^3	0.71 × 0.1 × 0.04
Radiation	Cu K_α (λ = 1.54184)
2Θ range for data collection/°	8.312 to 157.074
Index ranges	-10 ≤ h ≤ 10, -9 ≤ k ≤ 11, -13 ≤ l ≤ 13
Reflections collected	31265
Independent reflections	2925 [R_{int} = 0.0337, R_{sigma} = 0.0125]
Data/restraints/parameters	2925/3/246
Goodness-of-fit on F^2	1.070
Final R indexes [I>=2σ (I)]	R_1 = 0.0440, wR_2 = 0.1252
Final R indexes [all data]	R_1 = 0.0498, wR_2 = 0.1303
Largest diff. peak/hole / e Å^{-3}	0.15/-0.23

9. References

[1] A. Volker, J. D. Steen, S. Crespi, *Beilstein J. Org. Chem.* **2024**, *20*, 1684–1692.
[2] R. Tovtik, D. Marzin, P. Weigel, S. Crespi, N. A. Simeth, *Beilstein J. Org. Chem.* **2025**, *21*, 830–838.
[3] J. J. Snellenburg, S. P. Laptenok, R. Seger, K. M. Mullen, I. H. M. V. Stokkum, *J. Stat. Softw.* **2012**, *49*, DOI 10.18637/jss.v049.i03.
[4] L. Stricker, E.-C. Fritz, M. Peterlechner, N. L. Doltsinis, B. J. Ravoo, *J. Am. Chem. Soc.* **2016**, *138*, 4547–4554.
[5] P. Kumar, A. Srivastava, C. Sah, S. Devi, S. Venkataramani, *Chem. – Eur. J.* **2019**, *25*, 11924–11932.
[6] B. C. Enache, A. Hanganu, C. Tablet, C. C. Anghel, C. C. Popescu, A. Paun, N. D. Hădade, A. M. Mădălan, M. Matache, *ACS Omega* **2022**, *7*, 39122–39135.
[7] O. G. Khudina, E. V. Shchegol'kov, Ya. V. Burgart, M. I. Kodess, O. N. Kazheva, A. N. Chekhlov, G. V. Shilov, O. A. Dyachenko, V. I. Saloutin, O. N. Chupakhin, *J. Fluor. Chem.* **2005**, *126*, 1230–1238.
[8] O. V. Dolomanov, L. J. Bourhis, R. J. Gildea, J. A. K. Howard, H. Puschmann, *J. Appl. Crystallogr.* **2009**, *42*, 339–341.
[9] G. M. Sheldrick, *Acta Crystallogr. Sect. Found. Adv.* **2015**, *71*, 3–8.
[10] G. M. Sheldrick, *Acta Crystallogr. Sect. C Struct. Chem.* **2015**, *71*, 3–8.
